# Current and Optimal Practices in Childhood Asthma Monitoring Among Multiple International Stakeholders

**DOI:** 10.1001/jamanetworkopen.2023.13120

**Published:** 2023-05-12

**Authors:** Nikolaos G. Papadopoulos, Alexander G. Mathioudakis, Adnan Custovic, Antoine Deschildre, Wanda Phipatanakul, Gary Wong, Paraskevi Xepapadaki

**Affiliations:** 1 Allergy Department, Second Paediatric Clinic, National and Kapodistrian University of Athens, Athens, Greece; 2 Division of Immunology, Immunity to Infection and Respiratory Medicine, Faculty or Biology, Medicine, and Health, The University of Manchester, Manchester, United Kingdom; 3 North West Lung Centre, Wythenshawe Hospital, Manchester University National Health Service Foundation Trust, Manchester Academic Health Science Centre, Manchester, United Kingdom; 4 National Heart and Lung Institute, Imperial College London, London, United Kingdom; 5 Center for Infection and Immunity of Lille, Institut Pasteur de Lille, Institut National de la Santé et de la Recherche Médicale, Centre National de la Recherche Scientifique, Université de Lille, Lille, France; 6 Department of Allergy and Immunology, Boston Children’s Hospital, Boston, Massachusetts; 7 Department of Pediatrics, Faculty of Medicine, The Chinese University of Hong Kong, Sha Tin, Hong Kong

## Abstract

**Importance:**

Childhood asthma control largely depends on rigorous and regular monitoring. Although various clinical parameters, biomarkers, and patient-reported outcomes are helpful for monitoring purposes, there is no consensus on the minimum and/or optimal set of parameters and their relative priority.

**Objective:**

To assess actual and perceived optimal childhood asthma monitoring practices used globally.

**Design, Setting, and Participants:**

This international, multistakeholder survey study surveyed health care professionals and clinical academics with a professional interest in and exposure to childhood asthma between April 12 and September 3, 2021, to test for differences between the frequency that different techniques are actually used in practice vs optimal practice, between-group differences, and differences across medical settings and country economies.

**Main Outcomes and Measures:**

Outcomes were frequency of duration of asthma monitoring visits as well as actual and perceived optimal use and importance of monitoring tools and domains.

**Results:**

A total of 1319 participants with expertise in childhood asthma from 88 countries completed the survey. Participants included 1228 health care professionals with a balanced distribution across different care settings (305 [22.7%] primary care, 401 [29.9%] secondary, and 522 [38.9%] tertiary care) and 91 researchers. Children with mild to moderate asthma attended regular monitoring visits at a median (IQR) of 5.0 (2.5-8.0) months, with visits lasting a median (IQR) of 25 (15-25) minutes, whereas severe asthma required more frequent visits (median [IQR], 2.5 [1.0-2.5] months; median [IQR] duration, 25 [25-35] minutes). Monitoring of symptoms and control, adherence, comorbidities, lung function, medication adverse effects, and allergy were considered to be very high or high priority by more than 75% of the respondents. Different patterns emerged when assessing differences between actual and perceived optimal use of monitoring tools. For some tools, current and optimal practices did not differ much (eg, spirometry), whereas in others, there was considerable space for improvement (eg, standardized control and adherence tests). The largest gap was observed for between-visit monitoring with electronic trackers, apps, and smart devices. Differences across country economies, care settings, and medical specialties were modest.

**Conclusions and Relevance:**

These survey results suggest that pediatric asthma monitoring is performed generally homogeneously worldwide, in most cases following evidence-based standards. Wider use of standardized instruments and the intensification of continuous between-visit monitoring, supported by electronic devices, is needed for further improvement of disease outcomes. The results of this survey, in conjunction with the available evidence base, can inform recommendations toward further optimization.

## Key Points

**Question:**

What are the actual and perceived optimal childhood asthma monitoring practices globally?

**Findings:**

This international survey study of 1319 health care professionals and clinical academics from 88 countries found that children with mild to moderate asthma attended regular monitoring visits, whereas those with severe asthma required more frequent visits. Health care professionals reported monitoring pediatric asthma holistically, prioritizing symptoms, treatment adherence, pulmonary function, safety, and quality of life; airway inflammation, airway responsiveness, and biomarkers were evaluated on clinical indication.

**Meaning:**

These findings suggest that pediatric asthma monitoring is performed generally homogeneously worldwide, in most cases following evidence-based standards.

## Teaser

This survey study of health care professionals and clinical academics examines how asthma monitoring practices diverge internationally and to what extent these practices fall short of an ideal monitoring care pathway.

## Introduction

Asthma remains a major cause of morbidity, with socioeconomically challenged populations experiencing the most cases.^1,2^ Asthma outcomes largely depend on the quality and intensity of regular monitoring.^3,4^ Periodic or continuous assessment and review are key elements of asthma monitoring, according to the Global Initiative for Asthma.^5^ Nevertheless, monitoring procedures have attracted less attention than approaches to diagnosis or treatment choices, and monitoring pathways have been extensively revisited during the COVID-19 pandemic.^6,7^ These considerations are vital when it comes to children, in whom disease variability is higher in parallel to the child’s growth.^8^

The literature offers substantial evidence on the efficacy of monitoring of particular domains, such as symptoms,^9^ lung function,^10^ airway inflammation,^11^ airway responsiveness,^12^ and adherence to treatment,^13^ as well as on specific tools used within each domain. Additional aspects, such as the assessment of medication adverse effects, inhaler technique, psychological status, exercise, environmental factors, or diet, are also considered important. However, there is little formal assessment of prioritization among domains or on the value of care pathways that may result from different monitoring strategies. Innovative strategies, particularly on remote patient monitoring, are rapidly appearing.^14^

An important step toward optimal monitoring is understanding current practices. Although the literature on monitoring has been reviewed,^15^ the characteristics of pediatric asthma monitoring in the clinical setting have never been assessed. Paediatric Asthma in Real Life (PeARL) is a think tank set up by the Respiratory Effectiveness Group that consists of international health care professionals (HCPs), clinical academics, and expert patient representatives with expertise in and professional exposure to pediatric asthma.^16,17^ Asthma monitoring techniques and frequency were prioritized in a previous PeARL report that explored unaddressed clinical needs in pediatric asthma.^16^

In this study, we surveyed monitoring practices worldwide, including in various health care settings. The aim of the study was to examine how monitoring practices diverge internationally and to what extent these practices fall short of an ideal monitoring care pathway, as perceived by HCPs. In addition, we extended our survey to crowdsource envisioned optimal approaches, providing the opportunity to compare and portray actual status and unmet needs.

## Methods

This quantitative survey was developed, conducted, and reported following guidance on survey research by the American Association of Public Opinion Research.^18^ A list was prepared on the potential domains of monitoring and the tools that can be used to assess each domain (eTable 1 in Supplement 1). This list was based on literature searches (eAppendix in Supplement 1) followed by a saturation exercise among the PeARL Think Tank members, including expert patient representatives. After deduplication, the list was used by the steering group to develop a survey designed to capture the frequency and priority of each of the monitoring domains, the frequency with which each of the tools is used in clinical practice, as well as the proportion of patients who require monitoring with each technique. The questions were repeated for optimal monitoring according to the perception of the respondent. Finally, information was captured on demographic and professional details of the respondents. Independent members of the group reviewed the survey to establish completeness and face validity. Ethics review was not required for this survey of HCPs in line with the UK National Health Service Research Ethics Committee guidance. Participant consent was implied by voluntary completion of the survey. The study followed the American Association for Public Opinion Research (AAPOR) reporting guideline.

The survey (eAppendix in Supplement 1) was launched via SurveyMonkey, version September 2021 (Momentive) between April 12 and September 3, 2021. It was disseminated globally to HCPs and clinical academics with a professional interest in and exposure to childhood asthma by email and through social media (Twitter and LinkedIn) by the Respiratory Effectiveness Group and European Academy of Allergy and Clinical Immunology. Responses were considered valid provided the participants answered at least 1 clinical question.

### Statistical Analysis

Responses are presented descriptively. We tested for differences between the frequency that different techniques are actually used in practice vs optimal practice using cumulative link models for ordinal regression based on analysis of variance. The same test was used to explore between-group differences. We assessed differences across medical settings and country economies (World Bank classification). Because this was an extensive survey with optional responses, it was anticipated that some participants may discontinue before completing it. To explore potential attrition bias, we compared baseline characteristics and responses of those who completed at least 25%, 50%, 75%, and 100% of the responses compared with those who did not reach each of these thresholds, using 1-way, ordinal analysis of variance. In addition, we explored potential differences in the responses of European vs non-European respondents.

Analysis was performed in R software, version 3.6.3 (R Foundation for Statistical Computing). All statistical tests were 2-sided, and the critical level of significance was set at *P* < .05.

## Results

The survey was completed by 1319 participants from 88 countries, including 1228 HCPs with a balanced distribution across different care settings (305 [22.7%] primary care, 401 [29.9%] secondary, and 522 [38.9%] tertiary care) and 91 researchers (Table; eFigures 1-5 in Supplement 1). Among HCPs, 871 (66.0%) were asthma specialists, whereas the remaining were generalists with an interest in asthma. Participants from low- or lower- to middle-income countries represented 10.2% of this sample. The attrition rate varied from 6.5% to 28.9% across the questions. Of 150 questions, the median completion rate per participant was 145 questions (IQR, 56-147 questions).

**Table.** Demographic Characteristics of the Survey Respondents^a^

Characteristic	Health care professionals, No. (%)			Researchers, No. (%) (n = 91)
	Primary care (n = 305)	Secondary care (n = 401)	Tertiary care (n = 522)	
Income				
Low to lower middle	9 (3.0)	25 (6.2)	72 (13.8)	28 (30.8)
Upper middle	58 (1.9)	85 (21.2)	99 (19.0)	9 (9.9)
High	232 (76.1)	291 (72.6)	347 (66.5)	51 (56.0)
Continent				
Africa	7 (2.3)	5 (1.2)	38 (7.3)	6 (6.6)
Americas	35 (11.5)	58 (14.5)	81 (15.5)	16 (17.6)
Asia	30 (9.8)	47 (11.7)	98 (18.8)	12 (13.2)
Europe	217 (71.1)	272 (67.8)	296 (56.7)	51 (56.0)
Oceania	16 (5.2)	19 (4.7)	9 (1.7)	6 (6.6)
Region				
East Asia and Pacific	21 (6.9)	38 (9.5)	52 (10.0)	9 (10.0)
Europe and Central Asia	231 (75.7)	285 (71.1)	311 (59.6)	53 (58.2)
Latin America and Caribbean	22 (7.2)	28 (7.0)	55 (10.5)	3 (3.3)
Middle East and North Africa	2 (0.7)	4 (1.0)	24 (4.6)	1 (1.1)
North America	13 (4.3)	30 (7.5)	26 (5.0)	13 (14.3)
South Asia	4 (1.3)	12 (3.0)	27 (5.2)	3 (3.3)
Sub-Saharan Africa	6 (2.0)	4 (1.0)	23 (4.4)	6 (6.6)
Sector				
Public	80 (26.2)	208 (51.9)	287 (55.0)	NA
Private	182 (59.7)	137 (34.2)	105 (20.1)	NA
Both	43 (14.1)	56 (14.0)	130 (24.9)	NA
Specialists vs generalists				
Asthma specialists	129 (42.3)	287 (71.6)	455 (87.2)	NA
Generalists	176 (57.7)	114 (28.4)	67 (12.8)	NA
Specialization				
General practice	26 (8.5)	1 (0.2)	3 (0.6)	NA
Allergy (adults and children)	56 (18.4)	105 (26.2)	109 (20.9)	NA
Pediatric allergy, immunology, or pulmonology	73 (23.9)	182 (45.4)	346 (66.3)	NA
Pediatrics	137 (44.9)	107 (26.7)	56 (10.7)	NA
Nurse specialist	5 (1.6)	5 (1.2)	4 (0.8)	NA
Other	8 (2.6)	1 (0.2)	4 (0.8)	NA

Abbreviation: NA, not applicable.

^a^ Demographic characteristics are summarized as a percentage of all participants; missing data are not listed in this table.

### Frequency, Duration, and Priorities

**Figure 1.**
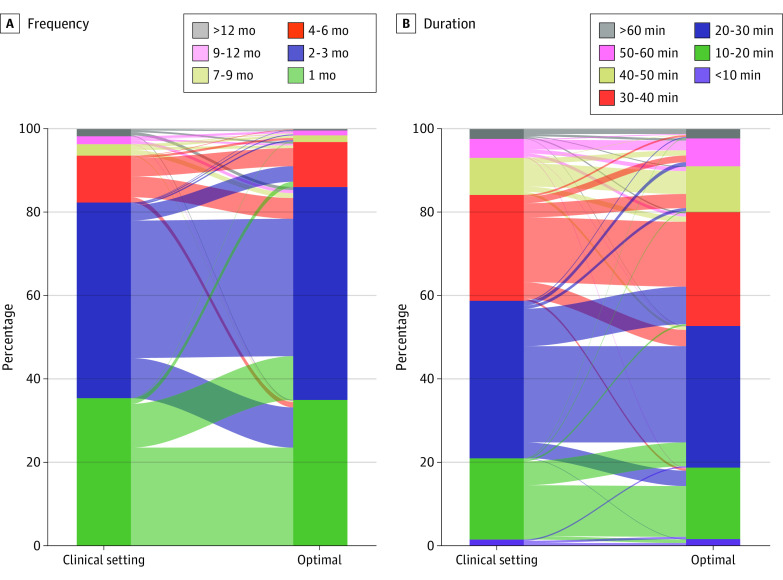
Actual and Perceived Optimal Monitoring Visit Frequency and Duration in Severe Asthma Differentiation of responses between actual and optimal conditions is shown as lines connecting the bars.

Most physicians follow a typical monitoring schedule (Figure 1; eFigure 6 in Supplement 1), which includes regular visits every 2 to 6 months (median [IQR], 5.0 [2.5-8.0] months) for mild to moderate asthma and 1 to 3 months (median [IQR], 2.5 [1.0-2.5] months) for severe asthma, with each visit lasting 10 to 40 minutes (median [IQR], 25 [15-25] minutes for mild-moderate asthma and 25 [25-35] minutes for severe asthma) (Figure 1C and 1D). These parameters were very close to the perceived optimal (mild to moderate asthma: median [IQR] frequency, 5.0 [2.5-5.0] months; median [IQR] duration, 25 [15-25] minutes; severe asthma: median [IQR] frequency, 2.5 [1.0-2.5] months; median [IQR] duration, 25 [25-35] minutes). Of 1283 participants, 833 (64.9%) did not suggest any change, whereas 431 (33.6%) suggested small changes in the duration of monitoring visits (1 step of the scale). Similarly, 753 (58.7%) did not suggest any change, and 425 (33.1%) suggested small changes in visits frequency.

**Figure 2.**
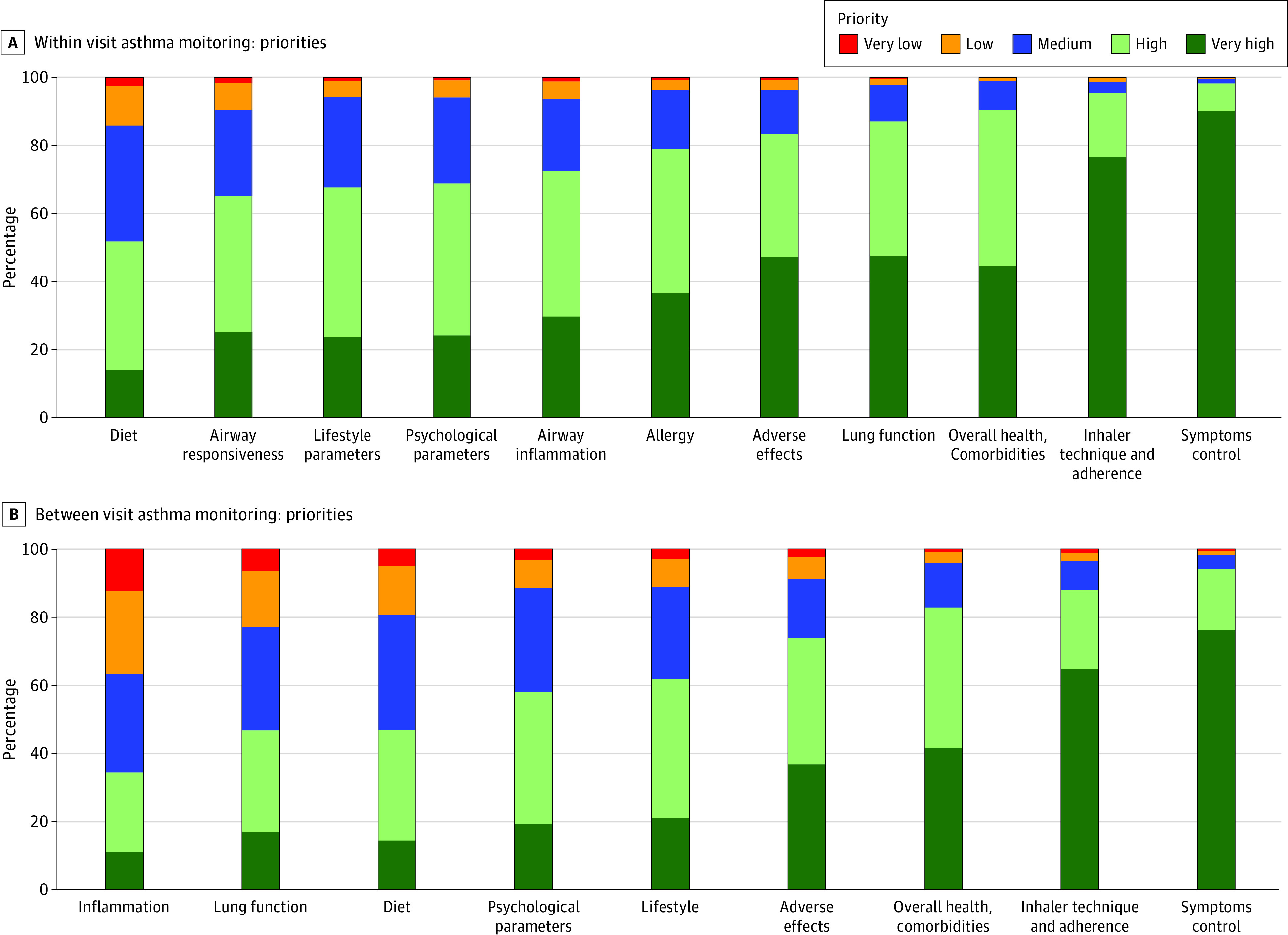
Prioritization of Asthma Monitoring Domains

The prioritization of monitoring domains is shown in Figure 2A. Control, adherence, and comorbidities were the highest priorities, with more than 90% of respondents considering them of high or very high priority. Nevertheless, all domains were considered of high priority in more than 50% of respondents. Respondents reported altering monitoring frequency mainly in response to exacerbations (1211 of 1248 [97.0%]) and to a lesser extent age (470 of 1248 [37.7%]), patient preference (422 of 1248 [33.8%]), or environmental changes (eg, moving residence) (367 of 1248 [29.4%]).

### Tools and Techniques Used During Monitoring Visits

**Figure 3.**
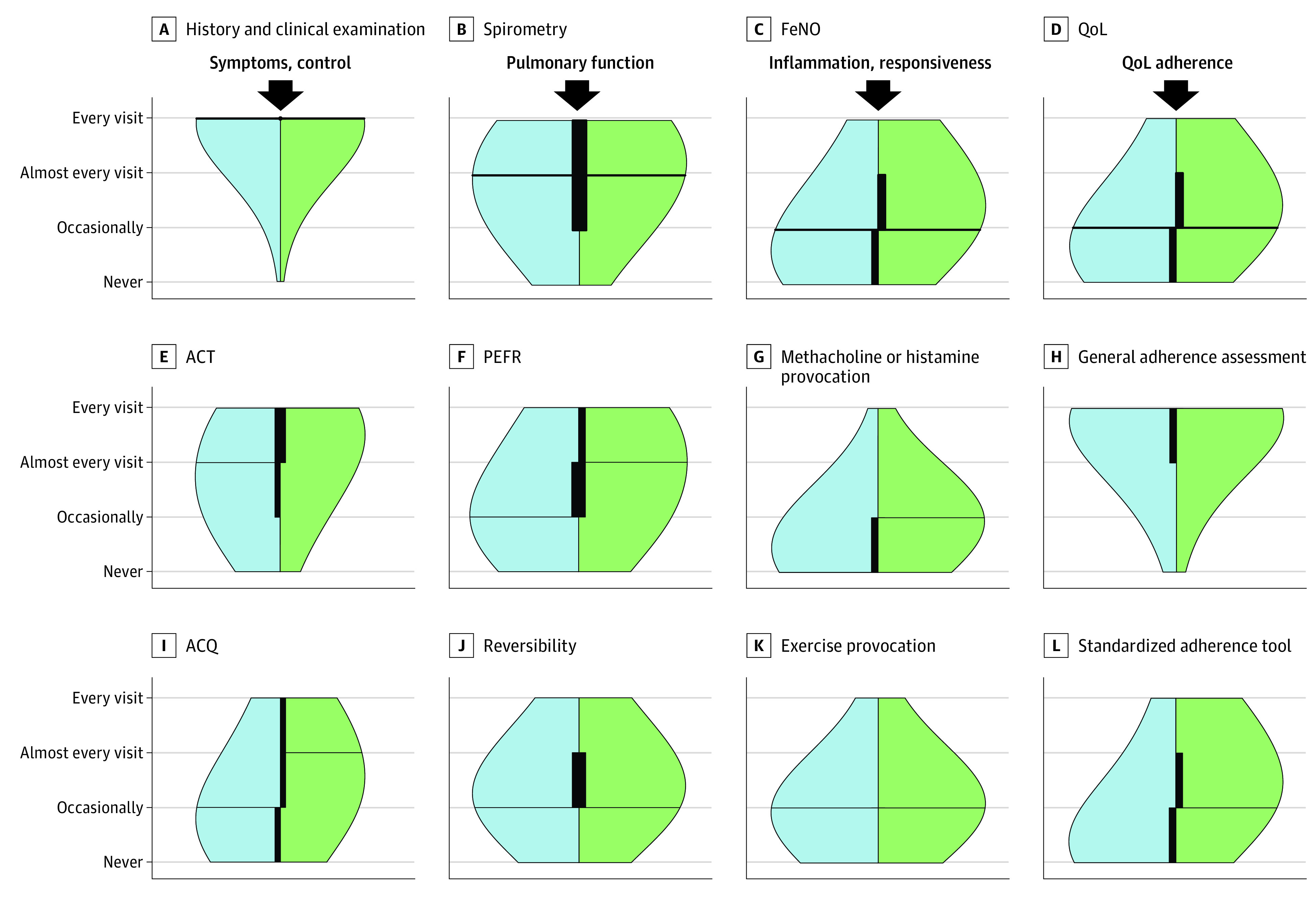
Use of Monitoring Tools During Asthma Monitoring Visits Actual (blue) and perceived (green) optimal use of monitoring tools during asthma monitoring visits. All outcomes were significant at *P* < .001 except for history and clinical examination (panel A). Lines represent medians; black bars, IQRs. The thickness of the IQR bars represents the density of data within the range. ACQ indicates asthma control questionnaire; ACT, asthma control test; FeNO, fractional exhaled nitric oxide; PEFR, peak expiratory flow rate; and QoL, quality of life.

The tools and techniques used during monitoring visits are presented in Figure 3 as well as eFigure 7 and eTable 2 in Supplement 1. History and clinical examination were performed almost ubiquitously (1095 of 1198 [97.9%]). Among the control tests, the Asthma Control Test was most often used, with 995 of 1104 HCPs (90.1%) using it at least occasionally and 334 of 1104 (30.3%) at every visit. The Asthma Control Questionnaire was used by 718 of 1069 HCPs (67.2%) and the Composite Asthma Severity Index by 478 of 1070 (44.7%). In the optimal scenario, respondents reported a need for a significant increase in the use of all control instruments.

Lung function was evaluated mostly with spirometry (1031 of 1107 [93.1%]) or with peak expiratory flow rate (PEFR) (840 of 1072 [78.4%]), whereas oscillometry (389 of 1089 [35.7%]) or plethysmography (406 of 1093 [37.1%]) is performed in only a few cases. Reversibility testing was used by 1036 of 1105 (93.8%), but mostly occasionally. Different patterns were observed when comparing actual with optimal conditions; there were no differences between actual and optimal for spirometry and reversibility testing, whereas a large move from no use to occasional use was observed for oscillometry and plethysmography.

Evaluation of airway responsiveness and reactivity was performed occasionally. Exercise challenges prevailed (900 of 1107 [81.3%]), followed by methacholine or histamine (503 of 1108 [45.4%]), cold air (396 of 1094 [36.2%]), mannitol or adenosine (173 of 1092 [15.8%]), and eucapnic voluntary hyperventilation (153 of 1097 [13.9%]). In all cases, 65% to 80% of respondents considered that, ideally, such tests should be available for occasional use.

Evaluation of airway inflammation was performed with fractional exhaled nitric oxide (FeNO) by 637 of 1113 respondents (57.2%), mostly occasionally. However, 450 of 973 (46.2%) suggested that FeNO should ideally be used in every or almost every visit. Exhaled breath condensate was used by 134 of 1104 respondents (12.1%) and volatile organic compounds by 77 of 1102 respondents (7.0%), but there was a wider interest for occasional use as part of optimal monitoring.

Total and specific serum IgE and peripheral blood eosinophils are used occasionally by more than 90% of respondents, who believe that this requires no change. This is the same with skin prick tests, for which a small increase in use is envisioned, whereas multiplex allergen tests are used by 706 of 1103 respondents (64.0%) but desired by 807 of 962 (83.9%).

Among potential treatment adverse events, growth was monitored by 1095 of 1107 respondents (98.9%). Adrenal function was monitored by 786 of 1103 (71.3%), ophthalmological evaluation was performed by 740 of 1107 (66.8%), and bone mineral density was measured by 635 of 1108 (57.3%). More than 90% considered these parameters important in an ideal setting.

Adherence and inhaler technique were monitored by 1093 of 1110 respondents (89.5%). Education was provided at almost every visit by 935 of 1110 respondents (84.2%). However, only 492 of 1106 respondents (44.5%) ever uses a standardized adherence tool, and 584 of 1106 (52.8%) referred to electronic patient and/or pharmacy records to assess adherence; both were suggested in an ideal setting by more than 90%.

Quality of life was assessed by 685 of 1110 HCPs (61.7%). In the perceived optimal setting, this increased to more than 90%. Psychological, nutritional, and lifestyle aspects were variably monitored; in all instances, these parameters were perceived as improvable.

### Between-Visit Monitoring

A more dynamic picture was drawn for monitoring between regular visits. The top priorities were similar as for regular visits: symptoms and control, adherence, comorbidities, and adverse events (90%-70%) (Figure 2B). However, lung function was considered a high or very high priority by only 449 of 960 respondents (46.8%).

**Figure 4.**
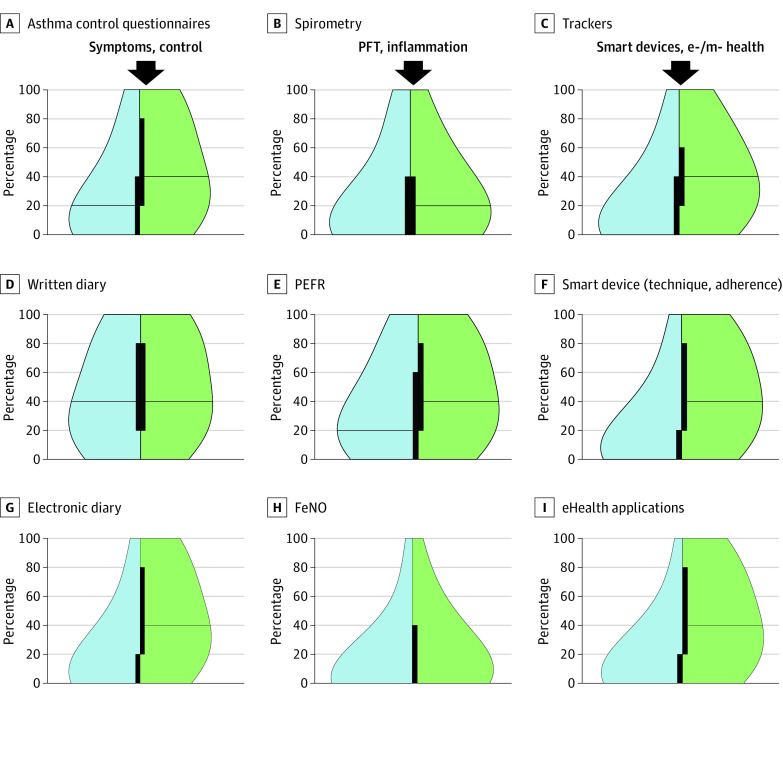
Use of Monitoring Tools Between Asthma Monitoring Visits Actual (blue) and perceived (green) optimal use of monitoring tools between asthma monitoring visits. The proportions of patients in which the tool is or should be offered are shown. All outcomes were significant at *P* < .001. mHealth trackers refer to the use of wearables, mobile phones, tablets, and smartphone applications for collecting health-related information. PEFR indicates peak expiratory flow rate; FeNO, fractional exhaled nitric oxide; PFT, pulmonary function test.

Most between-visit monitoring options were offered to only a few patients (Figure 4). Written diaries were used in a median (IQR) of 40% (20%-80%) of patients, and electronic diaries were used rarely (median [IQR], 0% [0%-20%] of patients). Peak expiratory flow rate was used in a median (IQR) of 20% (0%-60%) of patients and spirometry in 0% (0%-40%) of patients. However, in almost all cases (except written diaries and FeNO), the aspired use was considerably higher, with new technologies (eHealth, mHealth, and smart devices) showing the largest gaps.

### Differentiation of Responses by Specialty, Level of Care, Geographic Region, and Affluence

Overall, we observed limited differences in the responses from different care settings or countries of different affluence per the World Health Organization (eFigures 12-17 in Supplement 1). The duration of visits for mild to moderate asthma was shorter in lower-income countries (median of 10-20 vs 20-30 minutes), whereas for severe asthma, visits were longer in upper- to middle-income economies (median of 30-40 vs 20-30 minutes). Moreover, the duration of severe asthma monitoring visits of those working in the tertiary care setting or more affluent countries was more dispersed, suggesting variability in practice. Prioritization was similar across respondents of different economies and care settings (eFigures 8-11 in Supplement 1).

Modest differences were observed when comparing professionals of different groups on use of monitoring instruments (eFigures 14-17 in Supplement 1). Although in low- and middle-income countries history and clinical examination were consistently used in every monitoring visit, in high-income countries, the use of standardized control questionnaires appeared to be prioritized for assessing symptoms and control. Spirometry was used more frequently in secondary and tertiary care and in more affluent countries, whereas an opposite trend was observed for peak expiratory flow rate. Our data suggest more widespread use of between-visit monitoring tools, predominantly symptom diaries but also spirometry and mHealth trackers in the less affluent countries. In addition, specialized care settings more frequently used body plethysmography, oscillometry, reversibility to bronchodilator, bronchial challenges, skin prick tests, and FeNO, whereas oxygen saturation was more often used in primary care. Finally, treatment adherence was more consistently assessed in secondary and tertiary care.

### Sensitivity Analyses

Sensitivity analyses did not reveal significant risk of attrition bias. A total of 1149 participants (87.0%) completed 25% of the questions, 981 (74.4%) completed 50%, 933 (70.7%) completed 75%, and 102 (7.7%) completed 100%. We did not observe significant differences between European vs non-European respondents.

## Discussion

To our knowledge, this survey study is the first description of actual pediatric asthma monitoring practices internationally. Combined with a prioritization exercise and a needs assessment, we point out priority areas of potential improvement. Different patterns of use of monitoring domains and tools emerged: (1) frequent use with expectations for further improvement (eg, control and adherence), (2) occasional use considered adequate (eg, spirometry and biomarkers), or (3) very low use with aspiration for availability (eg, technologically advanced tools available for research, oscillometry, and bronchoprovocations). Among several high-priority items, assessment of control, adherence, and growth were in the top tier, possibly reflecting empathetic monitoring directed at the needs of the patient (eBox in Supplement 1). In addition to clinical utility and efficacy, priorities may also reflect considerations such as time availability and access to technologies. Head-to-head studies comparing monitoring domains (eg, adherence vs adverse events) are not available or make little sense; therefore, consensus prioritization is important.

A surprising, but optimistic, finding was that differences not only in actual monitoring practices but also in aspirations across geography, economic status, or level of care were modest. Although actual practices may be influenced by international guidelines, it appears that, at least within the survey respondents, the core needs for pediatric asthma monitoring are universal. The area with the highest unmet need is that of new digital technologies for between-visit continuous monitoring, such as electronic patient diaries, smart devices for adherence monitoring, and health trackers.

Validated symptom and control scores are considered the cornerstone of asthma monitoring.^5^ They can identify and quantify poor asthma control, predict outcomes (such as exacerbations), and effectively guide further management.^19,20^ Not surprisingly, HCPs consistently use at least 1 score for monitoring asthma. Although the Asthma Control Test was more broadly used compared with the Asthma Control Questionnaire or the Composite Asthma Severity Index, our survey revealed a clear interest in expanding their use.

Spirometry is also used frequently, despite known limitations in childhood.^21^ The global lung initiative spirometry reference values and *z* scores for children and adolescents represent a significant step forward and need to be broadly adopted. Although PEFR is inferior to spirometry and its use has been questioned, it is a very simple and affordable measure of lung function. Because PEFR is an aerosol-generating procedure, its use was discouraged during the COVID-19 pandemic and its uptake may have been further reduced. In parallel, the use of novel tools, such as oscillometry, is limited, most likely because of limited availability and experience.^22^

Airway inflammation monitoring, through FeNO, may guide asthma treatment.^23^ Some added value has been shown in predicting response to inhaled corticosteroids and preventing exacerbations, but there was no effect on symptoms or use of medications.^24^ Availability, cost, and variability due to unrelated exposures are probably reflected in the current infrequent use of FeNO monitoring, with moderate interest for intensification.^25^

Airway hyperresponsiveness monitoring has been suggested as a strategy to improve outcomes in adult asthma,^26^ but this has not been confirmed in children.^12^ Bronchial challenges are relatively invasive and time-consuming and thus not currently recommended for monitoring.^5^ Unsurprisingly, their actual use for this purpose is rare, although they are only moderately desired. Exercise challenge stands out because it is occasionally performed in children, however with no interest for additional use in an optimal scenario.

Levels of total and specific IgE and numbers of sputum or circulating eosinophils are critical for phenotyping and often for choosing treatment, particularly in severe asthma.^27^ The presence of atopy also has important predictive value for asthma persistence.^28^ Sputum eosinophils may guide management; however, the process is difficult, especially in younger children; time-consuming; and not significantly better in the long term compared with symptom-based management.^29^ In addition, the extent and clinical significance of biomarker fluctuations are not well established, and phenotypes are not stable in adults and even less in children,^30,31^ which may explain their infrequent measurement and limited interest for expansion. Interestingly, the proposed optimal frequency of use of multiple allergen tests was less than the actual use (the only instance within the survey that such a reduction was suggested).

Besides growth monitoring, which was assessed by most respondents, other safety parameters were not considered a priority. Although extensive community studies do not support concerns about the potential long-term effect of inhaled corticosteroids on children’s development,^32^ inhaled corticosteroids, especially when used at excessive doses, as well as systemic corticosteroids and other asthma treatments, may have burdensome adverse events that require close monitoring.^33,34^ The structure of our survey, which only inquired about specific long-term safety parameters, possibly did not fully capture monitoring for adverse drug reactions.

Poor treatment adherence and inhalation technique are leading causes of treatment failure.^35,36^ Our findings suggest that HCPs are aware of this, assessing adherence and providing education regularly. However, most often, adherence is not assessed in a standardized way. Adherence evaluation could be optimized using clinically validated tools, including questionnaires such as the Medication Adherence Rating Scale or Test of Adherence to Inhalers, electronic monitoring adherence tools, and electronic patient records with prescription pick-up linkage^37,38^; however, this form of cooperation with patients may raise understandable ethical questions.

Although the importance of obesity,^39^ psychological status,^40^ and lifestyle^41^ has been highlighted, these factors are challenging to incorporate into regular monitoring because they require specialist input. In this regard, multidisciplinary teams may offer improved asthma management, although limited to tertiary care centers.^42^ Importantly, quality of life is not being adequately addressed, despite being prioritized as an outcome by patients, their families, and clinicians.^43^ Environmental exposures are highly relevant to asthma activity in children,^44,45^ but they also appear to be somehow neglected, possibly because of the challenge of identifying the environmental triggers and their fluctuations and the difficulty of modifying them.

In addition to visit-based intermittent monitoring, new technologies promise intensification and improvement of monitoring through regular or even continuous at-home evaluation.^37^ Although telemonitoring has not yet shown conclusive superiority,^46^ its potential and use have rapidly increased during the COVID-19 pandemic,^6^ whereas numerous approaches are being explored. Although the usefulness and reliability of traditional tools for between-visit monitoring, such as diary cards and peak expiratory flow measurements, have been challenged,^47^ there is much promise in mHealth and eHealth disease activity tracking,^48^ as well as development of smart devices for medication adherence.^49^ According to our findings, this innovation scene has only reached a small proportion of patients; nevertheless, there is high expectation toward the inclusion of such tools in an ideal setting. Interestingly, the pattern of use and expectation is almost identical for all the novel approaches, probably because progress (and consequent offer) is faster than validation. Nevertheless, highly promising and validated solutions, such as the MASK-air app, are rapidly being developed.^50^

### Strengths and Limitations

The study has several strengths. The sample size is large, with multistakeholders and international representation. The design allows direct comparisons between the actual and perceived ideal situation. Patient input was used in its design. A high proportion of the respondents were practicing physicians with direct clinical experience. The overrepresentation of tertiary care HCPs was addressed by subgroup analysis by treatment setting.

The study also has some limitations. As with any survey, this report is potentially biased by its sampling. Our study participants can be presumed to have had particular interest in pediatric asthma and therefore high clinical standards and expectations independent of their specialty. Nevertheless, these individuals would be the exact audience expected to study and appraise any further international harmonization efforts, as well as being the hubs for educating HCPs on a larger scale.

Although we aimed to cover all geographic areas and socioeconomic strata to the maximum possible extent, Europe is overrepresented, whereas low-income countries and Africa are less well represented in our sample. This limitation may reflect a lower asthma incidence but most probably represents reduced information access to and from these areas. In contrast to other studies^51,52^ that specifically focused on underrepresented parts of the globe, we did not specifically aim to oversample underrepresented parts of the world, which might have remedied this limitation. Because this was an extensive survey, a moderately high attrition rate was expected (range, 6.5%-29%); however, sensitivity analysis confirmed that this did not affect the outcomes.

Environmental factors were not adequately addressed in our survey and need to be addressed in future research. We only assessed whether environmental factors trigger a change in the frequency of asthma monitoring visits and not how often they are addressed in current or optimal practice.

Practice surveys are inherently limited by the subjectivity of responses and tendency of HCPs to overestimate their good care. Ideally, these findings will need to be validated in actual clinical data. Furthermore, we recognize that monitoring choices should be personalized at a level of complexity beyond the capacity of a survey.

## Conclusions

The results of this survey study suggest that asthma monitoring in children is performed rather homogeneously worldwide, with priorities consistent in most cases with the evidence. A core group of tools, including history, clinical examination, and treatment adherence assessment, are consistently implemented. Areas for improvement include standardized asthma control questionnaires, pulmonary function monitoring, delivery of patient education, and expansion of between-visit monitoring. Finally, a desire for access to advanced asthma phenotyping tools, such as airway inflammation and responsiveness assessment, emerged, especially among HCPs working in secondary or tertiary care and in more affluent health systems. There is an apparent need for additional standardization and increased availability of tools, although the promise of new technologies has already been translated into high expectations in continuous monitoring. The results of this survey, in conjunction with the available evidence base, can inform recommendations toward further optimization.

## Notes

Supplement 1. **eTable 1.** Monitoring Domains and Tools Assessed **eTable 2.** Heatmap Summarising the Proportion of Respondents That Would Prefer to Use Each Monitoring Tool More Frequently **eAppendix.** Supplementary Methods **eFigure 1.** Participants by Country Income **eFigure 2.** Participants by Geographic Region **eFigure 3.** Participants by Country of Residence (Global Figures) **eFigure 4.** Participants by Country of Residence (Europe) **eFigure 5.** Participants by Country of Residence **eFigure 6.** Actual and Perceived Optimal Monitoring Visit Frequency (in Months) and Duration (in Minutes) for Children With Mild or Moderate Asthma **eFigure 7.** Actual (Light Blue, Left Side of the Violin Plots) and Perceived Optimal (Green, Right Side of the Violin Plots) Use of Monitoring Tools During Asthma Monitoring Visits **eFigure 8.** Prioritisation of Different Domains of Asthma Monitoring During Follow-up Visits, Stratified by Country Income **eFigure 9.** Prioritisation of Different Domains of Asthma Monitoring During Follow-up Visits, Stratified by Care Setting **eFigure 10.** Prioritisation of Different Domains of Asthma Monitoring Between Follow-up Visits, Stratified by Country Income **eFigure 11.** Prioritisation of Different Domains of Asthma Monitoring Between Follow-up Visits, Stratified by Care Setting **eFigure 12.** Actual (Left) and Perceived Optimal (Right) Monitoring Visit Frequency (in Months), for Children With Mild/Moderate and With Severe Asthma, Stratified by the Country Income and Health Care Setting of the Respondents **eFigure 13.** Actual (Left) and Perceived Optimal (Right) Monitoring Visit Duration (in Minutes), for Children With Mild/Moderate and With Severe Asthma, Stratified by the Country Income and Health Care Setting of the Respondents **eFigure 14.** Actual (Light Blue, Left Side of the Violin Plots) and Perceived Optimal (Green, Right Side of the Violin Plots) Use of Monitoring Tools During Asthma Monitoring Visits, Stratified by Country Income **eFigure 15.** Actual (Light Blue, Left Side of the Violin Plots) and Perceived Optimal (Green, Right Side of the Violin Plots) Use of Monitoring Tools During Asthma Monitoring Visits, Stratified by Care Setting **eFigure 16.** Actual (Light Blue, Left Side of the Violin Plots) and Perceived Optimal (Green, Right Side of the Violin Plots) Use of Monitoring Tools Between Asthma Visits, Stratified by Country Income **eFigure 17.** Actual (Light Blue, Left Side of the Violin Plots) and Perceived Optimal (Green, Right Side of the Violin Plots) Use of Monitoring Tools Between Asthma Visits, Stratified by Care Setting **eBox.** Identified Priorities for Monitoring During Visits

Supplement 2.
Nonauthor Collaborators

Supplement 3.
Data Sharing Statement
