# Supplementary material for: Current and Optimal Practices in Childhood Asthma Monitoring Among Multiple International Stakeholders
Source: JAMA Netw Open. 2023 May 12;6(5):e2313120. doi: 10.1001/jamanetworkopen.2023.13120 (PMC10182430; doi:10.1001/jamanetworkopen.2023.13120)
Supplement: Supplement 1. — eTable 1. Monitoring Domains and Tools Assessed eTable 2. Heatmap Summarising the Proportion of Respondents That Would Prefer to Use Each Monitoring Tool More Frequently eAppendix. Supplementary Methods eFigure 1. Participants by Country Income eFigure 2. Participants by Geographic Region eFigure 3. Participants by Country of Residence (Global Figures) eFigure 4. Participants by Country of Residence (Europe) eFigure 5. Participants by Country of Residence eFigure 6. Actual and Perceived Optimal Monitoring Visit Frequency (in Months) and Duration (in Minutes) for Children With Mild or Moderate Asthma eFigure 7. Actual (Light Blue, Left Side of the Violin Plots) and Perceived Optimal (Green, Right Side of the Violin Plots) Use of Monitoring Tools During Asthma Monitoring Visits eFigure 8. Prioritisation of Different Domains of Asthma Monitoring During Follow-up Visits, Stratified by Country Income eFigure 9. Prioritisation of Different Domains of Asthma Monitoring During Follow-up Visits, Stratified by Care Setting eFigure 10. Prioritisation of Different Domains of Asthma Monitoring Between Follow-up Visits, Stratified by Country Income eFigure 11. Prioritisation of Different Domains of Asthma Monitoring Between Follow-up Visits, Stratified by Care Setting eFigure 12. Actual (Left) and Perceived Optimal (Right) Monitoring Visit Frequency (in Months), for Children With Mild/Moderate and With Severe Asthma, Stratified by the Country Income and Health Care Setting of the Respondents eFigure 13. Actual (Left) and Perceived Optimal (Right) Monitoring Visit Duration (in Minutes), for Children With Mild/Moderate and With Severe Asthma, Stratified by the Country Income and Health Care Setting of the Respondents eFigure 14. Actual (Light Blue, Left Side of the Violin Plots) and Perceived Optimal (Green, Right Side of the Violin Plots) Use of Monitoring Tools During Asthma Monitoring Visits, Stratified by Country Income eFigure 15. Actual (Light Blue, Left Side of the Violin Pl [file jamanetwopen-e2313120-s001.pdf]

Supplementary Online Content

Papadopoulos NG, Mathioudakis AG, Custovic A, et al; PeARL Think Tank. Current and optimal practices in childhood asthma monitoring among multiple international stakeholders. *JAMA Netw Open*. 2023;6(5):e2313120. doi:10.1001/jamanetworkopen.2023.13120

eTable 1. Monitoring Domains and Tools Assessed
eTable 2. Heatmap Summarising the Proportion of Respondents That Would Prefer to Use Each Monitoring Tool More Frequently
eAppendix. Supplementary Methods
eFigure 1. Participants by Country Income
eFigure 2. Participants by Geographic Region
eFigure 3. Participants by Country of Residence (Global Figures)
eFigure 4. Participants by Country of Residence (Europe)
eFigure 5. Participants by Country of Residence
eFigure 6. Actual and Perceived Optimal Monitoring Visit Frequency (in Months) and Duration (in Minutes) for Children With Mild or Moderate Asthma
eFigure 7. Actual (Light Blue, Left Side of the Violin Plots) and Perceived Optimal (Green, Right Side of the Violin Plots) Use of Monitoring Tools During Asthma Monitoring Visits
eFigure 8. Prioritisation of Different Domains of Asthma Monitoring During Follow-up Visits, Stratified by Country Income
eFigure 9. Prioritisation of Different Domains of Asthma Monitoring During Follow-up Visits, Stratified by Care Setting
eFigure 10. Prioritisation of Different Domains of Asthma Monitoring Between Follow-up Visits, Stratified by Country Income
eFigure 11. Prioritisation of Different Domains of Asthma Monitoring Between Follow-up Visits, Stratified by Care Setting
eFigure 12. Actual (Left) and Perceived Optimal (Right) Monitoring Visit Frequency (in Months), for Children With Mild/Moderate and With Severe Asthma, Stratified by the Country Income and Health Care Setting of the Respondents
eFigure 13. Actual (Left) and Perceived Optimal (Right) Monitoring Visit Duration (in Minutes), for Children With Mild/Moderate and With Severe Asthma, Stratified by the Country Income and Health Care Setting of the Respondents
eFigure 14. Actual (Light Blue, Left Side of the Violin Plots) and Perceived Optimal (Green, Right Side of the Violin Plots) Use of Monitoring Tools During Asthma Monitoring Visits, Stratified by Country Income
eFigure 15. Actual (Light Blue, Left Side of the Violin Plots) and Perceived Optimal (Green, Right Side of the Violin Plots) Use of Monitoring Tools During Asthma Monitoring Visits, Stratified by Care Setting
eFigure 16. Actual (Light Blue, Left Side of the Violin Plots) and Perceived Optimal (Green, Right Side of the Violin Plots) Use of Monitoring Tools Between Asthma Visits, Stratified by Country Income
eFigure 17. Actual (Light Blue, Left Side of the Violin Plots) and Perceived Optimal (Green, Right Side of the Violin Plots) Use of Monitoring Tools Between Asthma Visits, Stratified by Care Setting
eBox. Identified Priorities for Monitoring During Visits

This supplementary material has been provided by the authors to give readers additional information about their work.

© 2023 Papadopoulos NG et al. *JAMA Network Open.*

eTable 1. Monitoring Domains and Tools Assessed

**Domain**	**Tools**
Symptoms and control	History and Clinical Examination<br>Asthma Control Test (ACT)<br>Asthma Control Questionnaire (ACQ)<br>Composite Asthma Severity Score (CASI)<br>Other Standardised Questionnaire<br>Overall Health and Comorbidities
Lung function	Spirometry<br>Peak Expiratory Flow Rate (PEFR)<br>Reversibility Testing<br>Oscillometry<br>Plethysmography<br>Oxygen Saturation
Inflammation	Fractional Exhaled Nitric Oxide (FeNO)<br>Exhaled Breath Condensate (EBC)<br>Volatile Organic Compounds (VOCs)
Bronchial hyperresponsiveness	Methacholine or Histamine Challenge<br>Mannitol or Adenosine Challenge<br>Exercise Challenge<br>Cold Air Challenge<br>Eucapnic Volatile Hyperventilation
Biomarkers	Total IgE<br>Specific IgE<br>Blood Counts (EOS)<br>Skin Prick Tests<br>Multiplex Tests
Adverse events	Growth<br>Adrenal Function<br>Ophthalmological Assessment<br>Bone Mineral Density (BMD)

© 2023 Papadopoulos NG et al. *JAMA Network Open.*

Quality of life	Quality of Life<br>Stress, Depression and Coping<br>Psychologist or Psychiatrist Assessment<br>Nutritionist Assessment<br>Diet Questionnaires<br>Lifestyle Questionnaires
Adherence and Education	General Adherence Assessment<br>Standardised Adherence Evaluation<br>Electronic Patient Records<br>Education of Patients and Family Members<br>Inhalation Technique

© 2023 Papadopoulos NG et al. *JAMA Network Open.*

eAppendix. Supplementary Methods

Literature searches

The development of this survey was informed by scoping literature searches. We searched PubMed for studies published during the preceding decade (2012-2021) using the following search strategy:

#1 Asthma [MH]

#2 Asthma [tiab]

#3 Monitoring [tiab]

#4 Adults[MH] NOT (Children [MH] or (Adolescents [MH]))

#5 (#1 OR #2) AND #3 NOT #4

All studies that were yielded by the searches (n=1576) were screened by one author at a title and abstract level, followed by full text evaluation if that was deemed appropriate. We looked for tools used to monitor childhood asthma within and between monitoring visits. The results of this scoping review informed a saturation exercise among the PeARL members that are listed as co-authors.

© 2023 Papadopoulos NG et al. *JAMA Network Open.*

Supplementary Methods: Survey

Below we present the PeARL childhood asthma monitoring survey

Introduction:

Asthma in children is a major health concern. Following diagnosis, regular monitoring is necessary to keep the disease under control.

With this survey, PeARL intends to assess how pediatric asthma is currently monitored worldwide and crowdsource the views of different stakeholders on optimal monitoring.

The survey makes the following assumptions:

- A formal diagnosis of asthma has been established
- Physical monitoring visits with a healthcare professional
- Self-monitoring in-between visits.

Demographics

Country of practice (Dropdown)

Are you a healthcare professional caring regularly for children with asthma

- Yes
- No

Health care professionals

Do you work in:

Setting – Sector: Private, Public, Both

Setting – Level of care: Primary, Secondary, Tertiary Care

Which of the following best describes your specialisation?

- General Practice

© 2023 Papadopoulos NG et al. *JAMA Network Open.*

- Pediatrics
- Pediatric pulmonology
- Pediatric allergy
- Pediatric allergy & pulmonology
- Pediatric immunology
- Allergy (adult & pediatric)
- Pulmonology (adult & pediatric)
- Specialist nurse
- Other

* 5. In your daily practice, how often do you review/monitor children with diagnosed asthma, assuming there is no exacerbation in between ? How long does each visit last?

	Frequency (months)	Duration (minutes)
Mild-moderate asthma		
Severe asthma		

6. For which of the following reasons would you consider changing visit frequency? (choose any/all that apply)

- ☐ Exacerbations
- ☐ Envriromental changes (e.g. moving residence)
- ☐ Age
- ☐ Parent preference
- ☐ None

>

- ☐ Other (please specify)

© 2023 Papadopoulos NG et al. *JAMA Network Open.*

In your daily practice, how often do you use the following tools during a monitoring visit ?

8. Assessment of **Symptoms & Control** in real life

	Every visit	Almost every visit	Occasionally/Sometimes	Rarely	Never
History and clinical examination (asthma specific)	○	○	○	○	○
Asthma Control Test (ACT)	○	○	○	○	○
Asthma Control Questionnaire (ACQ)	○	○	○	○	○
Composite Asthma Severity Index (CASI)	○	○	○	○	○
Control of Allergic Rhinitis and Asthma Test for Children (CARATKids)	○	○	○	○	○
Other standardised questionnaire (asthma specific)	○	○	○	○	○
Overall health including comorbidities	○	○	○	○	○

© 2023 Papadopoulos NG et al. *JAMA Network Open.*

9. Assessment of **Lung function** in real life

	Every visit	Almost every visit	Occasionally/Sometimes	Rarely	Never
Spirometry	◯	◯	◯	◯	◯
Peak flow rate	◯	◯	◯	◯	◯
Reversibility to beta2-agonist	◯	◯	◯	◯	◯
Impulse oscillometry (IOS)	◯	◯	◯	◯	◯
Plethysmography	◯	◯	◯	◯	◯
Lung clearance index	◯	◯	◯	◯	◯
Oxygen Saturation (SpO2)	◯	◯	◯	◯	◯

10. Assessment of **Airway Inflammation** in real life

	Every visit	Almost every visit	Occasionally/Sometimes	Rarely	Never
Fractional exhaled nitric oxide (FeNO)	◯	◯	◯	◯	◯
Exhaled breath condensate (EBC)	◯	◯	◯	◯	◯
Breath biopsy (Volatile organic compounds - VOCs)	◯	◯	◯	◯	◯

11. Assessment of **Airway hyperresponsiveness** in real life

	Every visit	Almost every visit	Occasionally/Sometimes	Rarely	Never
Direct (Methacholine, Histamine)	◯	◯	◯	◯	◯
Indirect (Mannitol, Adenosine)	◯	◯	◯	◯	◯
Exercise	◯	◯	◯	◯	◯
Cold air	◯	◯	◯	◯	◯
Eucapnic voluntary hyperventilation (EVH) test	◯	◯	◯	◯	◯

© 2023 Papadopoulos NG et al. *JAMA Network Open.*

12. Assessment of **Allergy** in real life

	Every visit	Almost every visit	Occasionally/Sometimes	Once	Never
Total IgE	○	○	○	○	○
Serum specific IgE	○	○	○	○	○
Blood counts (eosinophils)	○	○	○	○	○
Skin Prick Tests	○	○	○	○	○
Multiplex tests (e.g. ImmunoCap ISAC, Alex)	○	○	○	○	○

13. Assessment of **Medication Adverse Events** in real life

	Every visit	Almost every visit	Occasionally/Sometimes	Rarely	Never
Growth: Height, Weight, BMI	○	○	○	○	○
Adrenal function	○	○	○	○	○
Ophthalmological assessment	○	○	○	○	○
Bone density	○	○	○	○	○

14. Assessment of **Psychological, Diet, Lifestyle Parameters** in real life

	Every visit	Almost every visit	Occasionally/Sometimes	Rarely	Never
Quality of life questionnaires (e.g. PAQLQ)	○	○	○	○	○
Stress/depression/coping questionnaires	○	○	○	○	○
Psychologist / Psychiatrist referral	○	○	○	○	○
Nutritionist referral	○	○	○	○	○
Diet questionnaire (e.g. FFQ, 24 hour recall)	○	○	○	○	○
Lifestyle questionnaire	○	○	○	○	○

© 2023 Papadopoulos NG et al. *JAMA Network Open.*

15. Assessment of **Adherence** & provision of **Education** in real life

	Every visit	Almost every visit	Occasionally/Sometimes	Rarely	Never
General adherence history	◯	◯	◯	◯	◯
Standardised adherence questionnaire (e.g. TAI)	◯	◯	◯	◯	◯
Electronic health/pharmacy record check for adherence	◯	◯	◯	◯	◯
General asthma education	◯	◯	◯	◯	◯
Inhalation technique, testing/feedback	◯	◯	◯	◯	◯

16. In your practice, in what proportion of patients do you recommend the following tools for **between-visit home monitoring**?

	0%	20%	40%	60%	80%	100%
ACT/ACQ/CASI/CARATKids	◯	◯	◯	◯	◯	◯
Written diary	◯	◯	◯	◯	◯	◯
Electronic diary	◯	◯	◯	◯	◯	◯
Spirometry	◯	◯	◯	◯	◯	◯
Peak Flow	◯	◯	◯	◯	◯	◯
FeNO (exhaled nitric oxide)	◯	◯	◯	◯	◯	◯
Physical activity trackers (e.g. fitbit)	◯	◯	◯	◯	◯	◯
Smart device (technique, adherence)	◯	◯	◯	◯	◯	◯
eHealth Apps	◯	◯	◯	◯	◯	◯

© 2023 Papadopoulos NG et al. *JAMA Network Open.*

* 17. How often should a child with asthma monitored, optimally, assuming there is no exacerbation in between? How long should each visit last?

	Frequency (months)	Duration (minutes)
Mild-moderate asthma		
Severe asthma		

18. How high should be the priority of each of the monitoring parameters below?

	Very high	High	Medium	Low	Very low
Asthma symptoms and control	○	○	○	○	○
Other symptoms (overall health, comorbitidies)	○	○	○	○	○
Lung function	○	○	○	○	○
Airway inflammation	○	○	○	○	○
Airway resposiveness	○	○	○	○	○
Allergy	○	○	○	○	○
Medication adverse events	○	○	○	○	○
Psychological parameters	○	○	○	○	○
Diet	○	○	○	○	○
Lifestyle parameters	○	○	○	○	○
Medication adherence & technique	○	○	○	○	○

© 2023 Papadopoulos NG et al. *JAMA Network Open.*

19. How frequently each of the following monitoring parameters should be evaluated?

	Each visit	Every 2-3 visits - yearly or biannually, but not every time	Upon indication/medical judgement	Rarely or never
Asthma symptoms and signs, control	○	○	○	○
Other symptoms (overall health, comorbitidies)	○	○	○	○
Lung function	○	○	○	○
Airway inflammation	○	○	○	○
Airway resposiveness	○	○	○	○
Allergy	○	○	○	○
Medication adverse events	○	○	○	○
Psychological parameters	○	○	○	○
Diet	○	○	○	○
Lifestyle parameters	○	○	○	○
Medication adherence & technique	○	○	○	○

20. For optimal symptoms and control monitoring, how often should the following be evaluated?

	Every time	Almost every time	Occasionally/Sometimes	Rarely	Never
History and clinical examination (asthma specific)	○	○	○	○	○
Asthma Control Test (ACT)	○	○	○	○	○
Asthma Control Questionnaire (ACQ)	○	○	○	○	○
Composite Asthma Severity Index (CASI)	○	○	○	○	○
Control of Allergic Rhinitis and Asthma Test for Children (CARATKids)	○	○	○	○	○
Other standardised questionnaire (asthma specific)	○	○	○	○	○
Overall health including comorbidities	○	○	○	○	○

© 2023 Papadopoulos NG et al. *JAMA Network Open.*

21. For optimal lung function monitoring, how often should the following be evaluated?

	Every visit	Almost every visit	Occasionally/Sometimes	Rarely	Never
Spirometry					
Peak Flow Rate					
Reversibility to beta2-agonist					
Impulse oscillometry (IOS)					
Plethysmography					
Oxygen Saturation (SpO2)					

22. For optimal airway inflammation monitoring, how often should the following be evaluated?

	Every visit	Almost every visit	Occasionally/Sometimes	Rarely	Never
Fractional exhaled nitric oxide (FeNO)					
Exhaled breath condensate (EBC)					
Breath biopsy (Volatile organic compounds - VOCs)					

23. For optimal airway hyperresponsiveness monitoring, how often should the following challenges be performed?

	Every visit	Almost every visit	Occasionally/Sometimes	Rarely	Never
Direct (Methacholine, Histamine)					
Indirect (Mannitol, Adenosine)					
Exercise					
Cold air					
Eucapnic voluntary hyperventilation (EVH) test					

© 2023 Papadopoulos NG et al. *JAMA Network Open.*

24. For optimal allergy monitoring, how often should the following be evaluated?

	Every visit	Almost every visit	Occasionally/Sometimes	Once	Never
Total IgE	○	○	○	○	○
Serum specific IgE	○	○	○	○	○
Blood counts (eosinophils)	○	○	○	○	○
Skin Prick Tests	○	○	○	○	○
Multiplex tests (e.g. ImmunoCap ISAC, Alex)	○	○	○	○	○

25. For optimal medication adverse events monitoring, how often should the following be evaluated?

	Every visit	Almost every visit	Occasionally/Sometimes	Rarely	Never
Growth: Height, Weight, BMI	○	○	○	○	○
Adrenal function	○	○	○	○	○
Ophthalmological assessment	○	○	○	○	○
Bone density	○	○	○	○	○

26. For optimal psychological, diet, lifestyle parameters monitoring, how often should the following be evaluated?

	Every visit	Almost every visit	Occasionally/Sometimes	Rarely	Never
Quality of life questionnaires (e.g. PAQLQ)	○	○	○	○	○
Stress/depression/coping questionnaires	○	○	○	○	○
Psychologist / Psychiatrist referral	○	○	○	○	○
Nutritionist referral	○	○	○	○	○
Diet questionnaire (e.g. FFQ, 24 hour recall)	○	○	○	○	○
Lifestyle questionnaire	○	○	○	○	○

© 2023 Papadopoulos NG et al. *JAMA Network Open.*

27. For optimal adherence & provision of education, how often should the following be done?

	Every visit	Almost every visit	Occasionally/Sometimes	Rarely	Never
General adherence history	○	○	○	○	○
Standardised adherence questionnaire (e.g. TAI)	○	○	○	○	○
Electronic health/pharmacy record check for adherence	○	○	○	○	○
General asthma education	○	○	○	○	○
Inhalation technique, testing/ feedback	○	○	○	○	○

28. How high is the priority of each of the monitoring parameters below **at home, in-between visits (self monitoring) ?**

	Very high	High	Medium	Low	Very low
Asthma symptoms and control	○	○	○	○	○
Other symptoms (overall health, comorbitidies)	○	○	○	○	○
Lung function	○	○	○	○	○
Airway inflammation	○	○	○	○	○
Medication adverse events	○	○	○	○	○
Psychological parameters	○	○	○	○	○
Diet parameters	○	○	○	○	○
Lifestyle parameters	○	○	○	○	○
Medication adherence & technique	○	○	○	○	○

© 2023 Papadopoulos NG et al. *JAMA Network Open.*

29. How frequently each of the following parameters should be evaluated **at home, in-between visits (self monitoring)** ?

	Daily	Weekly	Monthly	Ad hoc (when necessary)	Never
Asthma symptoms and control	○	○	○	○	○
Other symptoms (overall health, comorbitidies)	○	○	○	○	○
Lung function	○	○	○	○	○
Airway inflammation	○	○	○	○	○
Medication adverse events	○	○	○	○	○
Psychological parameters	○	○	○	○	○
Diet	○	○	○	○	○
Lifestyle parameters	○	○	○	○	○
Medication adherence & technique	○	○	○	○	○

30. In what proportion of patients should the following tools be used **at home, in-between visits (self monitoring)?**

	0%	20%	40%	60%	80%	100%
ACT/ACQ/CASI/CARATKids	○	○	○	○	○	○
Written diary	○	○	○	○	○	○
Electronic diary	○	○	○	○	○	○
Spirometry	○	○	○	○	○	○
Peak Flow	○	○	○	○	○	○
FeNO (exhaled nitric oxide)	○	○	○	○	○	○
Physical activity trackers (e.g. fitbit)	○	○	○	○	○	○
Smart device (techniques, adherence)	○	○	○	○	○	○
eHealth Apps	○	○	○	○	○	○

© 2023 Papadopoulos NG et al. *JAMA Network Open.*

Characteristics of the participants

eFigure 1. Participants by Country Income

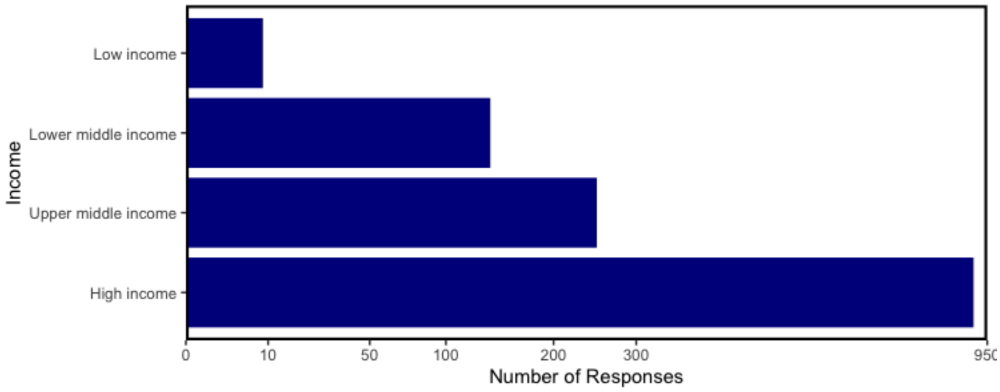

eFigure 2. Participants by Geographic Region

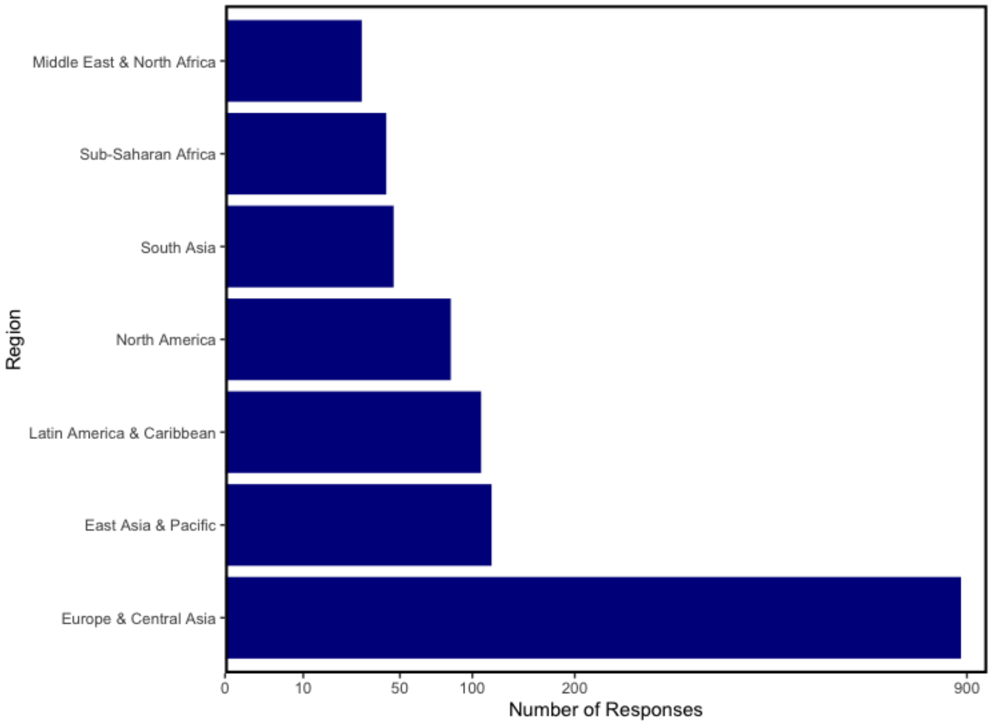

© 2023 Papadopoulos NG et al. *JAMA Network Open.*

eFigure 3. Participants by Country of Residence (Global Figures)

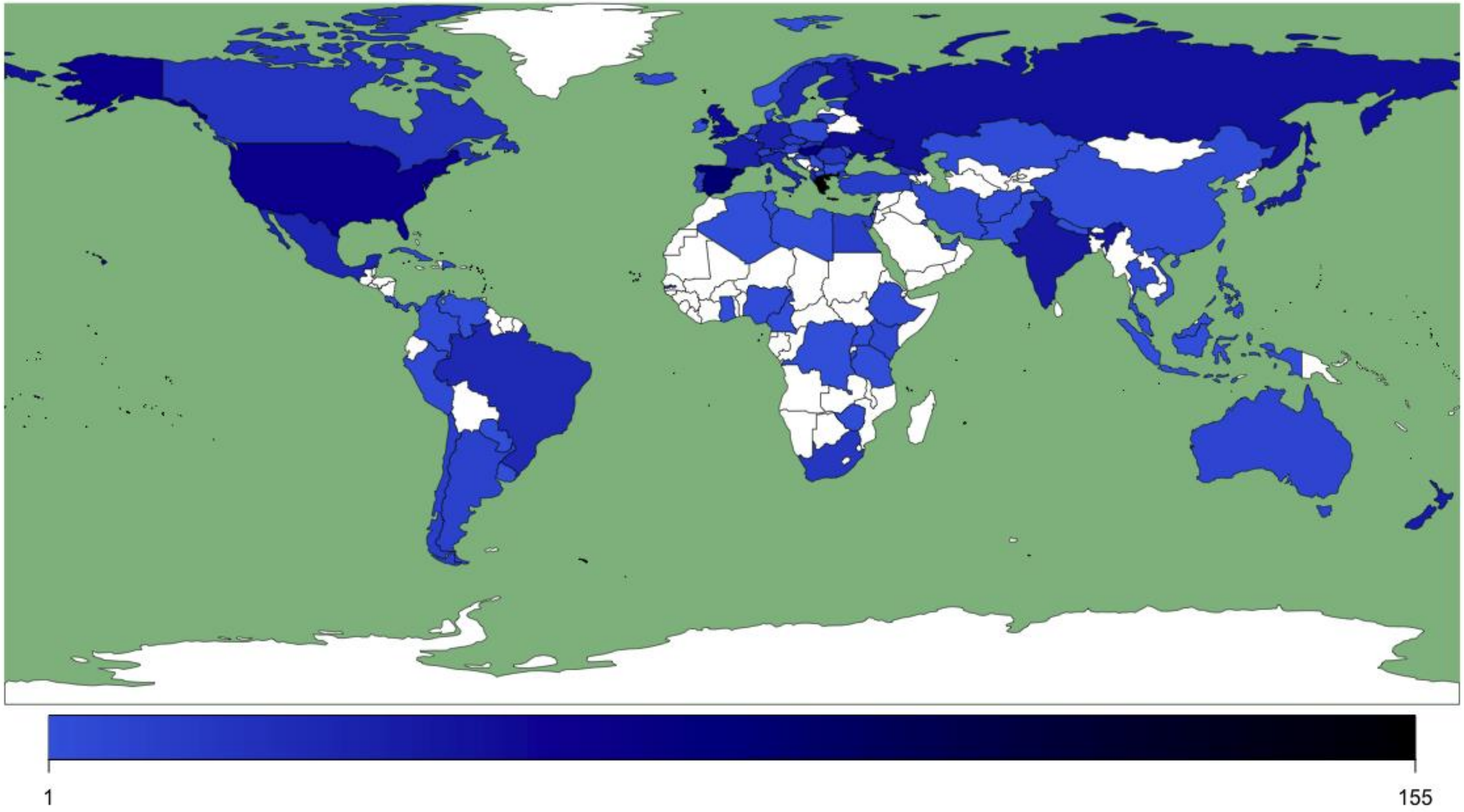

© 2023 Papadopoulos NG et al. *JAMA Network Open.*

eFigure 4. Participants by Country of Residence (Europe)

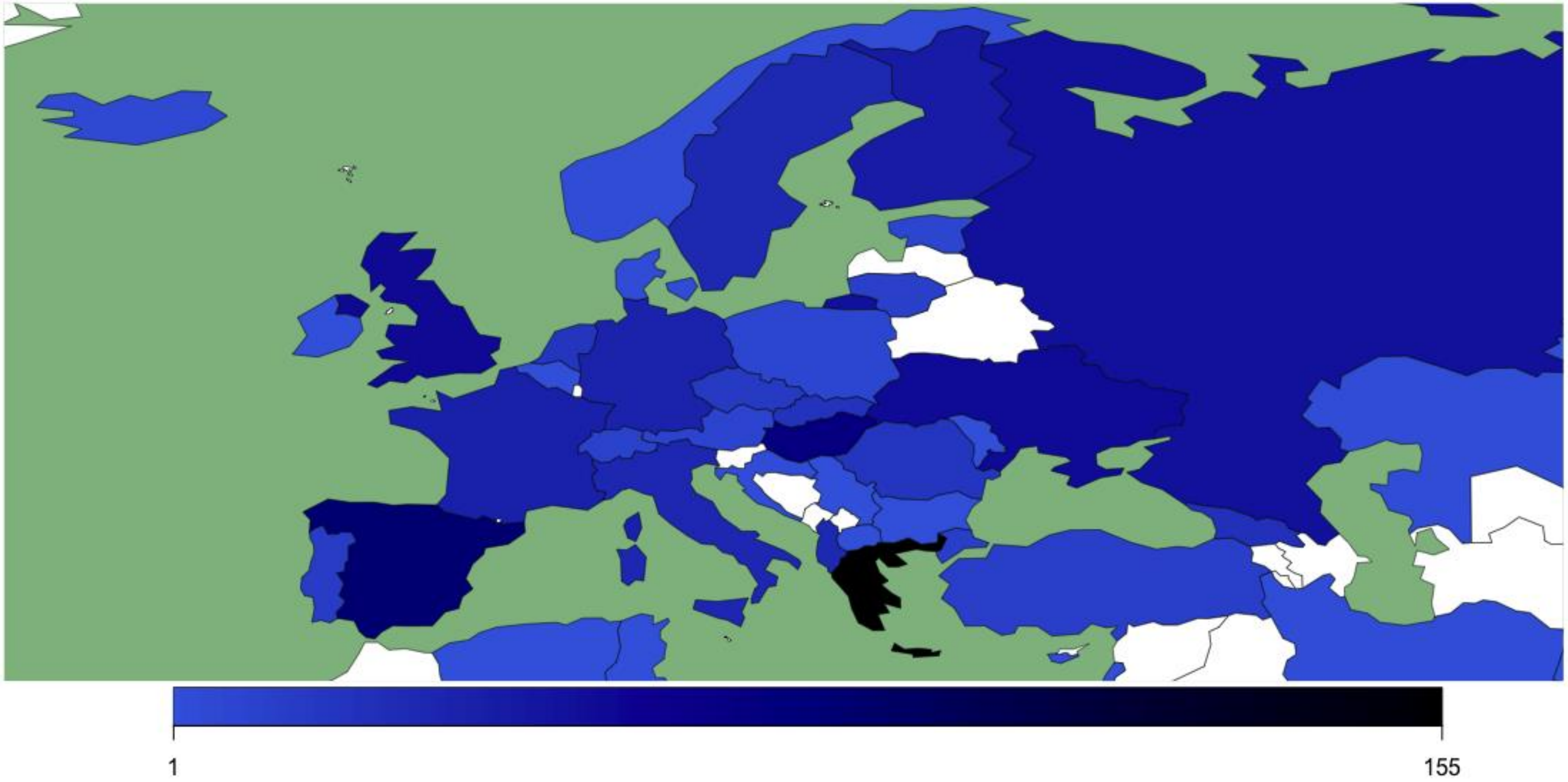

© 2023 Papadopoulos NG et al. *JAMA Network Open.*

eFigure 5. Participants by Country of Residence

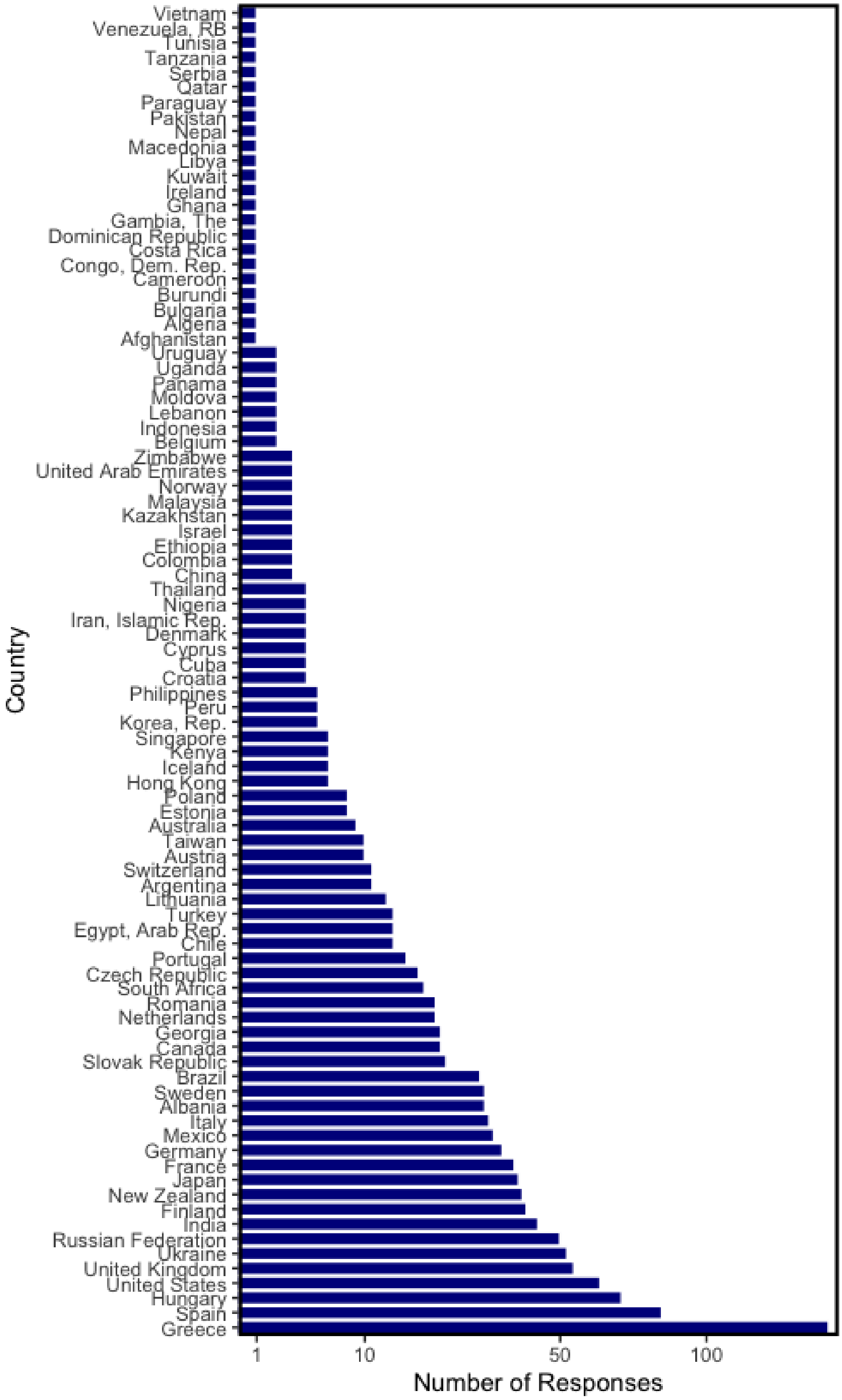

© 2023 Papadopoulos NG et al. *JAMA Network Open.*

Use of monitoring tools during asthma monitoring visit

eFigure 6. Actual and Perceived Optimal Monitoring Visit Frequency (in Months) and Duration (in Minutes) for Children With Mild or Moderate Asthma

Differentiation of respondents between real-life and optimal conditions, is shown as lines.

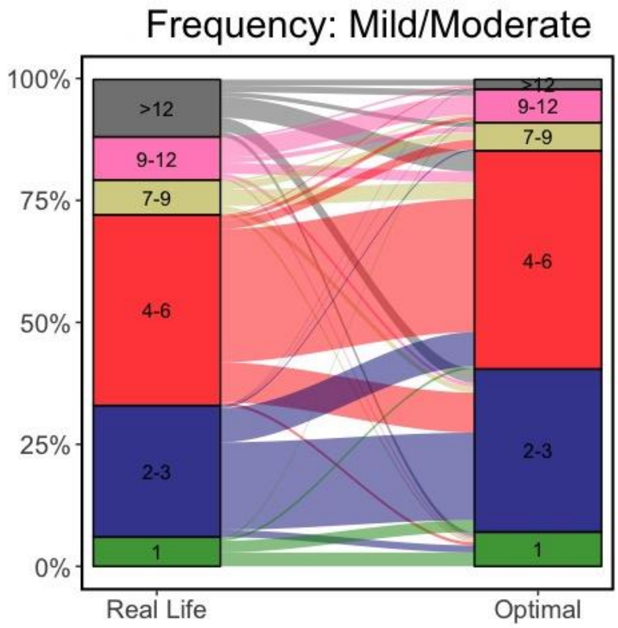

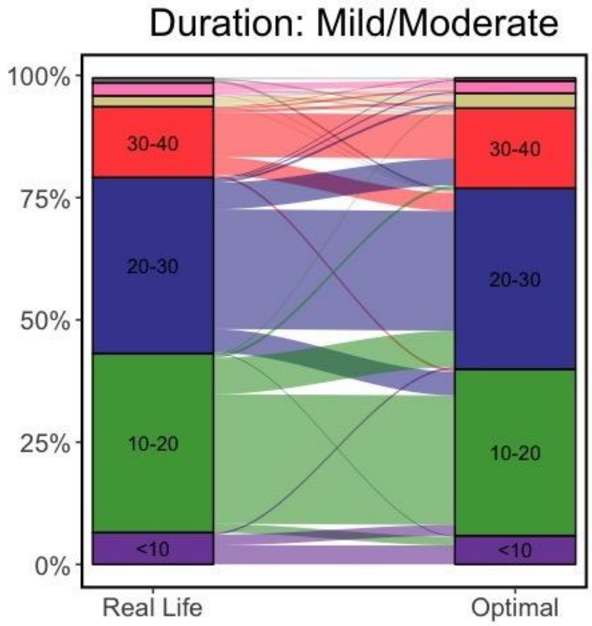

© 2023 Papadopoulos NG et al. *JAMA Network Open.*

eFigure 7. Actual (Light Blue, Left Side of the Violin Plots) and Perceived Optimal (Green, Right Side of the Violin Plots) Use of Monitoring Tools During Asthma Monitoring Visits

Medians and interquartile ranges are shown as lines and black bars, respectively. ** = $p<0.01$, *** = $p<0.001$, in comparing real-life with optimal conditions

© 2023 Papadopoulos NG et al. *JAMA Network Open.*

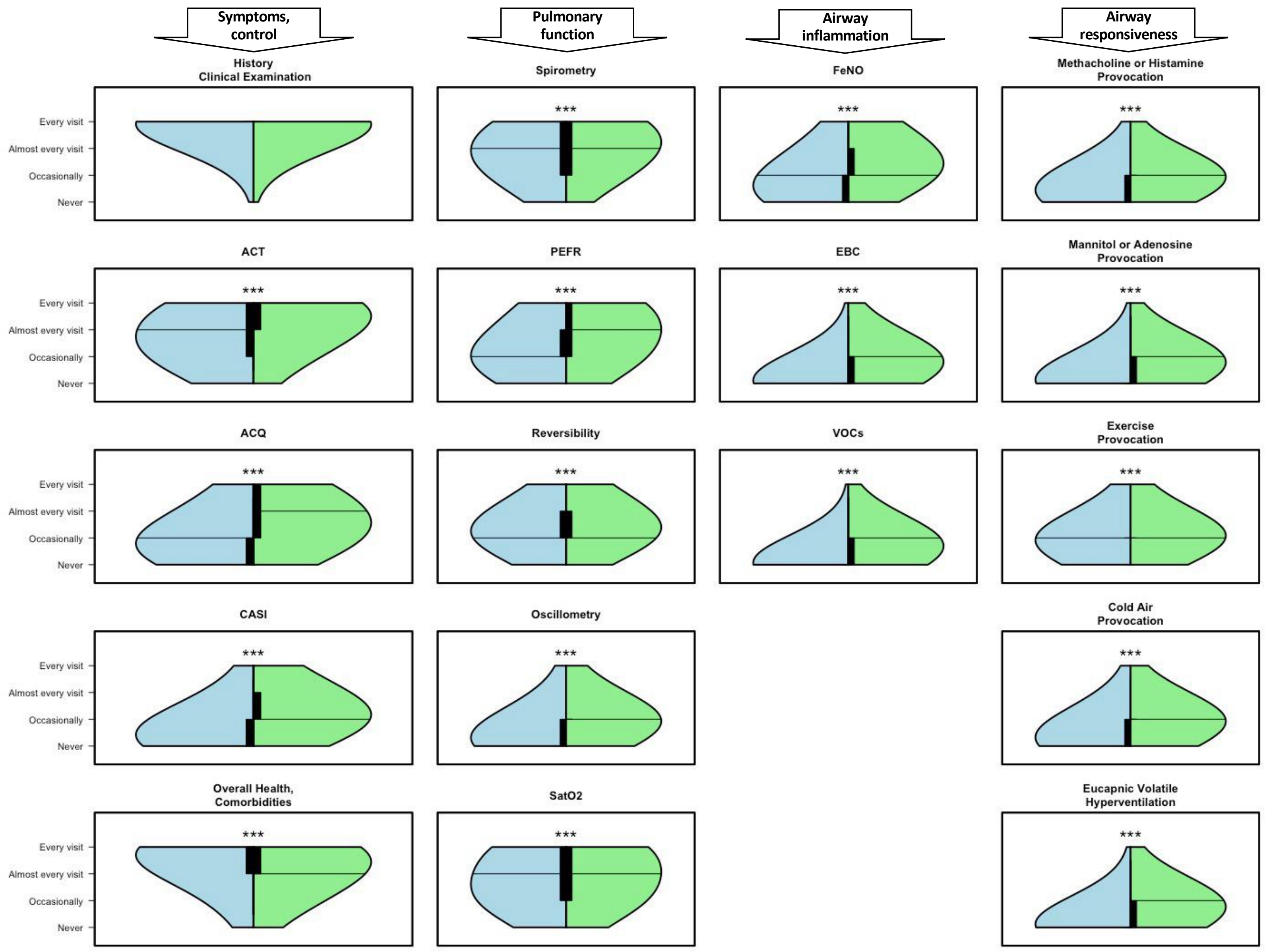

© 2023 Papadopoulos NG et al. *JAMA Network Open.*

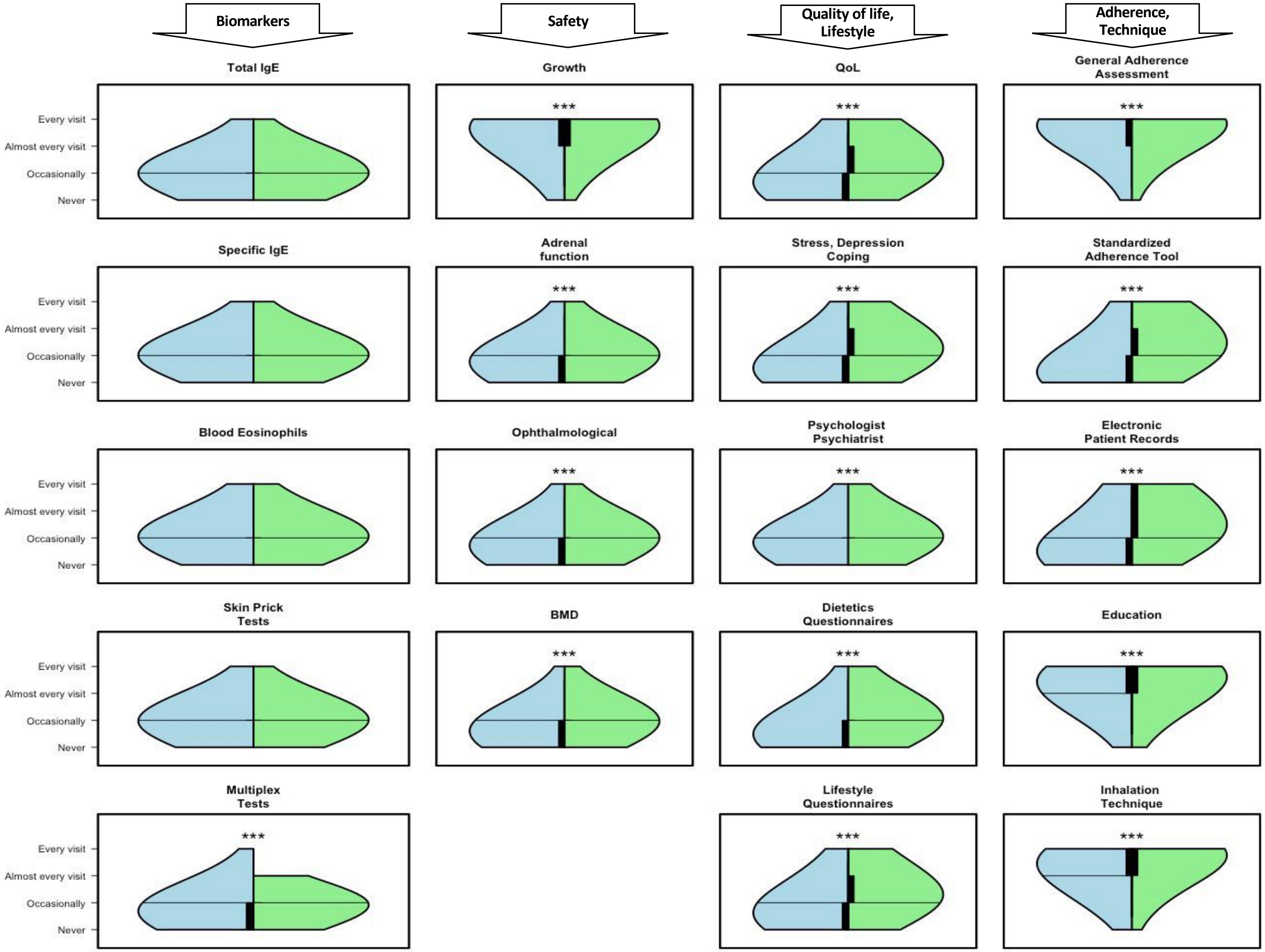

© 2023 Papadopoulos NG et al. *JAMA Network Open.*

Optimal use of childhood asthma monitoring tools

eTable 2. Heatmap Summarising the Proportion of Respondents That Would Prefer to Use Each Monitoring Tool More Frequently

Lower, intermediate and high percentages of respondents are highlighted in green, yellow and red colour, respectively. *: p<0.05, #: p <0.001

Monitoring during visits: Techniques	Respondents supporting intensification (%)	Never users that would like to frequently or always use the technique
**Symptoms & Control**		
History and Clinical Examination	4.99%	2/2 (100%)
Asthma Control Test (ACT)	42.3%#	28/109 (25.69%)
Asthma Control Questionnaire (ACQ)	48.69%#	88/351 (25.07%)
Composite Asthma Severity Score (CASI)	57.08%#	110/592 (18.58%)
Other Standardised Questionnaire	49.49%#	76/541 (14.05%)
Overall Health and Comorbidities	13.81%#	18/48 (37.5%)
**Lung Function**		
Spirometry	28.08%#	18/76 (23.68%)
Peak Expiratory Flow Rate (PEFR)	45.47%#	49/232 (21.12%)
Reversibility testing	24.36%#	8/69 (11.59%)
Oscillometry	51.05%#	37/700 (5.29%)
Plethysmography	45.88%#	24/687 (3.49%)
Oxygen Saturation	22.34%#	12/83 (14.46%)
**Inflammation**		
Fractional Exhaled Nitric Oxide (FeNO test)	53.63%#	92/476 (19.33%)
Exhaled Breath Condensate (EBC)	61.51%#	50/970 (5.15%)
Volatile Organic Compounds (VOCs)	58.3%#	22/1025 (2.15%)

© 2023 Papadopoulos NG et al. *JAMA Network Open.*

**Bronchial hyperresponsiveness**		
Methacholine or Histamine Challenge	41.58%#	11/605 (1.82%)
Mannitol or Adenosine Challenge	56.84%#	15/919 (1.63%)
Exercise Challenge	22.39%#	14/207 (6.76%)
Cold Air Challenge	50.99%#	20/698 (2.87%)
Eucapnic Volatile Hyperventilation	58.56%#	17/944 (1.8%)
**Biomarkers**		
Total IgE	6.57%	0/86 (0%)
Specific IgE	5.3%	0/51 (0%)
Blood Counts (Blood eosinophils - EOS)	7.86%	0/56 (0%)
Skin Prick Tests	9.91%*	4/128 (3.12%)
Multiplex Tests	21.59%#	1/397 (0.25%)
**Adverse events**		
Growth	20.58%#	4/12 (33.33%)
Adrenal function	30.6%#	8/317 (2.52%)
Ophthalmological assessment	35.27%#	4/367 (1.09%)
Bone Mineral Density (BMD)	38.78%#	4/473 (0.85%)
**Quality of Life**		
Quality of Life	55.71%#	106/425 (24.94%)
Stress, Depression and Coping	60.06%#	111/520 (21.35%)
Psychologist or Psychiatrist Assessment	27.8%#	20/250 (8%)
Nutritionist Assessment	23.95%#	13/159 (8.18%)
Diet Questionnaires	55.42%#	44/564 (7.8%)
Lifestyle Questionnaires	57.08%#	91/458 (19.87%)
**Adherence & Education**		
General Adherence Assessment	15.51#	8/17 (47.06%)
Standardised Adherence Evaluation	64.85#	181/614 (29.48%)
Electronic Patient Records	60.93#	148/522 (28.35%)
Education of Patient and Family Members	23.58#	8/18 (44.44%)
Inhalation Technique	31.66#	4/10 (40%)

© 2023 Papadopoulos NG et al. *JAMA Network Open.*

Responses grouped by (a) country income and (b) health care setting of the respondents

eFigure 8. Prioritisation of Different Domains of Asthma Monitoring During Follow-up Visits, Stratified by Country Income

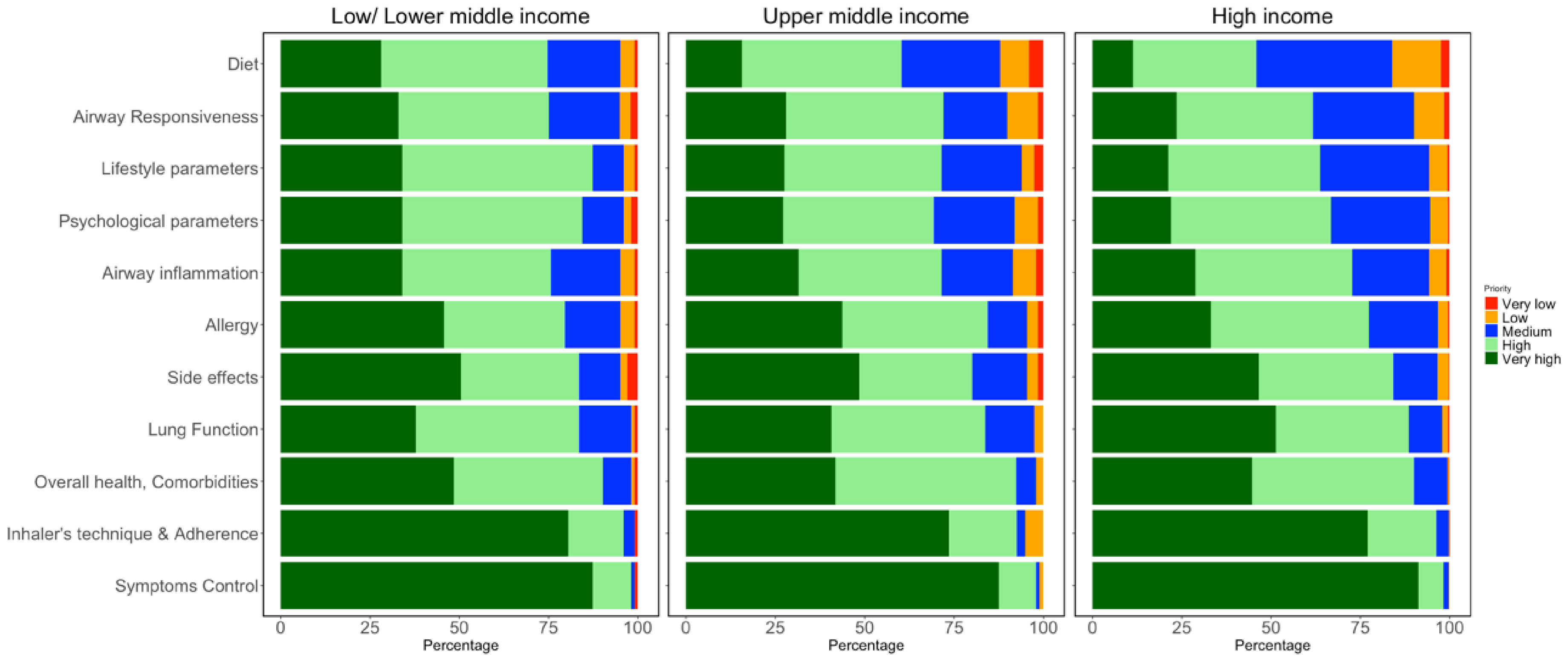

© 2023 Papadopoulos NG et al. *JAMA Network Open.*

eFigure 9. Prioritisation of Different Domains of Asthma Monitoring During Follow-up Visits, Stratified by Care Setting

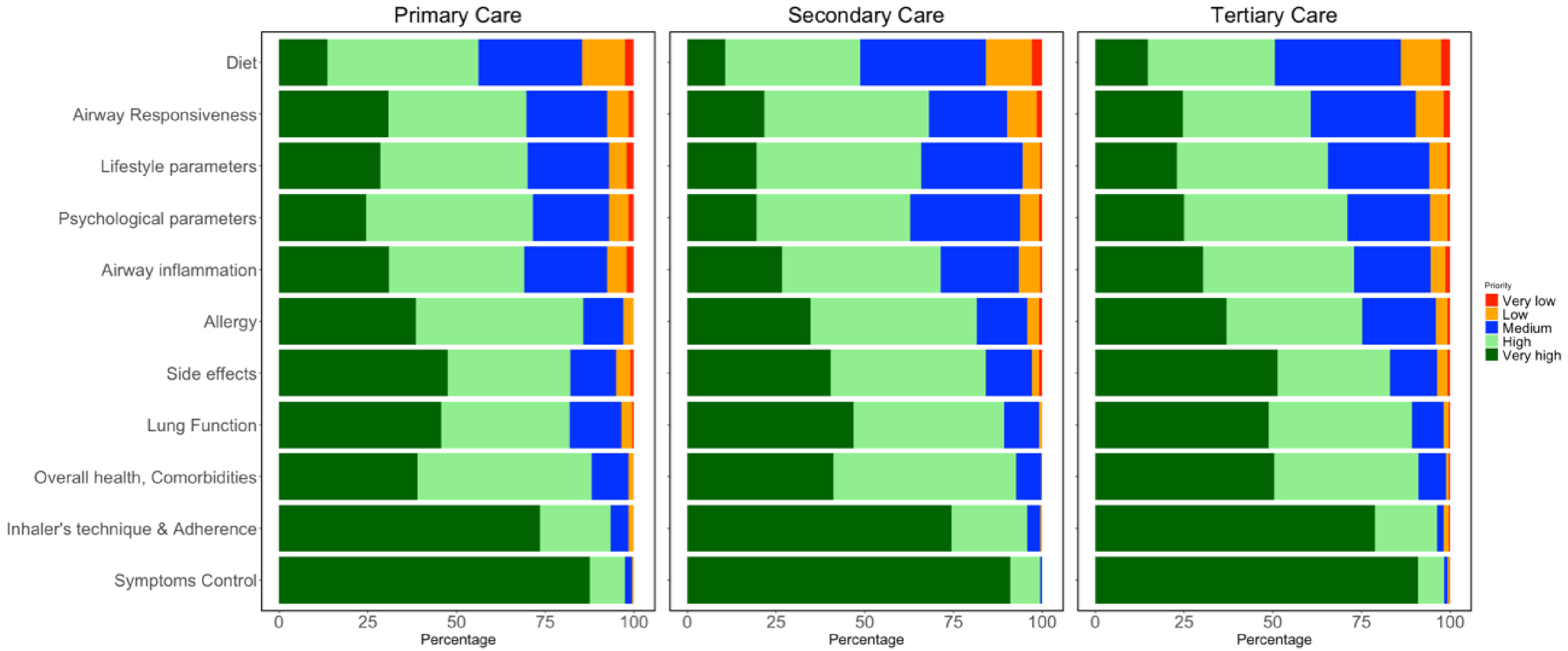

© 2023 Papadopoulos NG et al. *JAMA Network Open.*

eFigure 10. Prioritisation of Different Domains of Asthma Monitoring Between Follow-up Visits, Stratified by Country Income

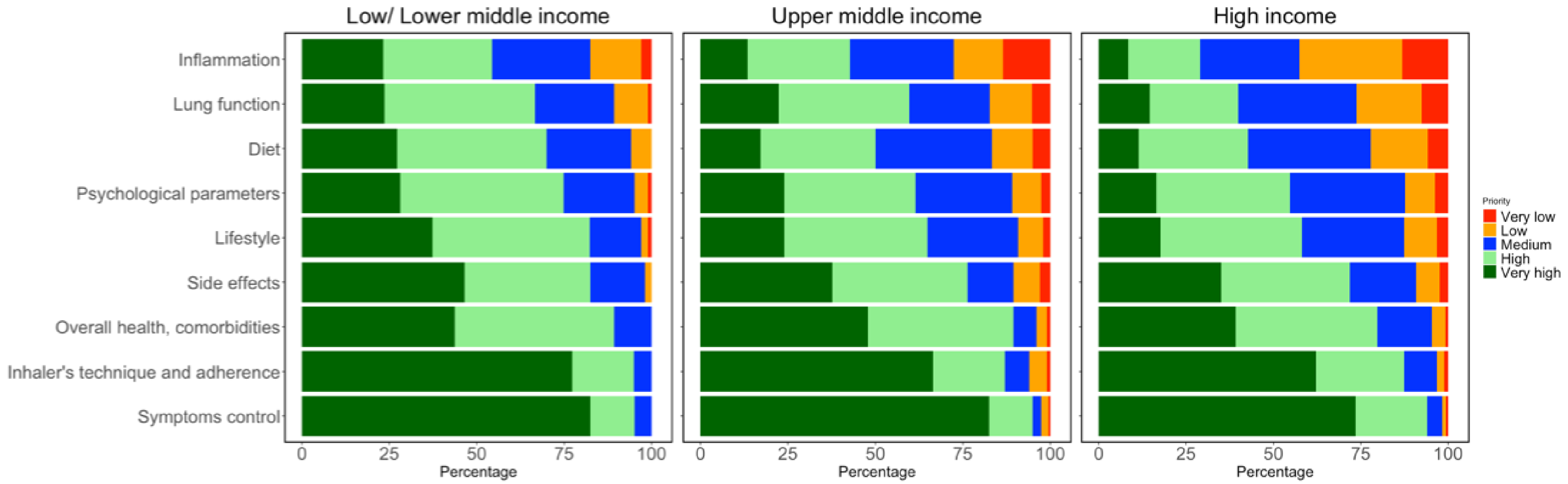

© 2023 Papadopoulos NG et al. *JAMA Network Open.*

eFigure 11. Prioritisation of Different Domains of Asthma Monitoring Between Follow-up Visits, Stratified by Care Setting

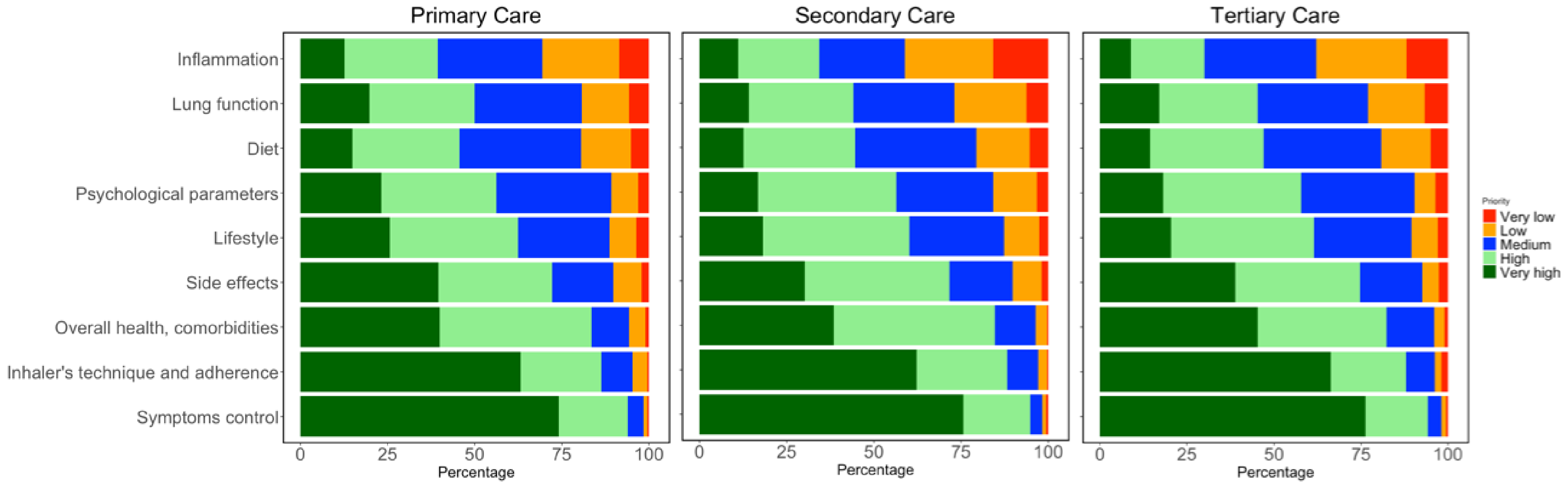

© 2023 Papadopoulos NG et al. *JAMA Network Open.*

eFigure 12. Actual (Left) and Perceived Optimal (Right) Monitoring Visit Frequency (in Months), for Children With Mild/Moderate and With Severe Asthma, Stratified by the Country Income and Health Care Setting of the Respondents
*: p<0.05; **: p<0.01; ***: p<0.001

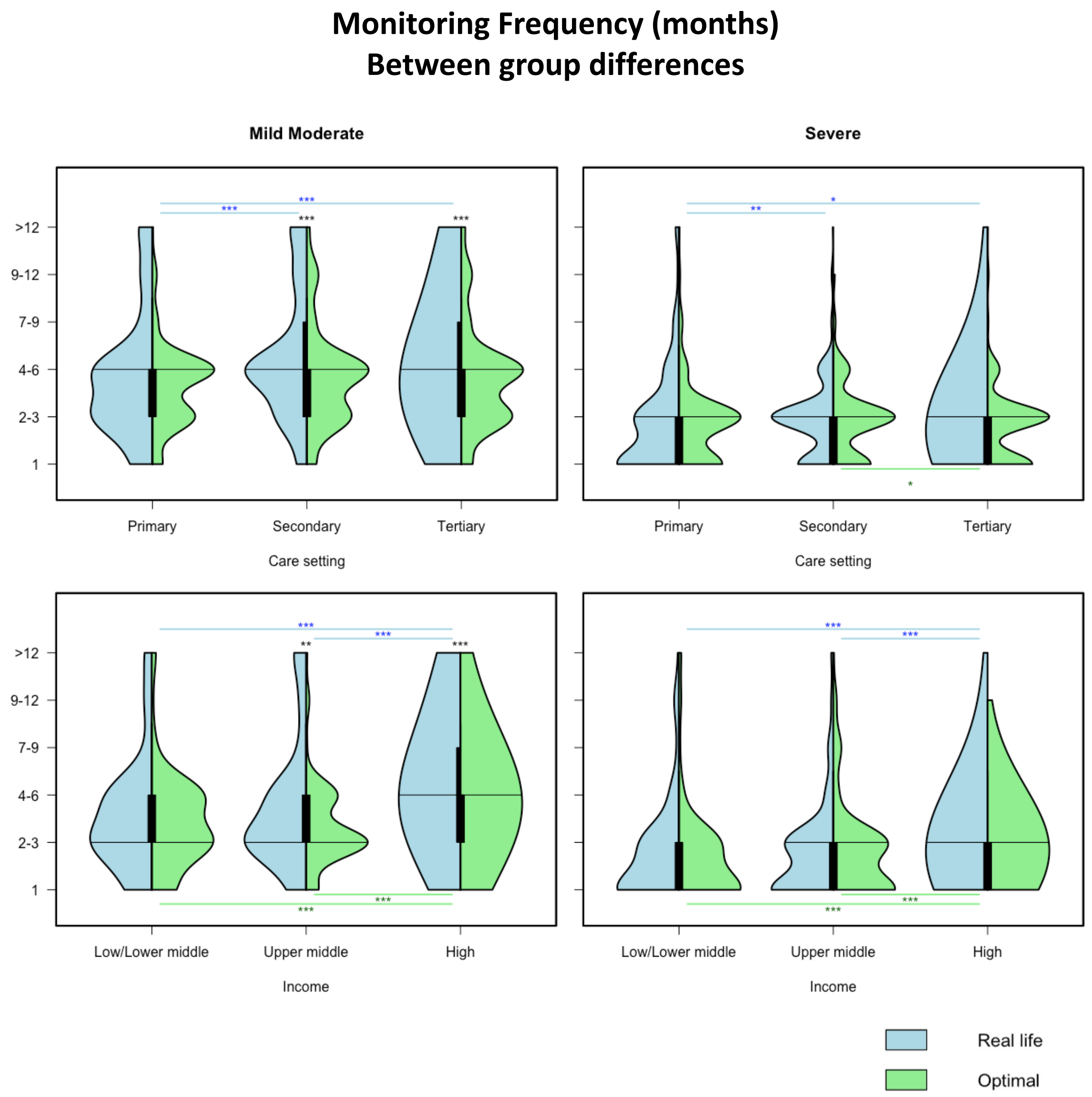

© 2023 Papadopoulos NG et al. *JAMA Network Open.*

eFigure 13. Actual (Left) and Perceived Optimal (Right) Monitoring Visit Duration (in Minutes), for Children With Mild/Moderate and With Severe Asthma, Stratified by the Country Income and Health Care Setting of the Respondents
*: p<0.05; **: p<0.01; ***: p<0.001

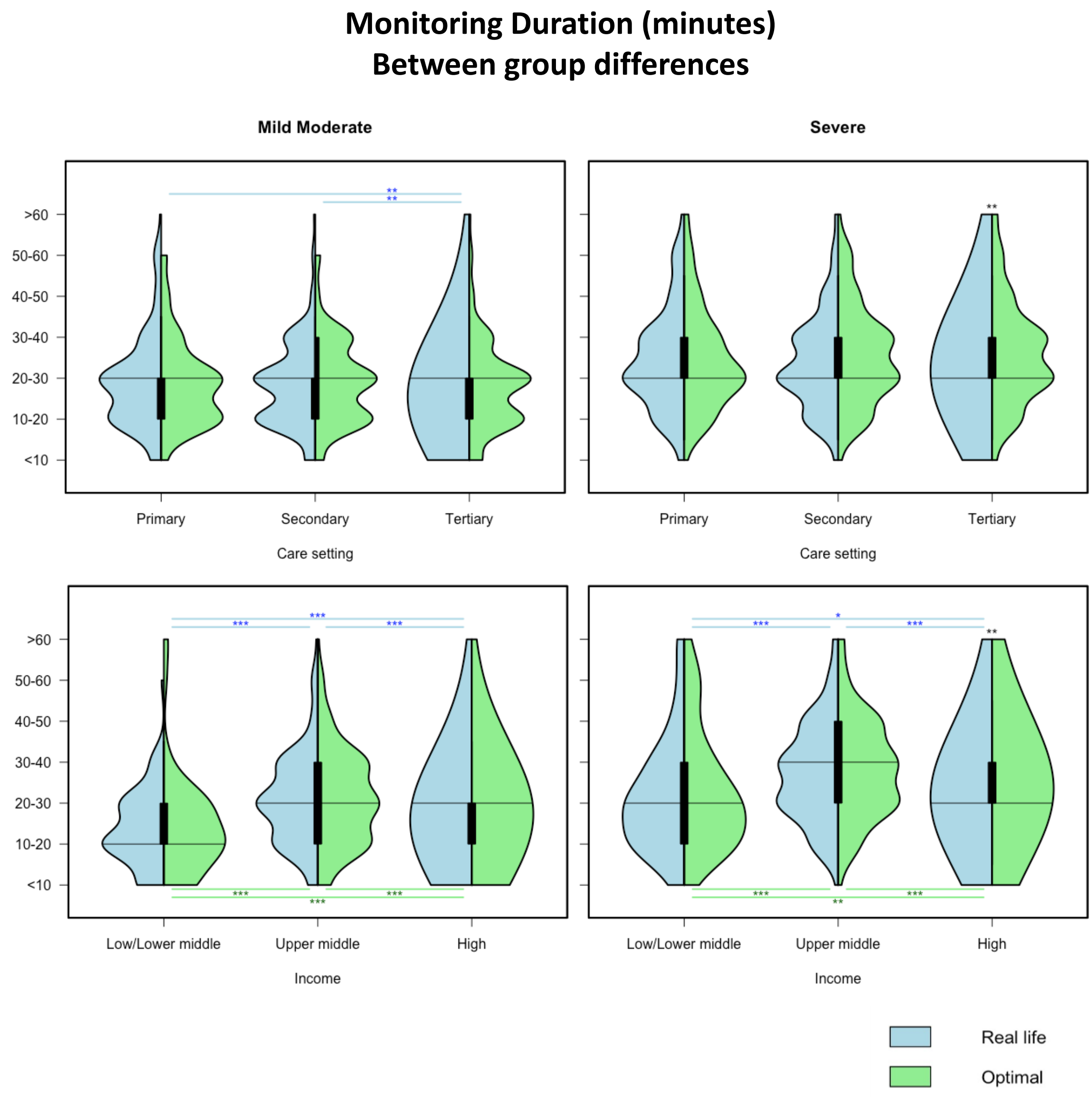

© 2023 Papadopoulos NG et al. *JAMA Network Open.*

eFigure 14. Actual (Light Blue, Left Side of the Violin Plots) and Perceived Optimal (Green, Right Side of the Violin Plots) Use of Monitoring Tools During Asthma Monitoring Visits, Stratified by Country Income

*: p<0.05; **: p<0.01; ***: p<0.001

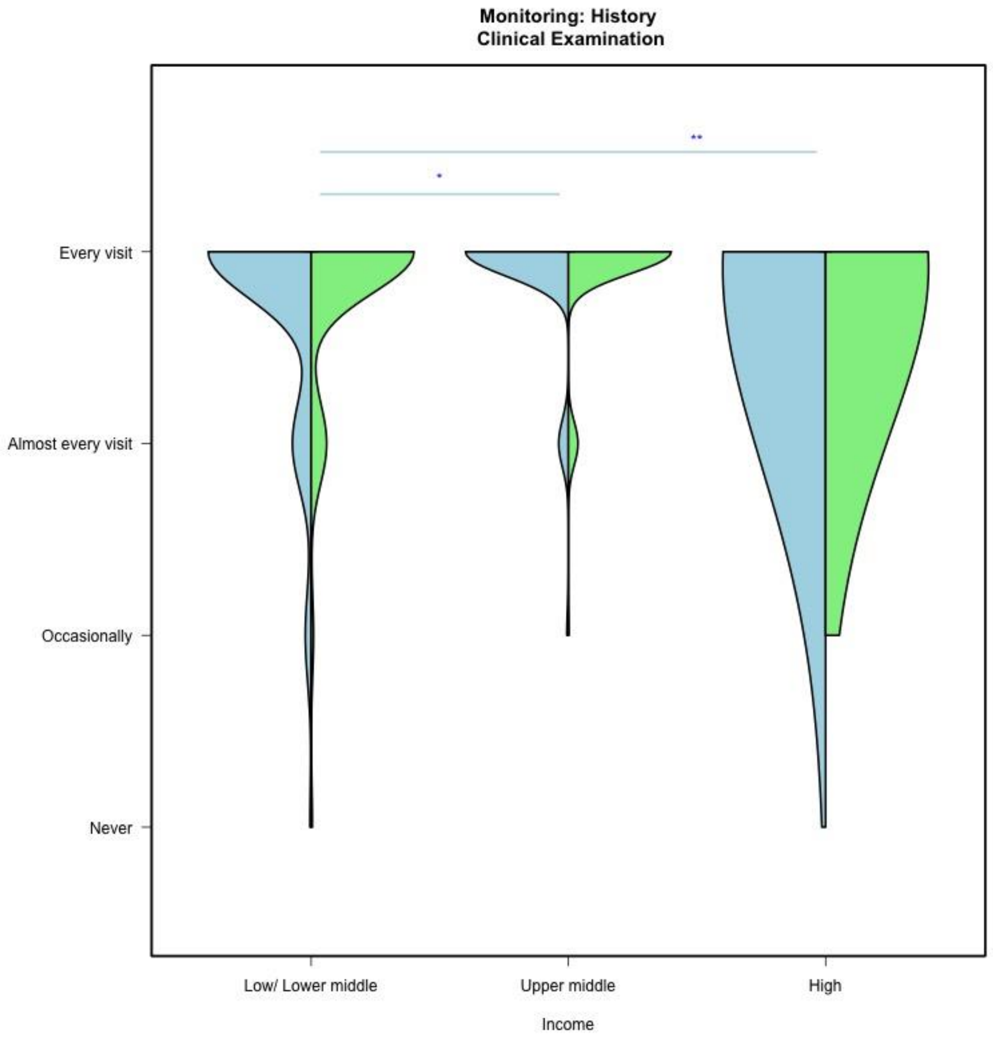

© 2023 Papadopoulos NG et al. *JAMA Network Open.*

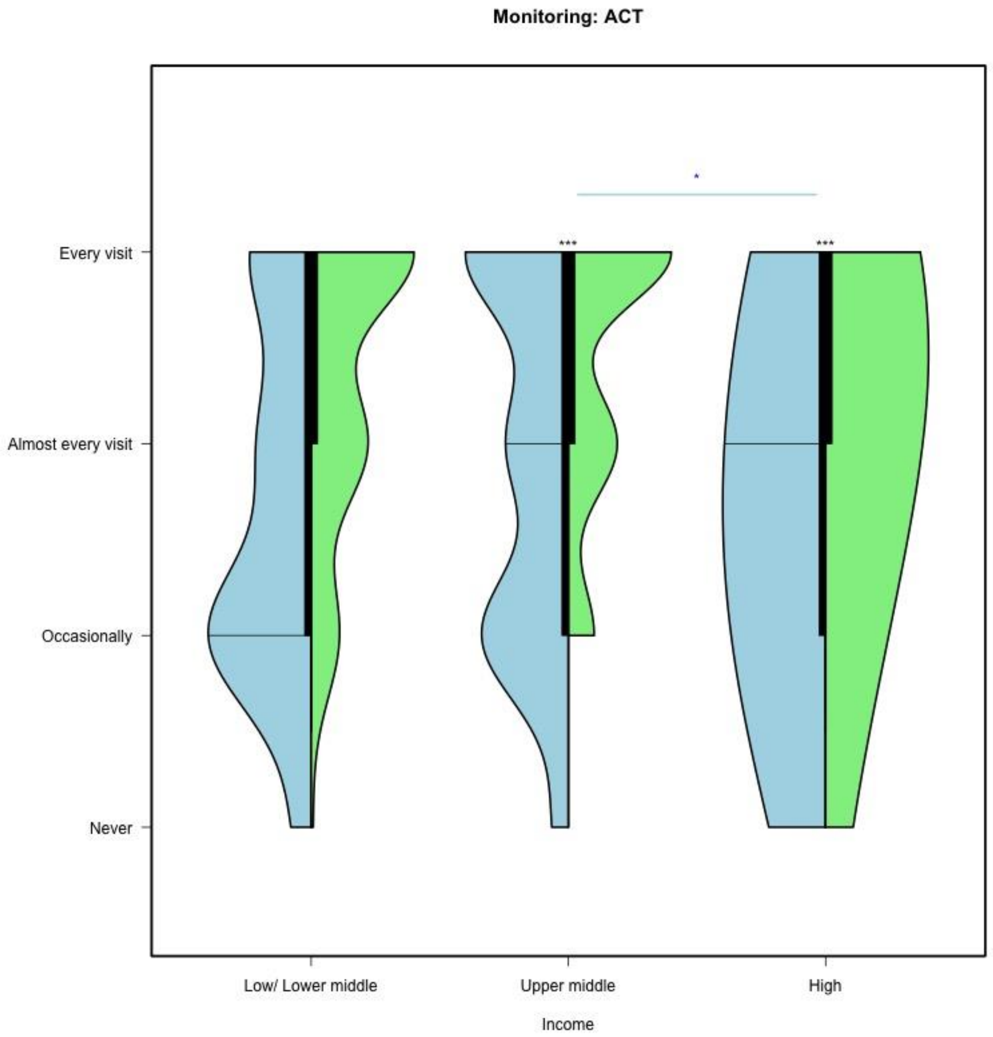

© 2023 Papadopoulos NG et al. *JAMA Network Open.*

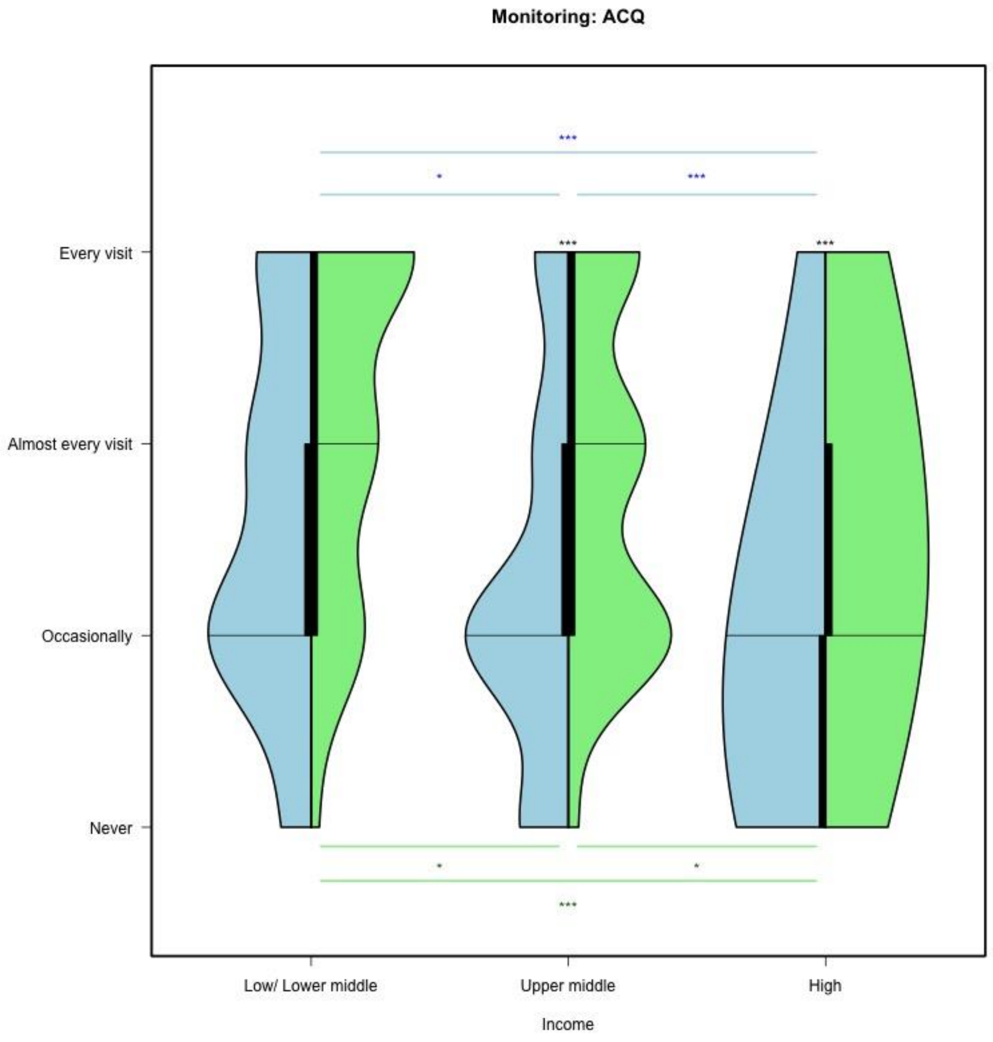

© 2023 Papadopoulos NG et al. *JAMA Network Open.*

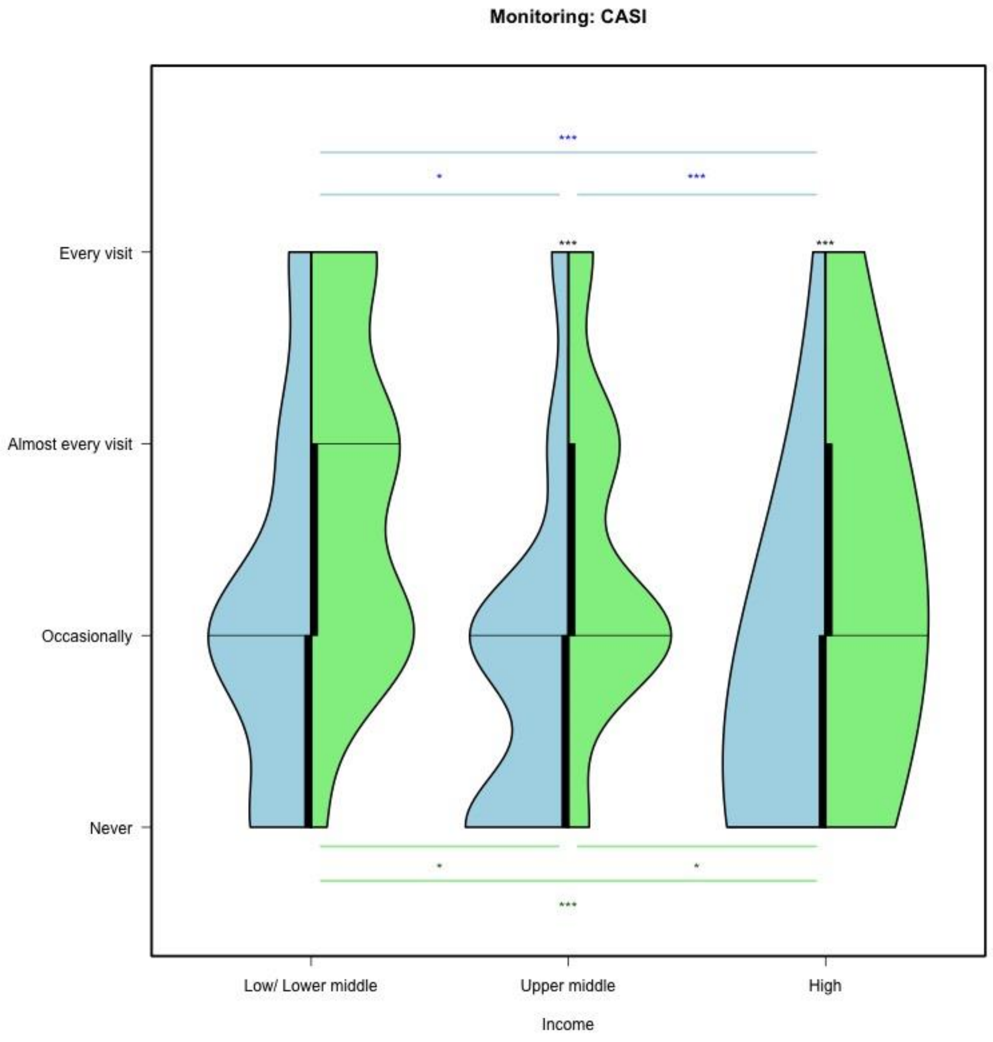

Monitoring: CASI
***
*
***
Every visit
Almost every visit
Occasionally
Never
***
***
*
*
***
Low/ Lower middle
Upper middle
High
Income

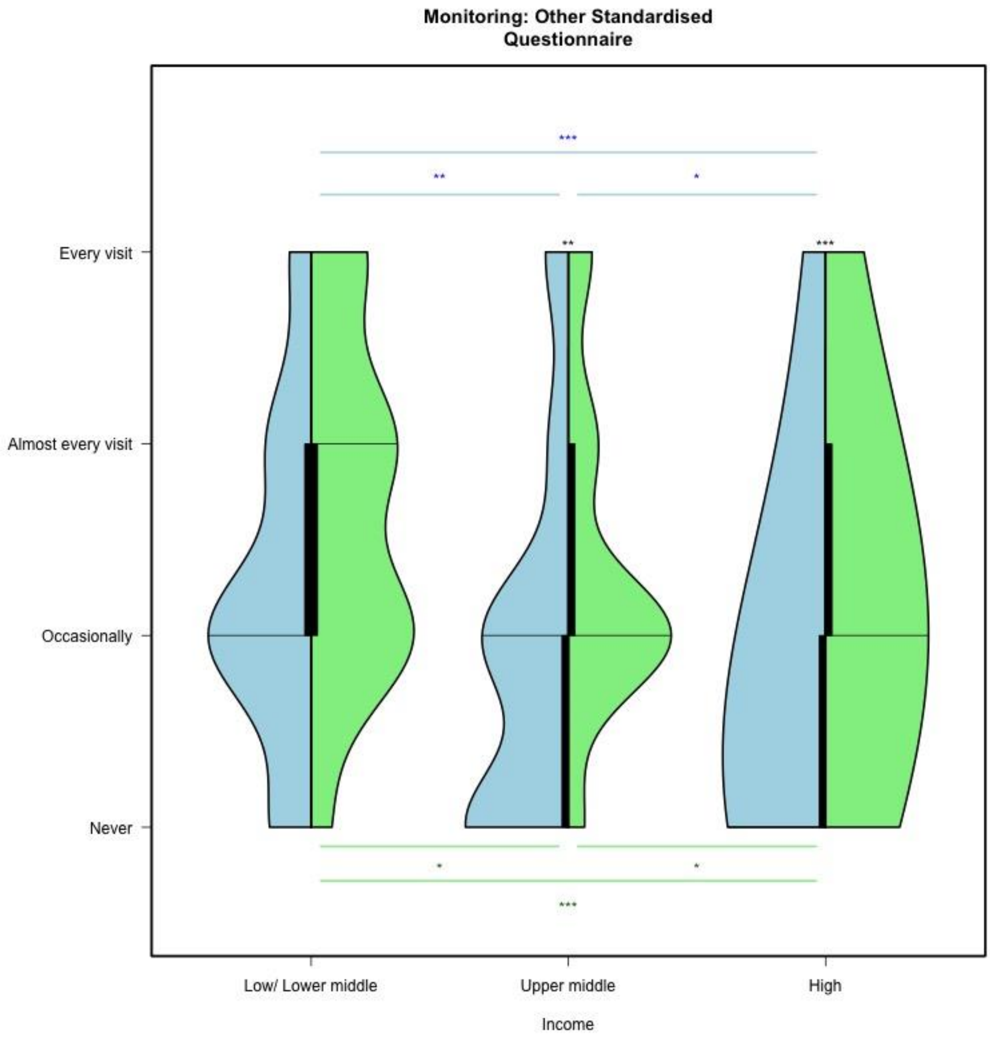

© 2023 Papadopoulos NG et al. *JAMA Network Open.*

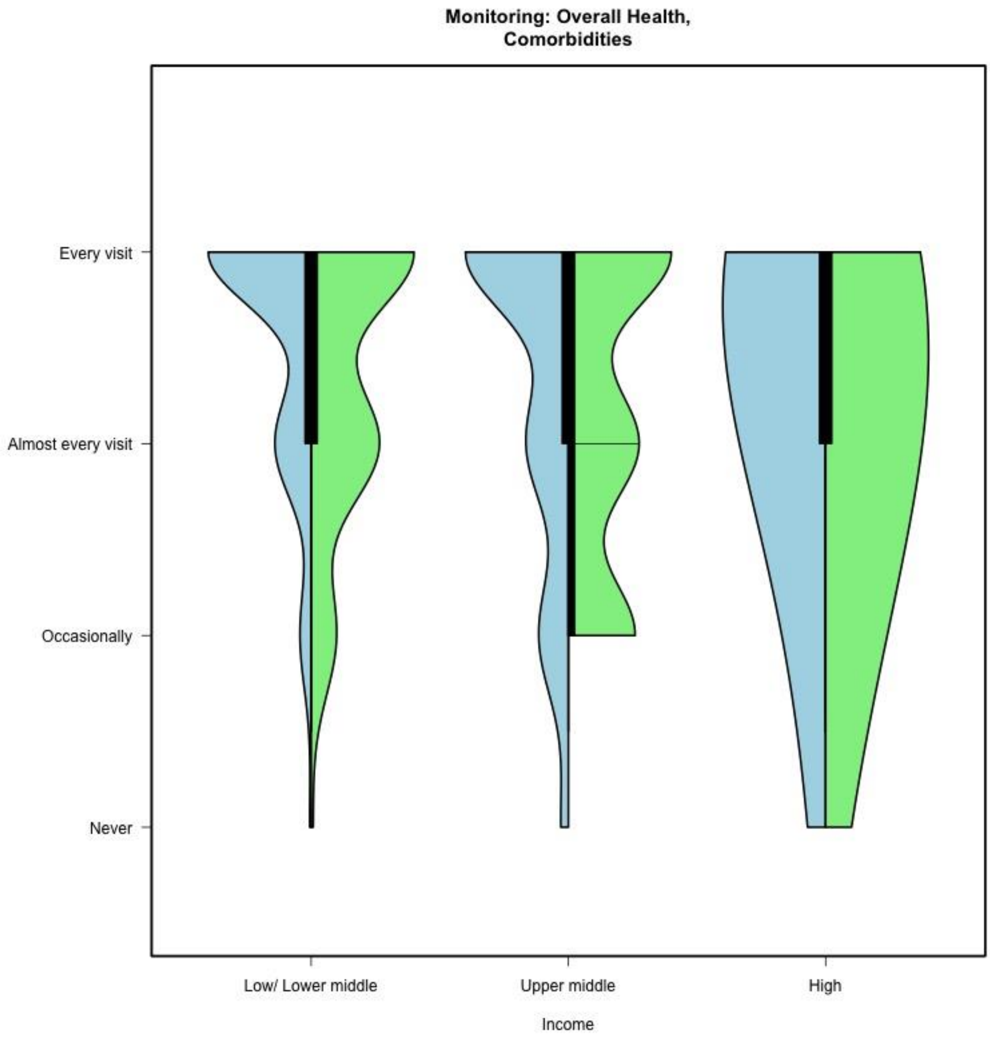

© 2023 Papadopoulos NG et al. *JAMA Network Open.*

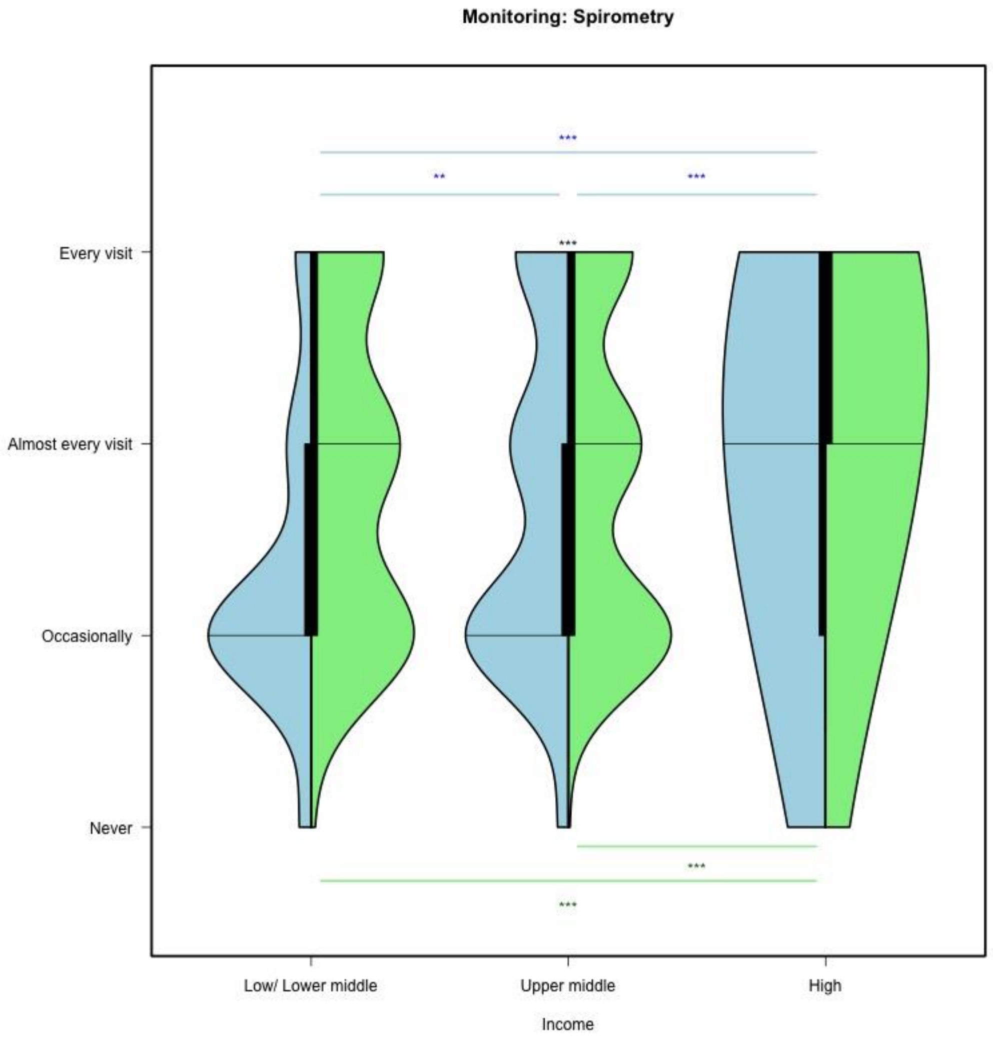

© 2023 Papadopoulos NG et al. *JAMA Network Open.*

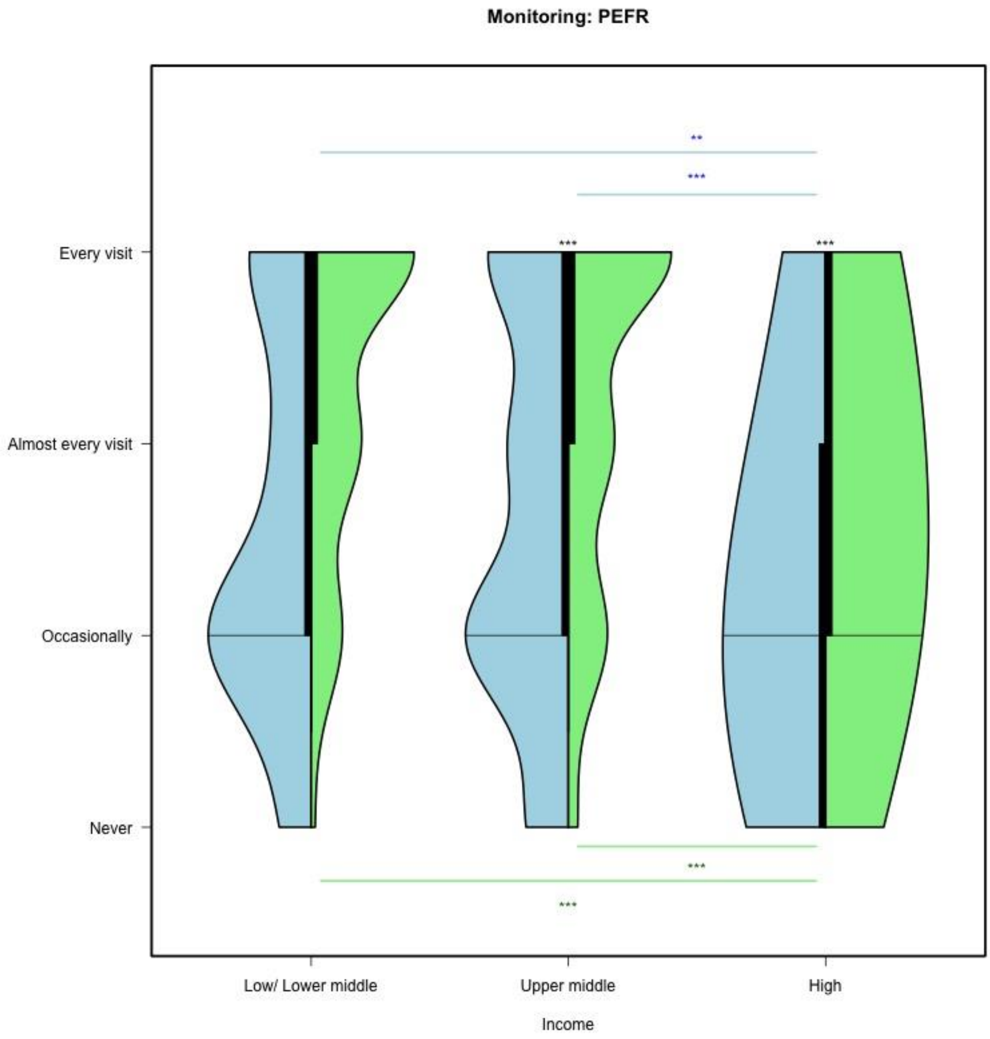

© 2023 Papadopoulos NG et al. *JAMA Network Open.*

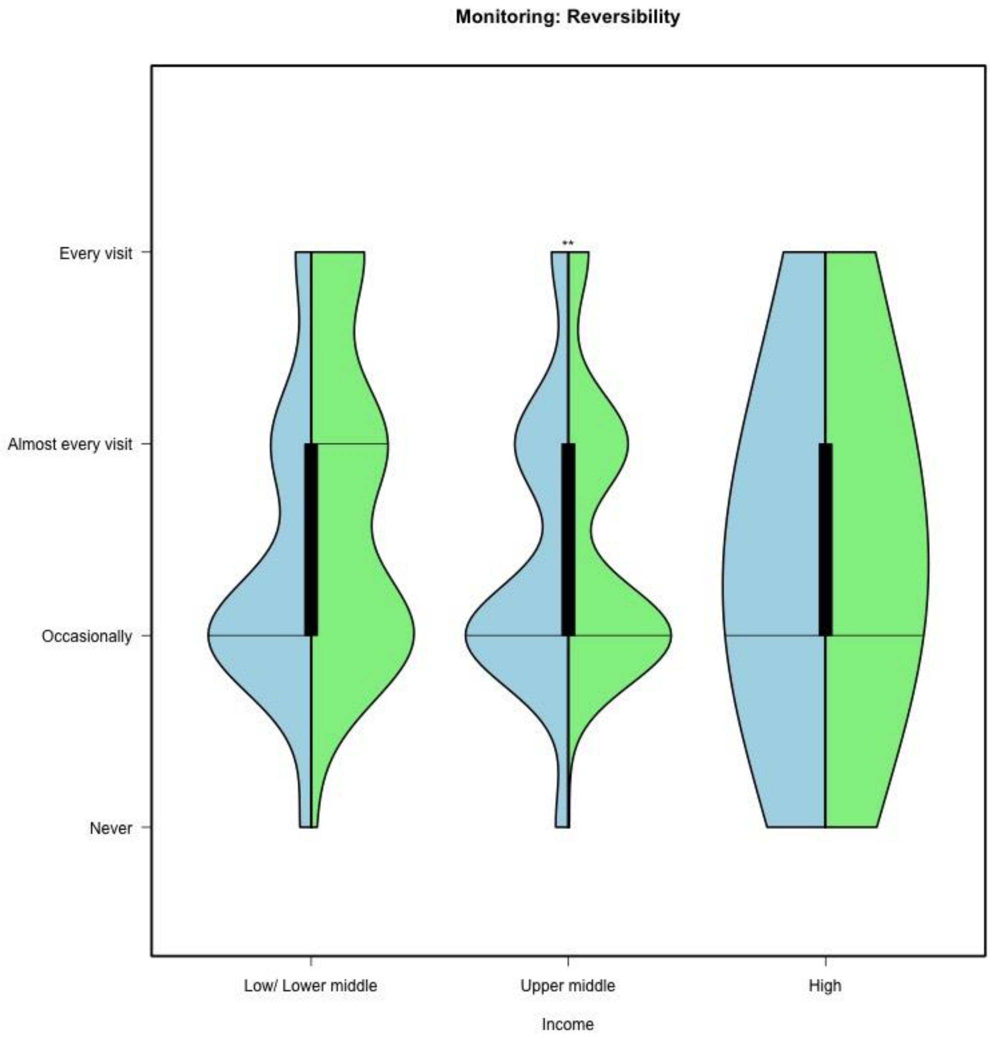

© 2023 Papadopoulos NG et al. *JAMA Network Open.*

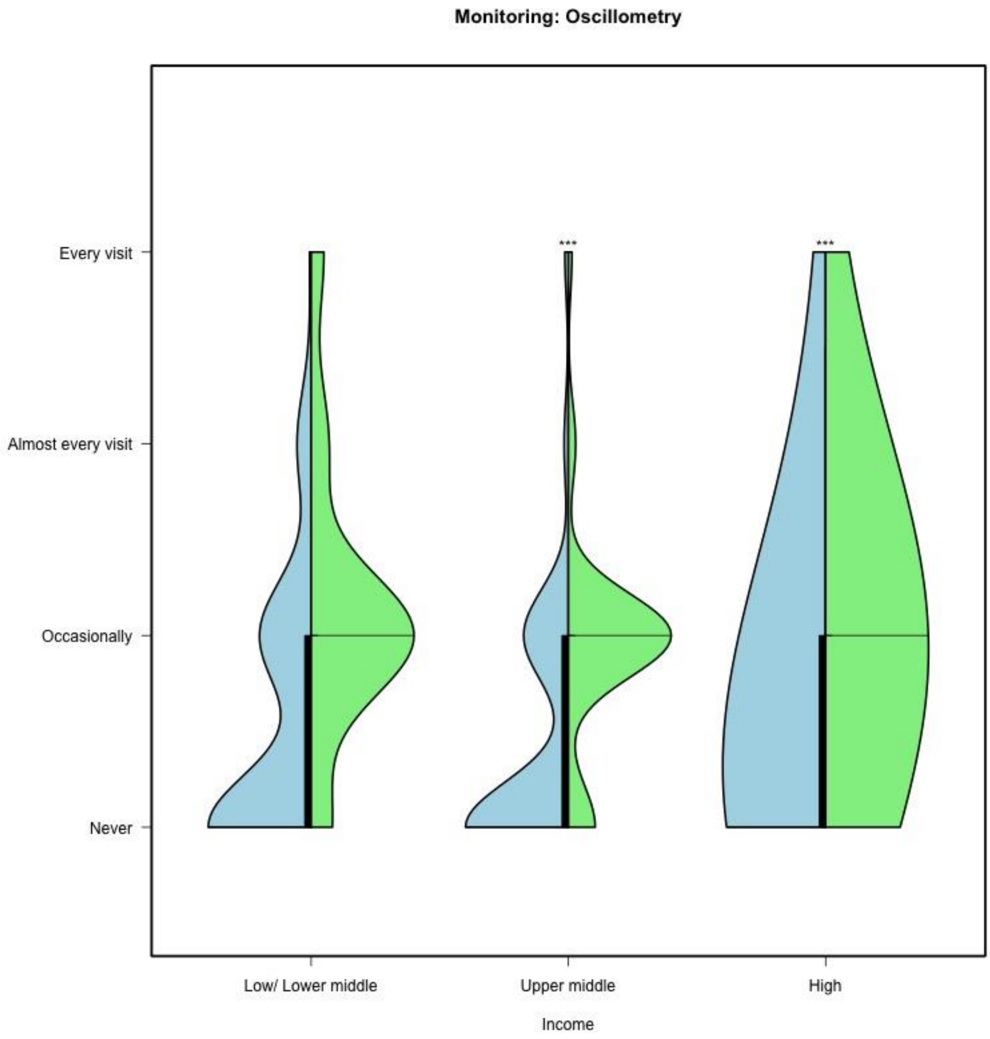

© 2023 Papadopoulos NG et al. *JAMA Network Open.*

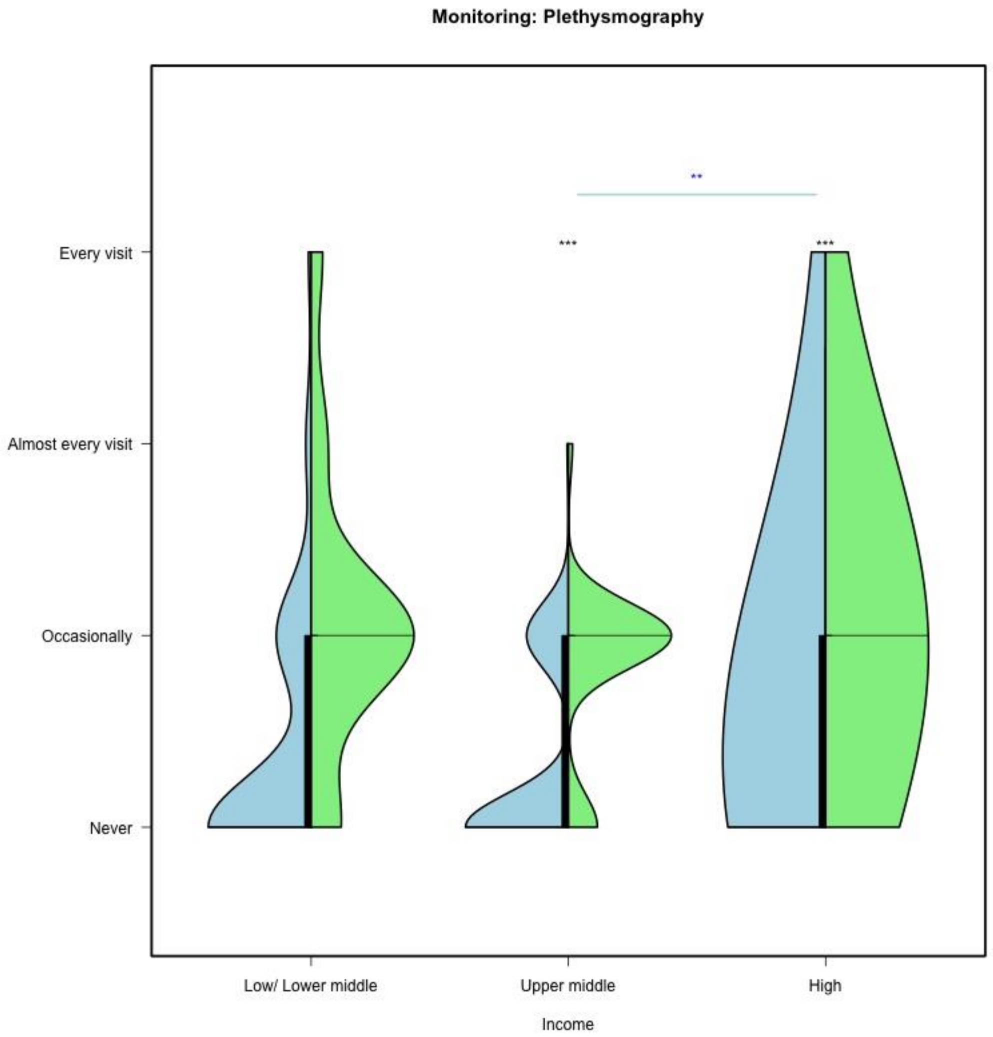

© 2023 Papadopoulos NG et al. *JAMA Network Open.*

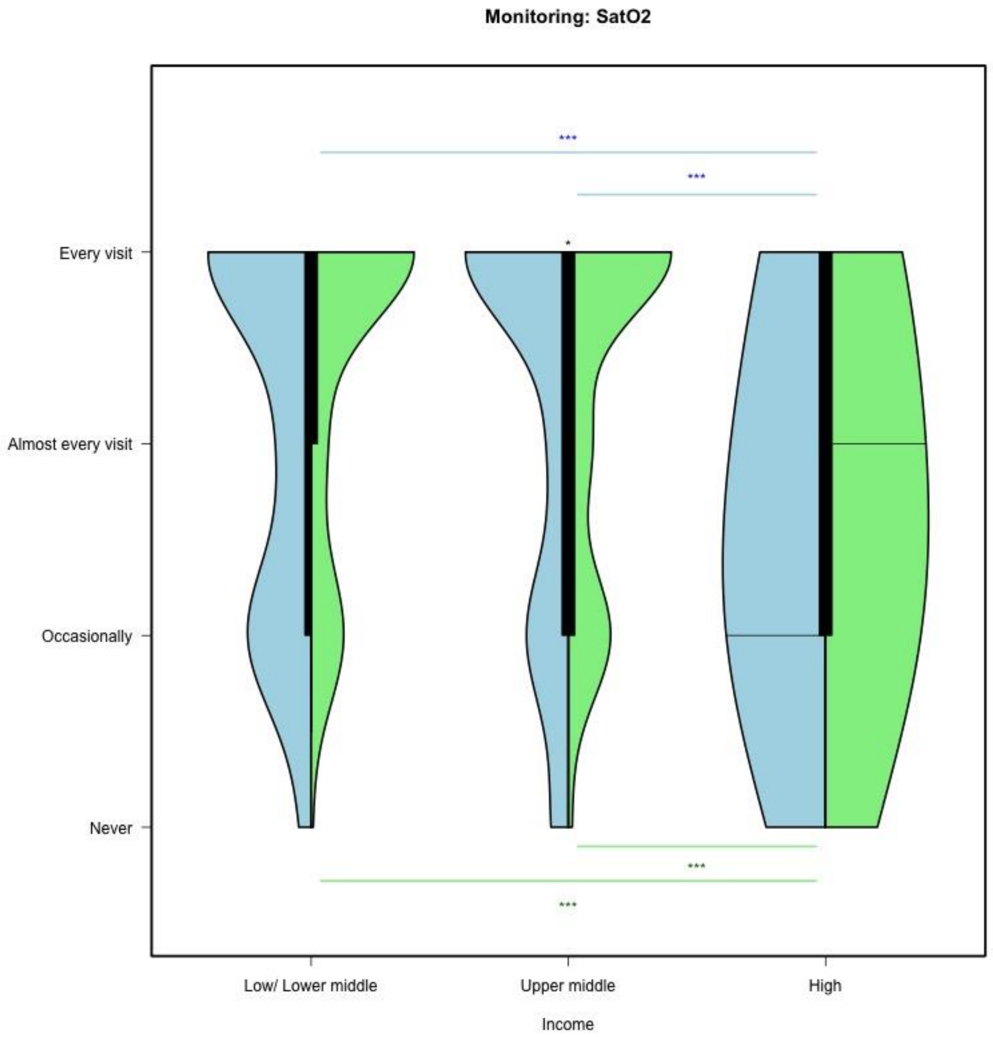
Monitoring: SatO2
***
***
*
Every visit
Almost every visit
Occasionally
Never
***
***
Low/ Lower middle
Upper middle
High
Income

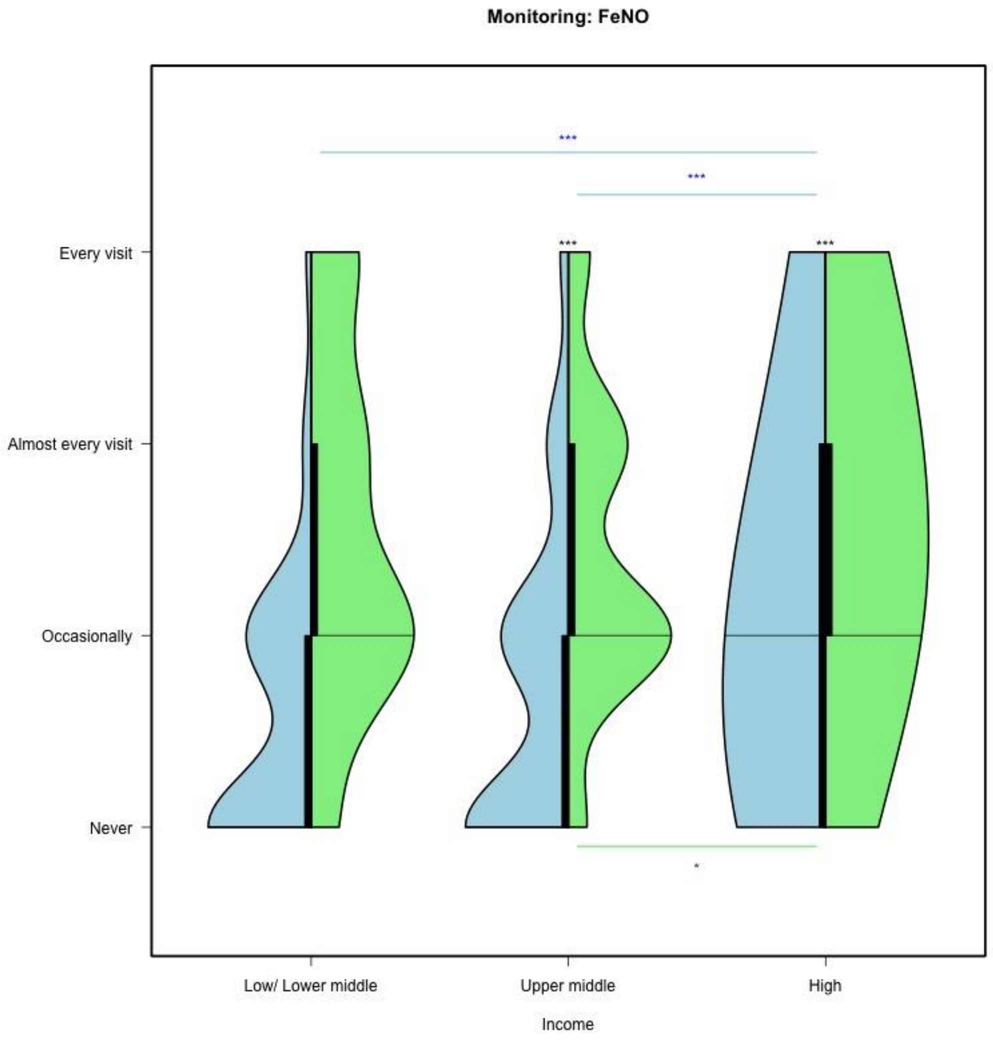

© 2023 Papadopoulos NG et al. *JAMA Network Open.*

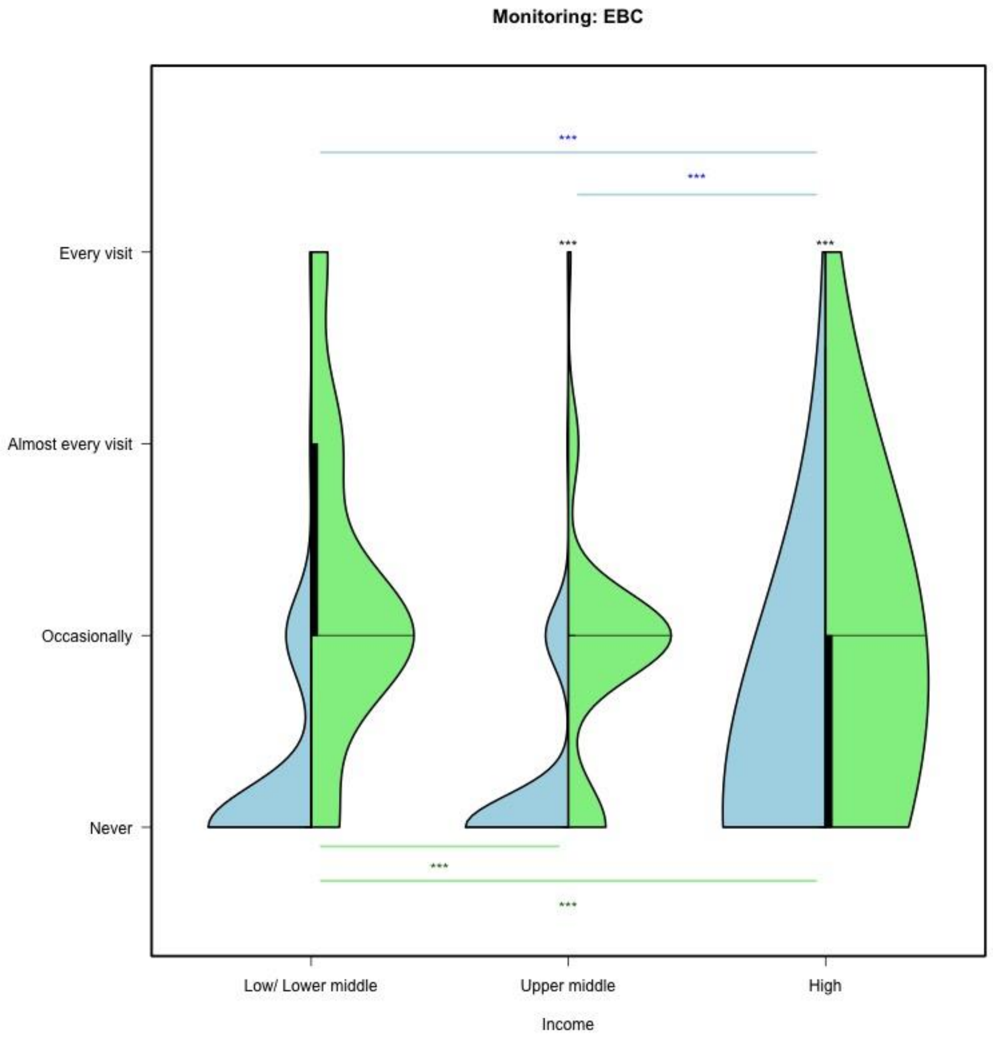

© 2023 Papadopoulos NG et al. *JAMA Network Open.*

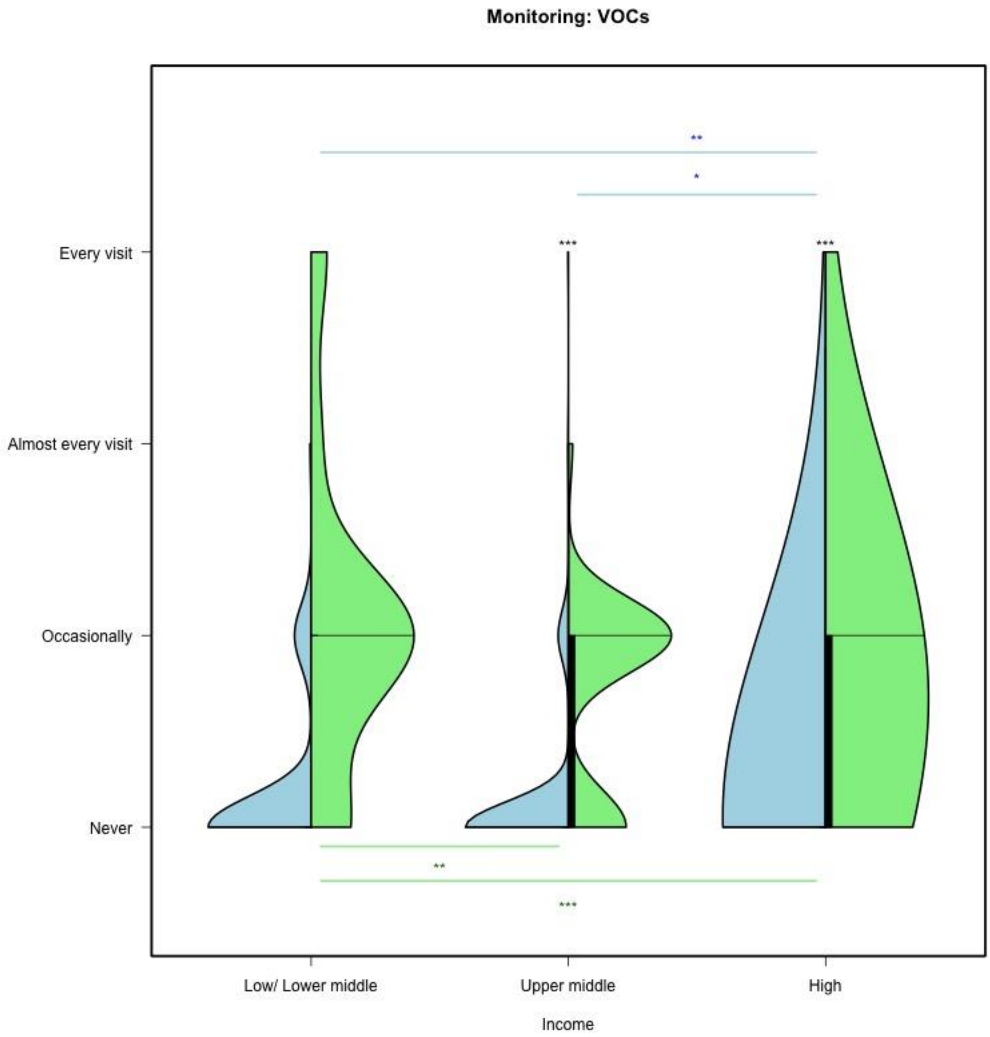

© 2023 Papadopoulos NG et al. *JAMA Network Open.*

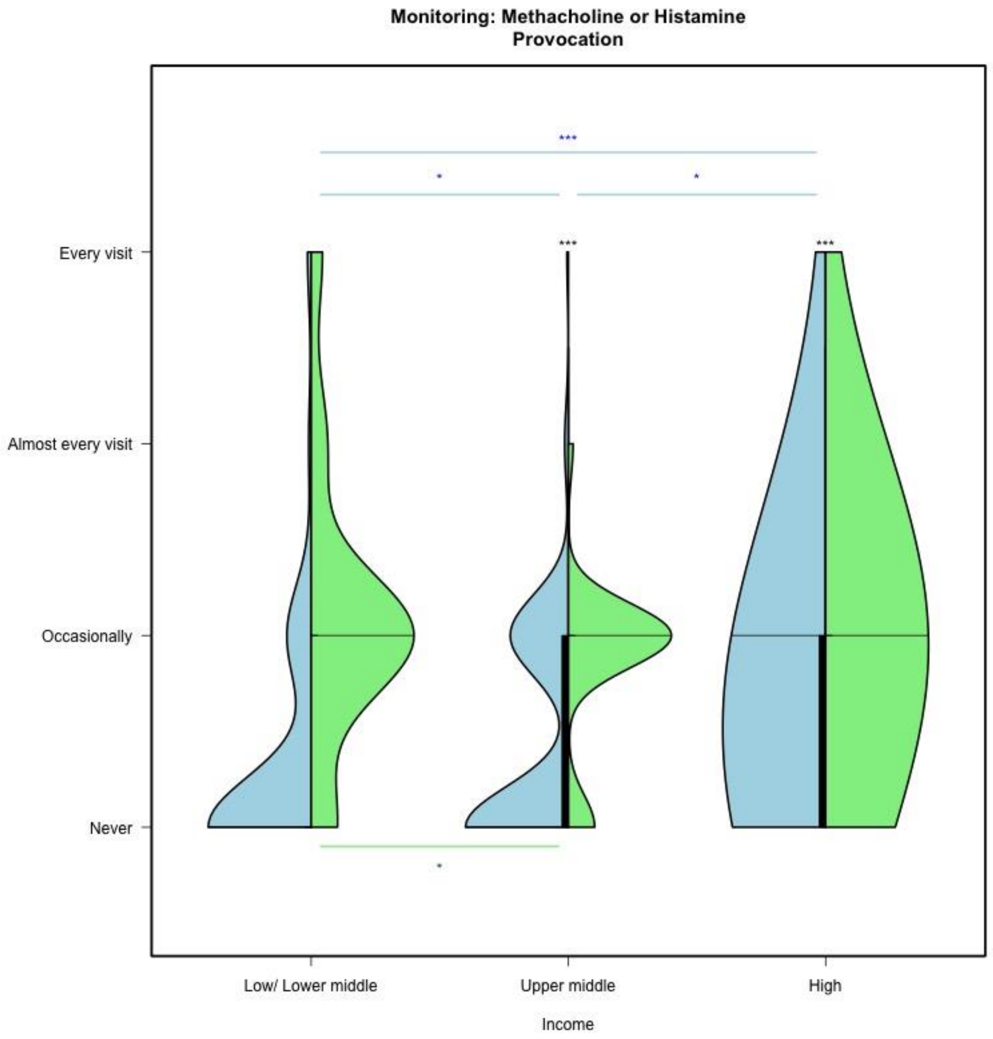

© 2023 Papadopoulos NG et al. *JAMA Network Open.*

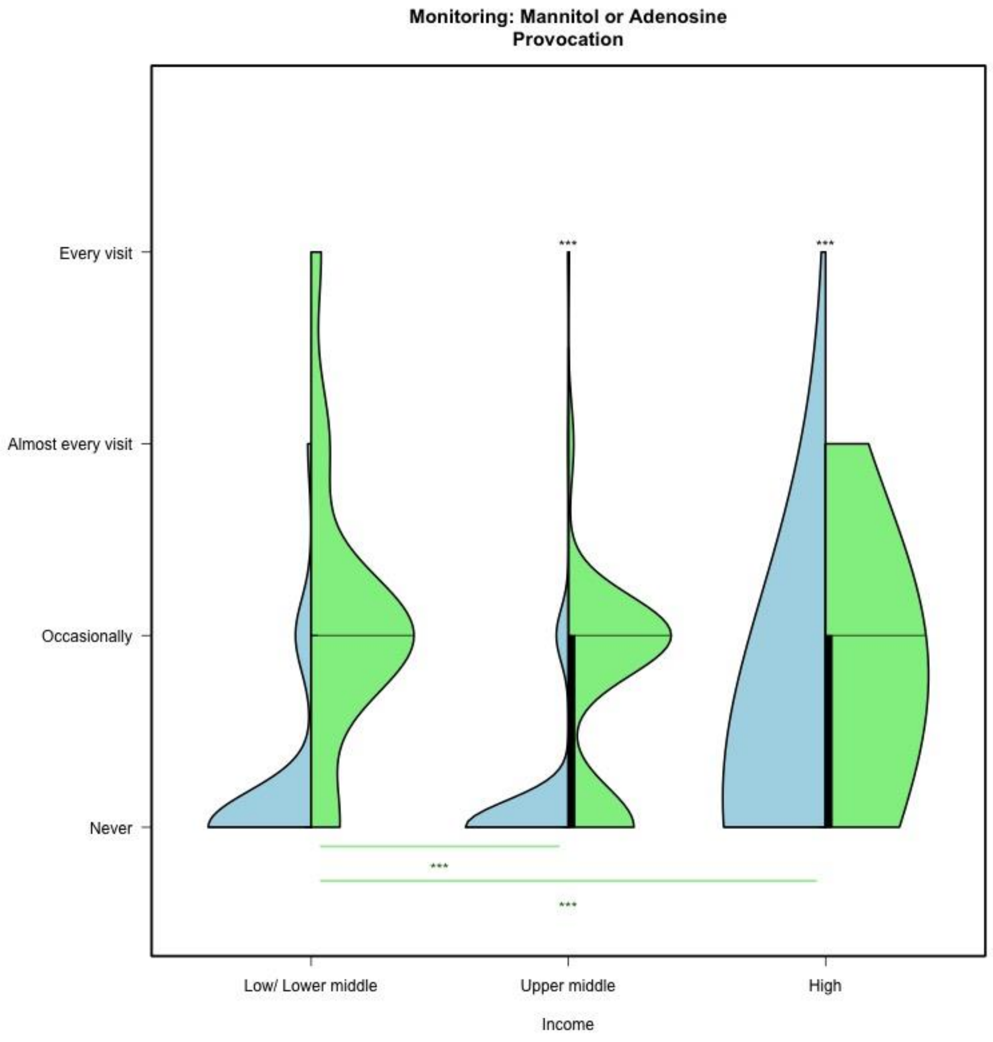

© 2023 Papadopoulos NG et al. *JAMA Network Open.*

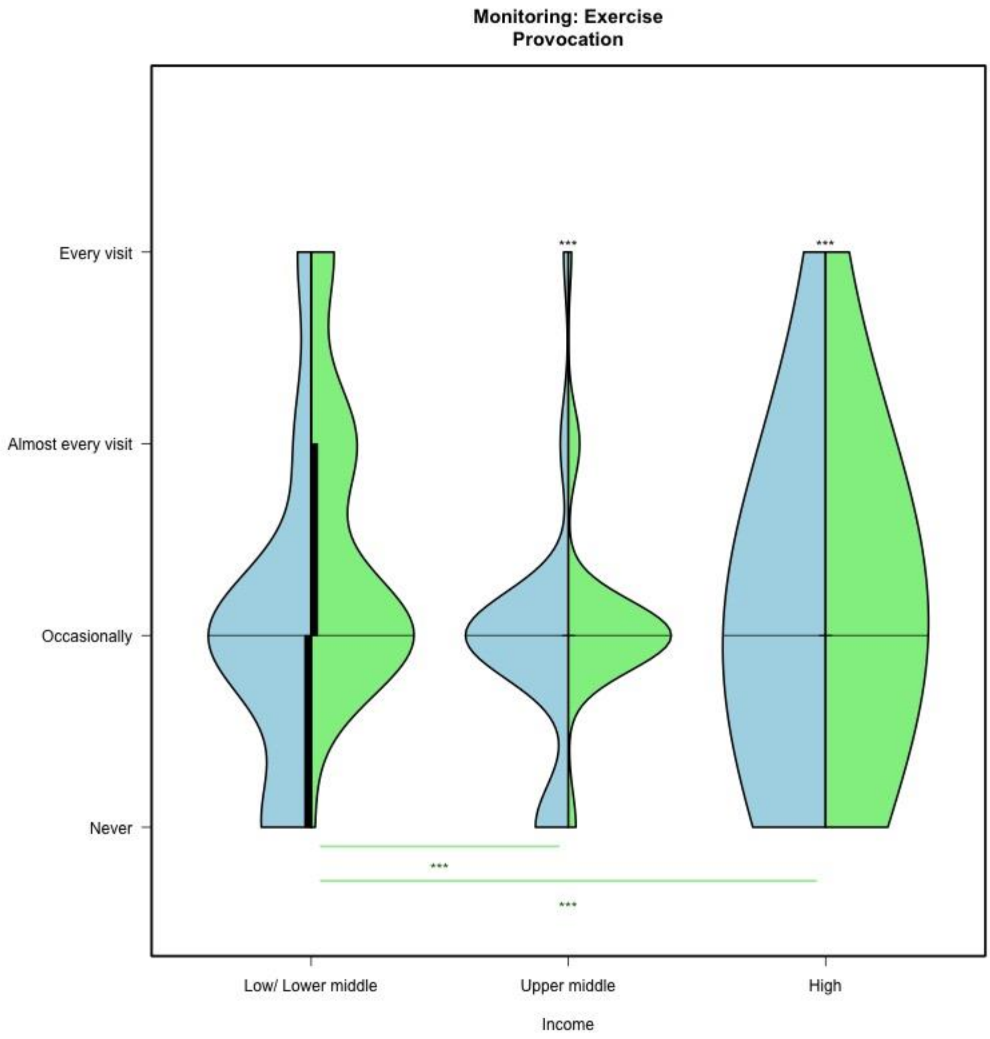

© 2023 Papadopoulos NG et al. *JAMA Network Open.*

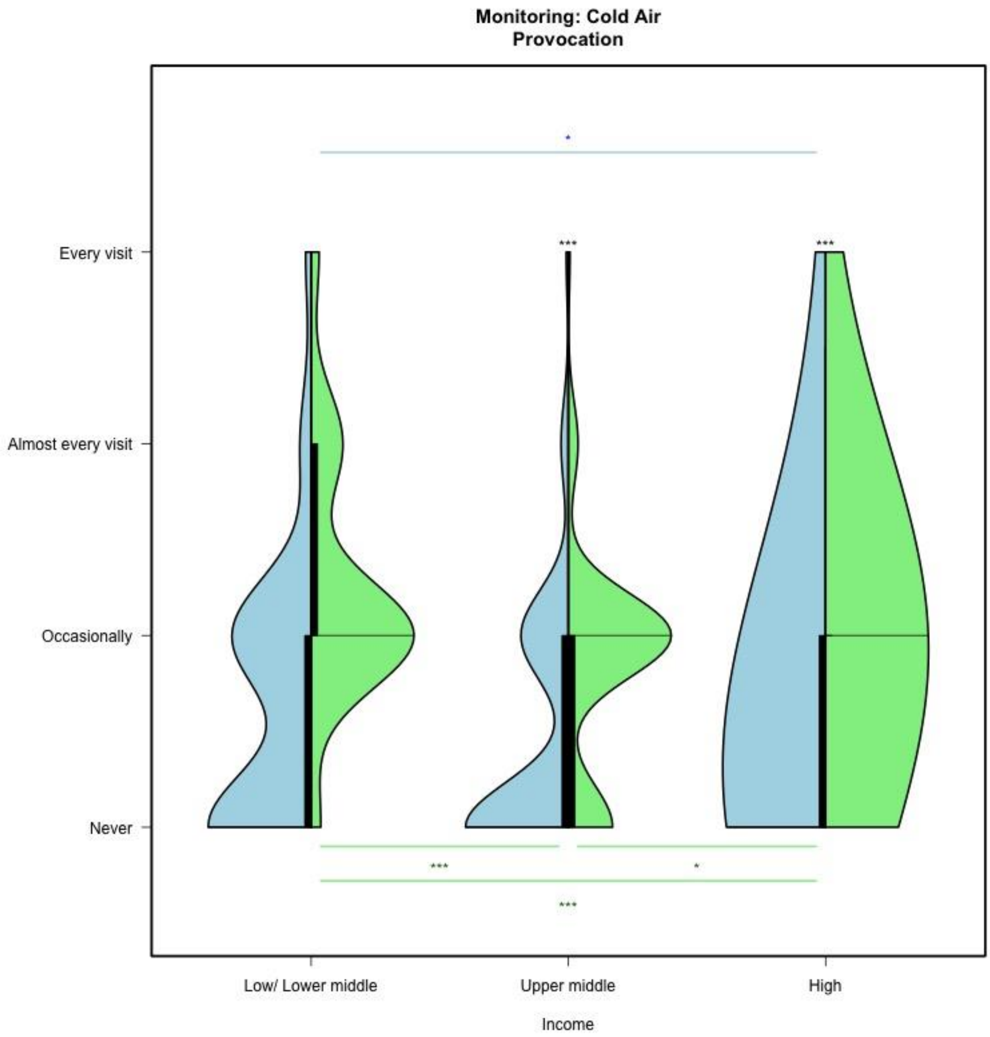

© 2023 Papadopoulos NG et al. *JAMA Network Open.*

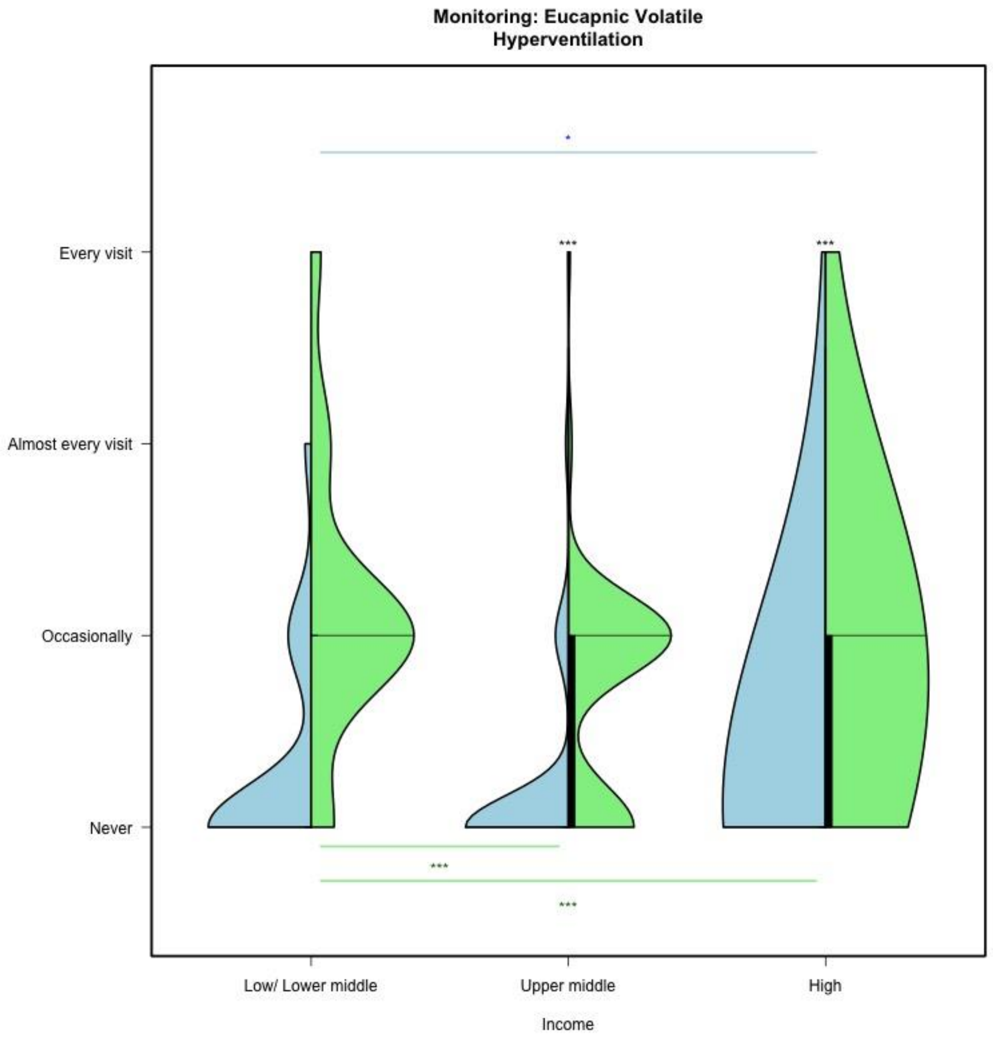

© 2023 Papadopoulos NG et al. *JAMA Network Open.*

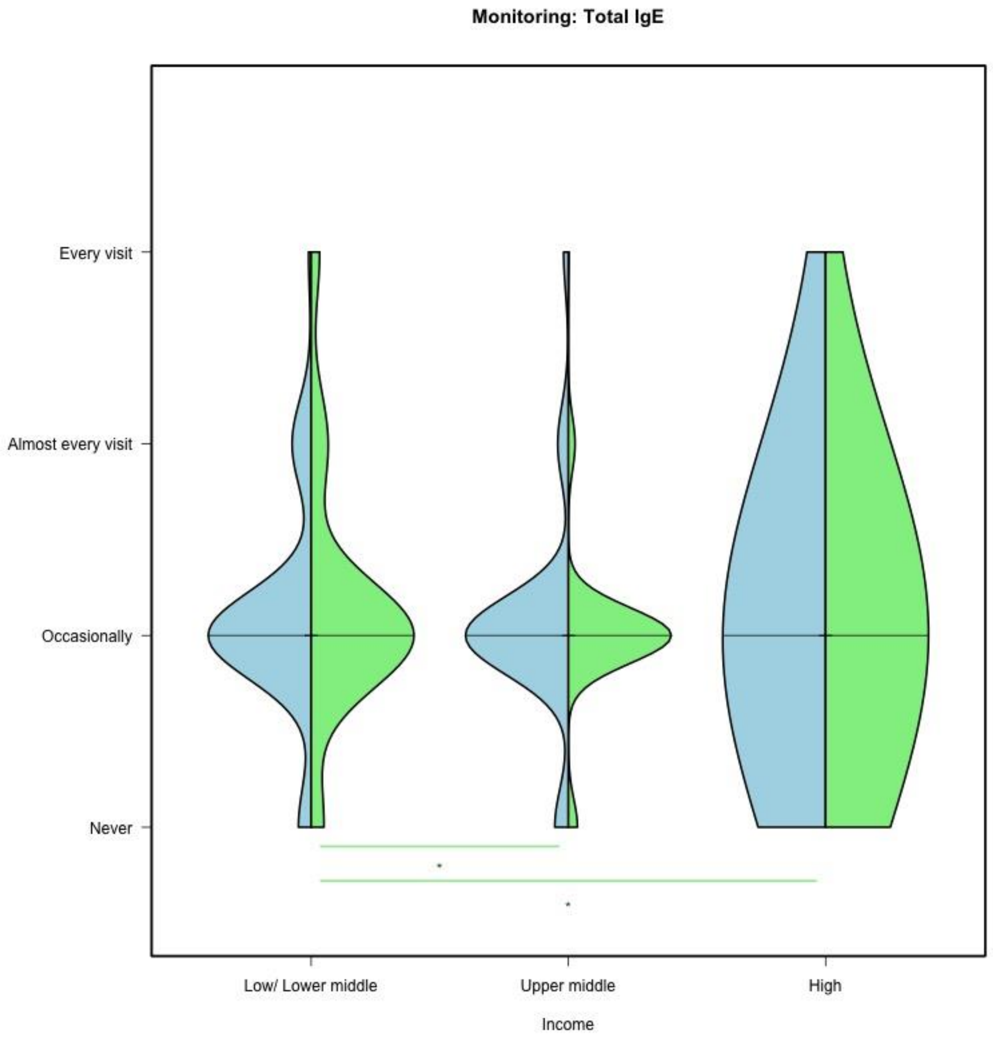

© 2023 Papadopoulos NG et al. *JAMA Network Open.*

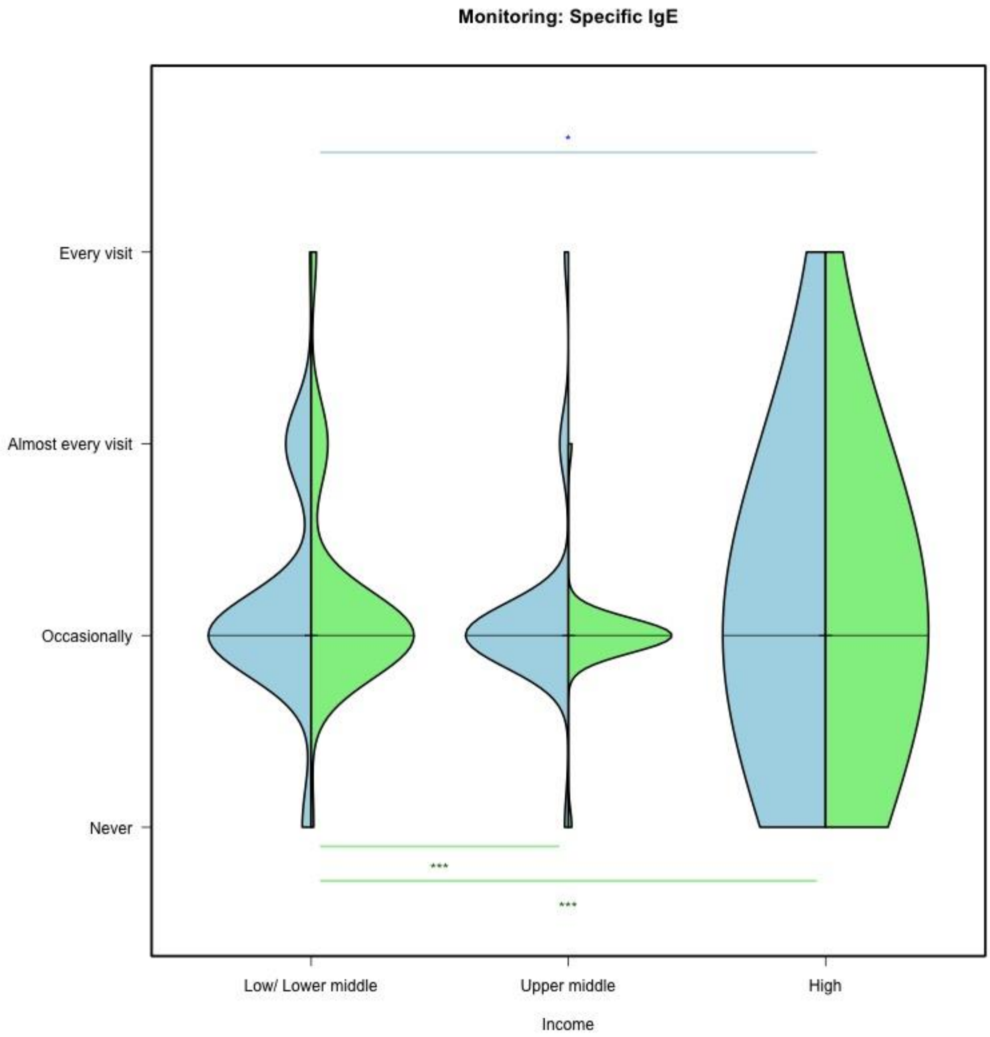

© 2023 Papadopoulos NG et al. *JAMA Network Open.*

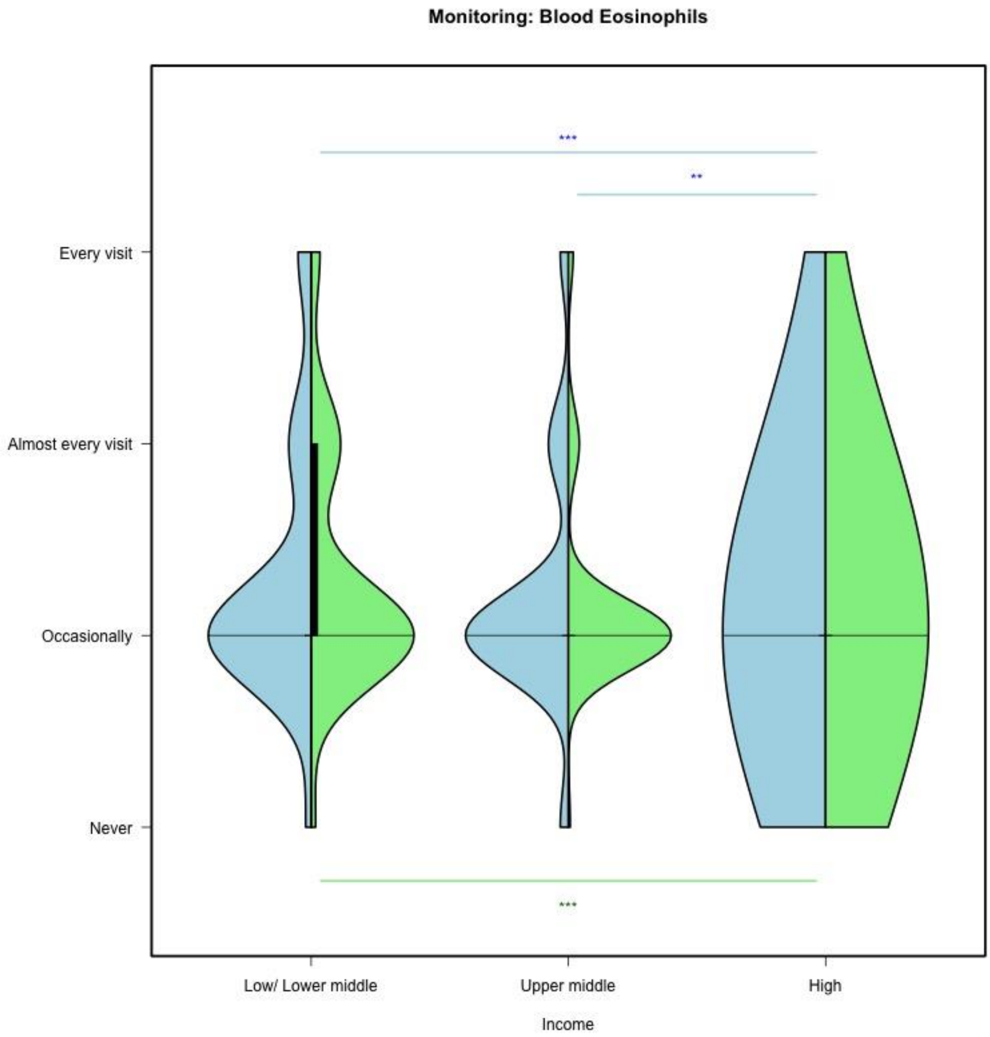

© 2023 Papadopoulos NG et al. *JAMA Network Open.*

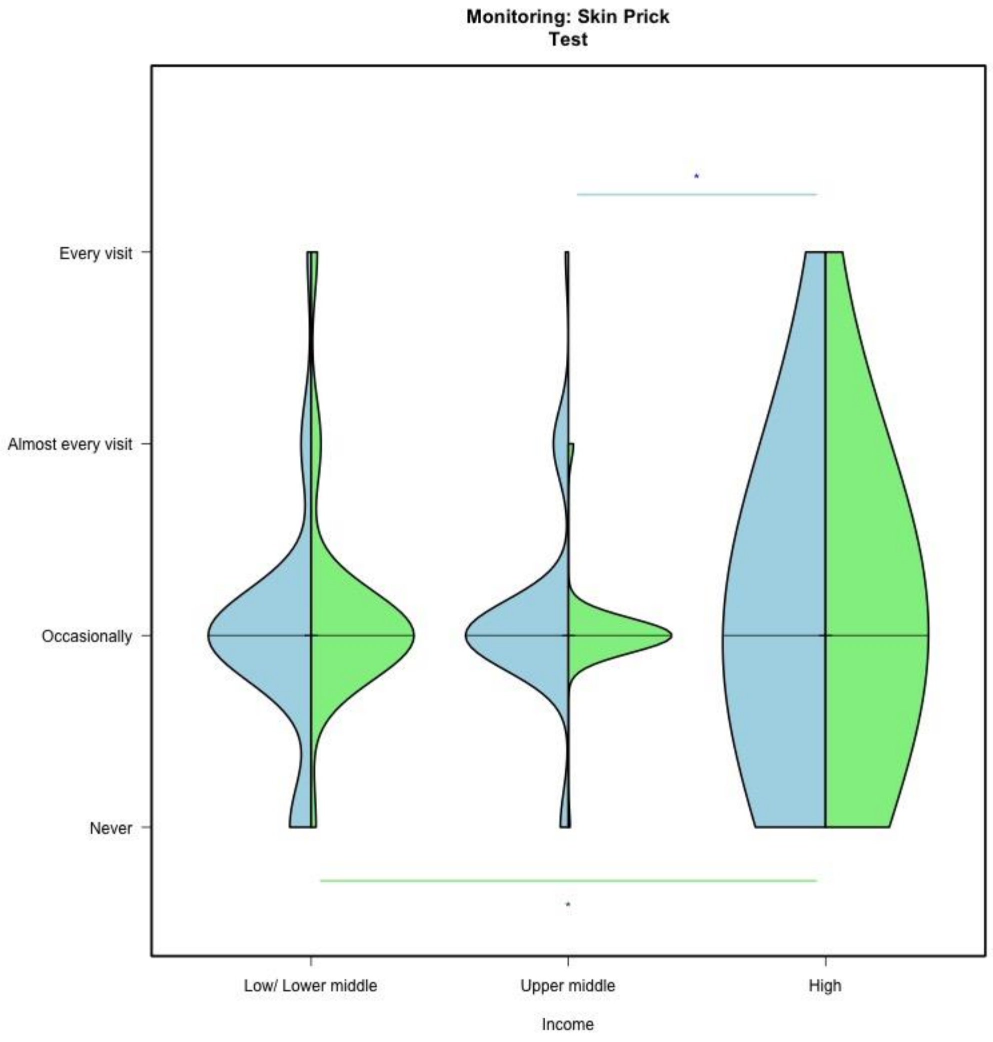

© 2023 Papadopoulos NG et al. *JAMA Network Open.*

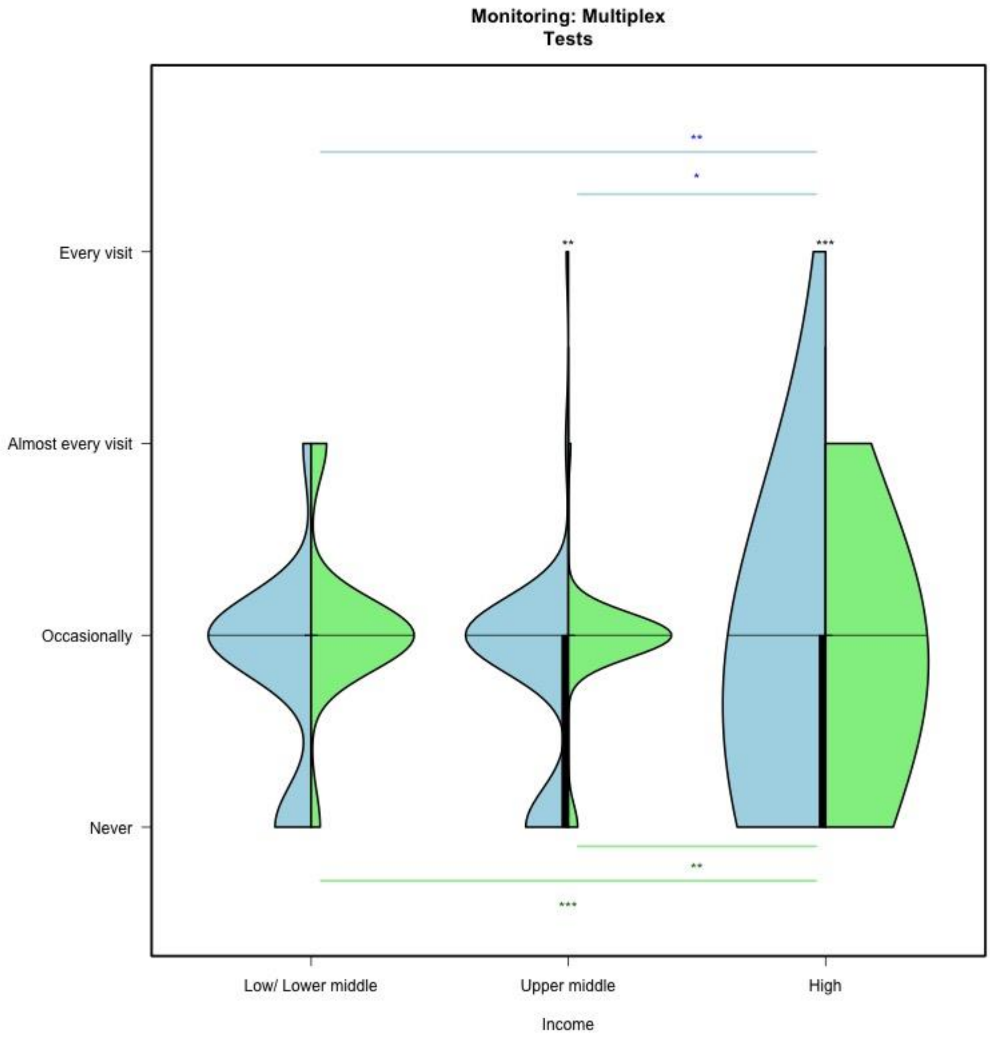

© 2023 Papadopoulos NG et al. *JAMA Network Open.*

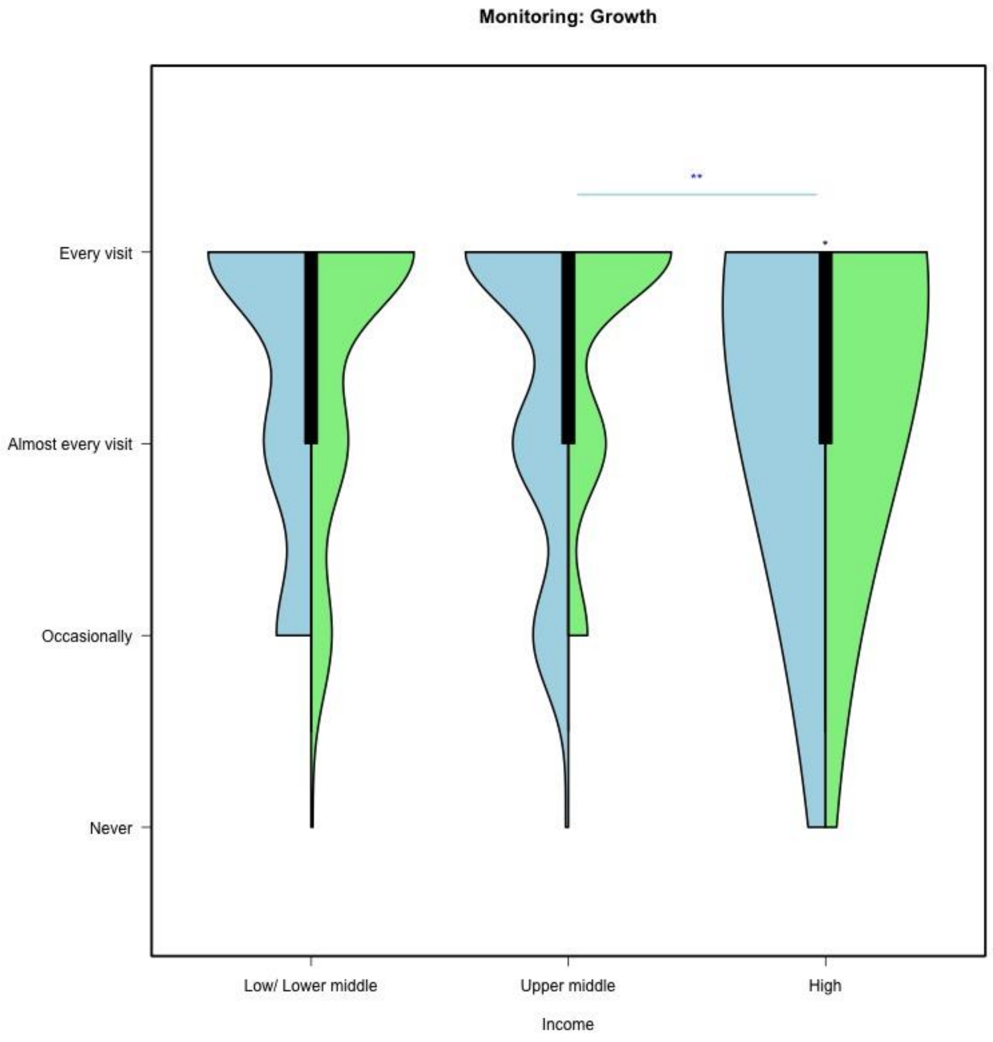

© 2023 Papadopoulos NG et al. *JAMA Network Open.*

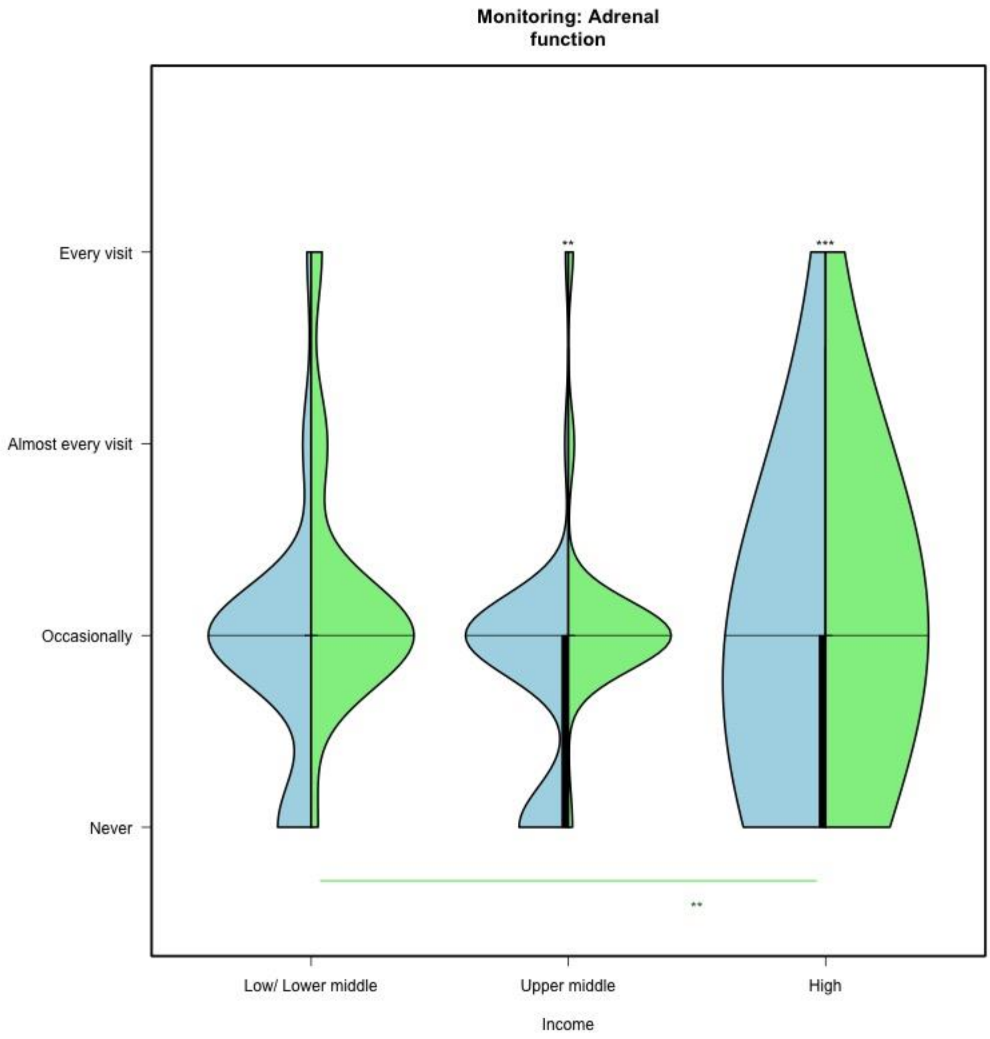

© 2023 Papadopoulos NG et al. *JAMA Network Open.*

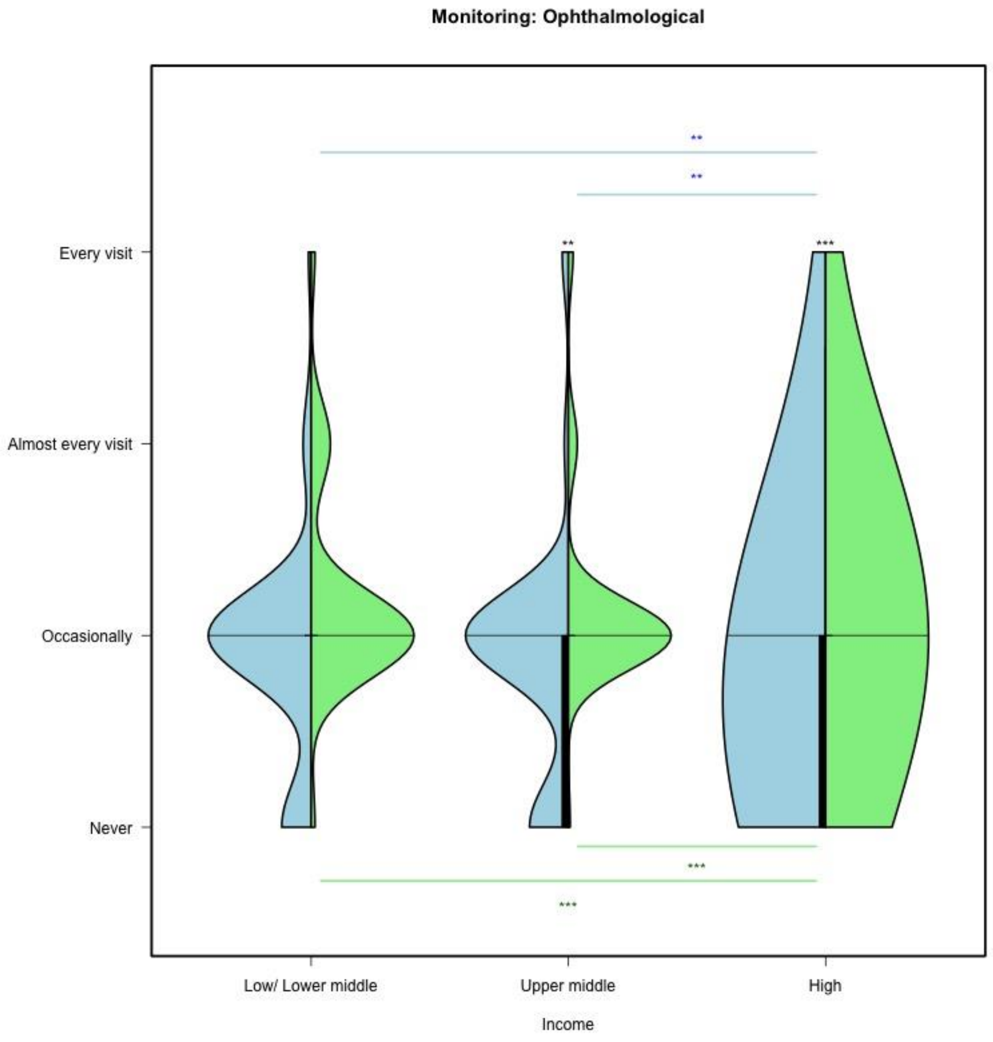

© 2023 Papadopoulos NG et al. *JAMA Network Open.*

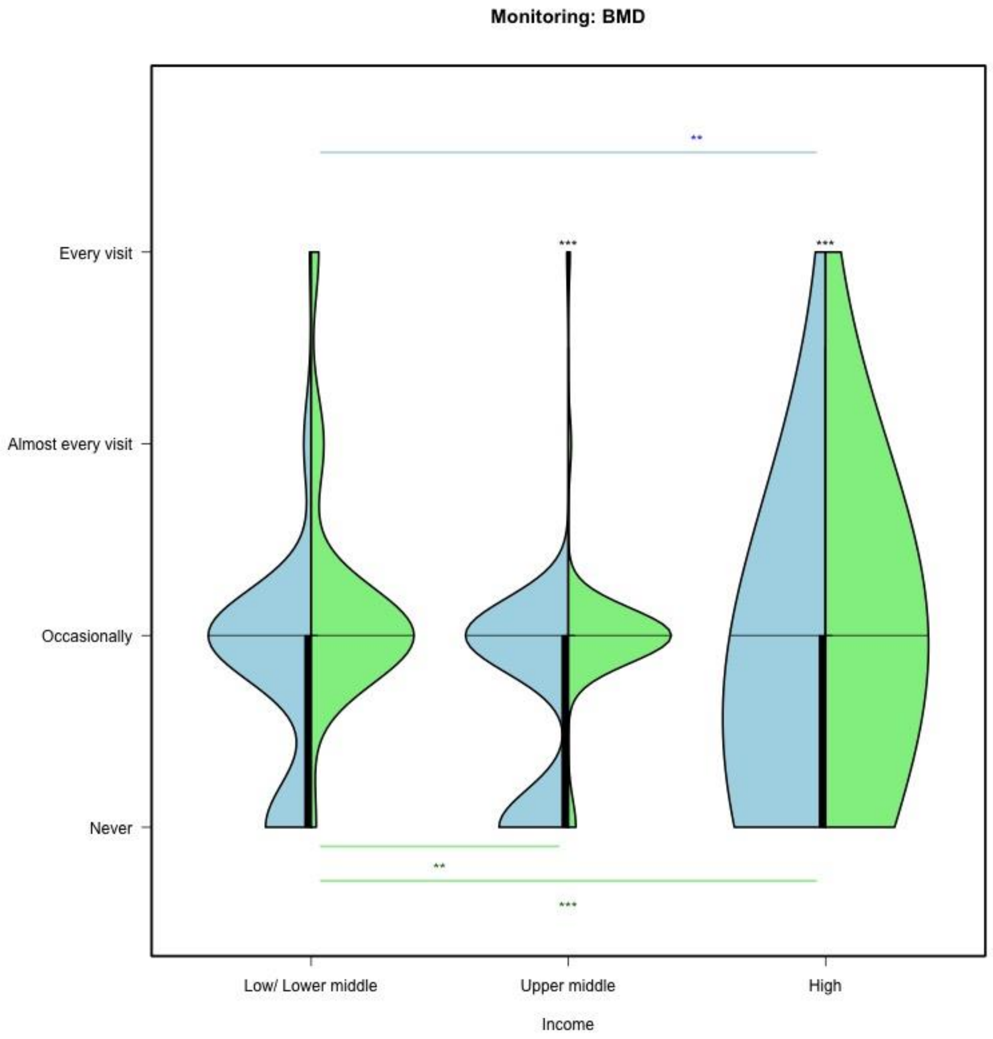

© 2023 Papadopoulos NG et al. *JAMA Network Open.*

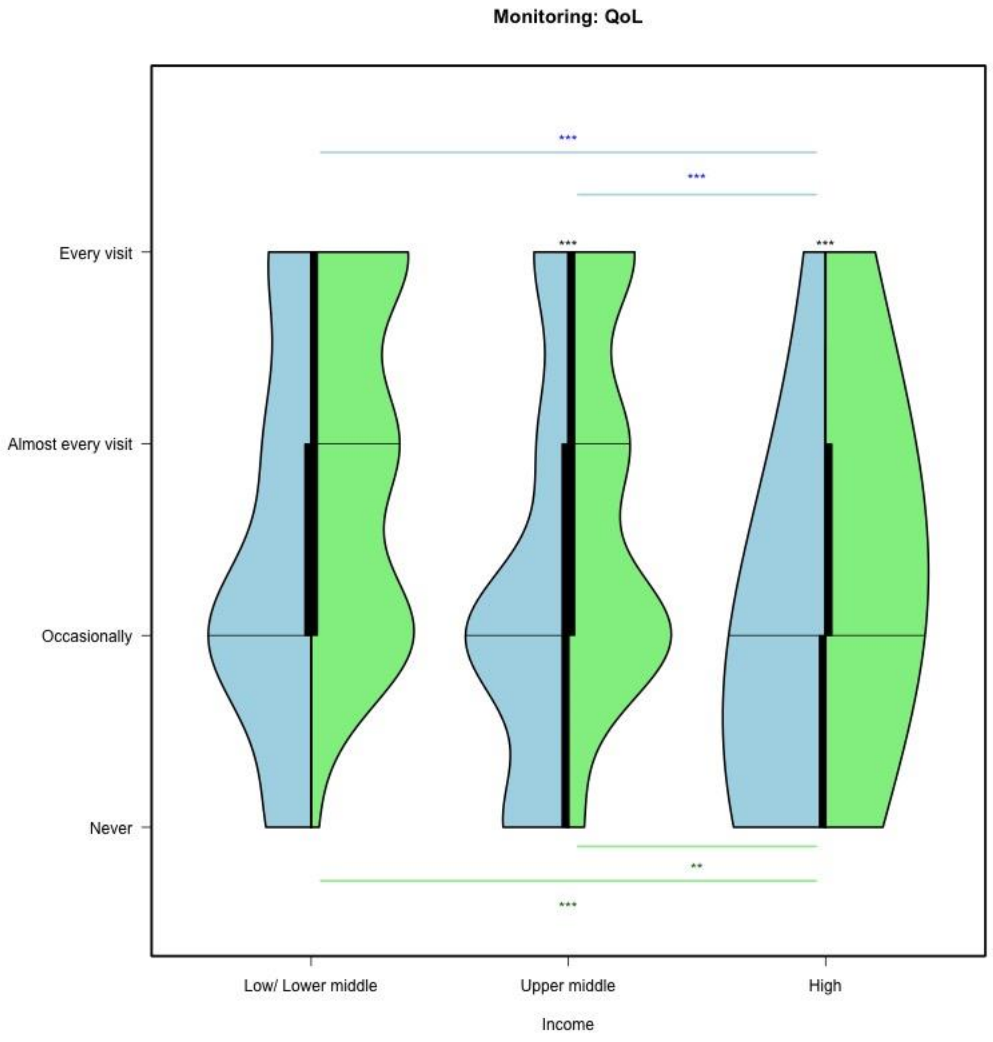

© 2023 Papadopoulos NG et al. *JAMA Network Open.*

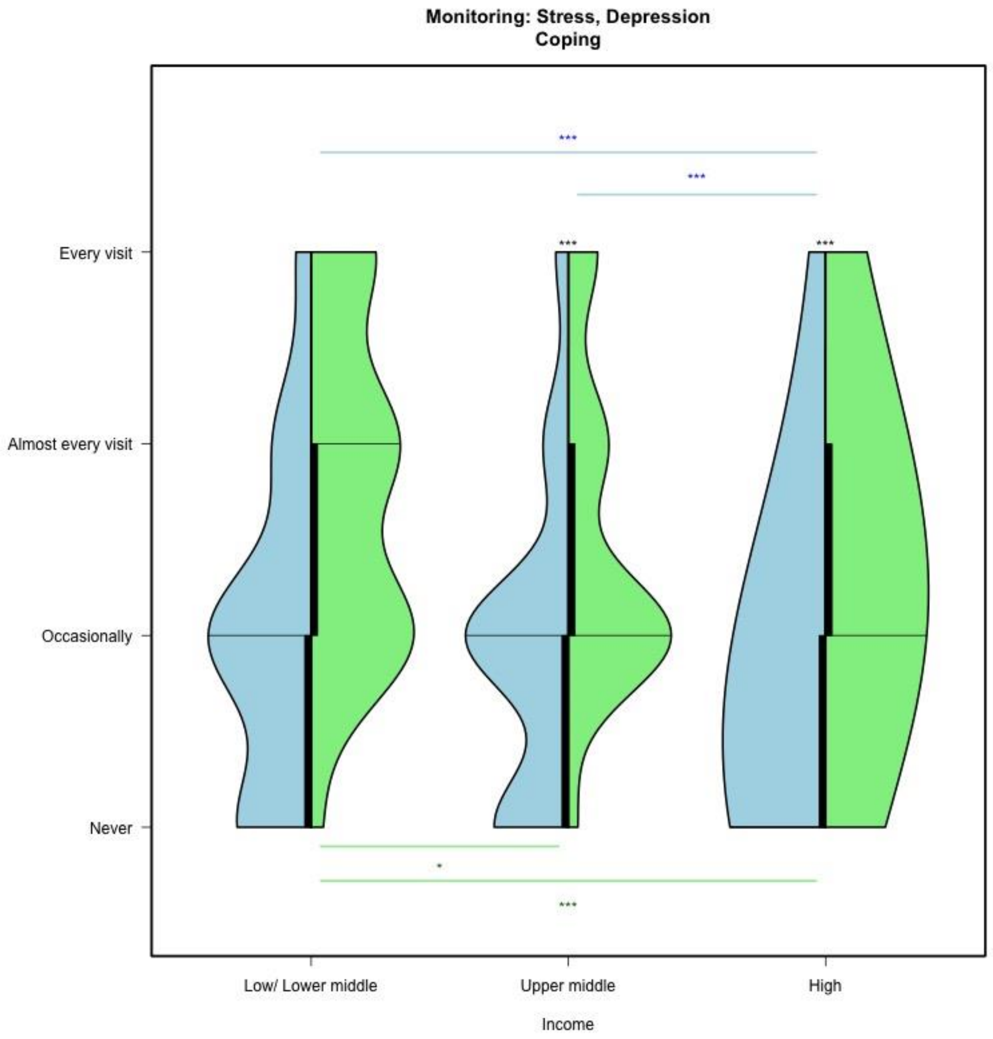

© 2023 Papadopoulos NG et al. *JAMA Network Open.*

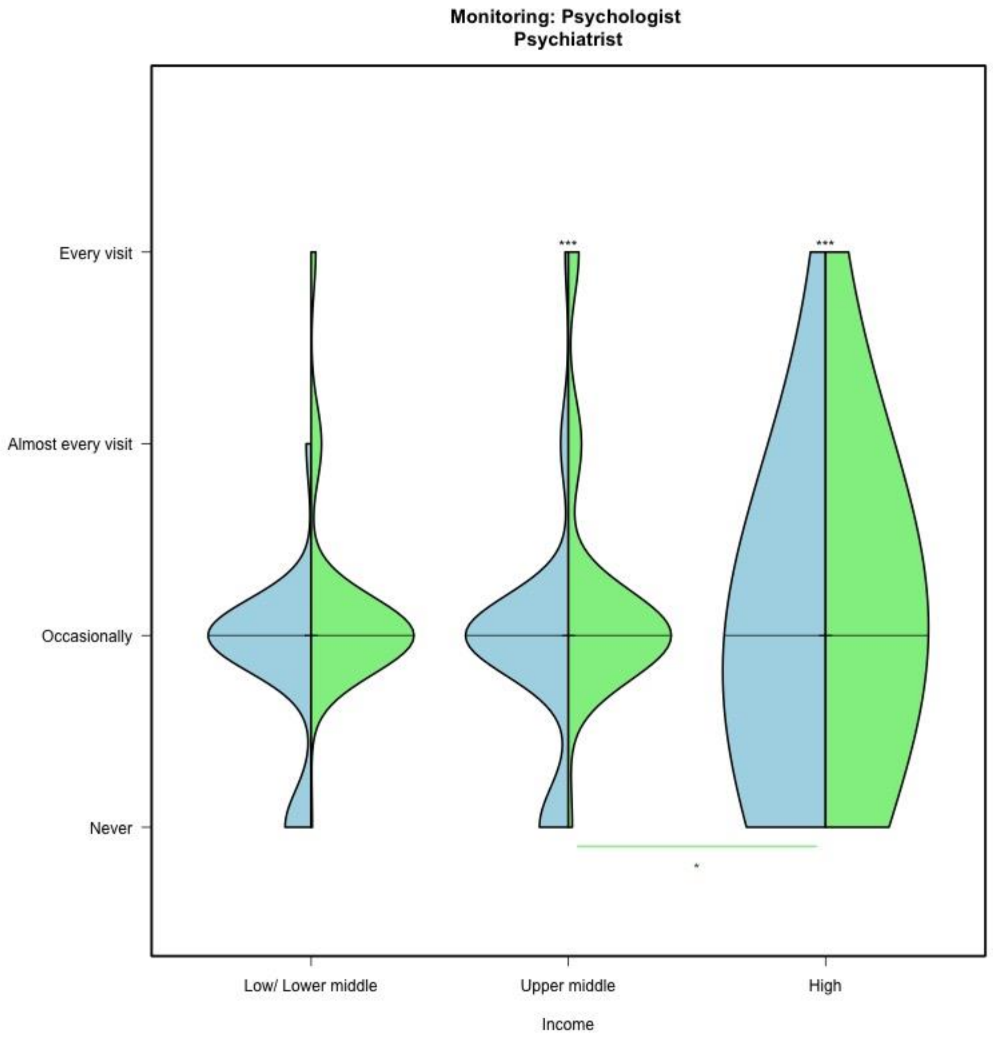

© 2023 Papadopoulos NG et al. *JAMA Network Open.*

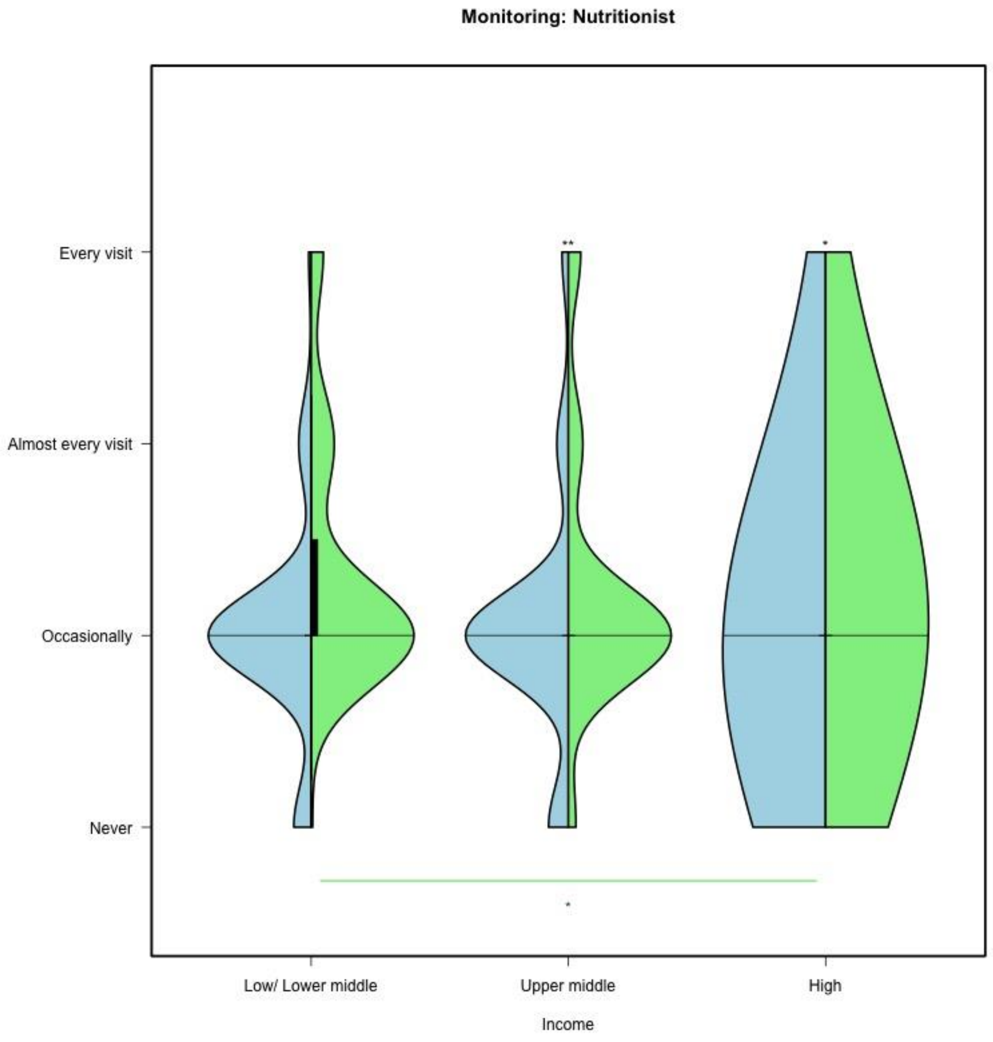

© 2023 Papadopoulos NG et al. *JAMA Network Open.*

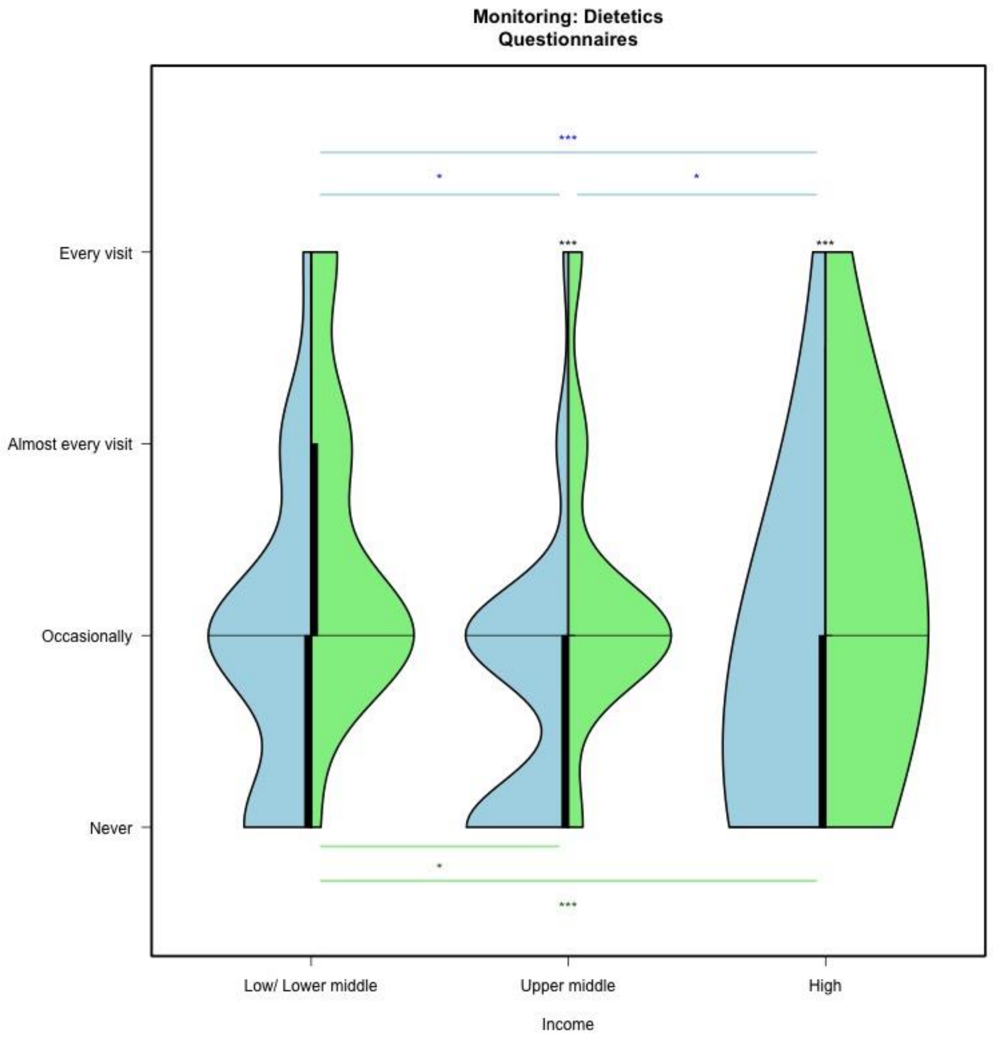

© 2023 Papadopoulos NG et al. *JAMA Network Open.*

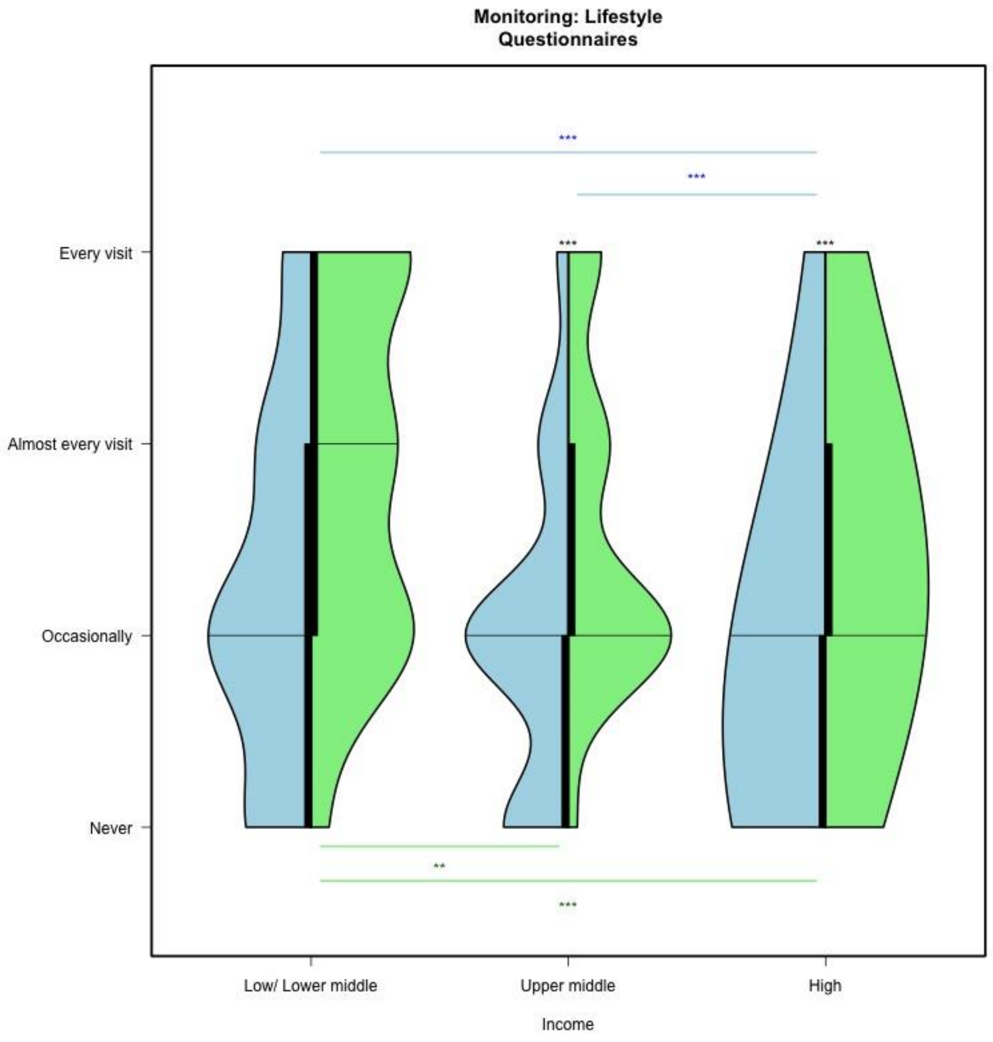

© 2023 Papadopoulos NG et al. *JAMA Network Open.*

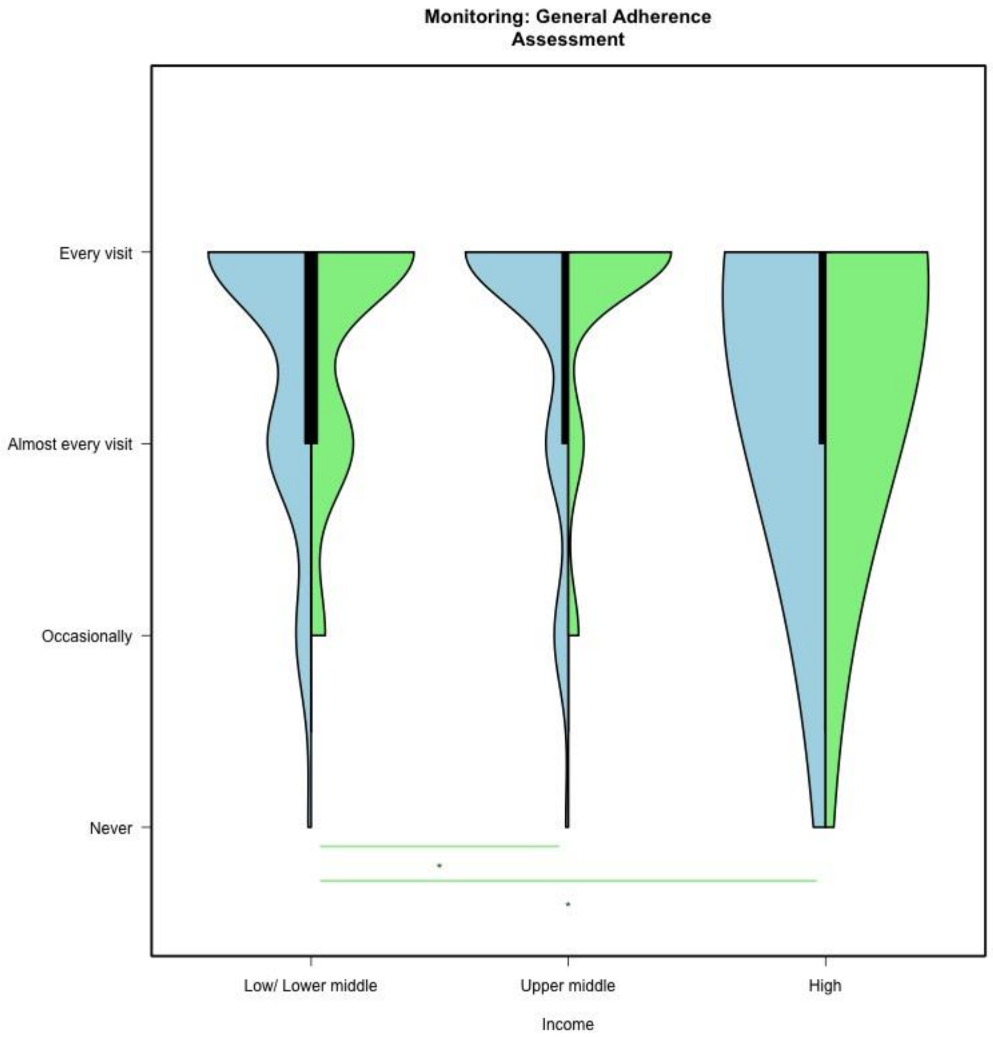

© 2023 Papadopoulos NG et al. *JAMA Network Open.*

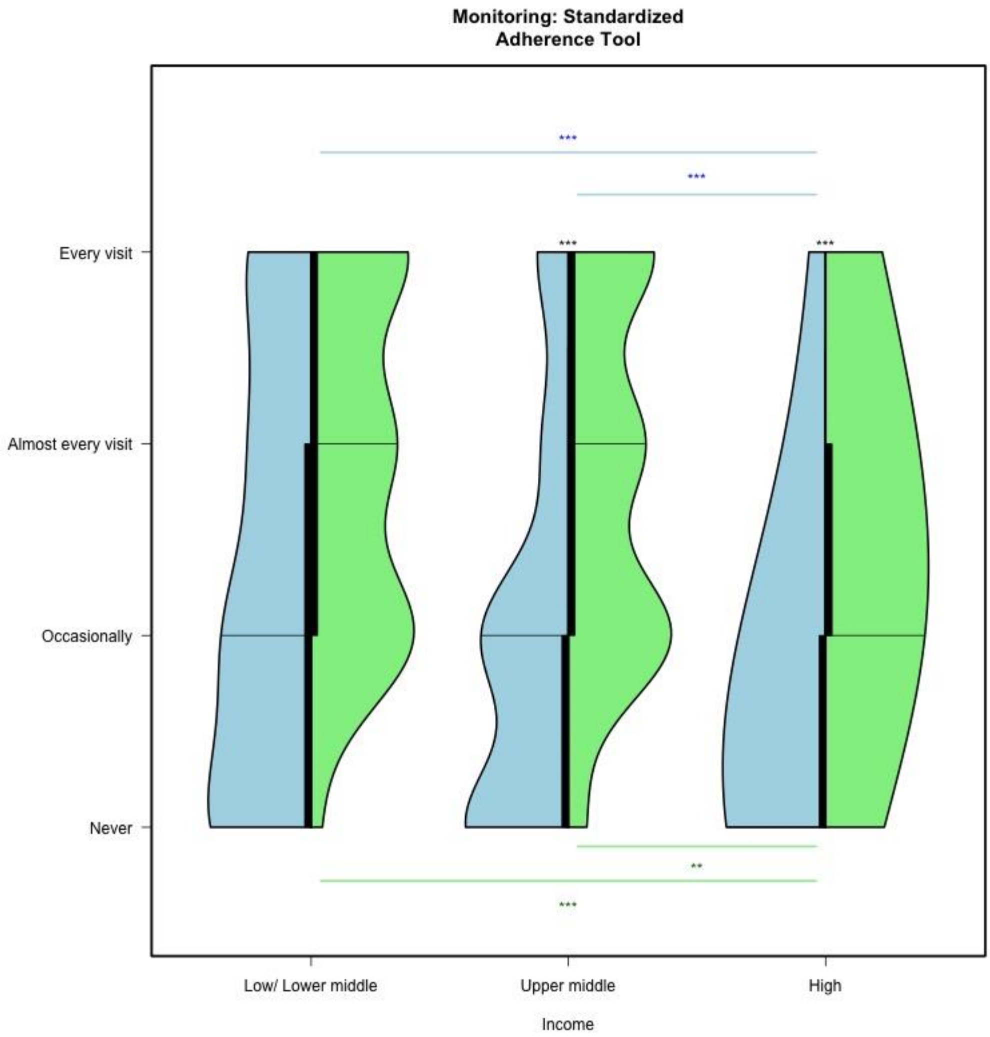

© 2023 Papadopoulos NG et al. *JAMA Network Open.*

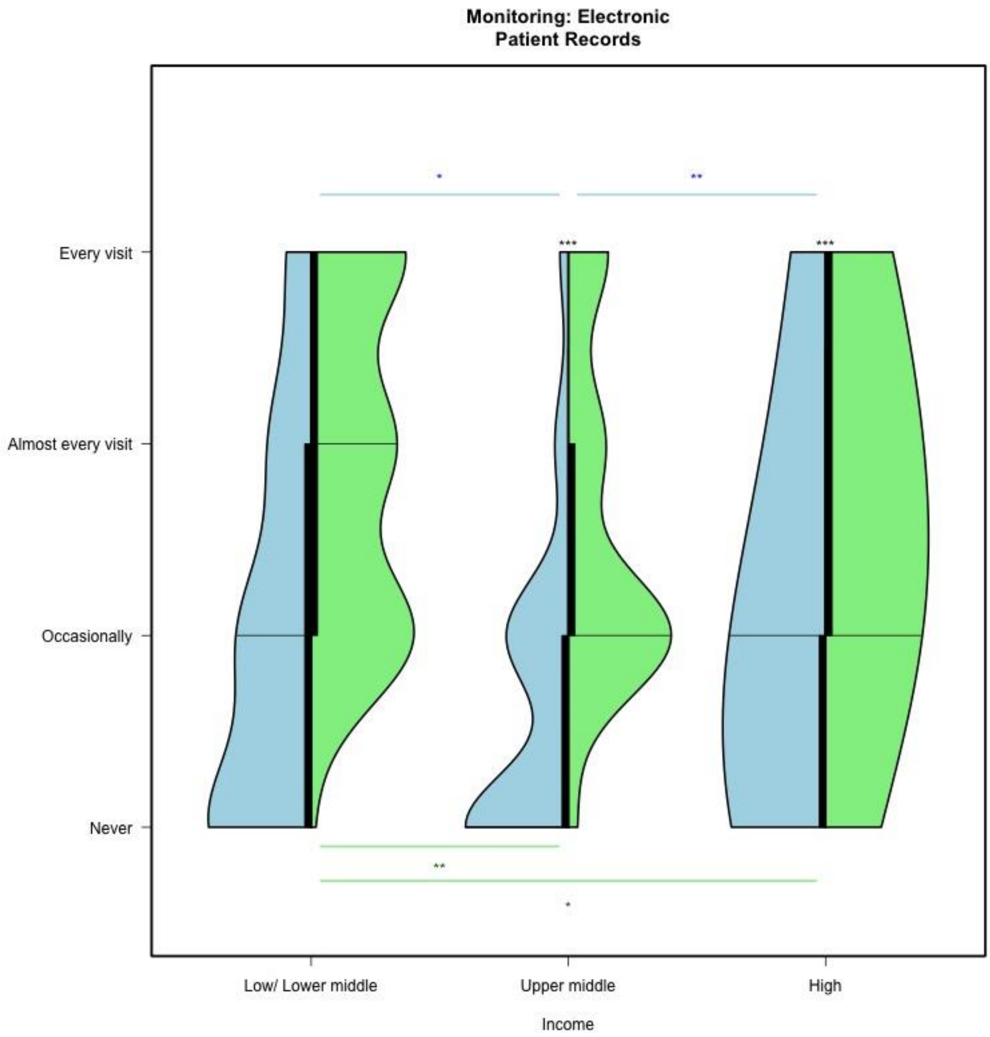

© 2023 Papadopoulos NG et al. *JAMA Network Open.*

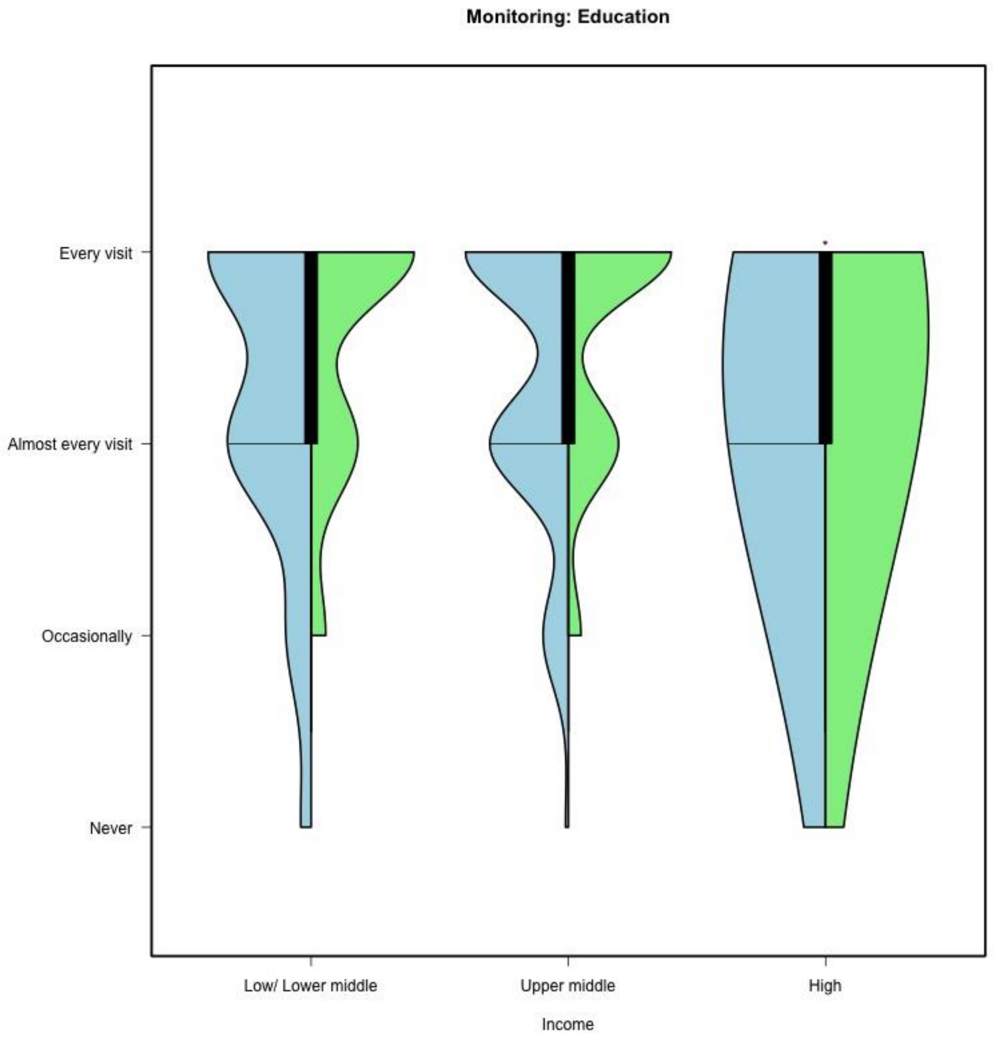

© 2023 Papadopoulos NG et al. *JAMA Network Open.*

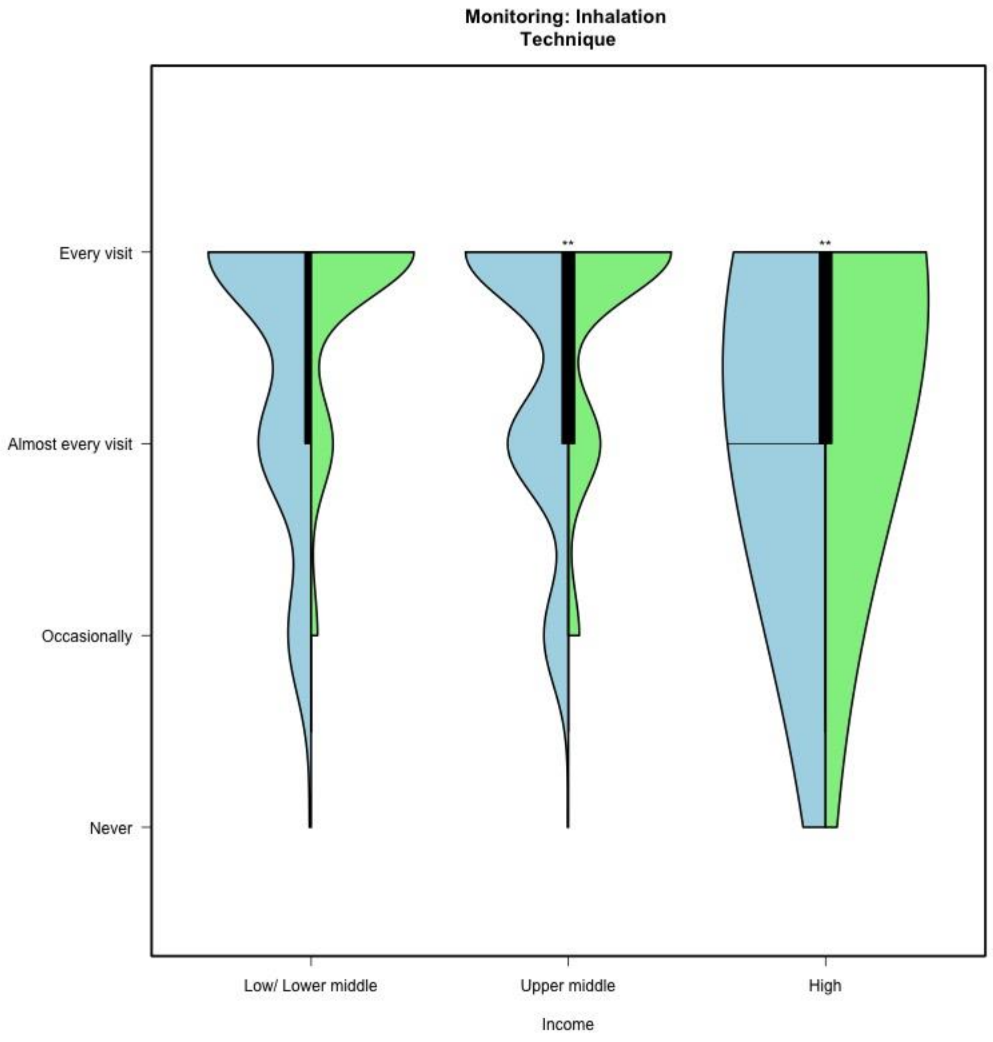

© 2023 Papadopoulos NG et al. *JAMA Network Open.*

eFigure 15. Actual (Light Blue, Left Side of the Violin Plots) and Perceived Optimal (Green, Right Side of the Violin Plots) Use of Monitoring Tools During Asthma Monitoring Visits, Stratified by Care Setting

*: p<0.05; **: p<0.01; ***: p<0.001

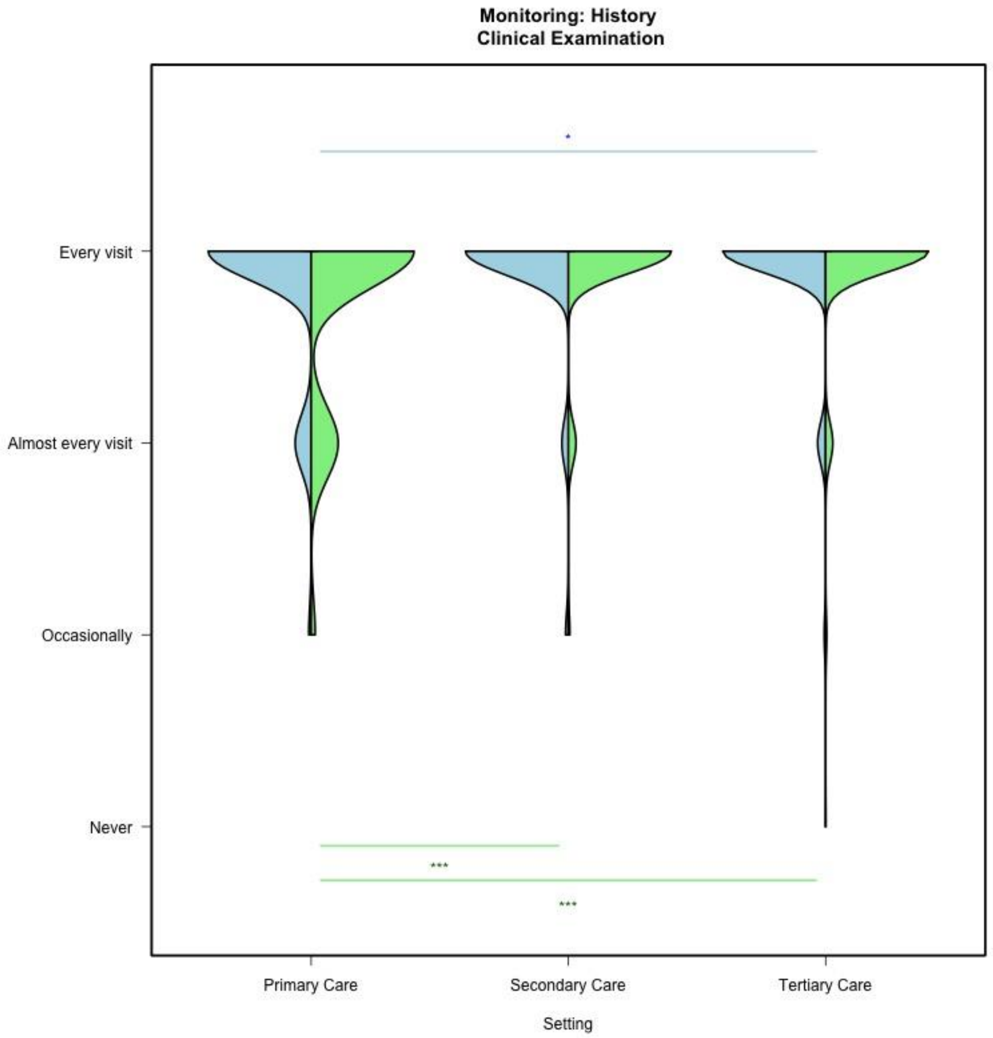

© 2023 Papadopoulos NG et al. *JAMA Network Open.*

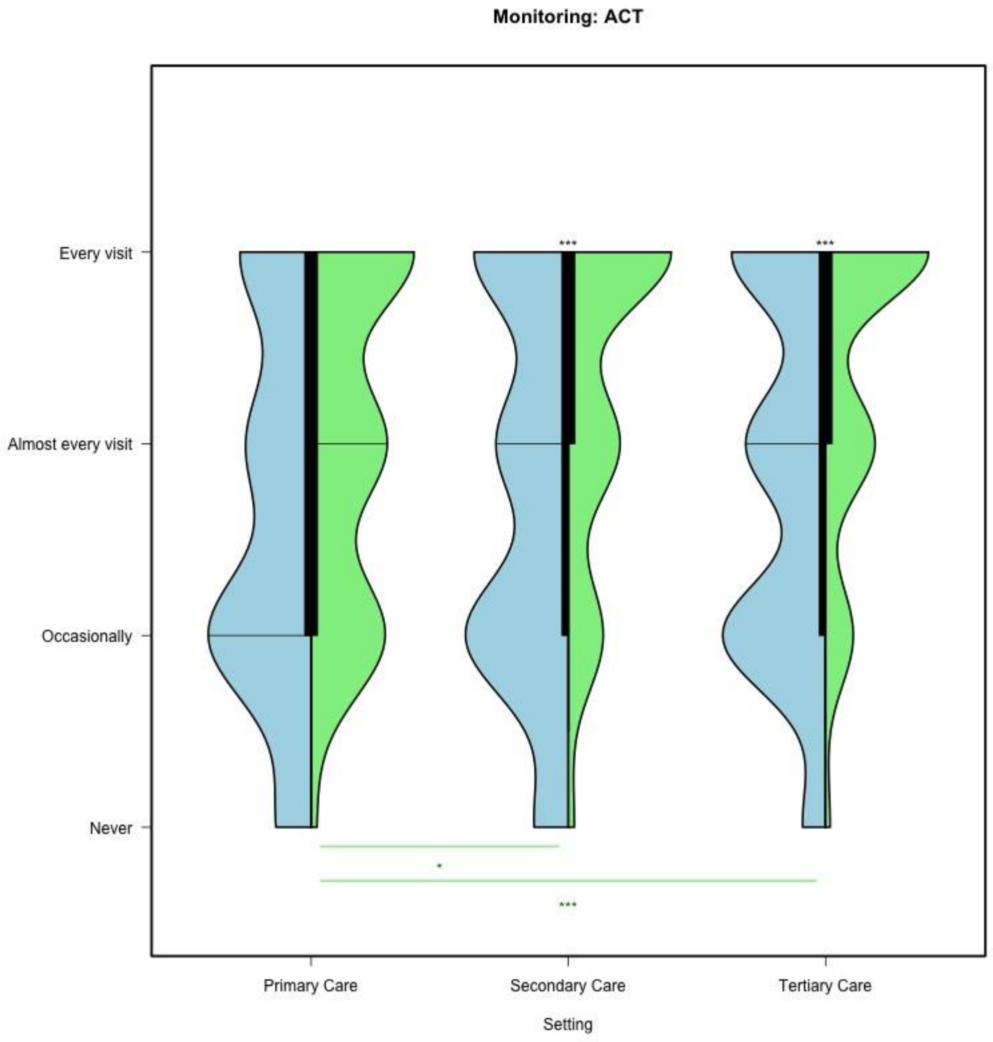

© 2023 Papadopoulos NG et al. *JAMA Network Open.*

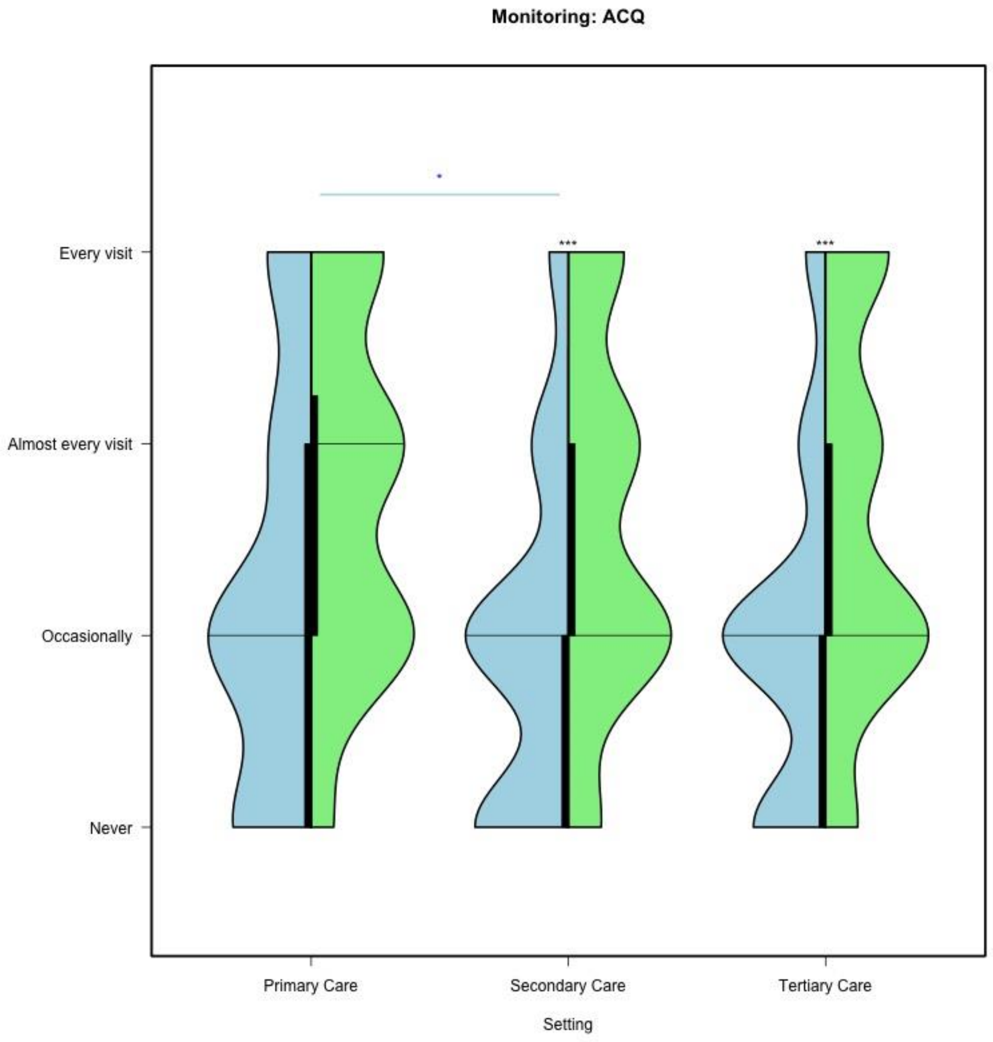

© 2023 Papadopoulos NG et al. *JAMA Network Open.*

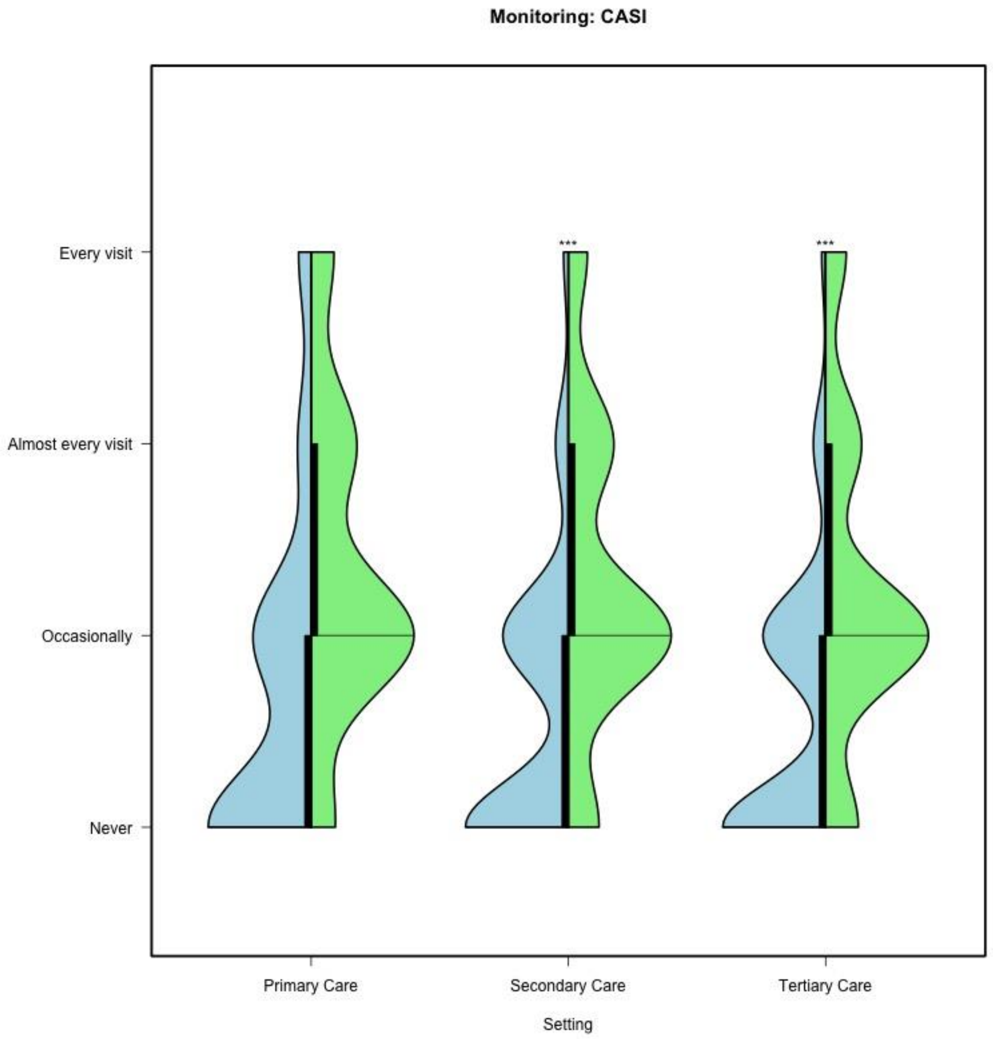

© 2023 Papadopoulos NG et al. *JAMA Network Open.*

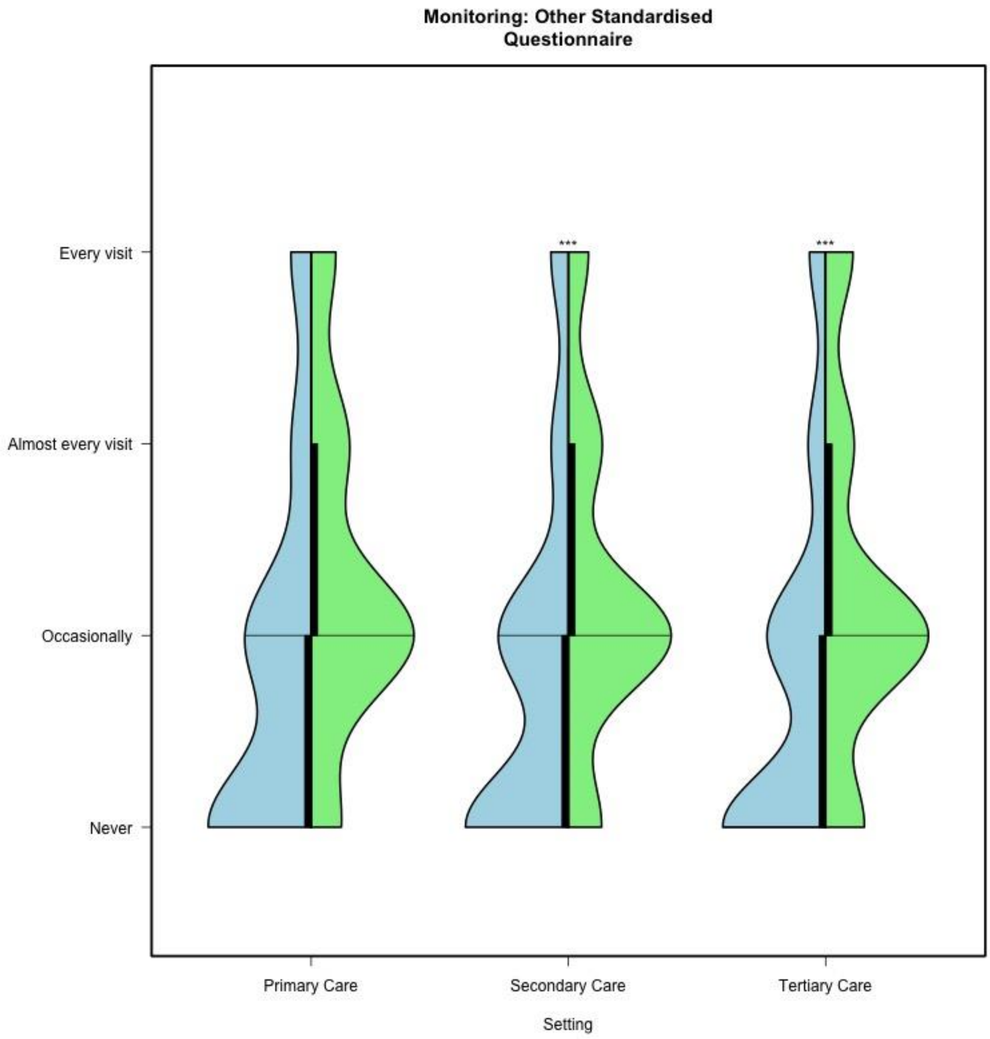

© 2023 Papadopoulos NG et al. *JAMA Network Open.*

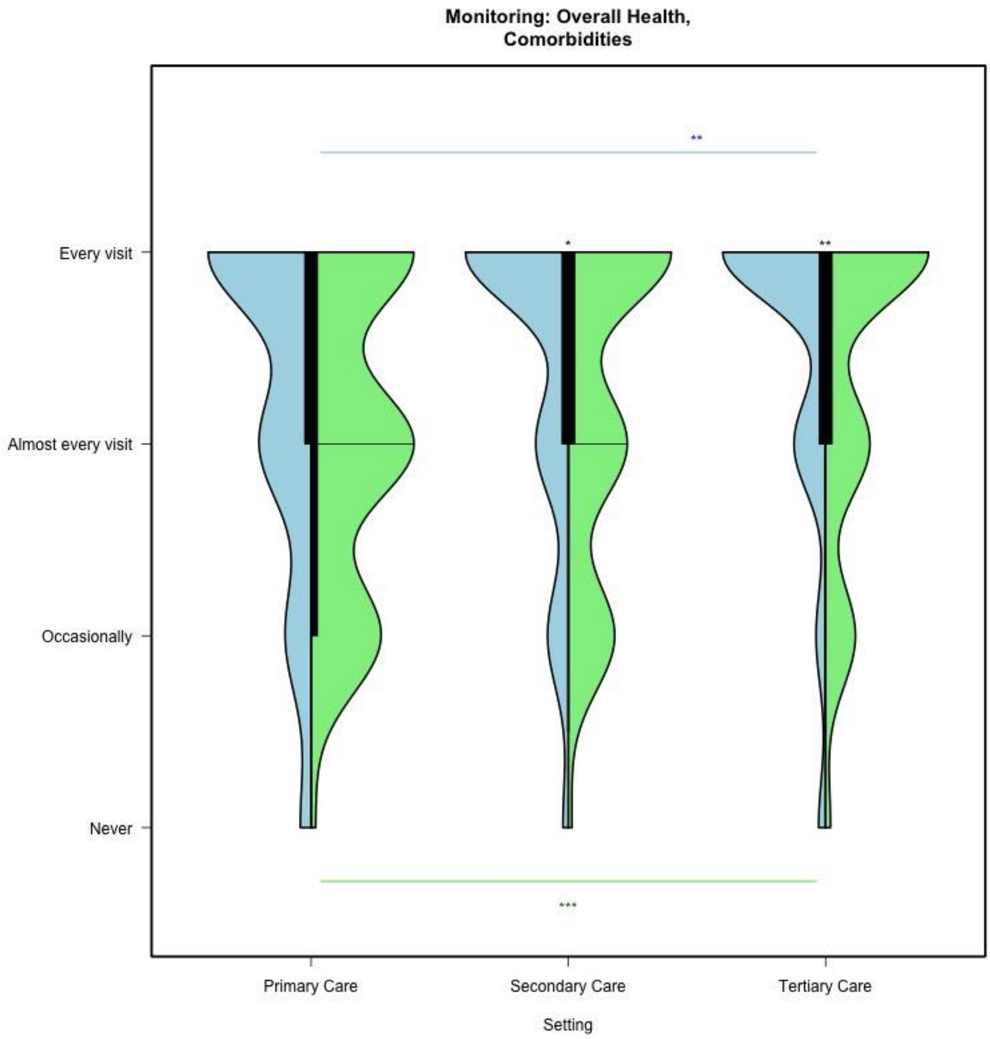

© 2023 Papadopoulos NG et al. *JAMA Network Open.*

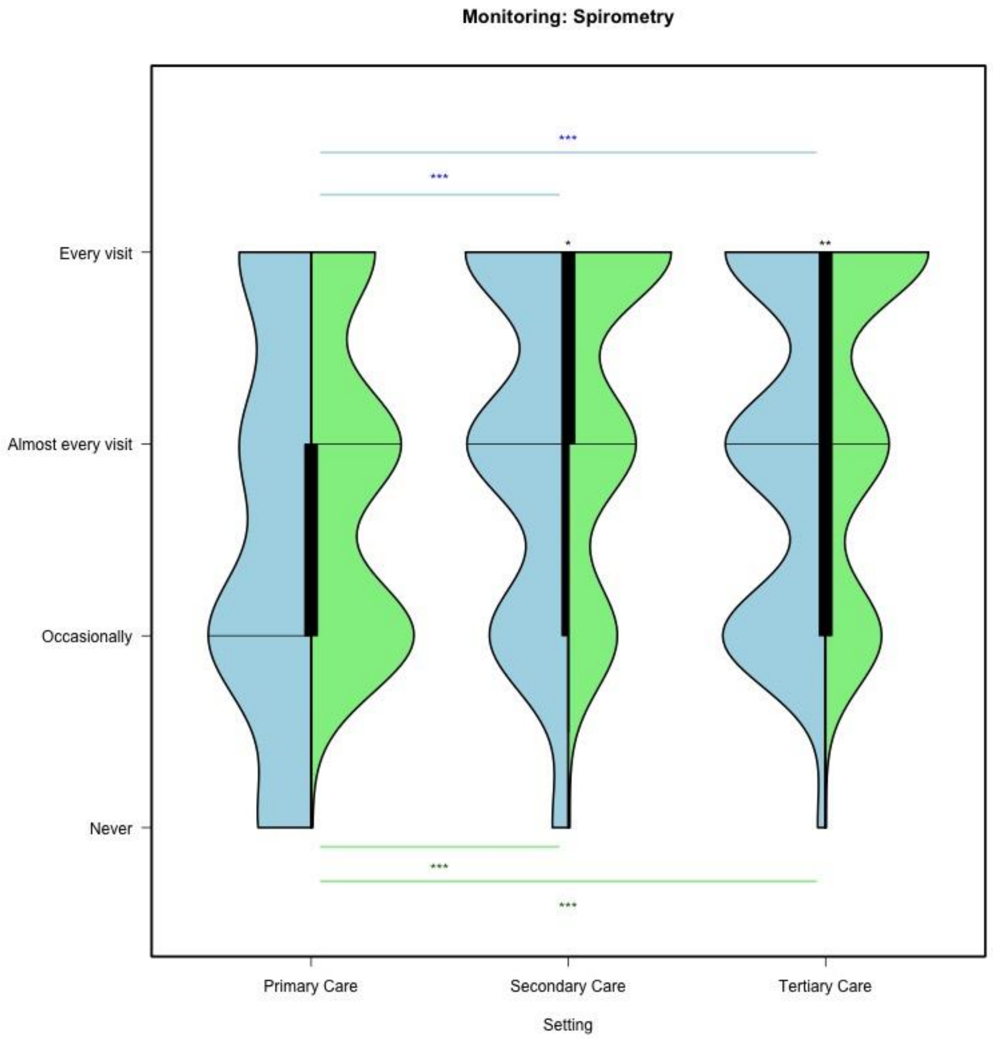

© 2023 Papadopoulos NG et al. *JAMA Network Open.*

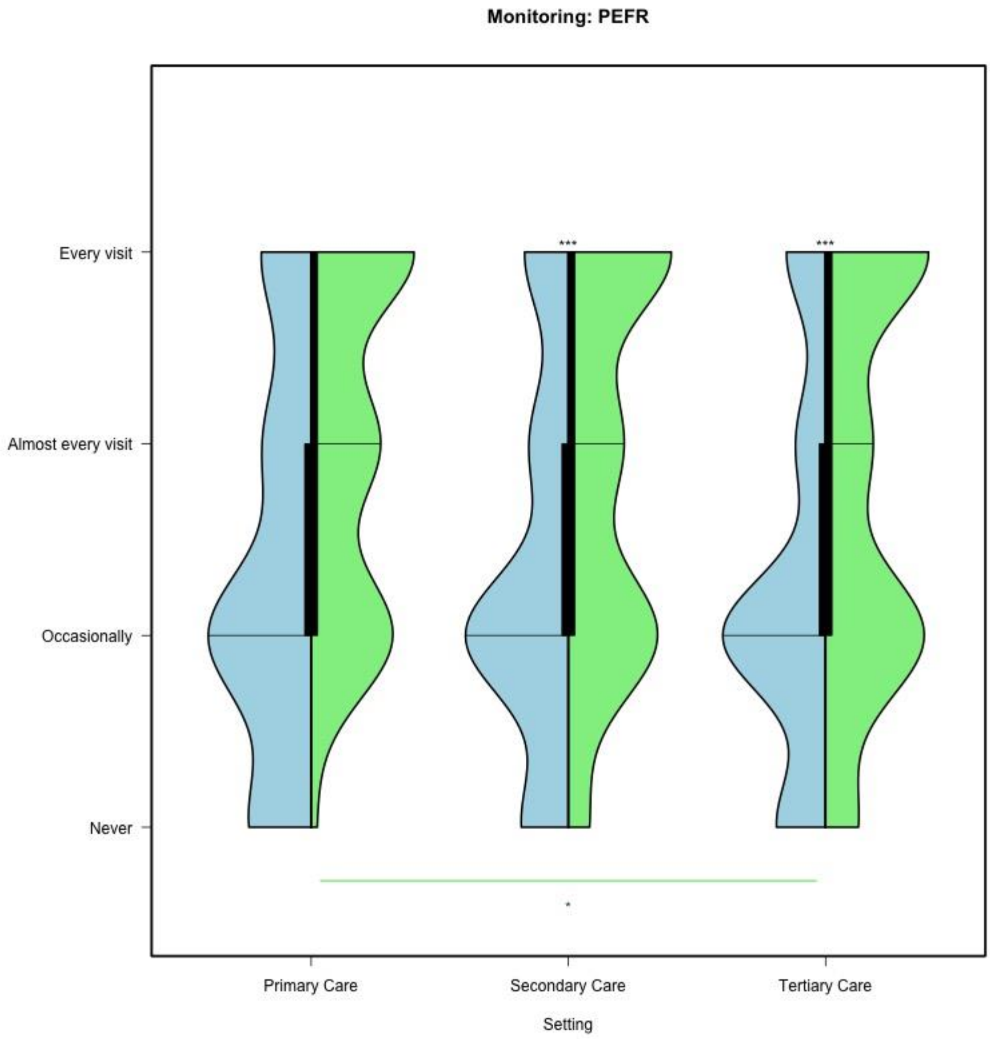

© 2023 Papadopoulos NG et al. *JAMA Network Open.*

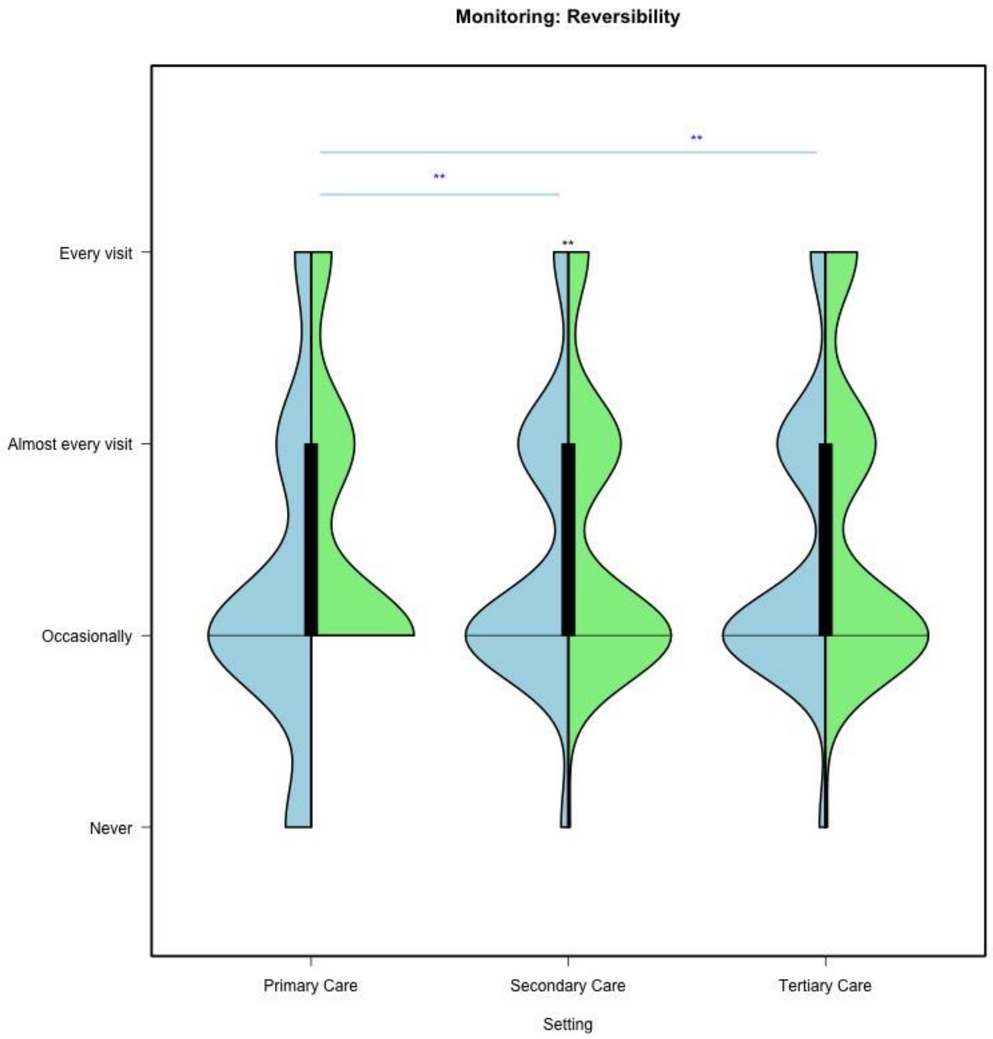

© 2023 Papadopoulos NG et al. *JAMA Network Open.*

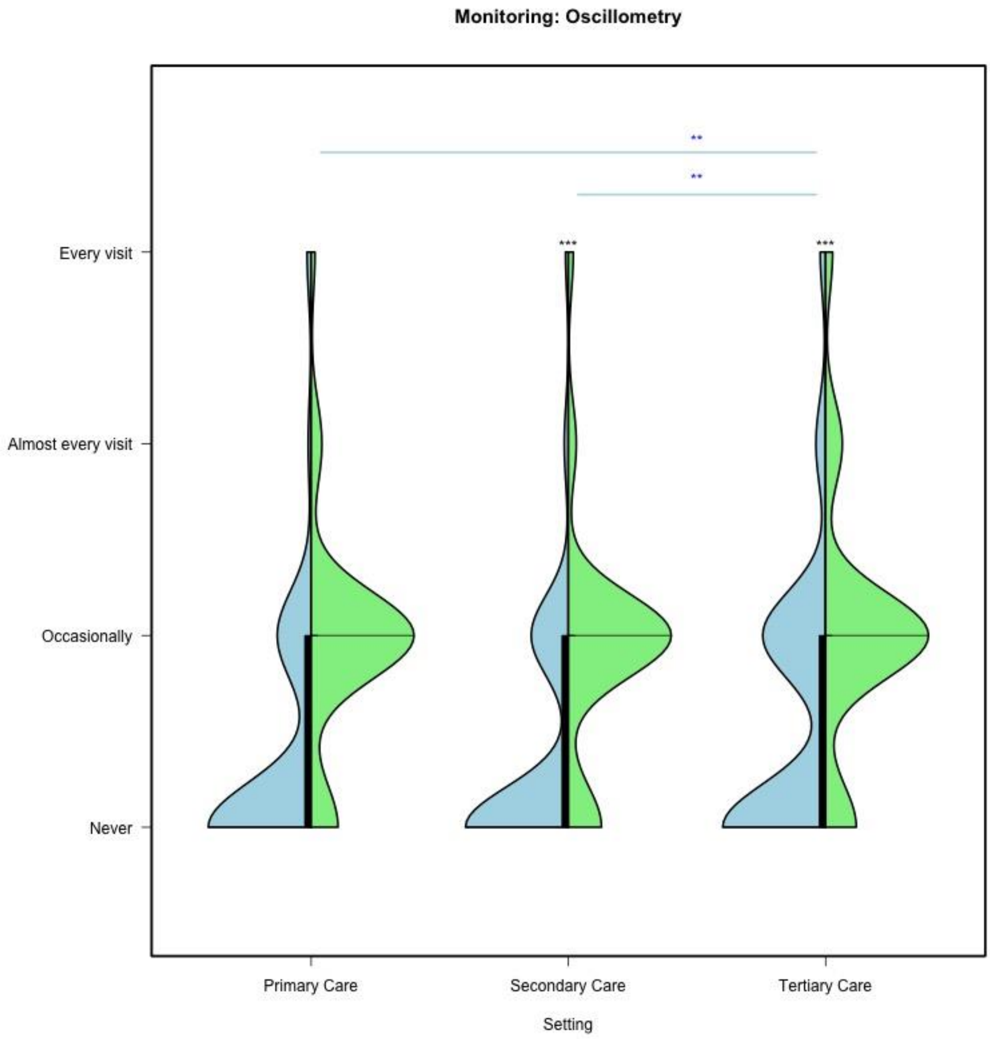

© 2023 Papadopoulos NG et al. *JAMA Network Open.*

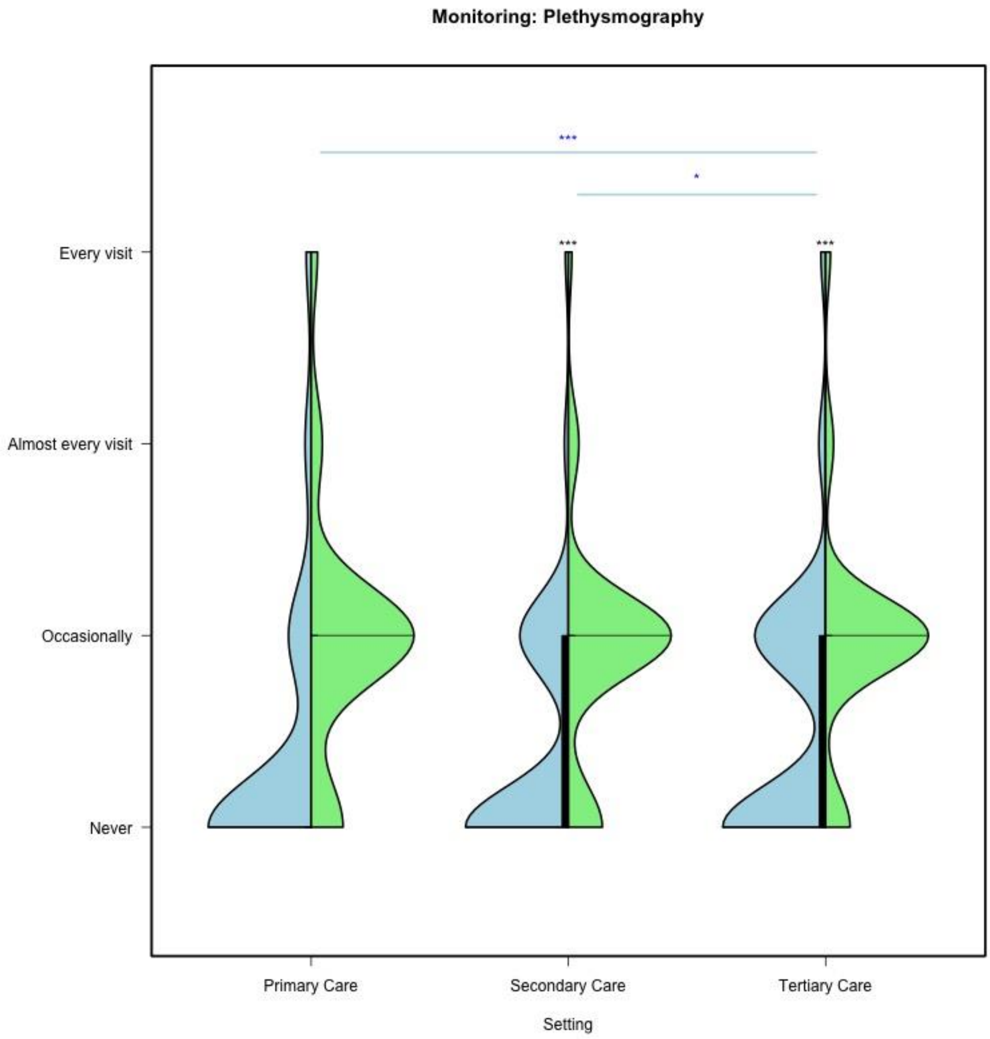

© 2023 Papadopoulos NG et al. *JAMA Network Open.*

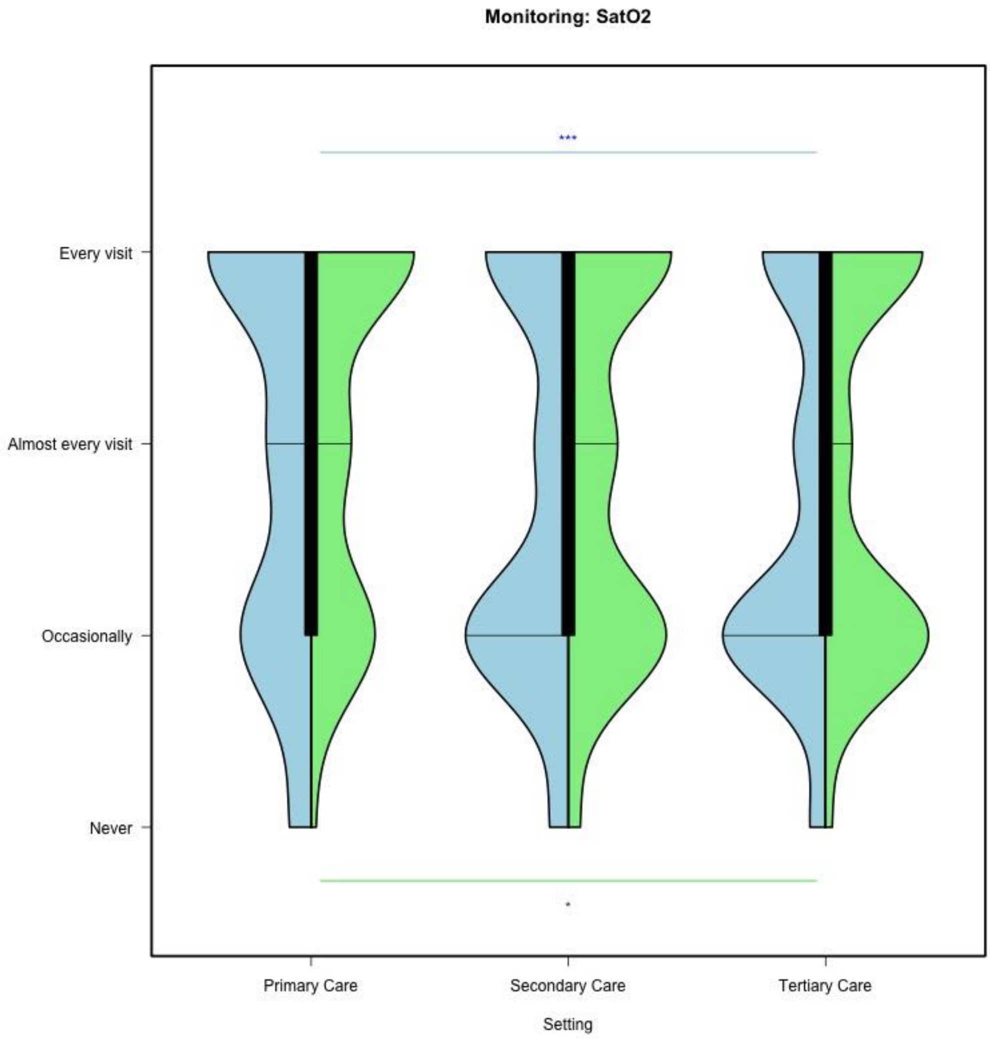

© 2023 Papadopoulos NG et al. *JAMA Network Open.*

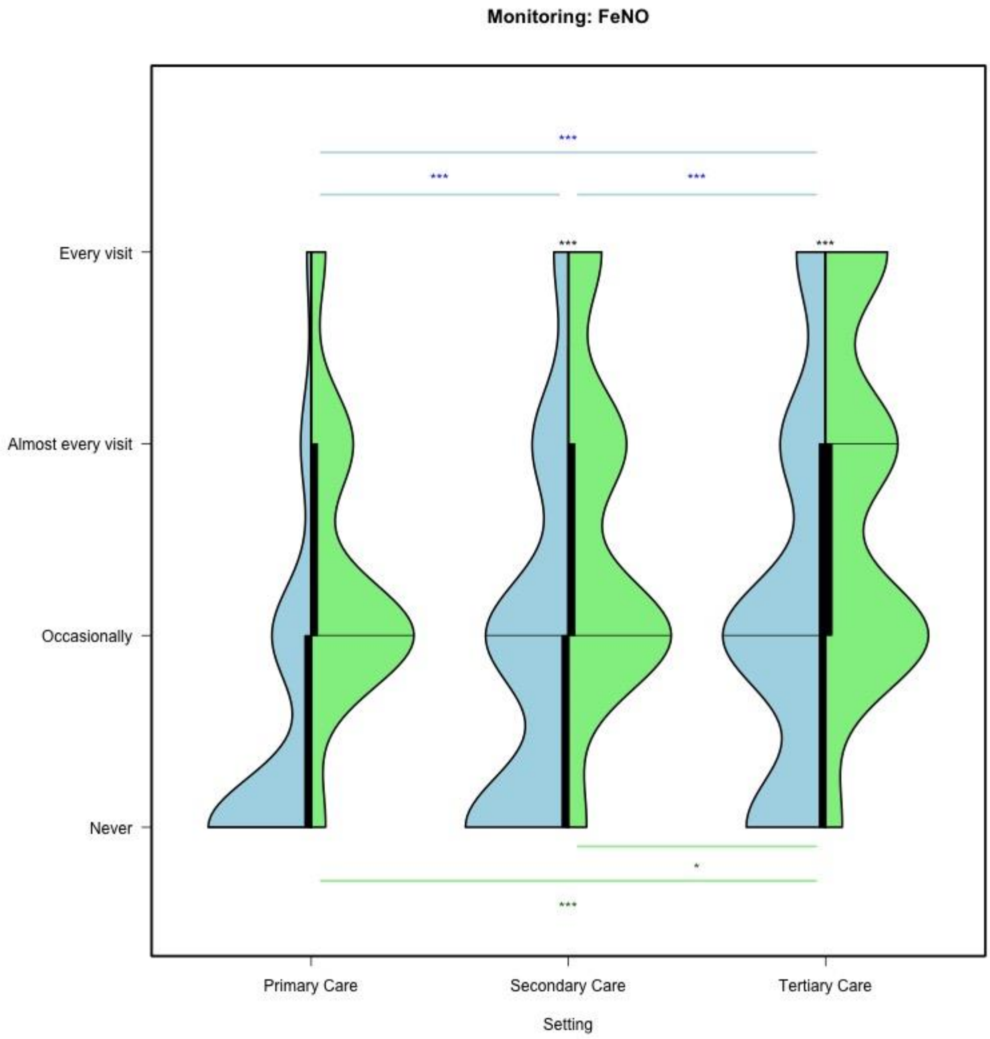

© 2023 Papadopoulos NG et al. *JAMA Network Open.*

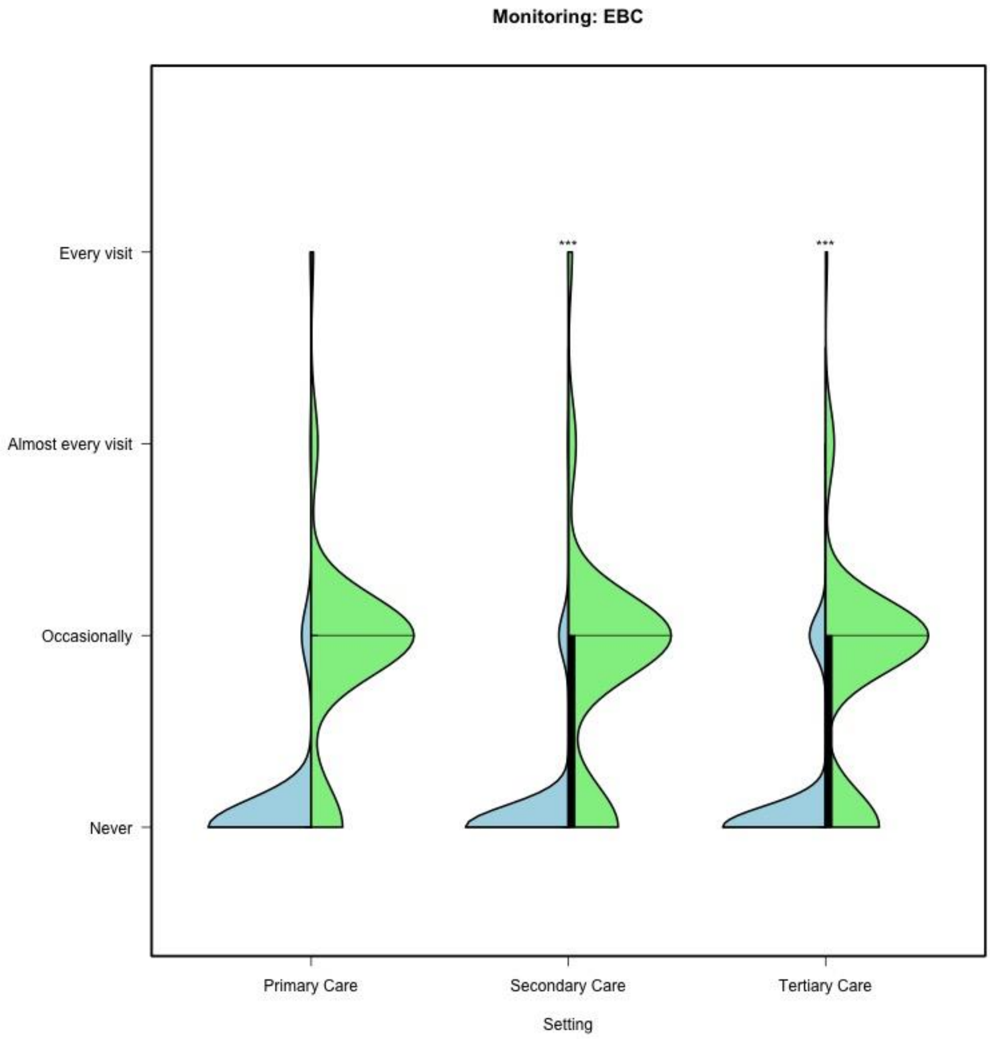

© 2023 Papadopoulos NG et al. *JAMA Network Open.*

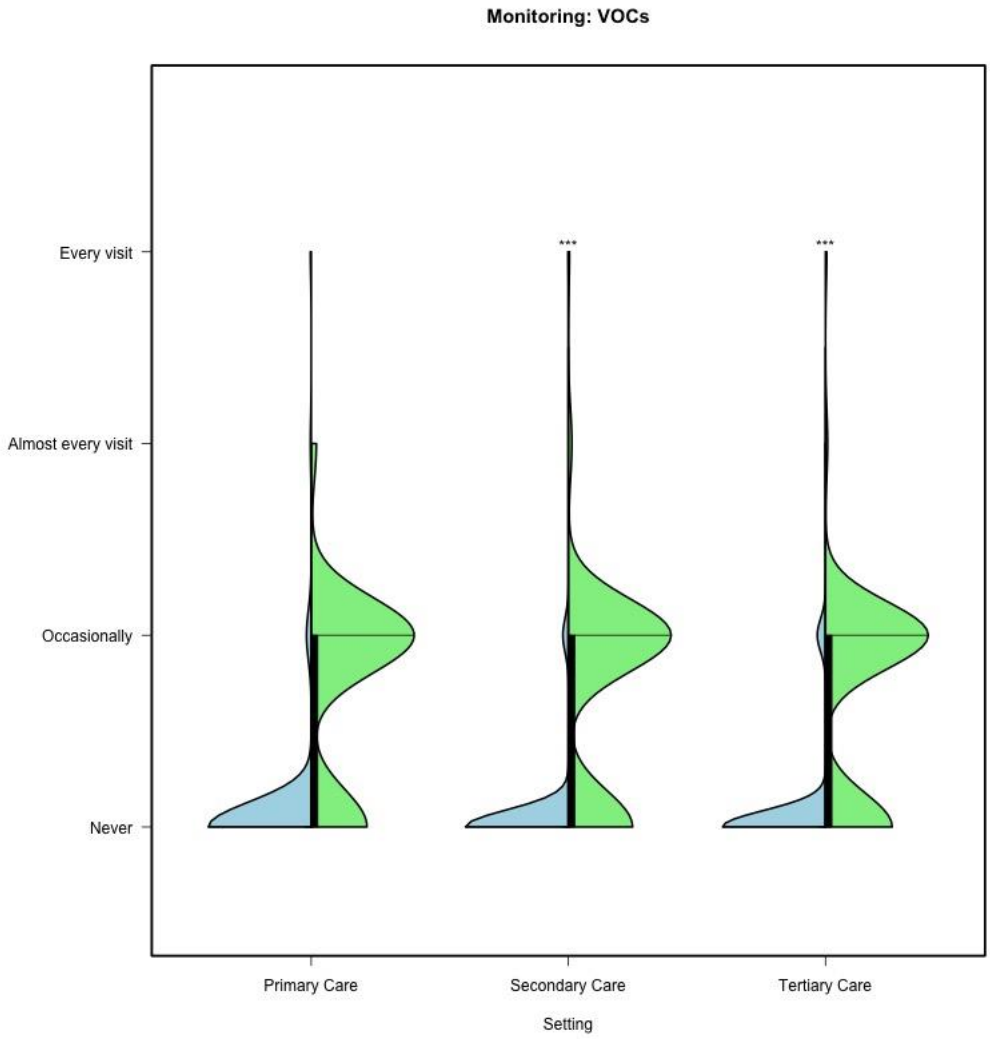

© 2023 Papadopoulos NG et al. *JAMA Network Open.*

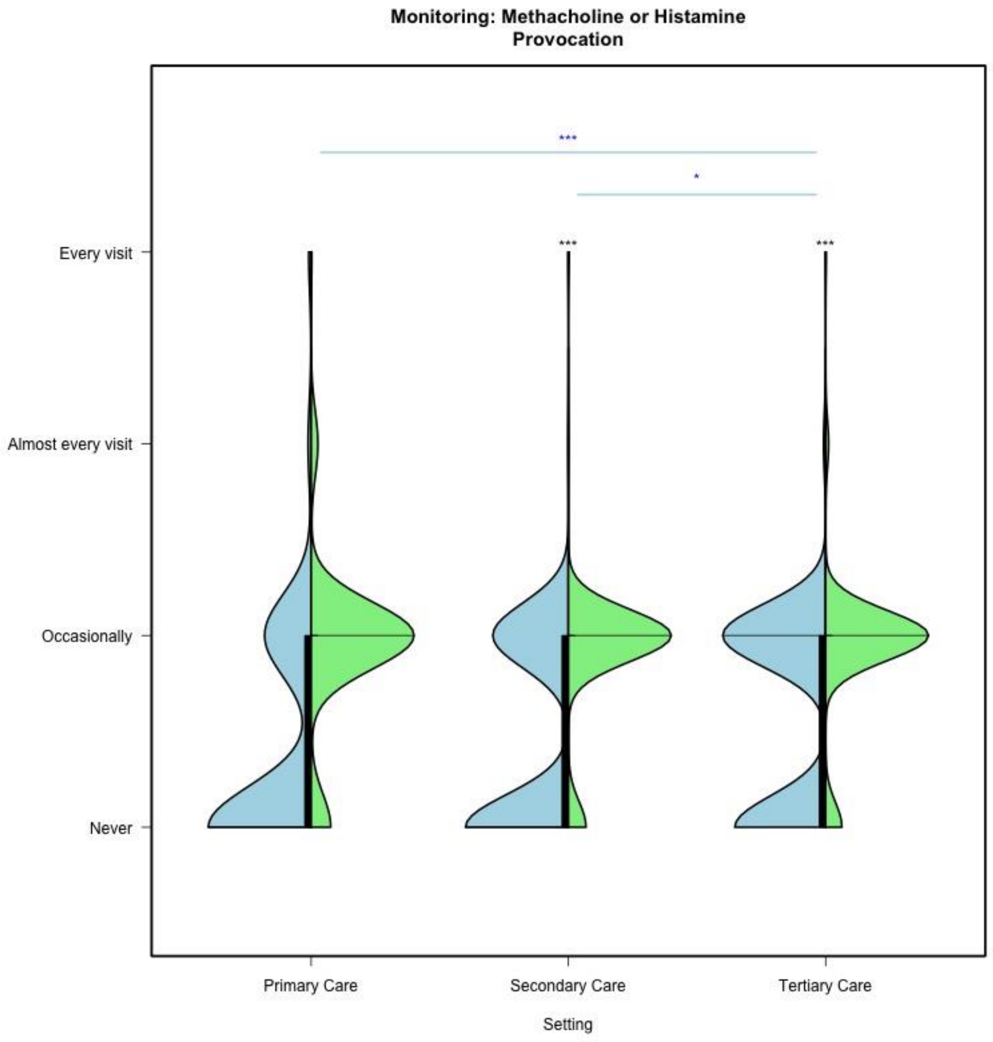

© 2023 Papadopoulos NG et al. *JAMA Network Open.*

Monitoring: Mannitol or Adenosine Provocation

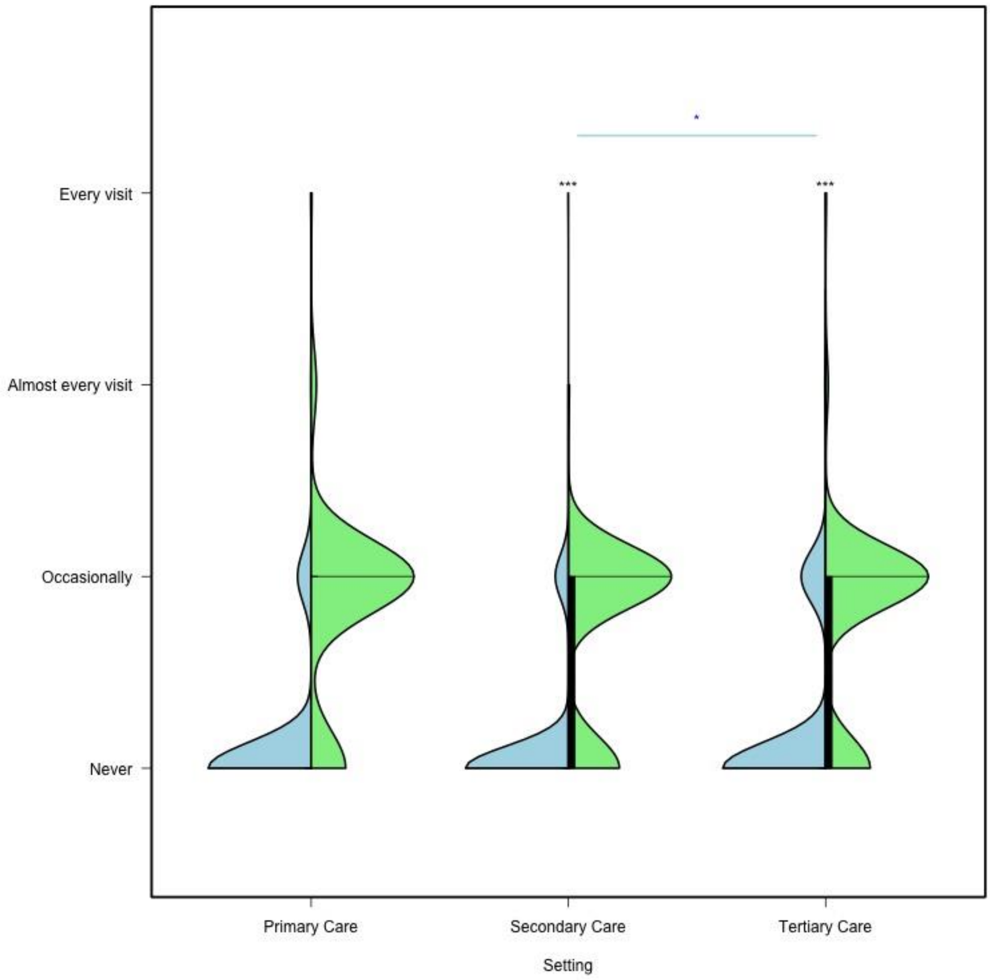

© 2023 Papadopoulos NG et al. *JAMA Network Open.*

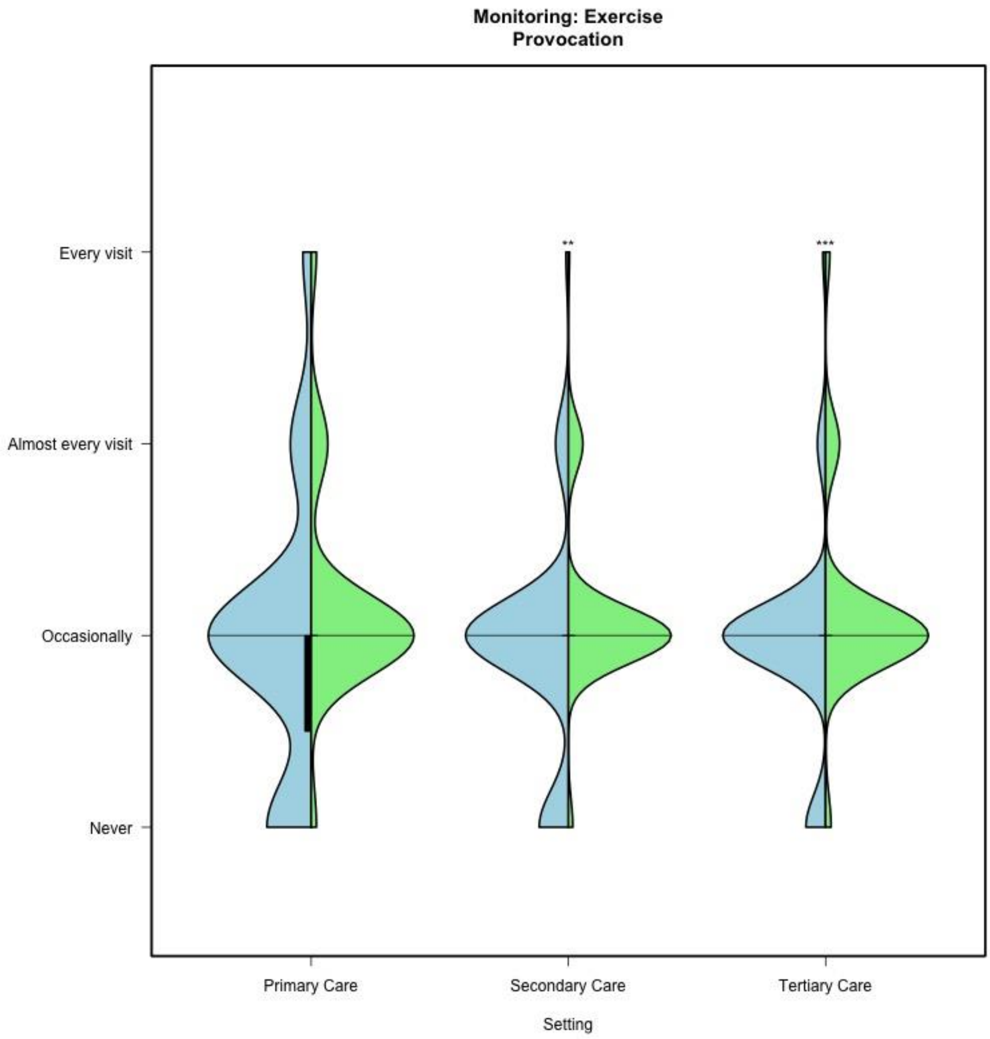

© 2023 Papadopoulos NG et al. *JAMA Network Open.*

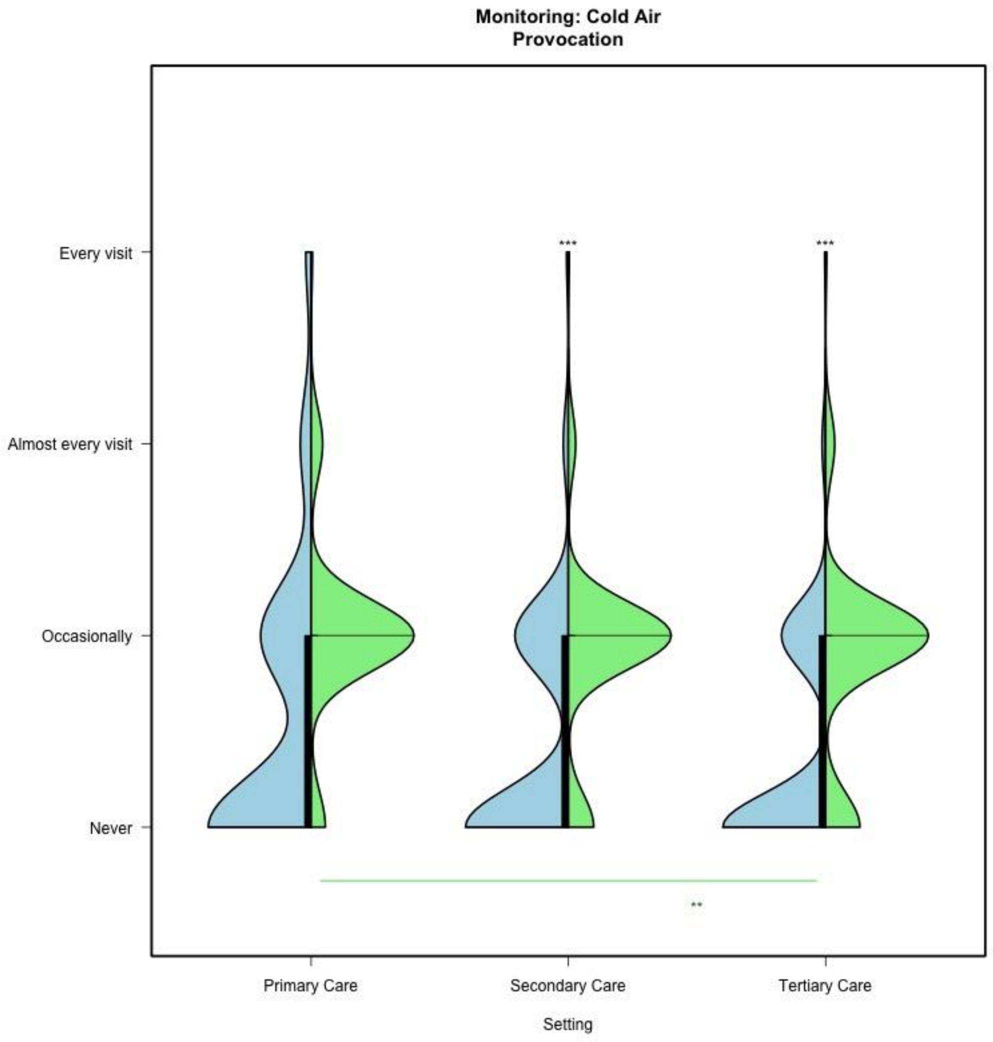

© 2023 Papadopoulos NG et al. *JAMA Network Open.*

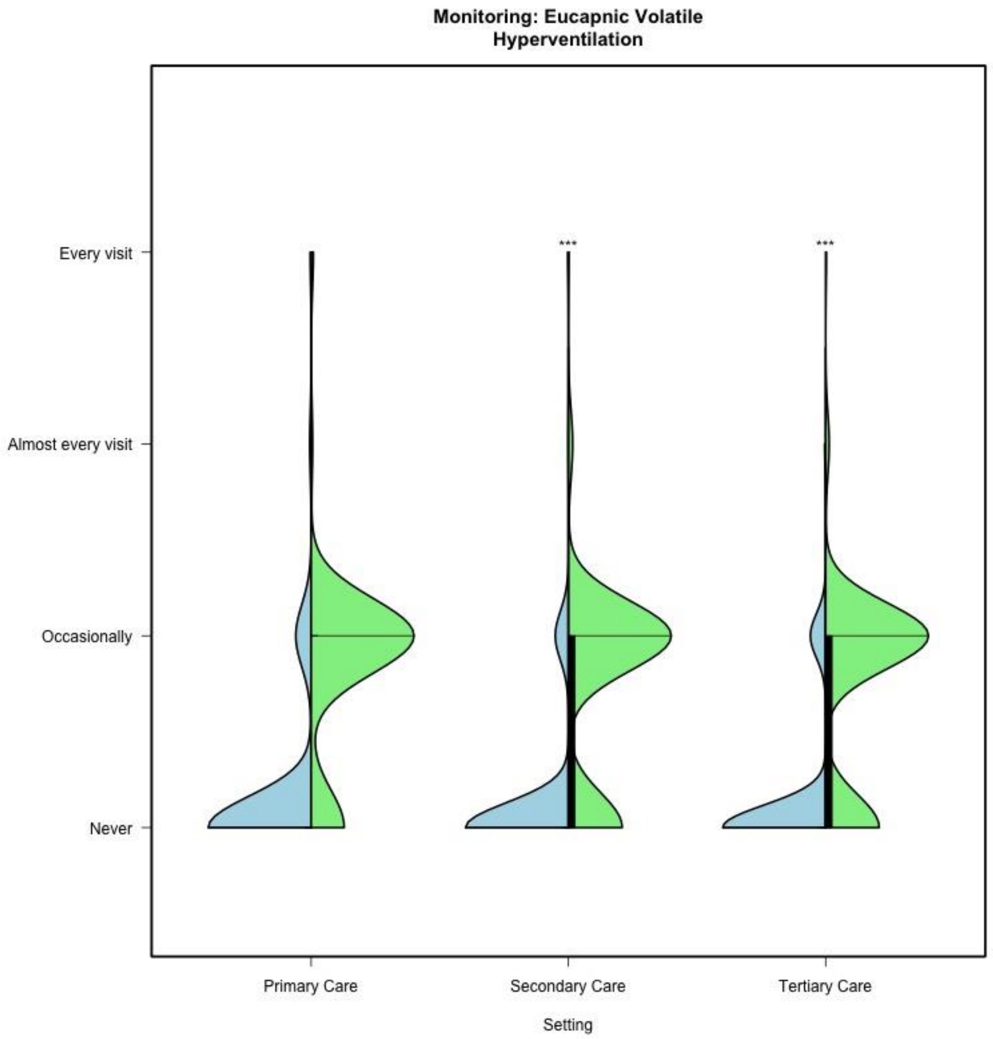

© 2023 Papadopoulos NG et al. *JAMA Network Open.*

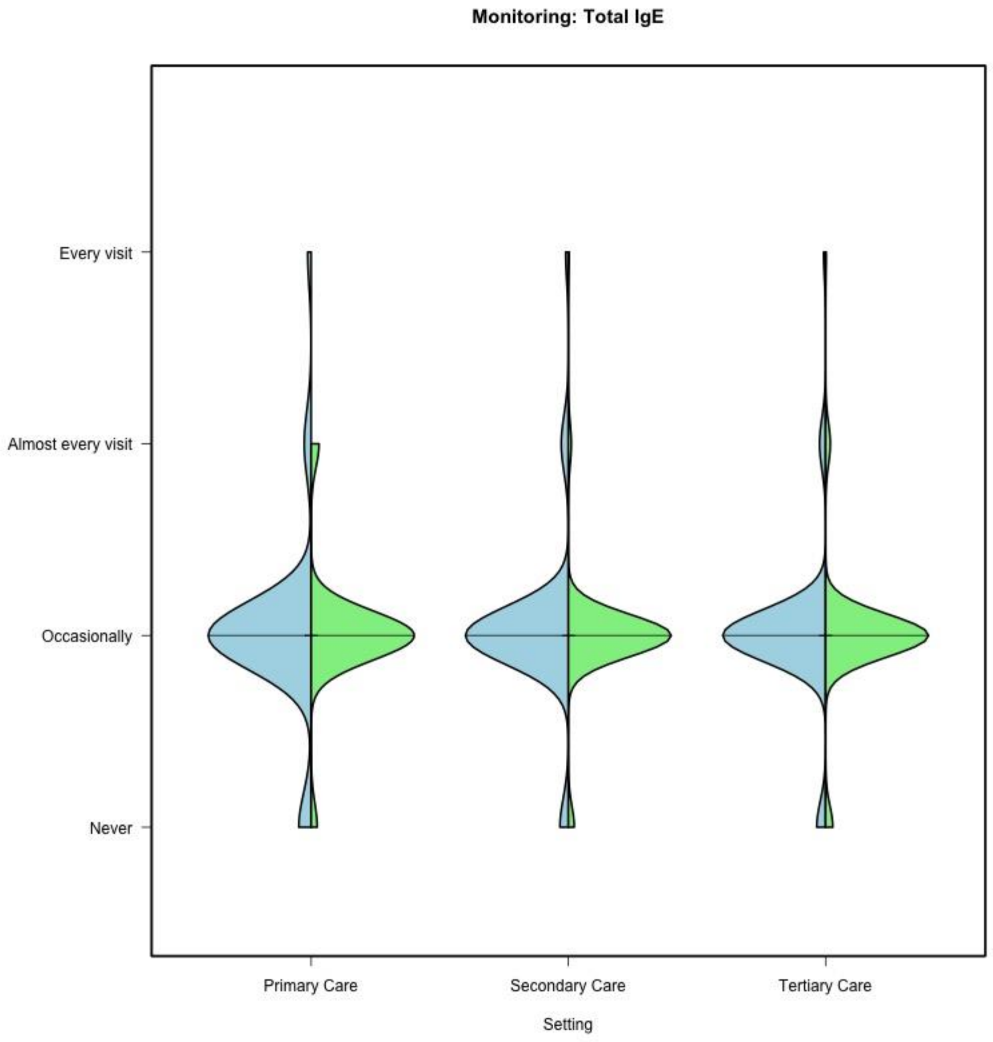

© 2023 Papadopoulos NG et al. *JAMA Network Open.*

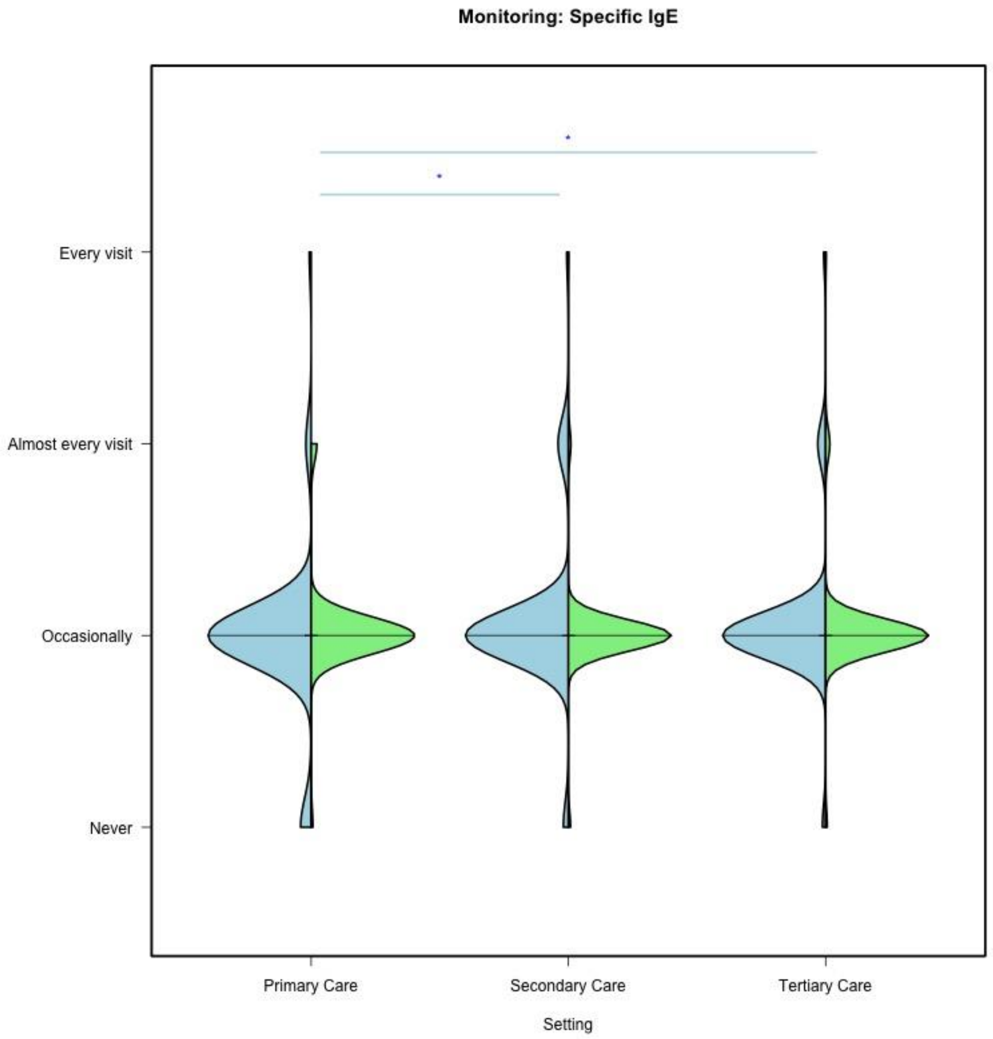

© 2023 Papadopoulos NG et al. *JAMA Network Open.*

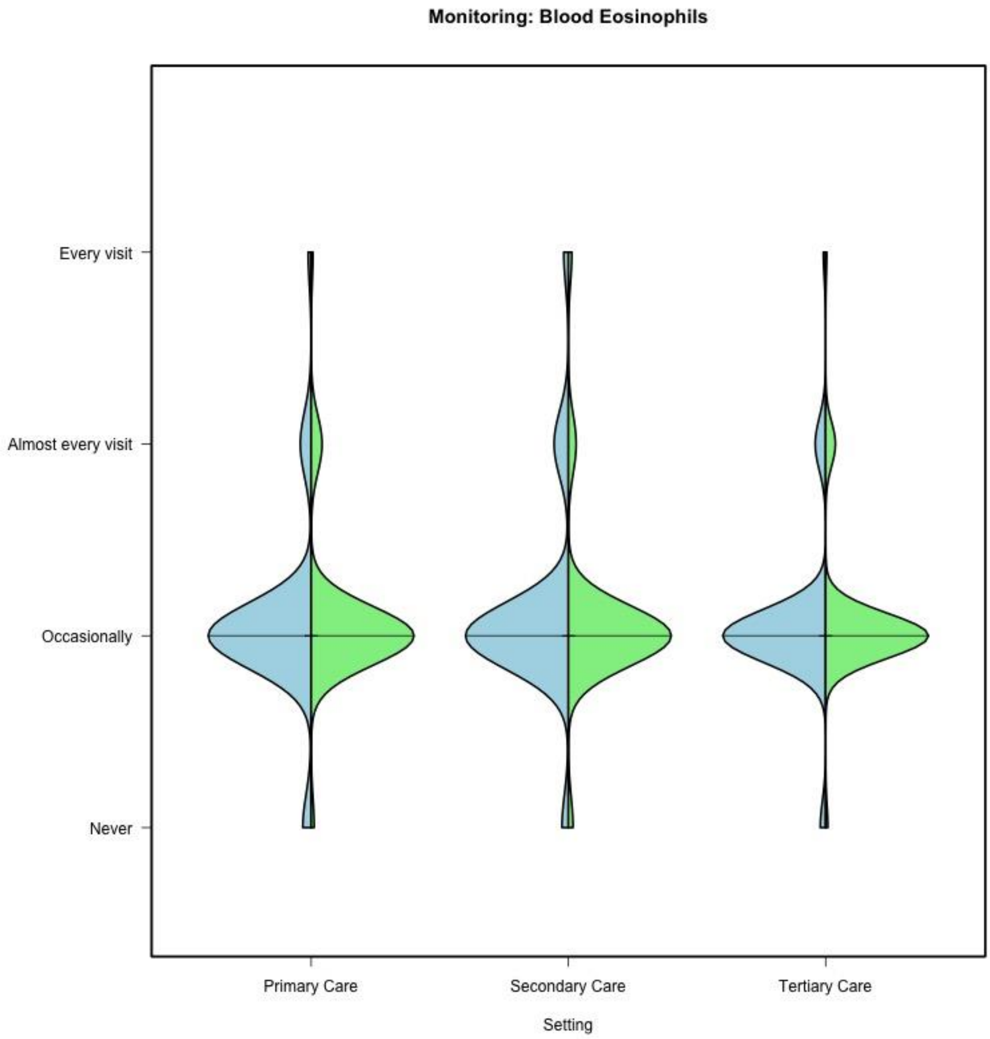

© 2023 Papadopoulos NG et al. *JAMA Network Open.*

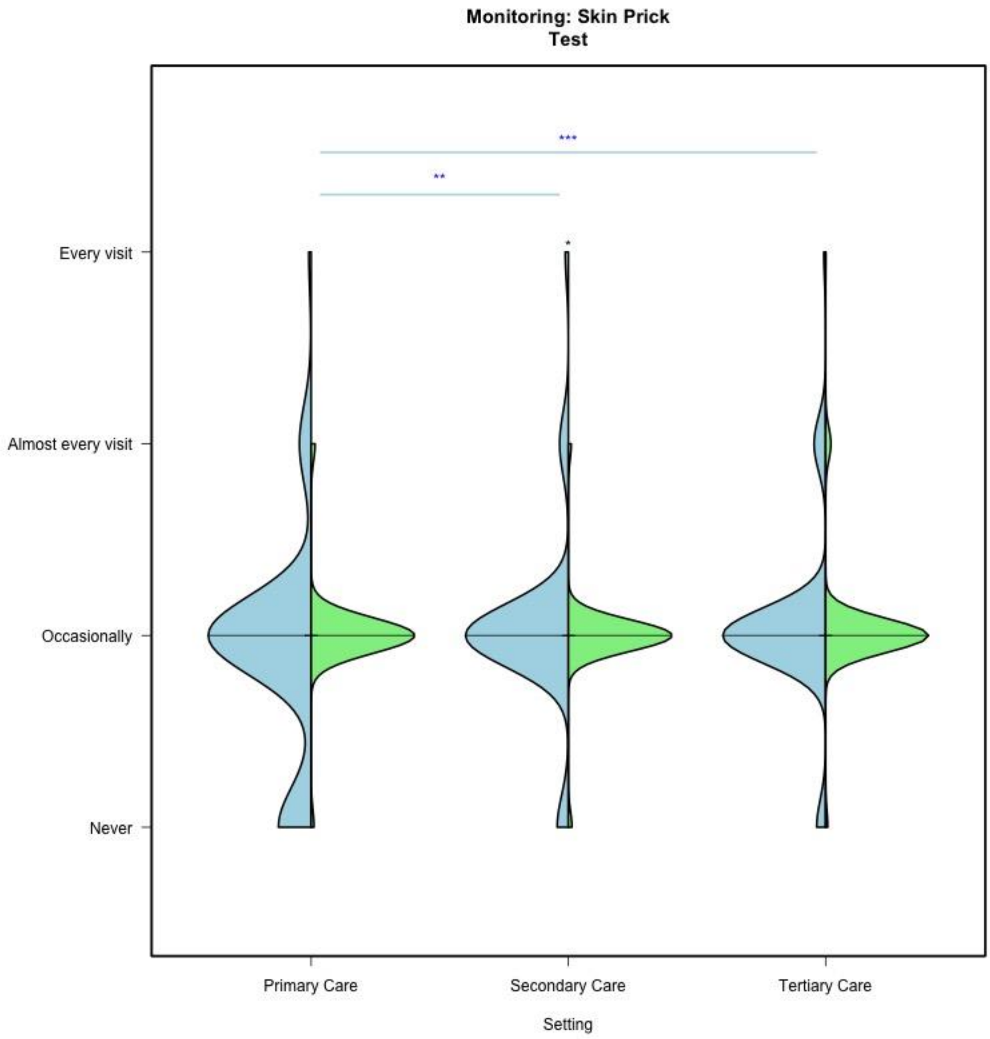

© 2023 Papadopoulos NG et al. *JAMA Network Open.*

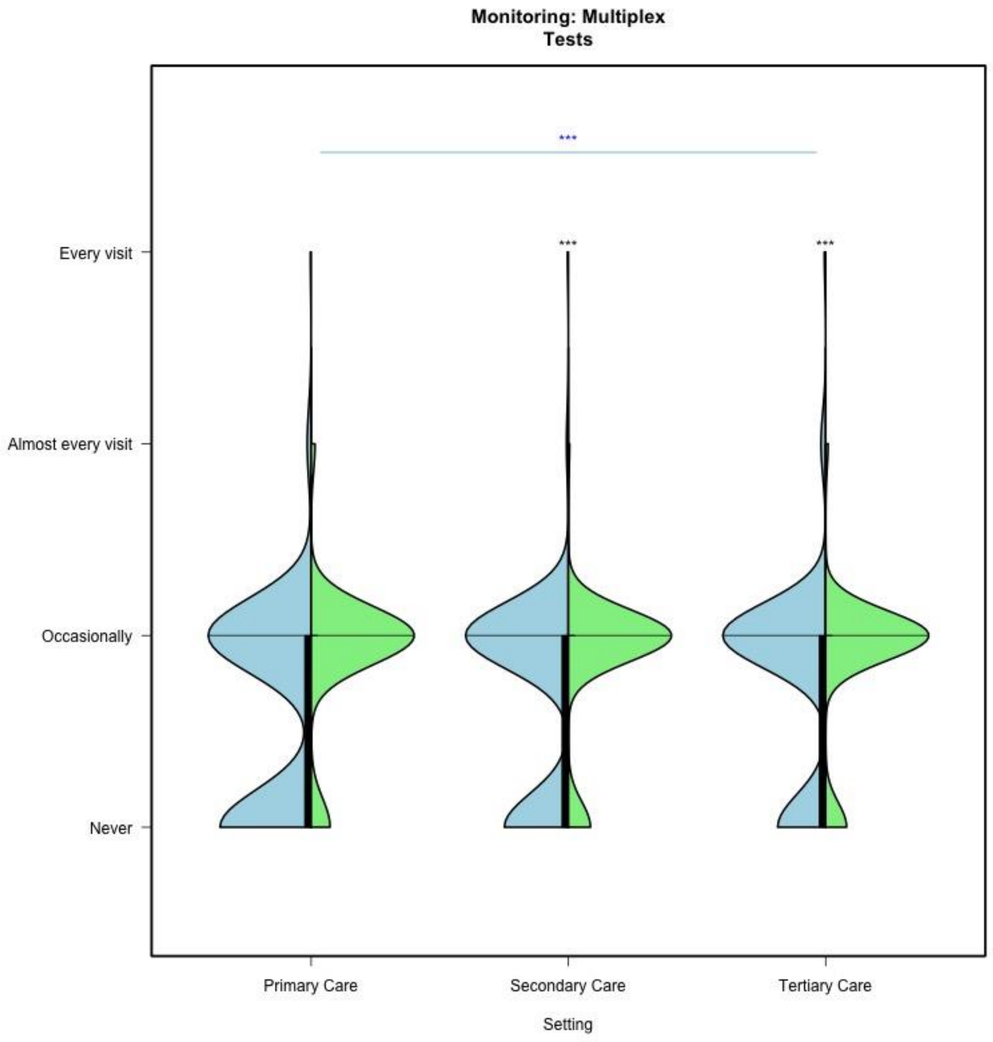

© 2023 Papadopoulos NG et al. *JAMA Network Open.*

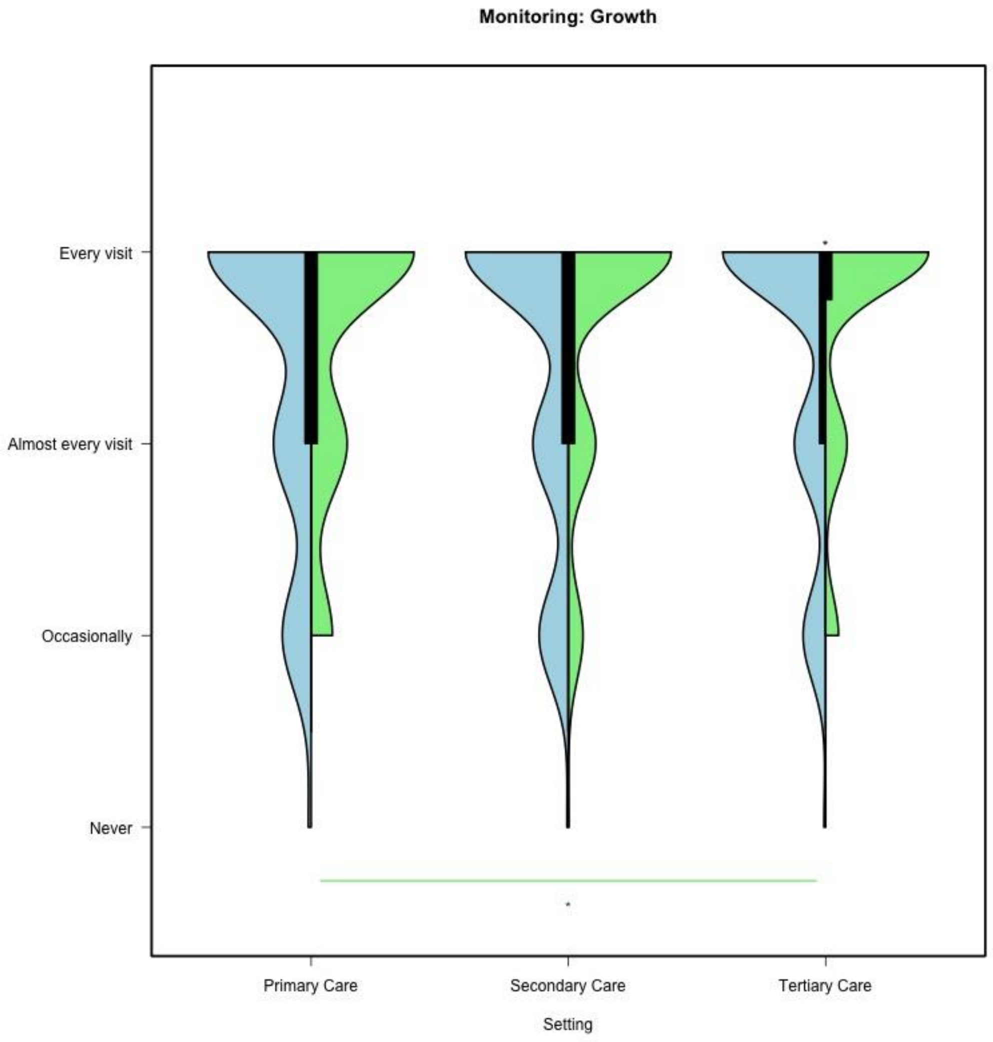

© 2023 Papadopoulos NG et al. *JAMA Network Open.*

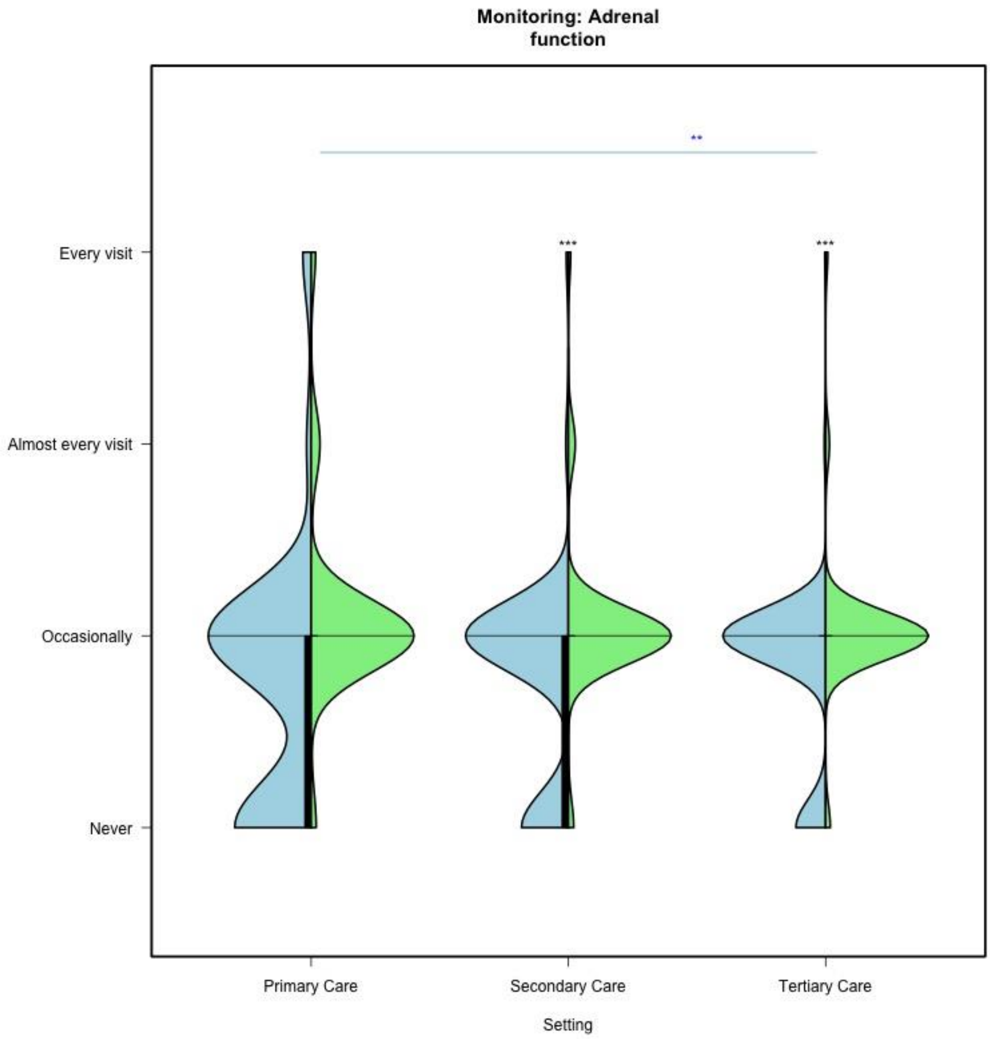

© 2023 Papadopoulos NG et al. *JAMA Network Open.*

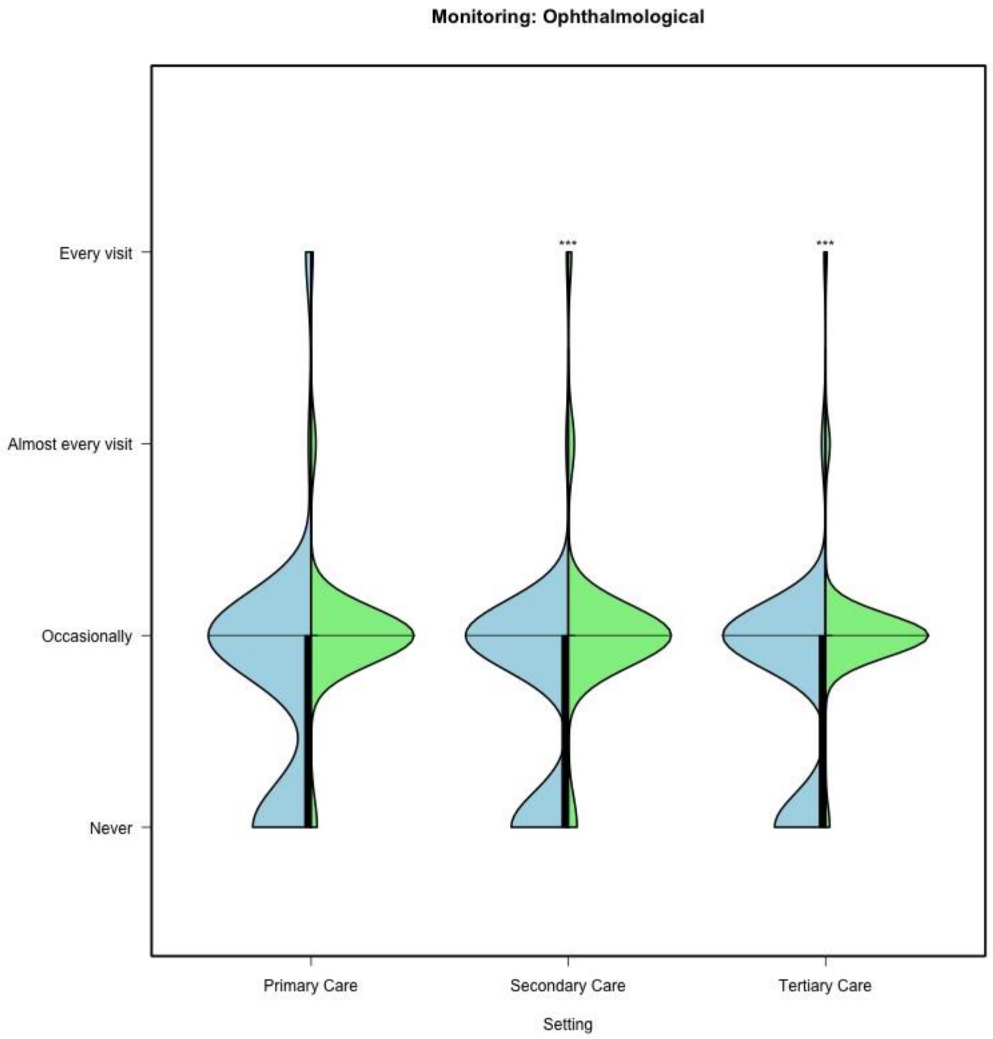

© 2023 Papadopoulos NG et al. *JAMA Network Open.*

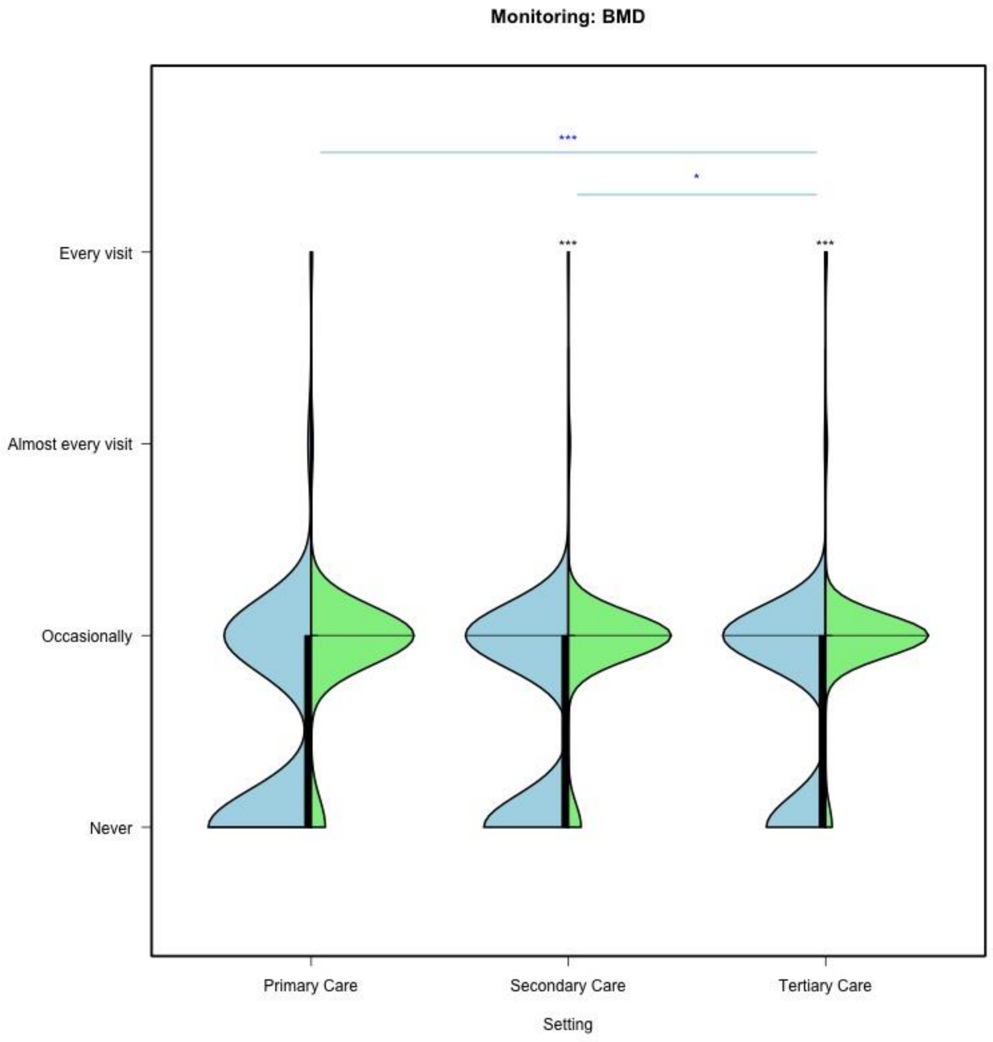

© 2023 Papadopoulos NG et al. *JAMA Network Open.*

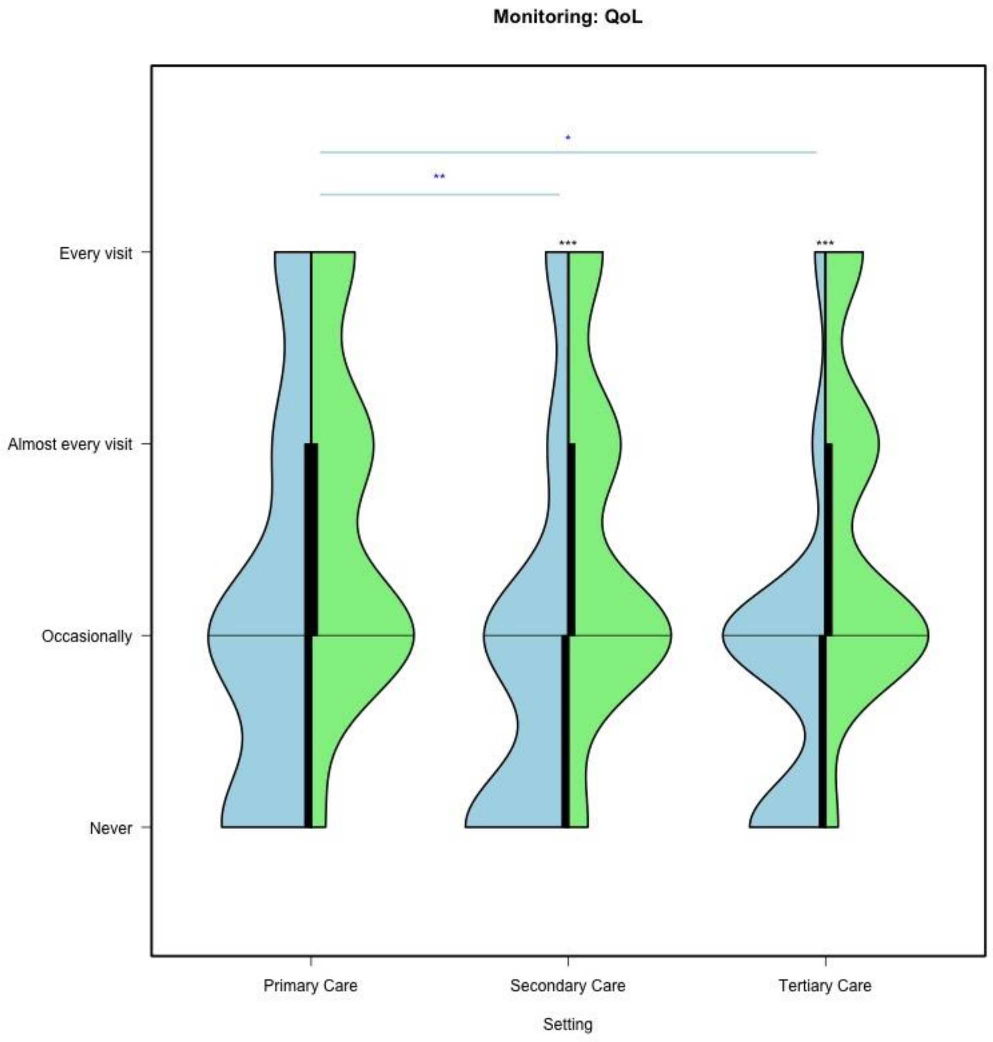

© 2023 Papadopoulos NG et al. *JAMA Network Open.*

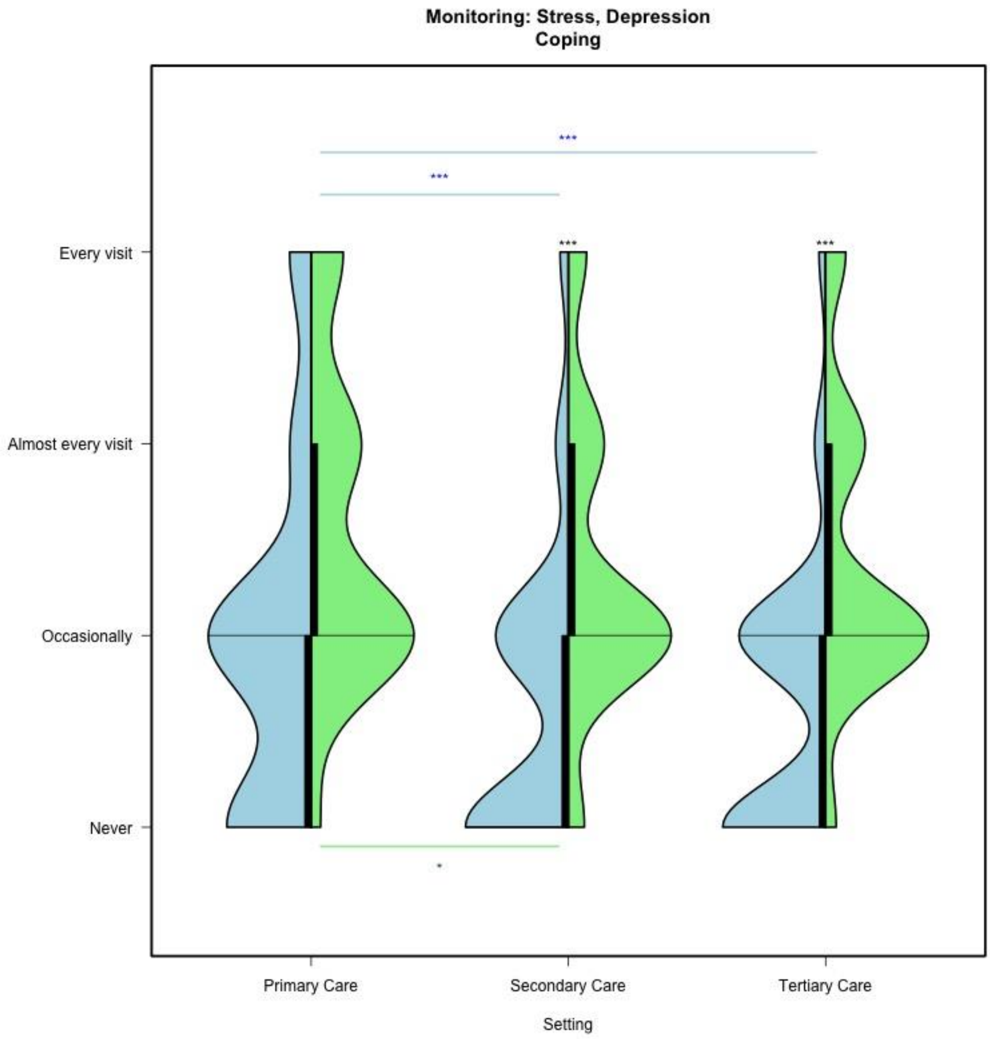

© 2023 Papadopoulos NG et al. *JAMA Network Open.*

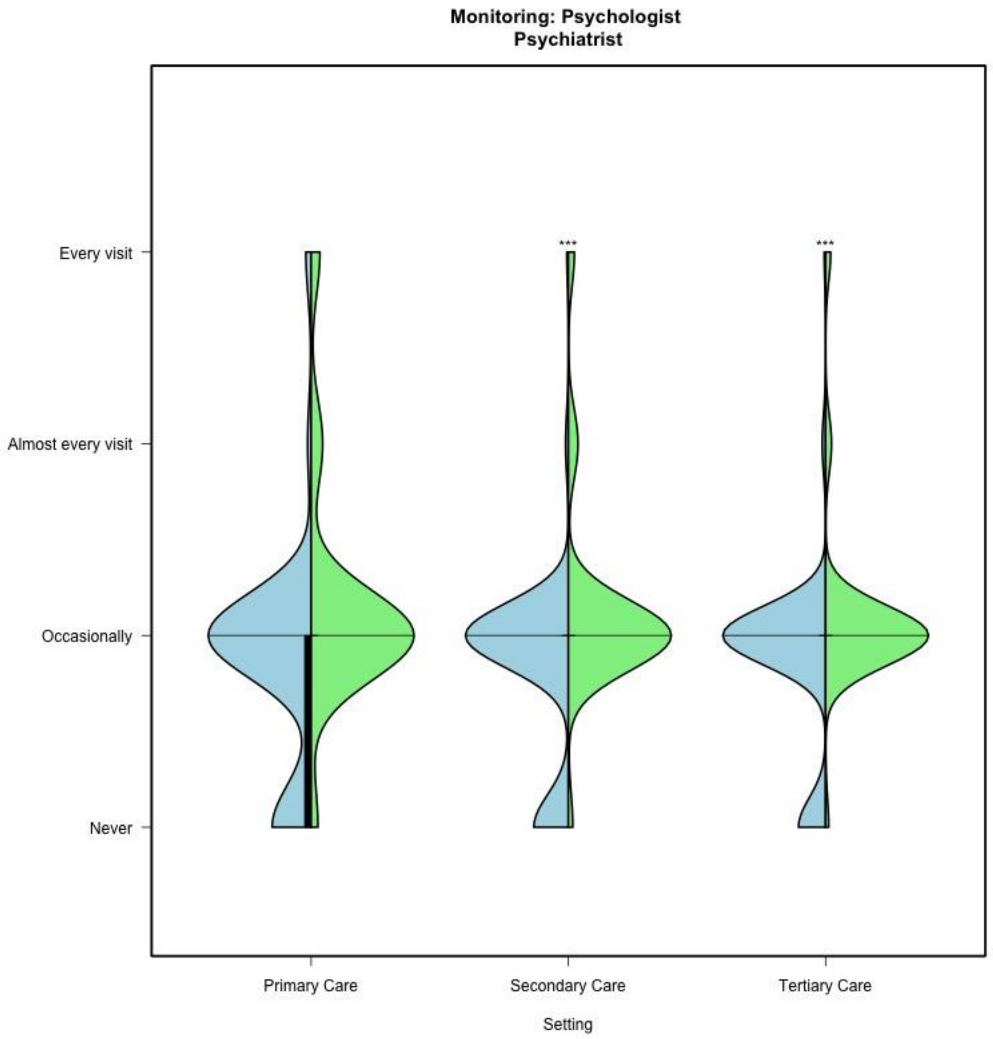

© 2023 Papadopoulos NG et al. *JAMA Network Open.*

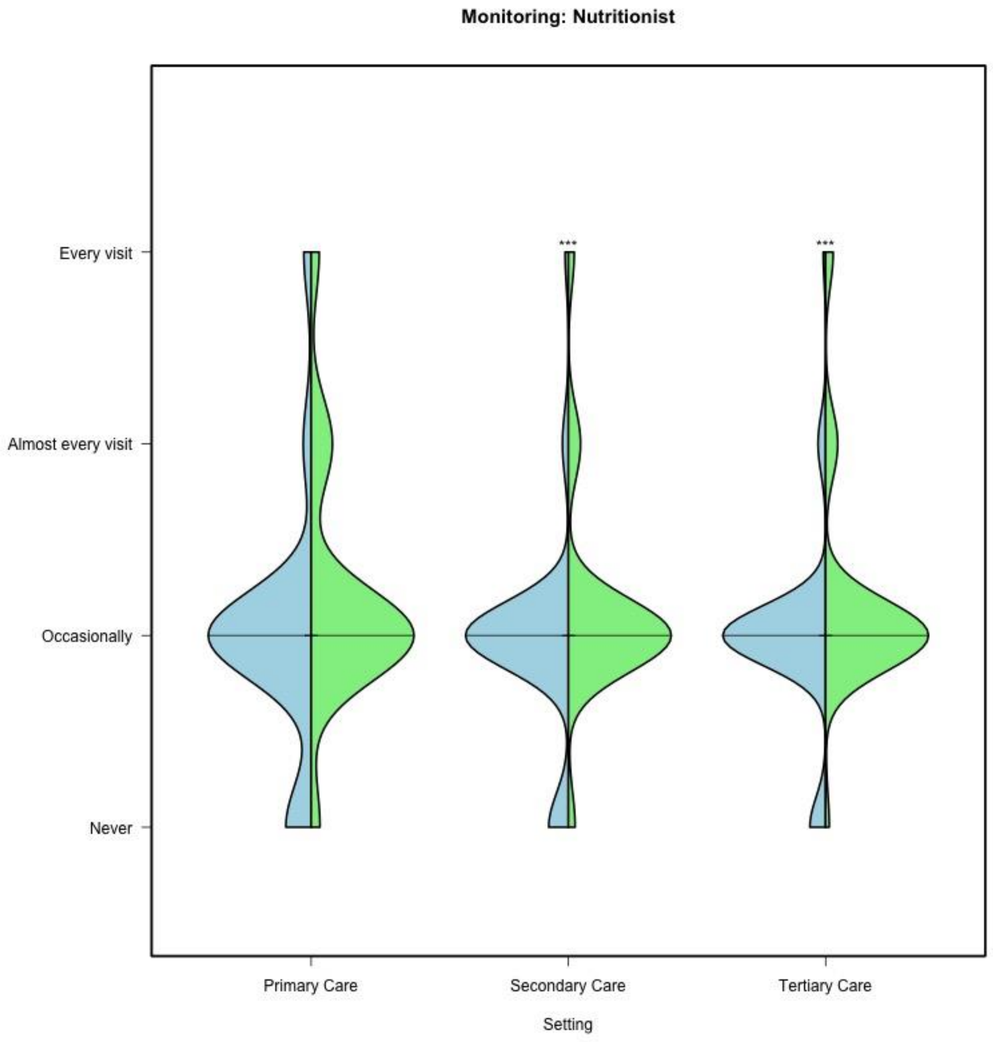

© 2023 Papadopoulos NG et al. *JAMA Network Open.*

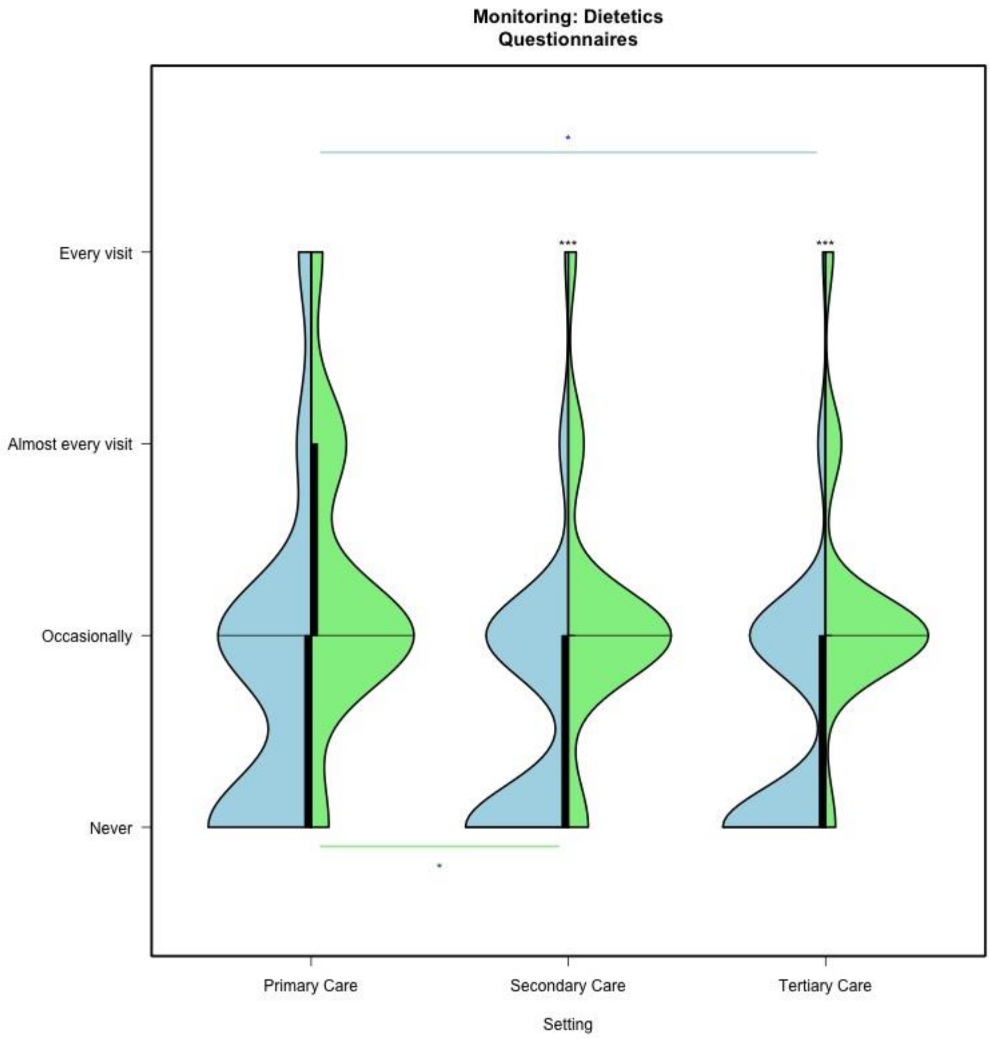

© 2023 Papadopoulos NG et al. *JAMA Network Open.*

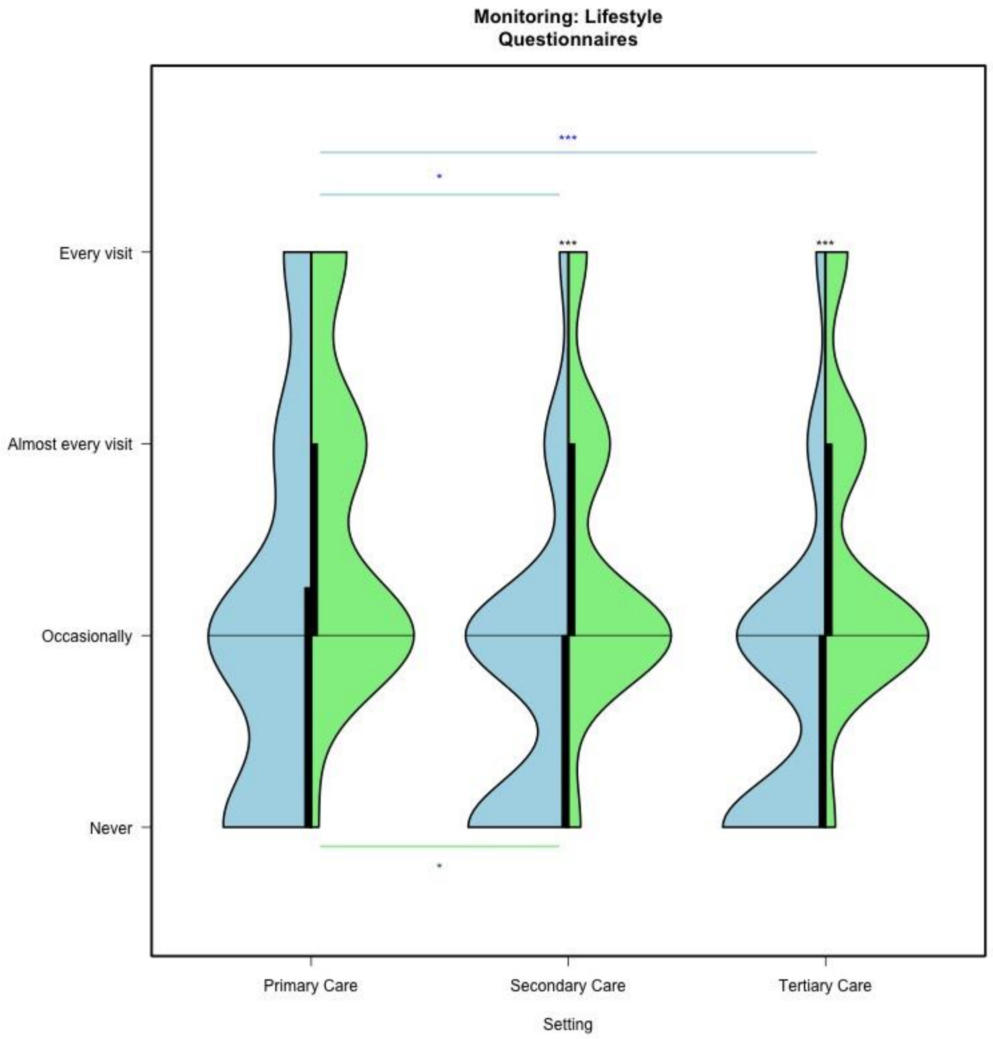

© 2023 Papadopoulos NG et al. *JAMA Network Open.*

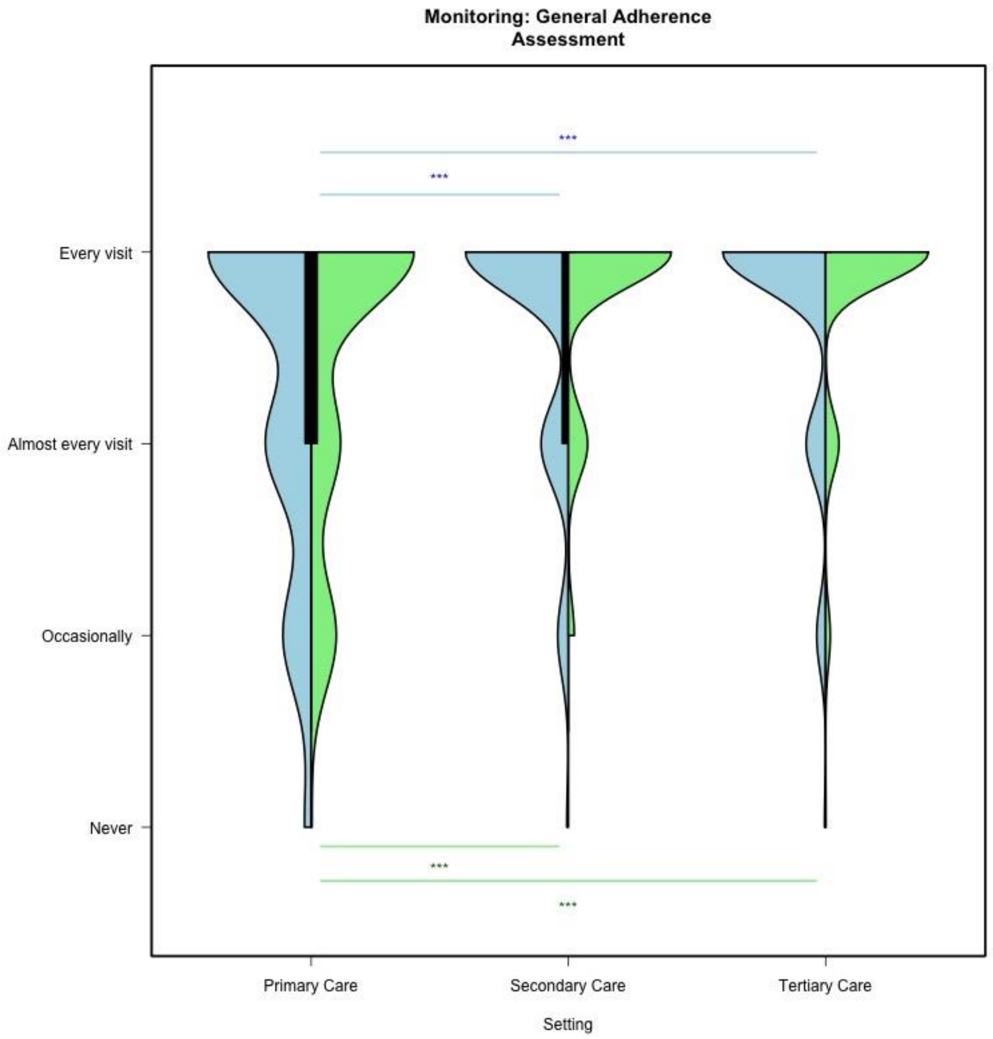

© 2023 Papadopoulos NG et al. *JAMA Network Open.*

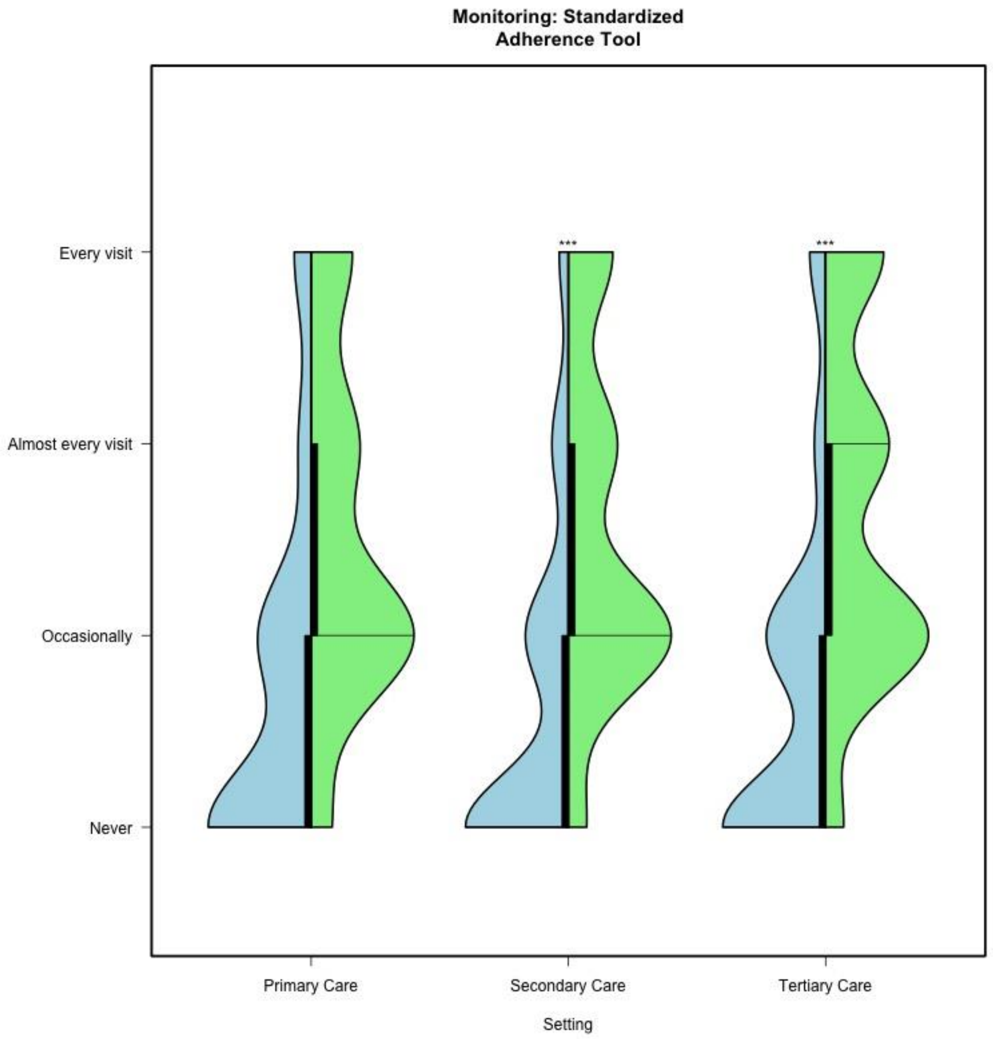

© 2023 Papadopoulos NG et al. *JAMA Network Open.*

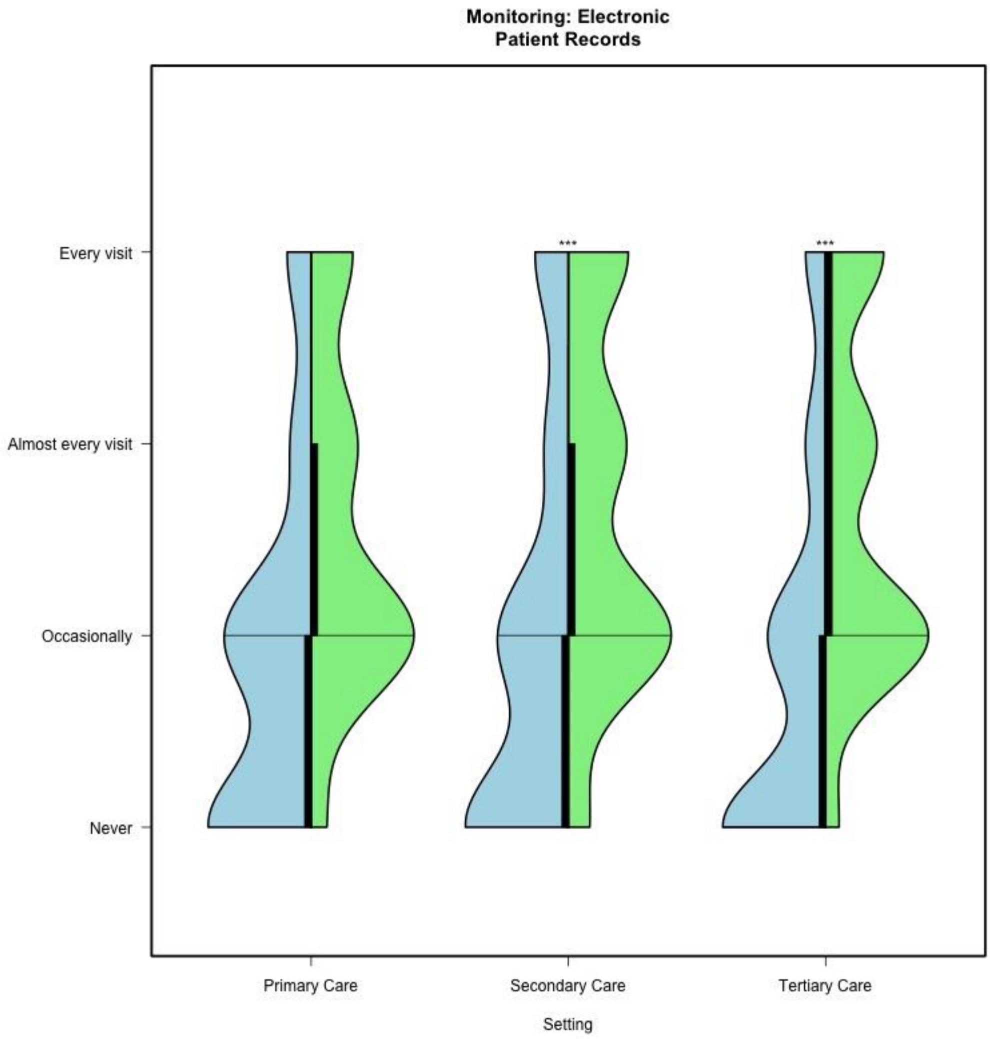

© 2023 Papadopoulos NG et al. *JAMA Network Open.*

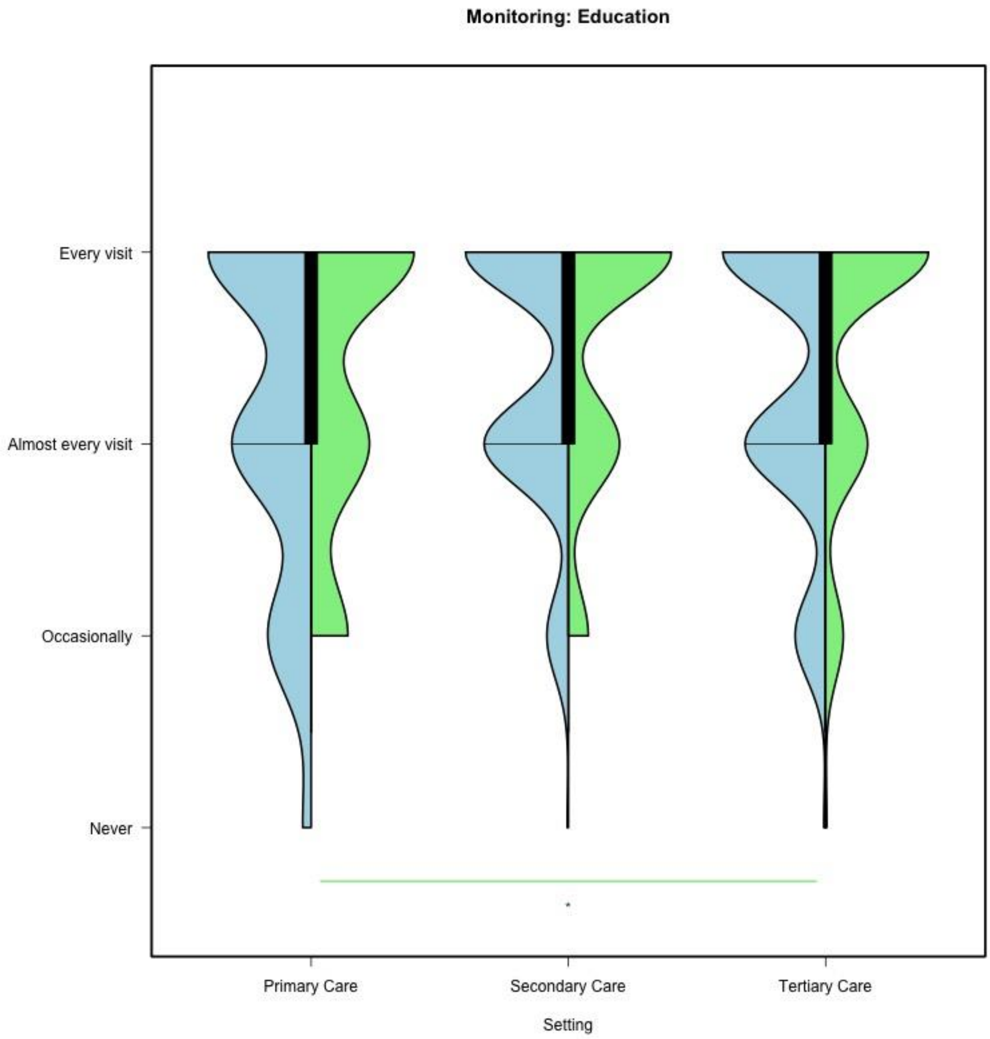

© 2023 Papadopoulos NG et al. *JAMA Network Open.*

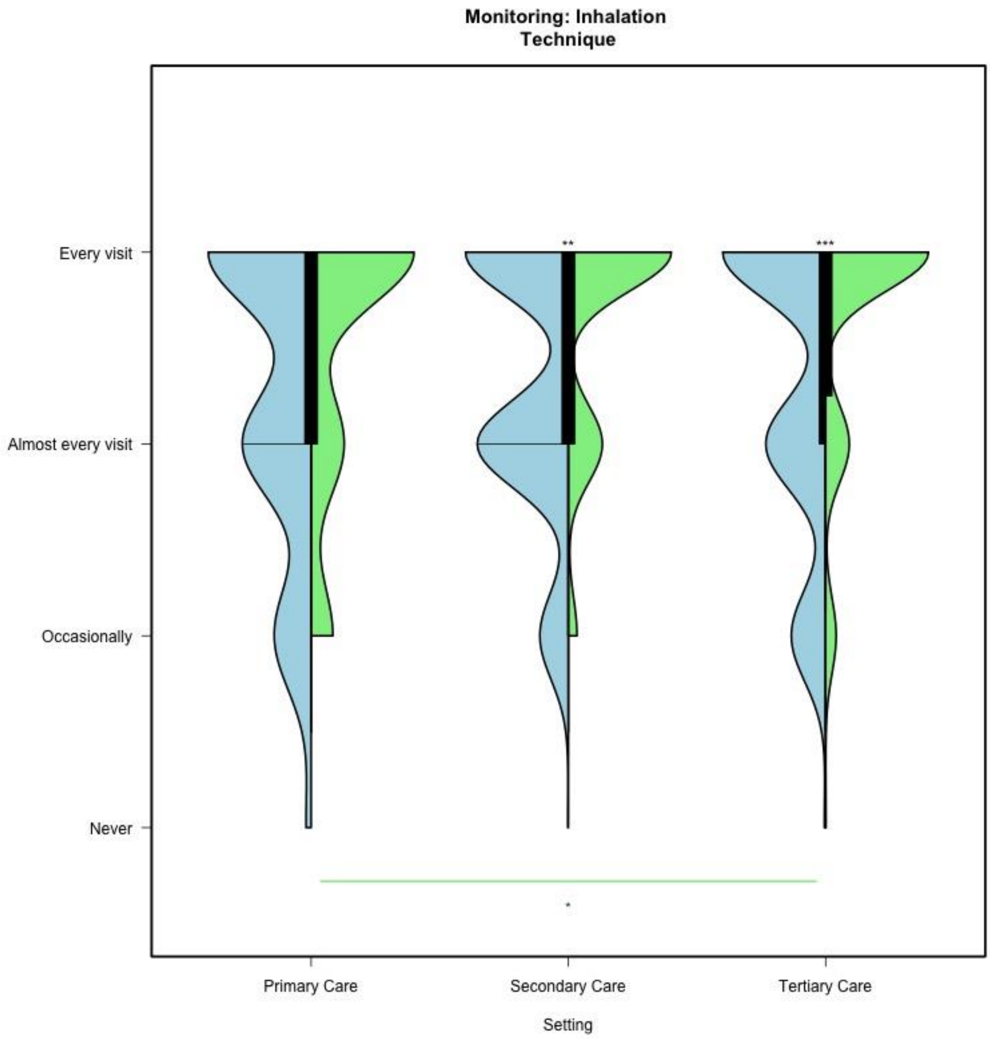

© 2023 Papadopoulos NG et al. *JAMA Network Open.*

eFigure 16. Actual (Light Blue, Left Side of the Violin Plots) and Perceived Optimal (Green, Right Side of the Violin Plots) Use of Monitoring Tools Between Asthma Visits, Stratified by Country Income

Clinicians were asked to report on the approximate percentage of patients they recommend each of the monitoring tools. *: p<0.05; **: p<0.01; ***: p<0.001

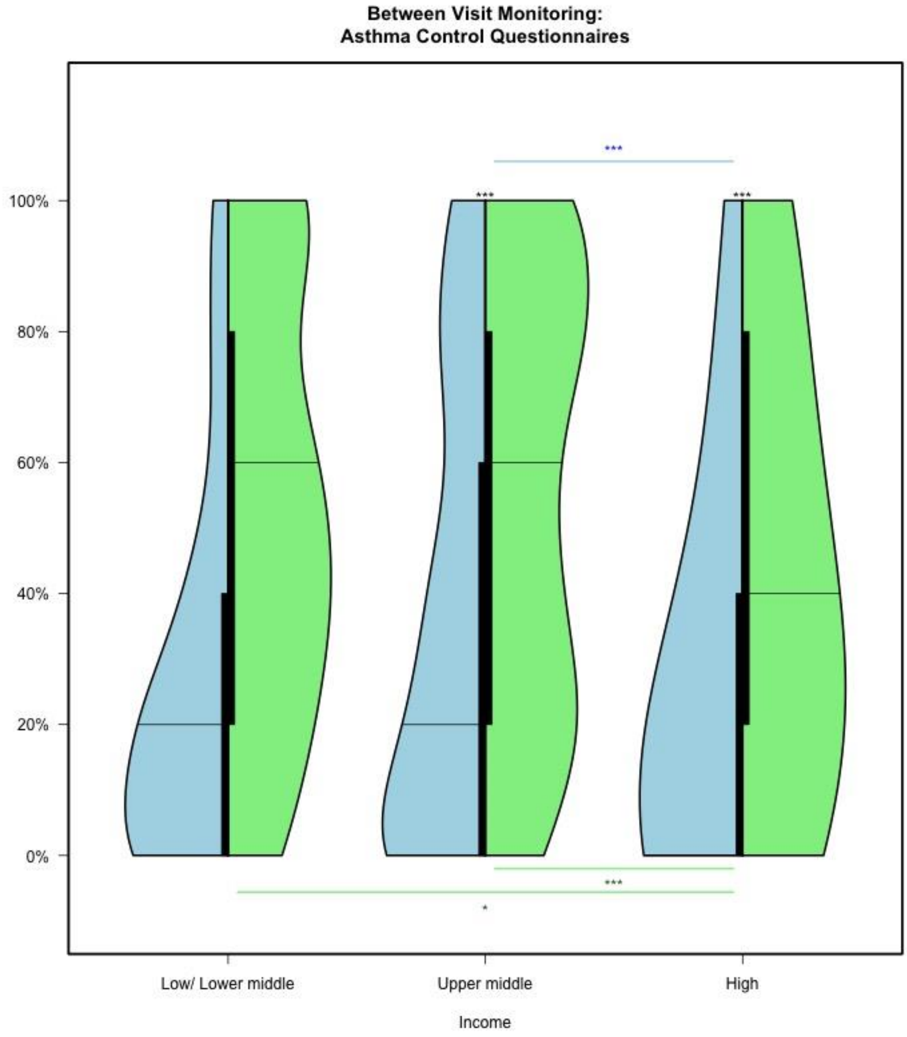

© 2023 Papadopoulos NG et al. *JAMA Network Open.*

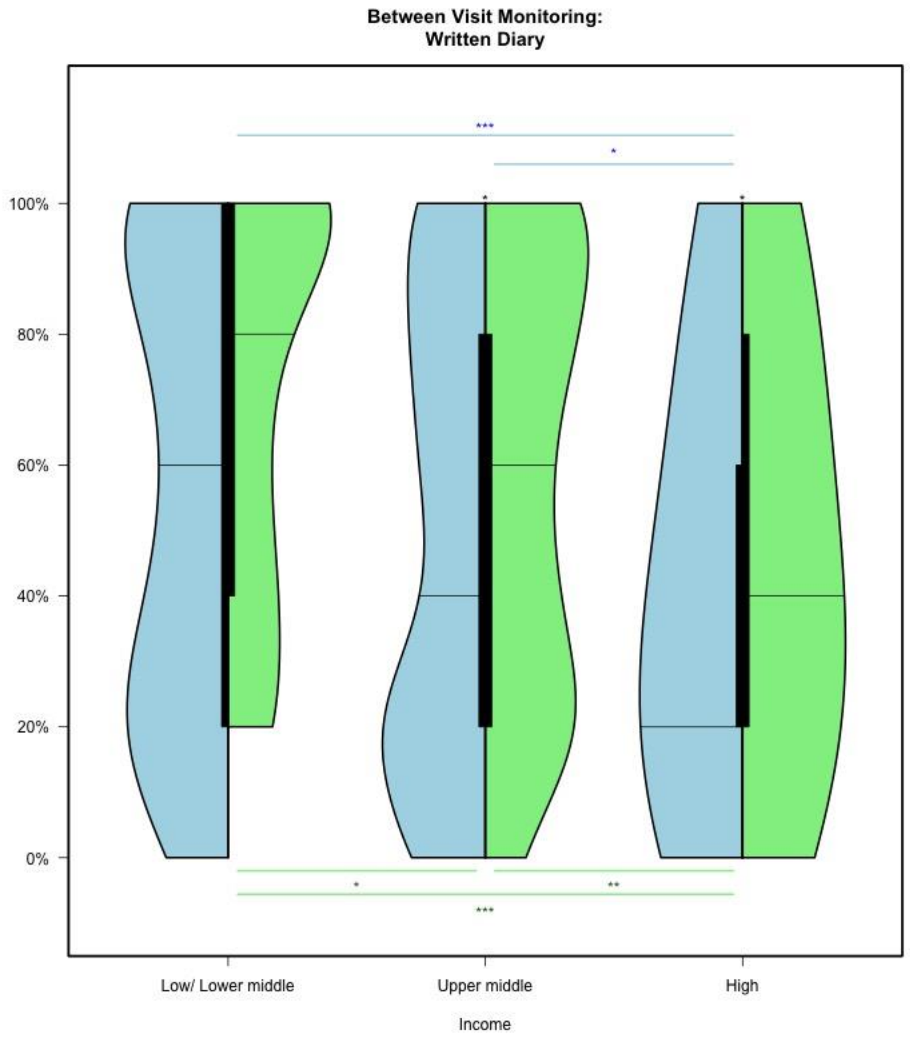

© 2023 Papadopoulos NG et al. *JAMA Network Open.*

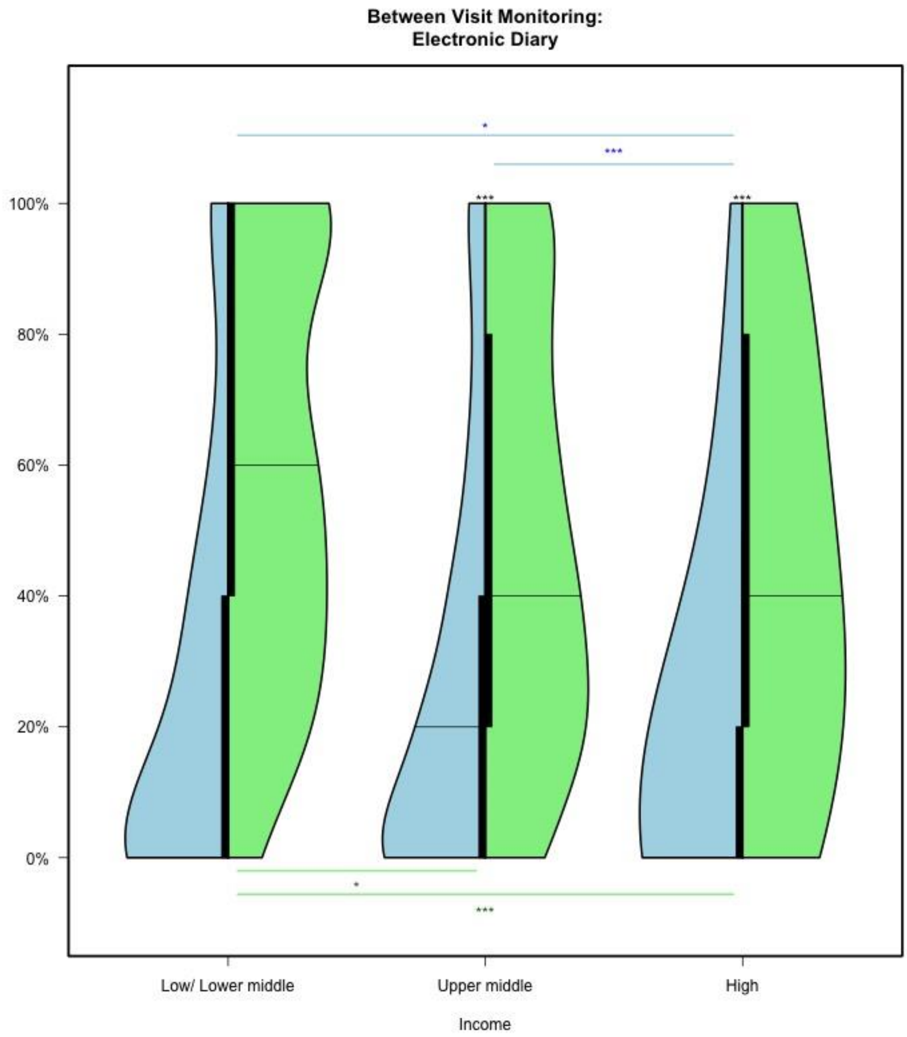

© 2023 Papadopoulos NG et al. *JAMA Network Open.*

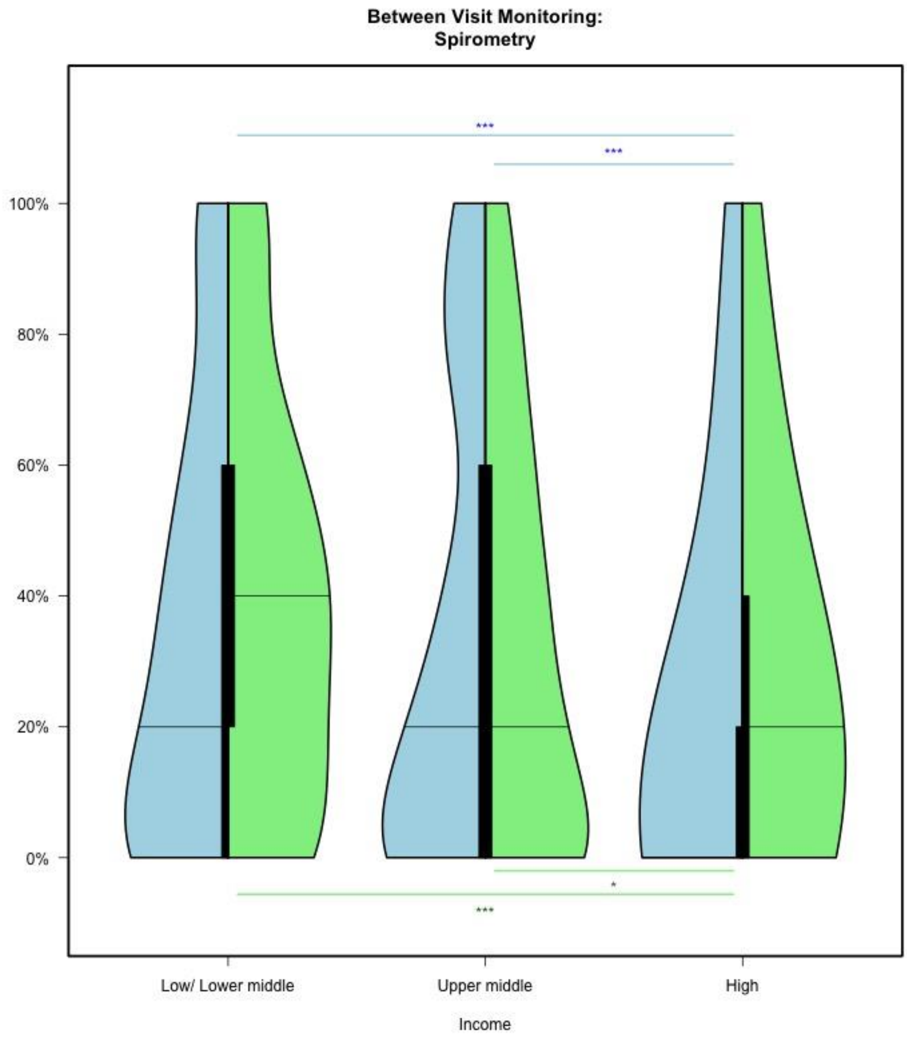

© 2023 Papadopoulos NG et al. *JAMA Network Open.*

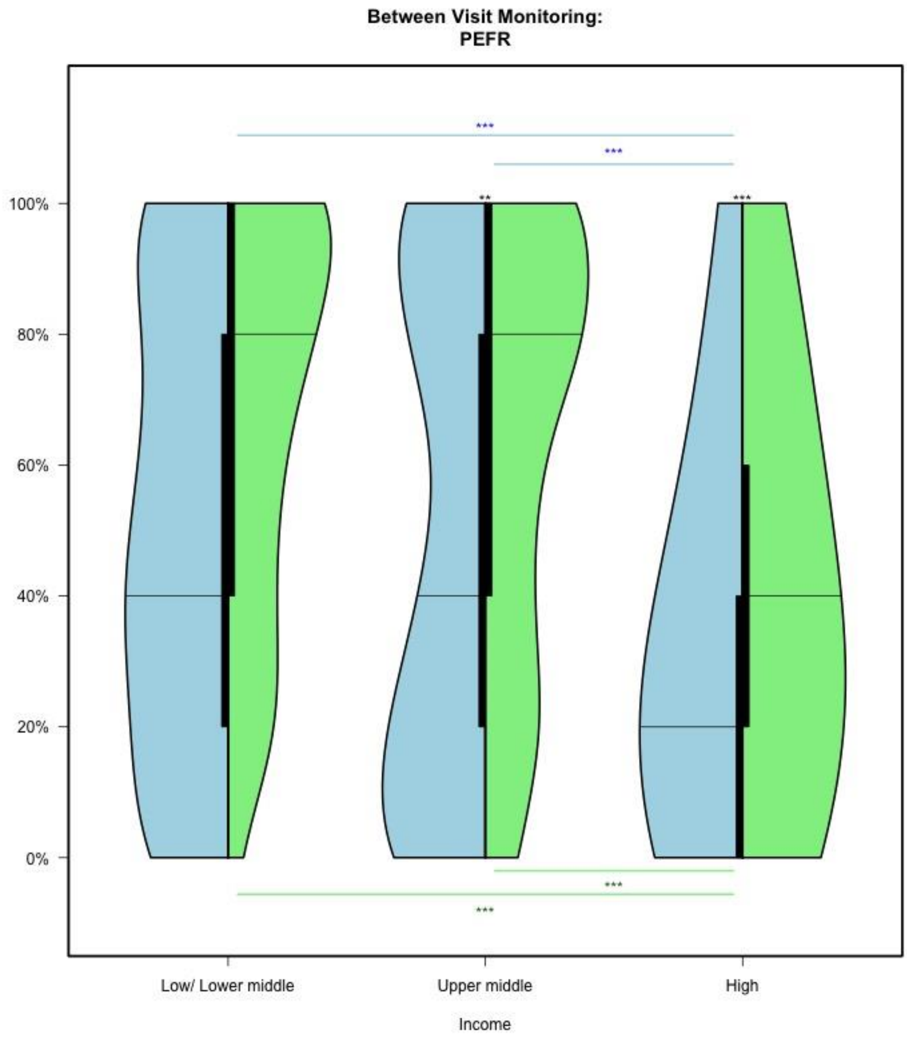

© 2023 Papadopoulos NG et al. *JAMA Network Open.*

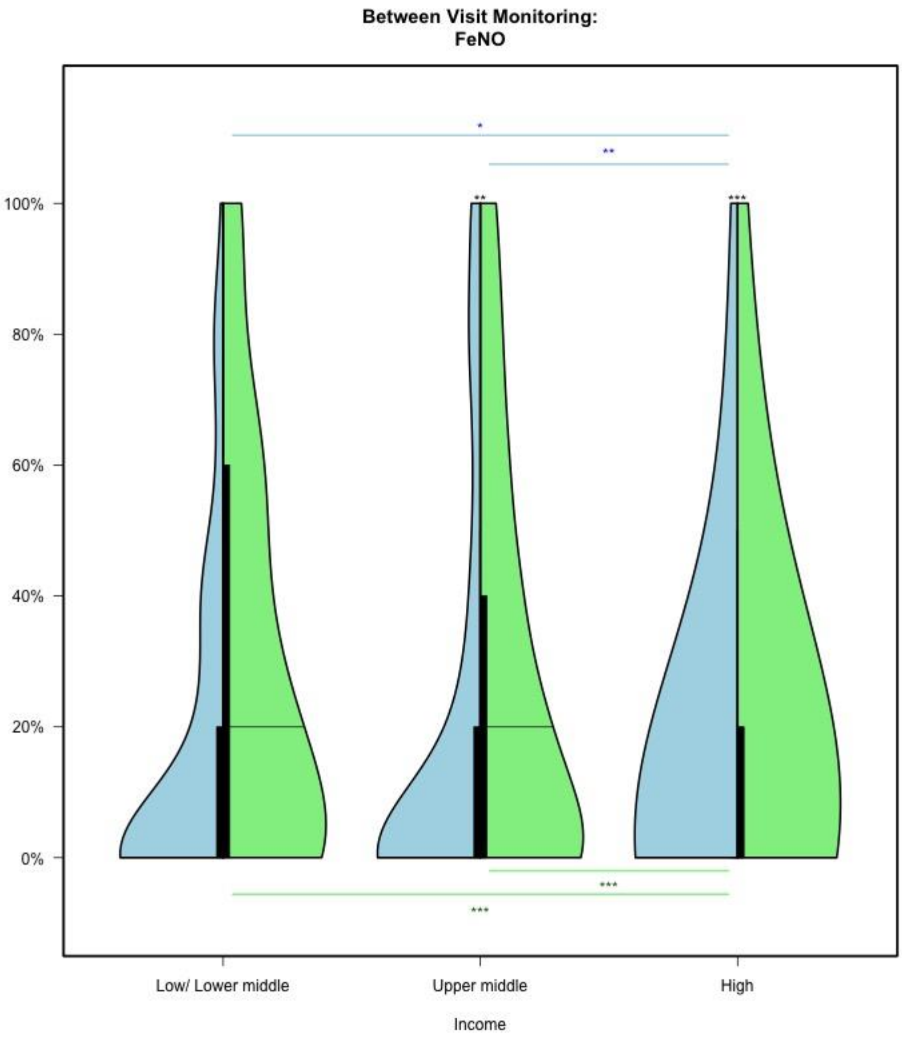

© 2023 Papadopoulos NG et al. *JAMA Network Open.*

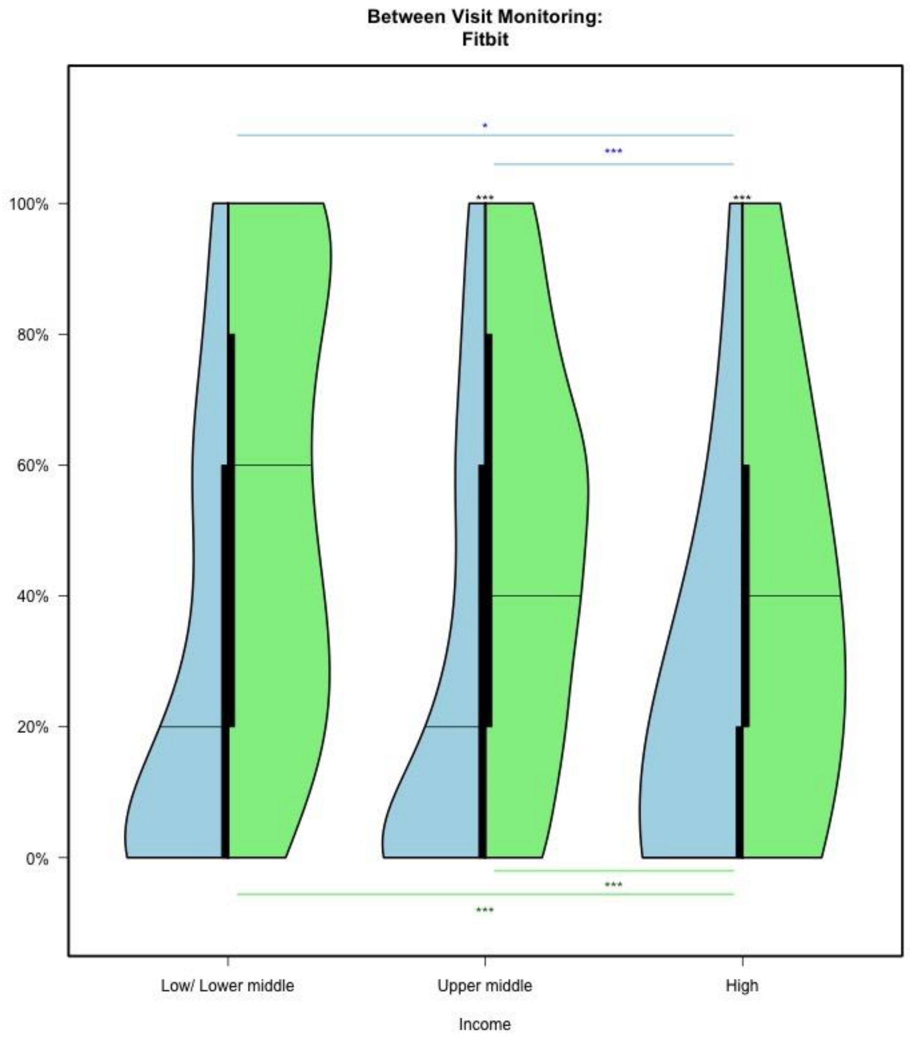

© 2023 Papadopoulos NG et al. *JAMA Network Open.*

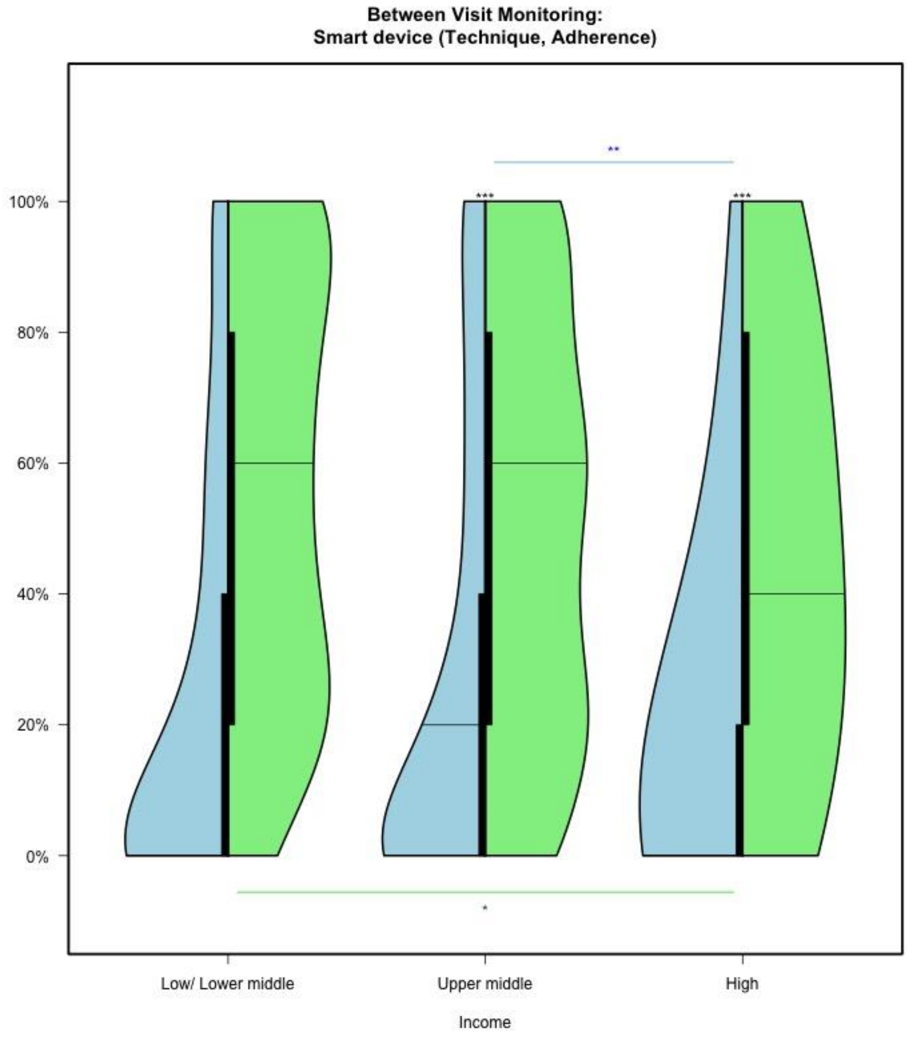

© 2023 Papadopoulos NG et al. *JAMA Network Open.*

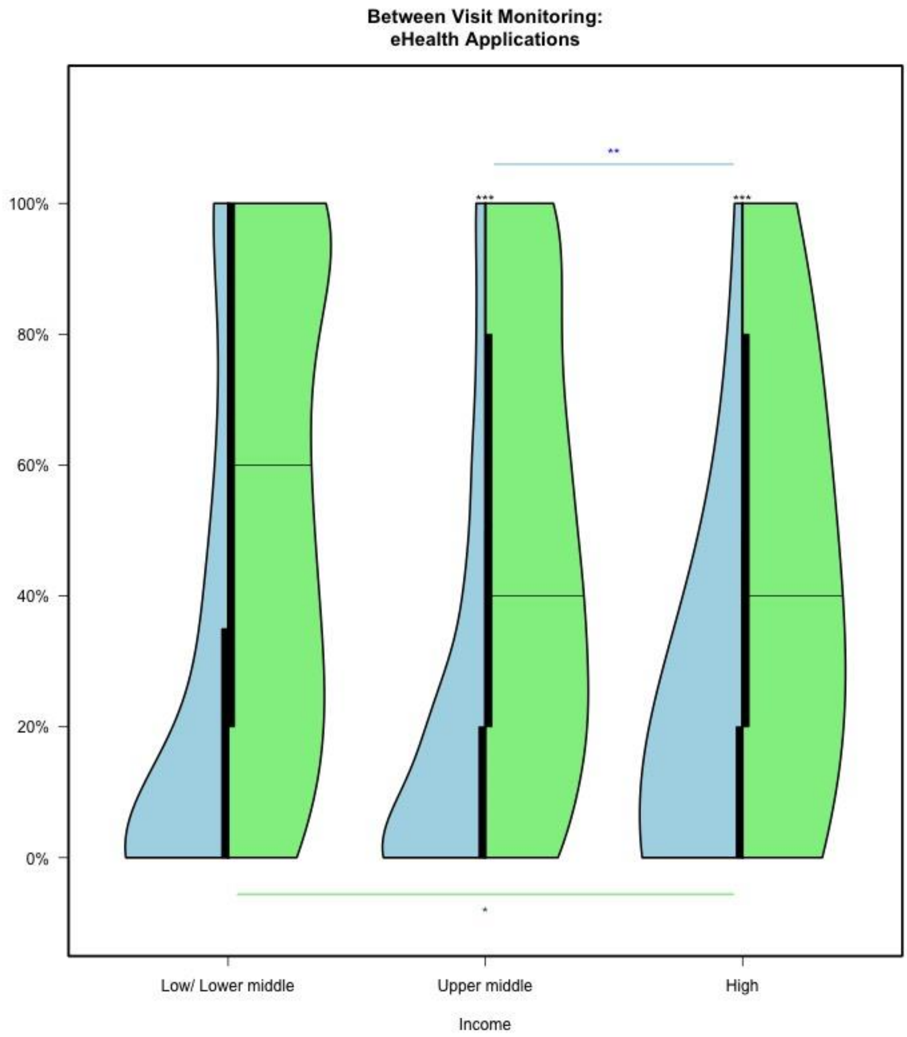

© 2023 Papadopoulos NG et al. *JAMA Network Open.*

eFigure 17. Actual (Light Blue, Left Side of the Violin Plots) and Perceived Optimal (Green, Right Side of the Violin Plots) Use of Monitoring Tools Between Asthma Visits, Stratified by Care Setting

Clinicians were asked to report on the approximate percentage of patients they recommend each of the monitoring tools. *: p<0.05; **: p<0.01; ***: p<0.001

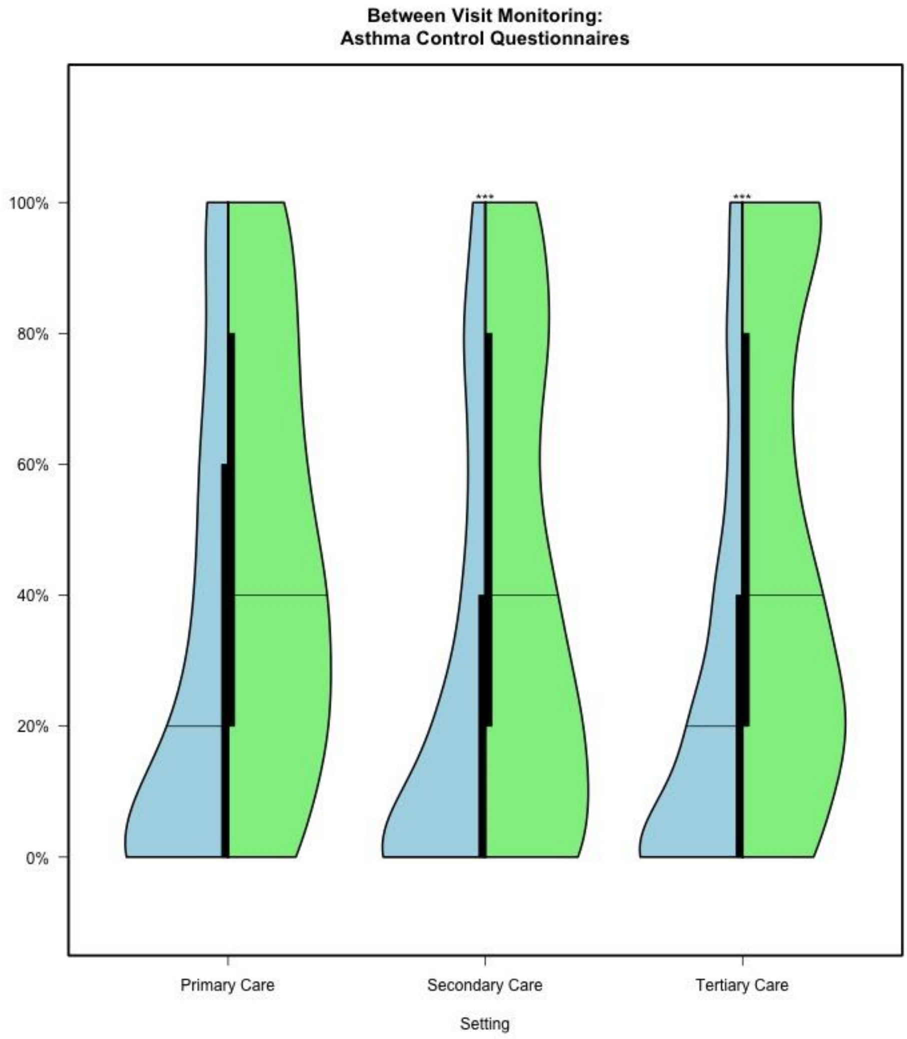

© 2023 Papadopoulos NG et al. *JAMA Network Open.*

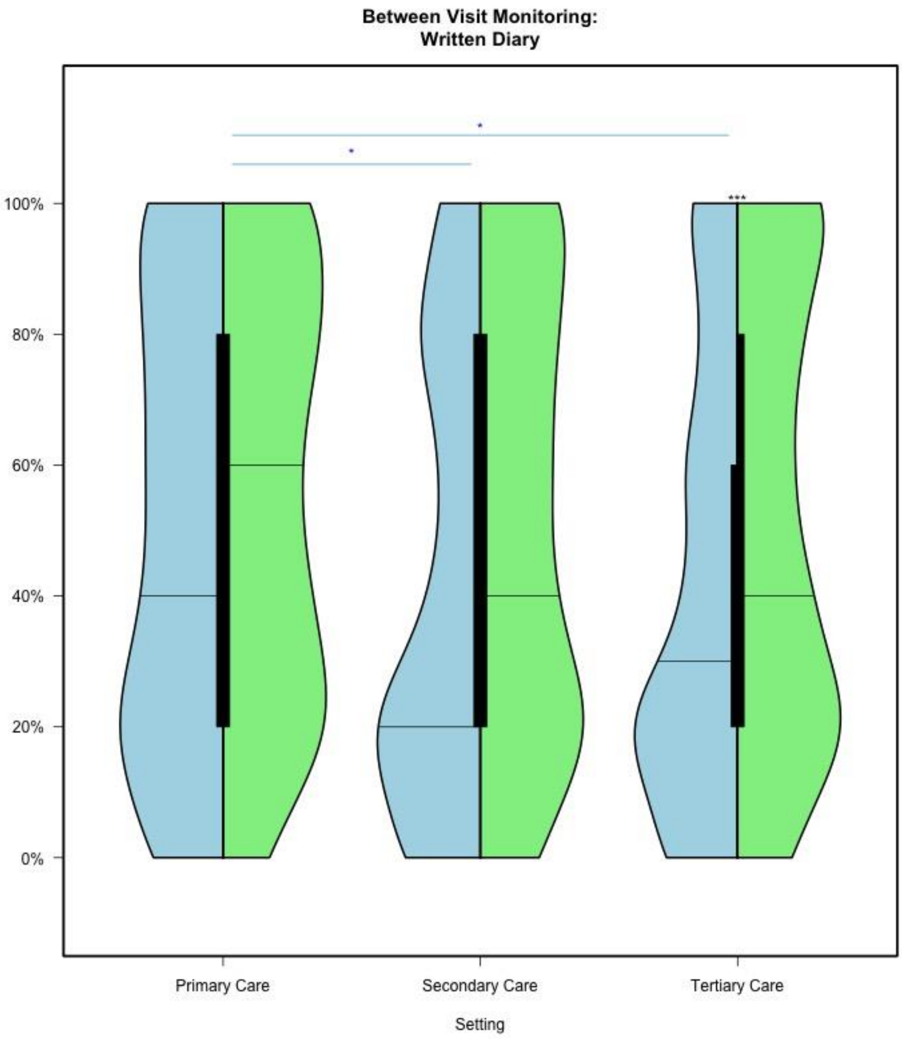

© 2023 Papadopoulos NG et al. *JAMA Network Open.*

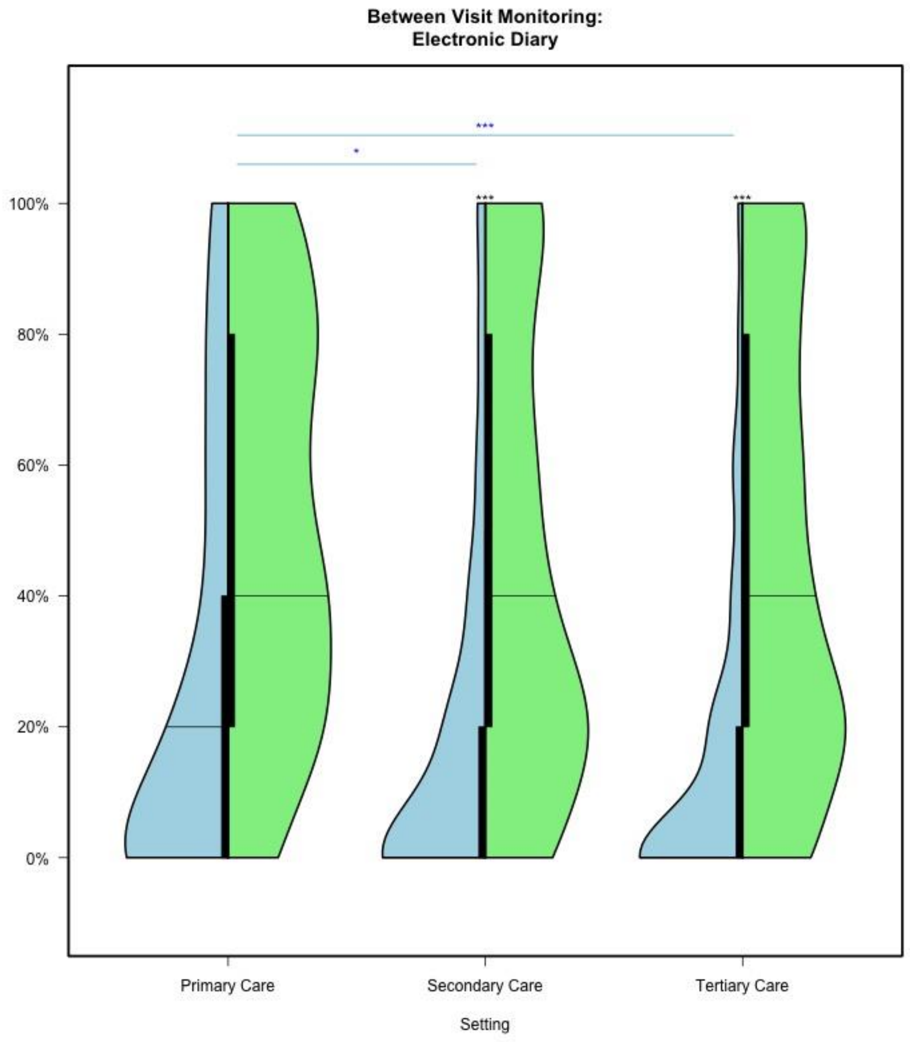

© 2023 Papadopoulos NG et al. *JAMA Network Open.*

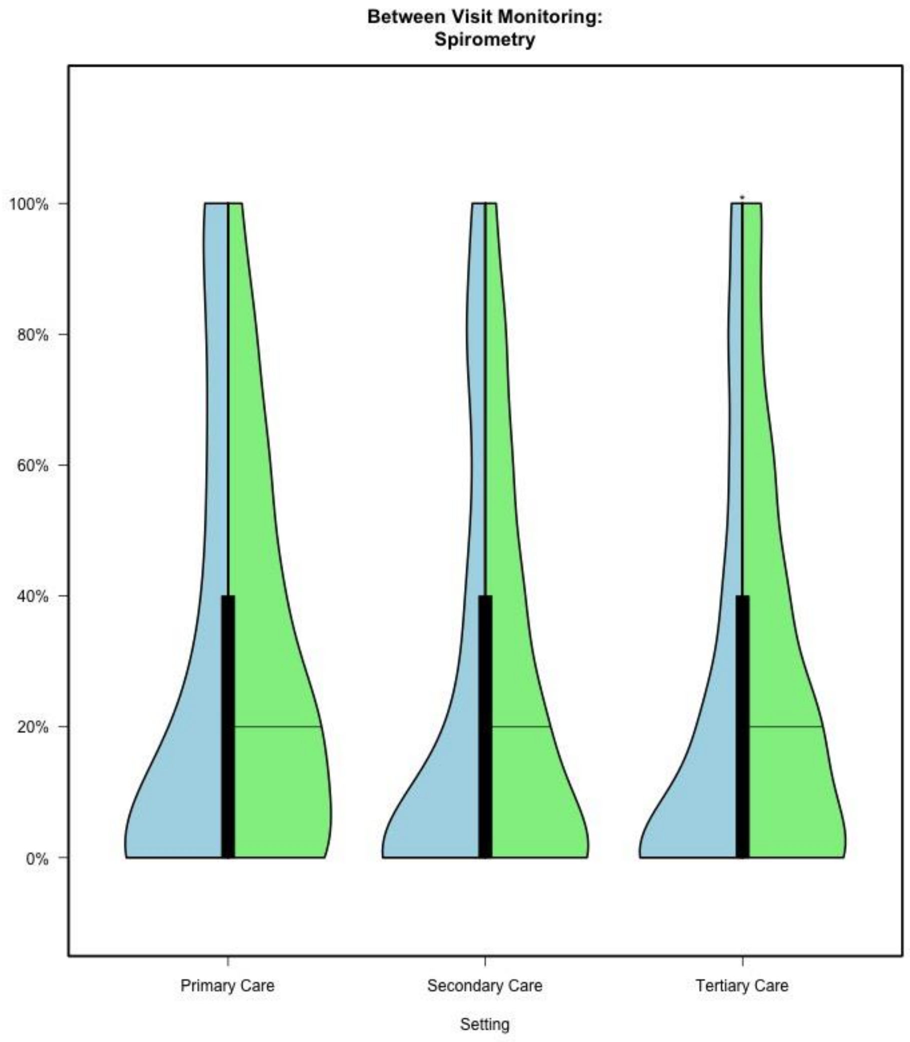

© 2023 Papadopoulos NG et al. *JAMA Network Open.*

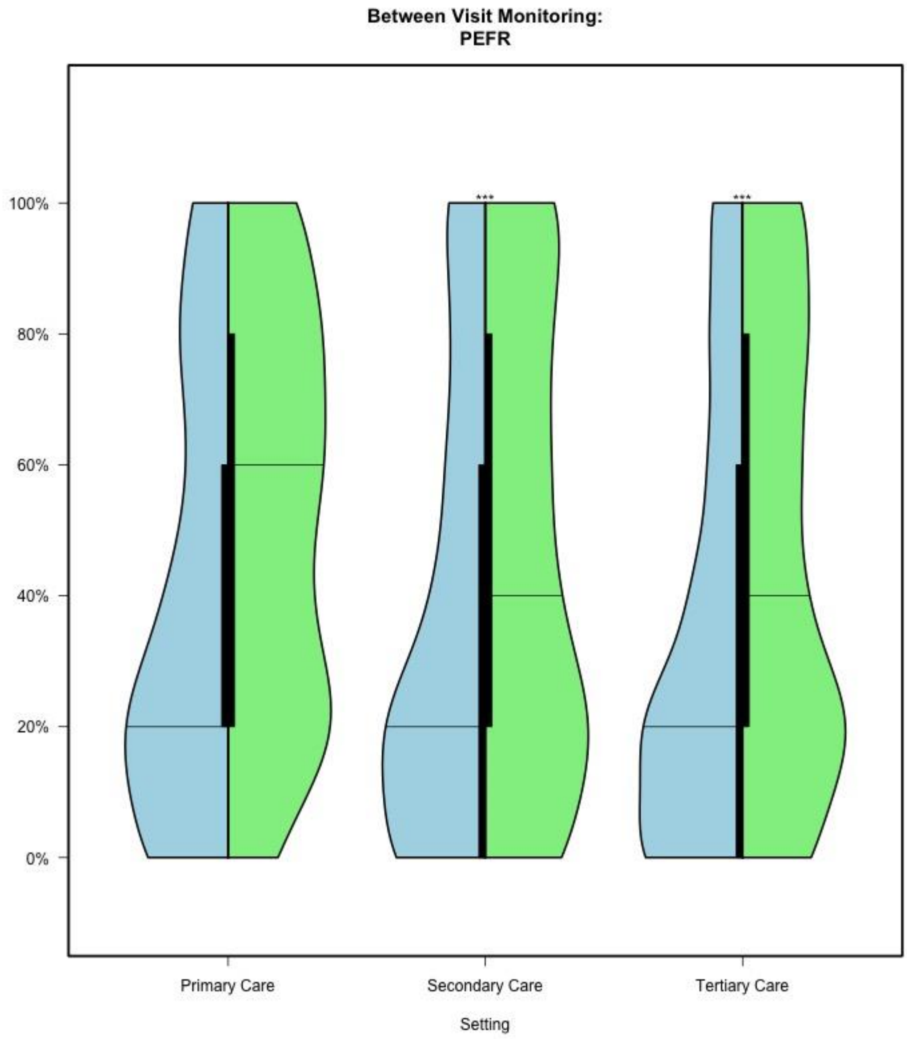

© 2023 Papadopoulos NG et al. *JAMA Network Open.*

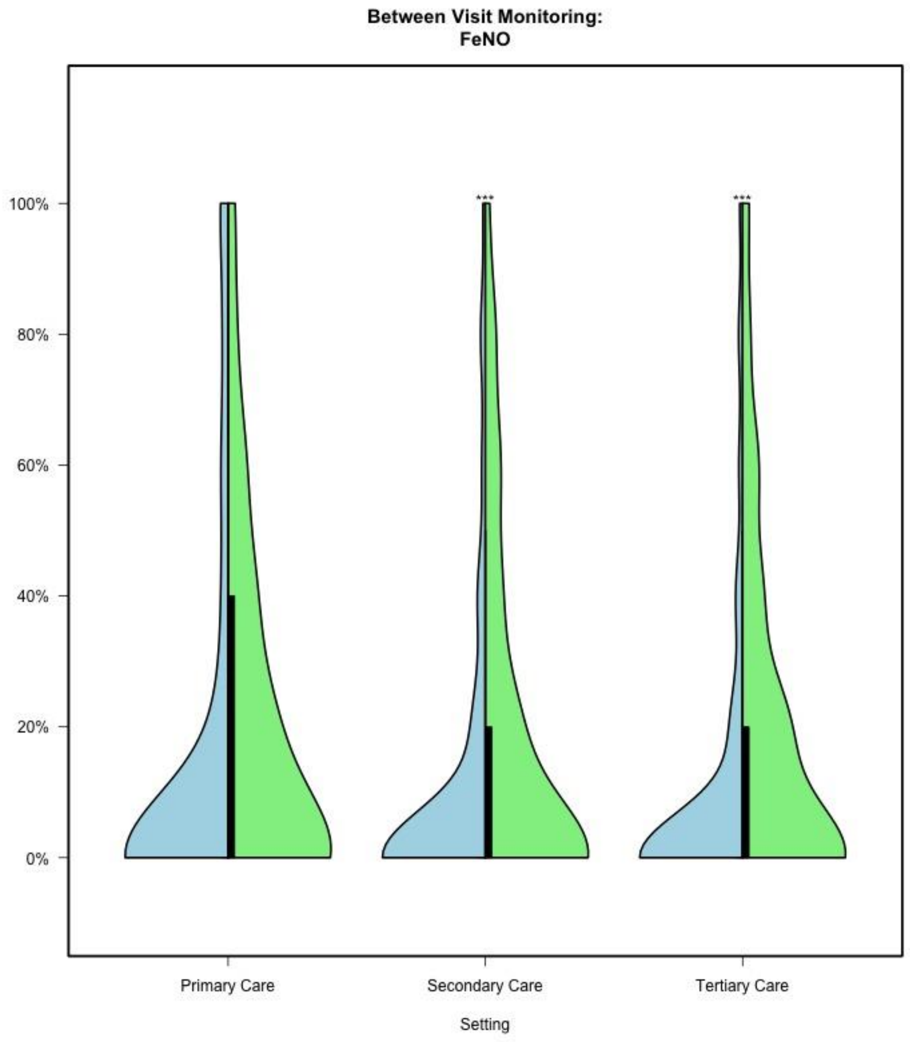

© 2023 Papadopoulos NG et al. *JAMA Network Open.*

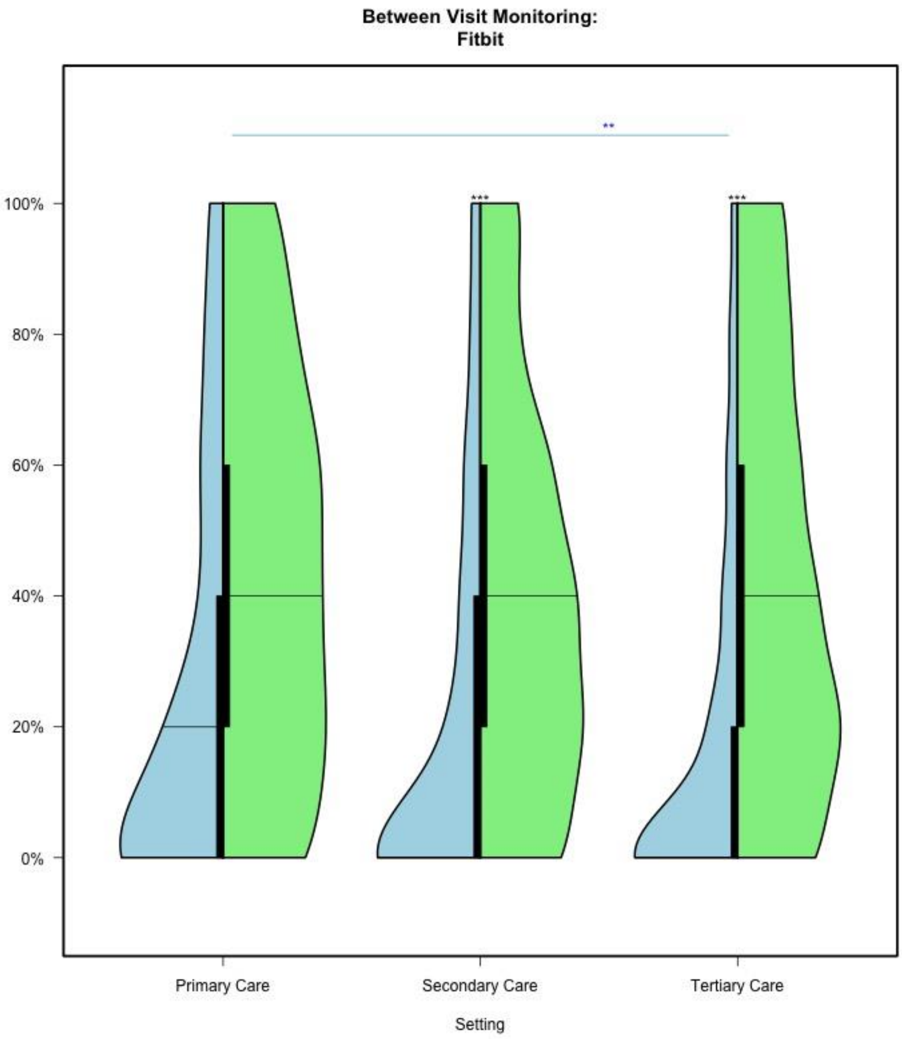

© 2023 Papadopoulos NG et al. *JAMA Network Open.*

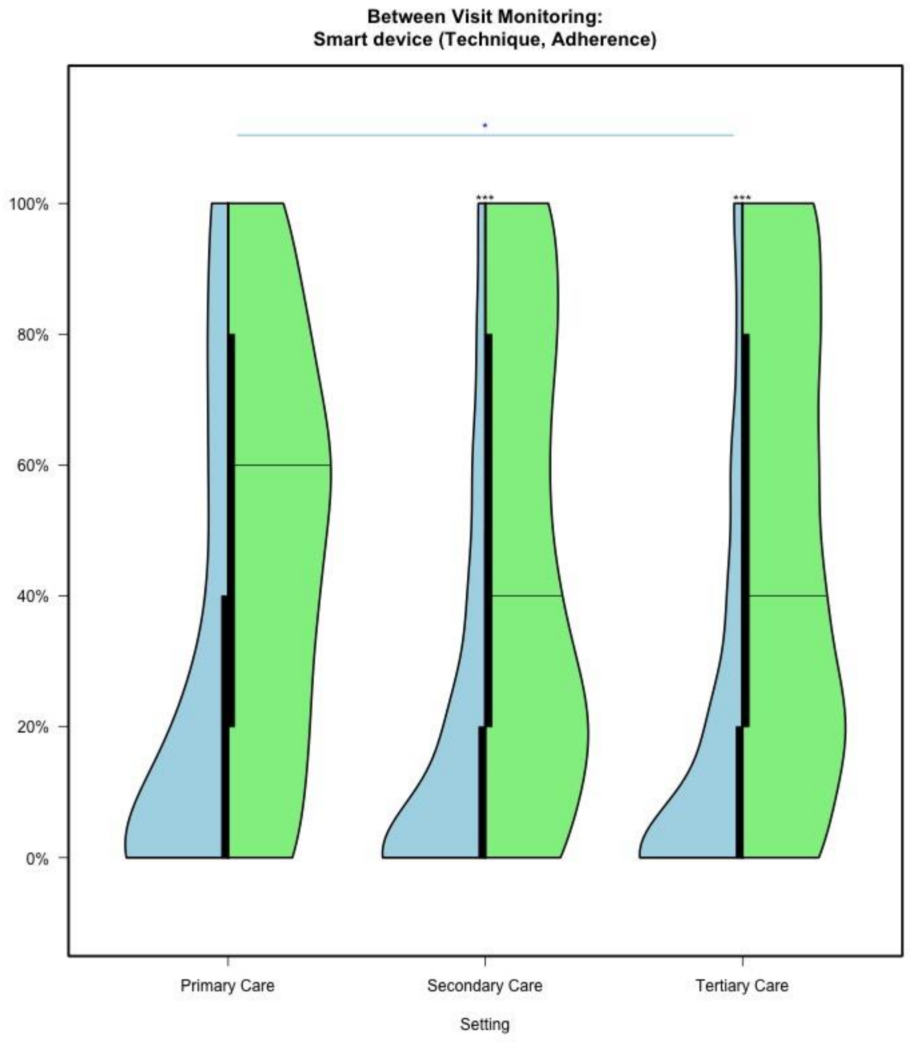

© 2023 Papadopoulos NG et al. *JAMA Network Open.*

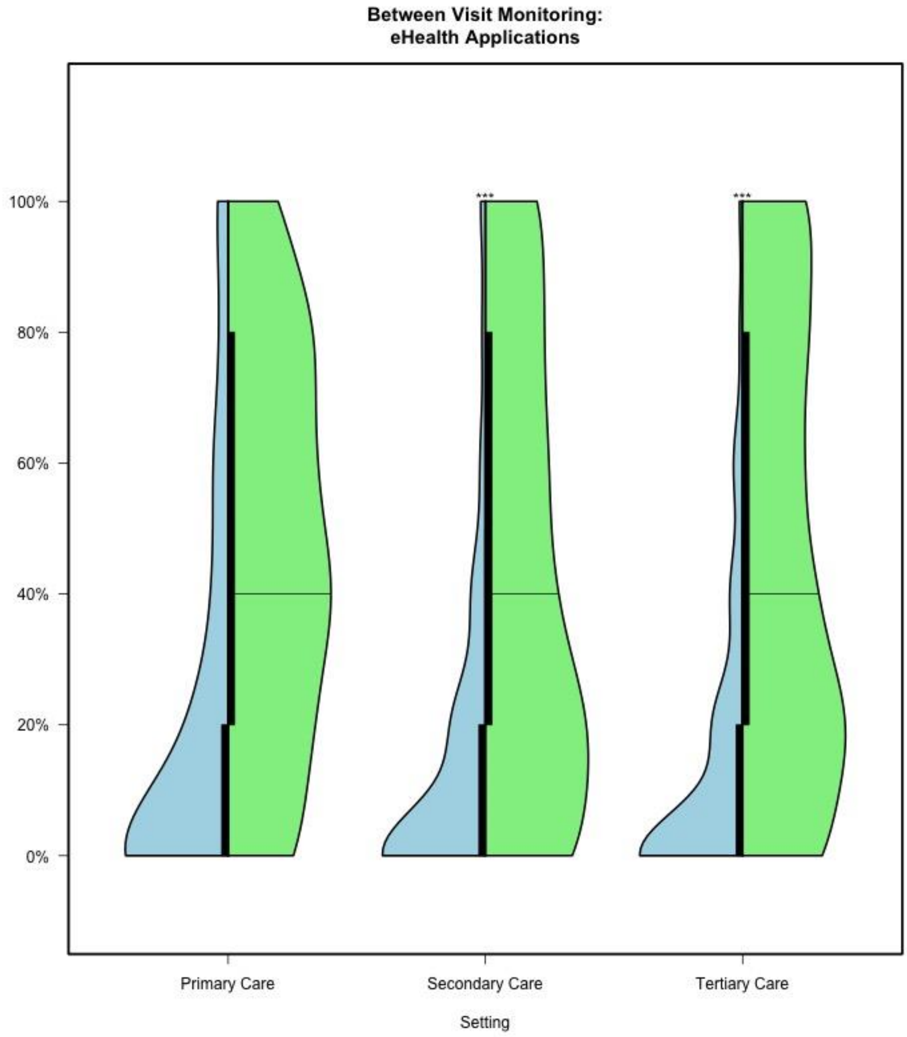

© 2023 Papadopoulos NG et al. *JAMA Network Open.*

Identified priorities for monitoring during visits

eBox. Identified Priorities for Monitoring During Visits

Every visit
Symptoms, control, comorbidities, adherence, education, growth

Almost every visit
Lung function (spirometry)
Quality of life
Exposures

Occasional performance
Medication side effects (adrenal function, ophthalmological, bone density)
Allergy, Airway inflammation (FeNO)
Psychological parameters, Diet, Exercise responsiveness

© 2023 Papadopoulos NG et al. *JAMA Network Open.*
