# Supplementary material for: Current and Optimal Practices in Childhood Asthma Monitoring Among Multiple International Stakeholders
Source: JAMA Netw Open. 2023 May 12;6(5):e2313120. doi: 10.1001/jamanetworkopen.2023.13120 (PMC10182430; doi:10.1001/jamanetworkopen.2023.13120)
Supplement: Supplement 2. — Nonauthor Collaborators [file jamanetwopen-e2313120-s002.pdf]

Supplemental Online Content: Nonauthor Collaborators
*First name, last name, and suffix (if applicable) are required and will appear in PubMed.

*Group Name(s): PeARL Think Tank							
***First Name and Middle Initial(s)**	***Last Name**	***Suffix (eg, Jr, III)**	Academic Degrees	Institution	Location (city, state/province, country)	Role or Contribution, eg, chair, principal investigator	Group (if more than 1 Group listed in the byline) and/or Subgroup (eg, Steering Committee)
Ioana	Agache		MD, PhD	Allergy & Clinical Immunology, Transylvania University, Brasov, Romania.	Brasov, Romania		
Stefania	Arasi		MD, PhD	Division of Allergy, University Department of Pediatrics, Pediatric Hospital Bambino Gesù, Rome, Vatican City, Italy	Rome, Italy		
Zeinab Awad	Awad El-Sayed		MD	Pediatric Allergy and Immunology Unit, Children's Hospital, Ain Shams University, Cairo, Egypt.	Cairo, Egypt.		
Leonard	Bacharier		MD	Division of Allergy, Immunology, and Pulmonary Medicine, Department of Pediatrics, Washington University, St. Louis, USA	St. Louis, USA		
Matteo	Bonini		MD, PhD	Department of Cardiovascular and Thoracic Sciences, Università Cattolica del Sacro Cuore, Fondazione Policlinico Universitario A. Gemelli – IRCCS, Rome, Italy	Rome, Italy		
Jose A	Castro-Rodriguez		MD, PhD	Department of Pediatrics, School of Medicine, Pontifical Universidad Catolica de Chile, Santiago, Chile.	Santiago, Chile.		
Zhimin	Chen		MD, PhD	Pulmonology Department, Children's Hospital Zhejiang University School of Medicine	Zhejiang, China		
Michael	Clausen		MD	Children's Hospital, Landspitali University Hospital, Reykjavik, Iceland	Reykjavik, Iceland		

Supplemental Online Content: Nonauthor Collaborators
*First name, last name, and suffix (if applicable) are required and will appear in PubMed.

*First Name and Middle Initial(s)	*Last Name	*Suffix (eg, Jr, III)	Academic Degrees	Institution	Location (city, state/province, country)	Role or Contribution, eg, chair, principal investigator	Group (if more than 1 Group listed in the byline) and/or Subgroup (eg, Steering Committee)
Timothy	Craig		DO	Department of Allergy and Immunology, Penn State University, State College, PA, USA	PA, USA		
Zuzana	Diamant		MD, PhD	Department of Clinical Pharmacy & Pharmacology, University of Groningen, University Medical Center of Groningen and QPS-NL, Groningen, Netherlands	Groningen, Netherlands		
Francine M	Ducharme		MD, MSc, FRCPC, CAHS	Department of Pediatrics, University of Montreal, Department of Social and Preventive Medicine, University of Montreal, Canada	Montreal, Canada		
Philippe	Eigenmann		MD	Department of Women-Children-Teenagers, University Hospital of Geneva, Geneva, Switzerland	Geneva, Switzerland		
Wojciech	Feleszko		MD, PhD	Department of Department of Pediatric Respiratory Diseases and Allergy, The Medical University of Warsaw, Poland.	Warsaw, Poland		
Vincezo	Fierro		MD	Division of Allergy, University Department of Pediatrics, Pediatric Hospital Bambino Gesù, Rome, Vatican City, Italy	Rome, Italy		
Alessandro	Fiocchi		MD	Allergy Department, Bambino Gesù Children’s Hospital, Roma – Italy.	Rome, Italy		
Luis	Garcia-Marcos		MD, PhD	Pediatric Respiratory and Allergy Units, “Virgen de la Arrixaca” Children's University Clinical Hospital, University of Murcia, Spain	Murcia, Spain		

Supplemental Online Content: Nonauthor Collaborators
*First name, last name, and suffix (if applicable) are required and will appear in PubMed.

***First Name and Middle Initial(s)**	***Last Name**	***Suffix (eg, Jr, III)**	Academic Degrees	Institution	Location (city, state/province, country)	Role or Contribution, eg, chair, principal investigator	Group (if more than 1 Group listed in the byline) and/or Subgroup (eg, Steering Committee)
James E	Gern		MD	Department of Pediatrics and Medicine, University of Wisconsin School of Medicine and Public Health, Madison, USA	Wisconsin, USA		
Anne	Goh		MD	Department of Pediatric Respiratory Medicine, KK Women's and Children's Hospital, Singapore	Singapore		
René	Maximiliano Gómez		MD, PhD	Head, Research & Education, Ayre Foundation. Salta, Argentina.	Salta, Argentina		
Maia	Gotua		MD	Center of Allergy and Immunology, Tbilisi, Georgia	Tbilisi, Georgia		
Eckard	Hamelmann		MD, PhD	Children’s Center Bethel, EvKB, University Bielefeld, Germany	Bielefeld, Germany		
Gunilla	Hedlin		MD, PhD	Paediatric Allergy, Centre for Allergy Research, Karolinska Institutet, Sweden.	Stockholm, Sweden		
Elham M	Hossny		MD, PhD	Pediatric Allergy and Immunology Unit, Children's Hospital, Ain Shams University, Cairo, Egypt.	Cairo, Egypt		
Zhanat	Ispayeva		MD, PhD	Department of Allergology and Clinical Immunology of the Kazakh National Medical University, Kazakhstan	Almaty, Kazakhstan		
Tuomas	Jartti		MD	Department of Pediatrics and Adolescent Medicine, Turku University Hospital and University of Turku, Finland.	Turku, Finland		
Miloš	Jeseňák		MD., MSc., PhD., MBA, Dott.Ric., MHA, FAAAAI	Department of Paediatrics, University Hospital in Martin, Slovak republic	Martin, Slovak republic		

Supplemental Online Content: Nonauthor Collaborators
*First name, last name, and suffix (if applicable) are required and will appear in PubMed.

*First Name and Middle Initial(s)	*Last Name	*Suffix (eg, Jr, III)	Academic Degrees	Institution	Location (city, state/province, country)	Role or Contribution, eg, chair, principal investigator	Group (if more than 1 Group listed in the byline) and/or Subgroup (eg, Steering Committee)
Omer	Kalayci		MD	Pediatric Allergy and Asthma Unit, Hacettepe University School of Medicine, Ankara, Turkey.	Ankara, Turkey		
Alan	Kaplan		MD, CCFP(EM), FCFP	Chair Family Physician Airways Group of Canada	Toronto, Canada		
Jon	Konradsen		MD, PhD	Astrid Lindgren Children´s Hospital, Karolinska University Hospital, Stockholm, Sweden.	Stockholm, Sweden		
Piotr	Kuna		MD, PhD	Department of Internal Medicine, Asthma and Allergy, Medical University of Lodz, Poland.	Lodz, Poland		
Susanne	Lau		MD, PhD	Charité Universitätsmedizin Berlin, Pediatric Pulmonology, Immunology and Intensive Care Medicine, Germany.	Berlin, Germany		
Peter	Le Souef		MBBS(WA), MD, MRCP(UK), FRACP	School of Paediatrics & Child Health, Faculty of Medicine, Dentistry and Health Sciences, University of Western Australia.	Perth, Australia		
Robert F	Lemanske		MD FAAAAI	Departments of Pediatrics and Medicine, University of Wisconsin School of Medicine and Public Health, Madison, Wis.	Madison, Wisconsin		
Mika J	Makela		MD, PhD	Department of Allergy, Helsinki University Central Hospital, Finland	Helsinki, Finland		
Paolo M	Matricardi		MD	Department of Pediatric Pulmonology, Immunology and Intensive Care Medicine, Charité - University Medicine Berlin, Germany.	Berlin, Germany		

*First name, last name, and suffix (if applicable) are required and will appear in PubMed.

***First Name and Middle Initial(s)**	***Last Name**	***Suffix (eg, Jr, III)**	Academic Degrees	Institution	Location (city, state/province, country)	Role or Contribution, eg, chair, principal investigator	Group (if more than 1 Group listed in the byline) and/or Subgroup (eg, Steering Committee)
Oleksandr	Mazulov		MD, PhD	Department of Pediatrics, National Pirogov Memorial Medical University	Vinnytsya,Ukraine		
Michael	Miligkos		MD, MSc, PhD	Allergy Department, 2nd Paediatric Clinic, National and Kapodistrian University of Athens, Athens, Greece	Athens, Greece		
Mário	Morais-Almeida		MD, MSc, PhD	Allergy Center, CUF Descobertas Hospital, Lisbon, Portugal.	Lisbon, Portugal		
Clare	Murray		MBChB, MD, MRCP, MRCPHCH	Division of Infection, Immunity and Respiratory Medicine, School of Biological Sciences, The University of Manchester, UK.	Manchester, UK		
Karthik	Nagaraju		MD	VN Allergy & Asthma Research Centre, Chennai, Tamilnadu, India.	Chennai, India		
Antonio	Nieto Garcia		MD, PhD	Pediatric Pulmonology & Allergy Unit Children’s Hospital la Fe, Spain.	Valencia, Spain		
Zoltan	Novak		MD	Department of Gynaecology, National Insitute of Oncology, Budapest, Hungary	Budapest, Hungary		
Ruby	Pawankar		MD	Department of Pediatrics, Nippon Medical School, Tokyo, Japan	Tokyo, Japan		
Marielle	Pijenburg		MD, PhD	Department of Pediatrics, division of Respiratory Medicine and Allergology, Rotterdam, The Netherlands	Rotterdam, Netherlands		
Helena	Pite		MD, PhD	Allergy Center, CUF Infante Santo Hospital, Lisbon, Portugal	Lisbon, Portugal		
Paulo MC	Pitrez		MD, PhD	Pediatric Pulmonology Division, Hospital Moinhos de Vento.	Porto Alegre, Brazil		

*First Name and Middle Initial(s)	*Last Name	*Suffix (eg, Jr, III)	Academic Degrees	Institution	Location (city, state/province, country)	Role or Contribution, eg, chair, principal investigator	Group (if more than 1 Group listed in the byline) and/or Subgroup (eg, Steering Committee)
Petr	Pohunek		MD, PhD, FCCP	Pediatric Pulmonology, Pediatric Department, 2nd Faculty of Medicine, Charles University, Prague, University Hospital Motol, Prague, Czech Republic.	Czech Republic		
David	Price		MB, BChir, MA, DRCOG, FRCGP	Centre of Academic Primary Care, Division of Applied Health Sciences.	Aberdeen, UK		
Alfred	Priftanji		MD, PhD	Dept. of Allergy, Mother Theresa School of Medicine, University of Tirana, Tirana, Albania	Tirana, Albania		
Valeria	Ramiconi			The European Federation of Allergy and Airways Diseases Patients' Associations (EFA)	Brussels, Belgium		
Daniela	Rivero Yeverino		MD	Benemérita Universidad Autónoma de Puebla, Hospital Universitario de Puebla, Puebla, México	Puebla, Mexico		
Graham C	Roberts		DM, MA, MSc, FRCPCH	Paediatric Allergy and Respiratory Medicine within Medicine at the University of Southampton.	Southampton, UK		
Aziz	Sheikh		BSc, MBBS, MSc, MD, FRCP, FRCPE, FFPH, FACMI, FFCI, FRSE, OBE, FMedSci	Asthma UK Centre for Applied Research, Usher Institute of Population Health Sciences and Informatics, The University of Edinburgh, Edinburgh, UK.	Edinburgh, UK		
Kun-Ling	Shen		MD	Department of Respiratory Medicine, Beijing Children’s Hospital, Capital Medical University; National Center for Children’s Health, China	Beijing, China		
Zsolt	Szepfalusi		MD	Division of Pediatric Pneumology, Department of Pediatrics, Medical University of Vienna	Vienna, Austria		

Supplemental Online Content: Nonauthor Collaborators
*First name, last name, and suffix (if applicable) are required and will appear in PubMed.

***First Name and Middle Initial(s)**	***Last Name**	***Suffix (eg, Jr, III)**	Academic Degrees	Institution	Location (city, state/province, country)	Role or Contribution, eg, chair, principal investigator	Group (if more than 1 Group listed in the byline) and/or Subgroup (eg, Steering Committee)
Ioanna	Tsiligianni		MD, PhD, MPH/HCM	Health Planning Unit, Department of Social Medicine, Faculty of Medicine, University of Crete, Crete, Greece	Heraklion, Greece		
Mirjana	Turkalj		MD, PhD	Srebrnjak Children's Hospital, Srebrnjak 100, 10000 Zagreb, Croatia	Zagreb, Croatia		
Steve	Turner		MBBS, MD, MRCP(UK), FRCPCH	Child Health, Royal Aberdeen Children’s Hospital and University of Aberdeen, Aberdeen, UK	Aberdeen, UK		
Tetiana	Umanets		MD	Institute of Pediatrics Ob&Gyn NAMS of Ukraine	Kyiv, Ukraine		
Arunas	Valiulis			Clinic of Children's Diseases, Institute of Clinical Medicine, Medical Faculty of Vilnius University, Vilnius, Lithuania.	Vilnius, Lithuania		
Susanne	Vijveberg		MD, PhD	Department of Paediatric Pulmonology, Amsterdam Public Health Research Institute, Amsterdam University Medical Centers, University of Amsterdam, Amsterdam, The Netherlands	Amsterdam, The Netherlands		
Jiu-Yao	Wang		MD, PhD	China Medical University Children's Hospital Taichung Taiwan	Taiwan		
Ran	Wang		MD	Division of Infection, Immunity and Respiratory Medicine, School of Biological Sciences, Faculty of Biology, Medicine and Health, The University of Manchester, Manchester, UK	Manchester, UK		
Tonya	Winders		MBA	Allergy and Asthma Network, Vienna, Virginia, USA	Virginia, USA		

Supplemental Online Content: Nonauthor Collaborators
*First name, last name, and suffix (if applicable) are required and will appear in PubMed.

***First Name and Middle Initial(s)**	***Last Name**	***Suffix (eg, Jr, III)**	Academic Degrees	Institution	Location (city, state/province, country)	Role or Contribution, eg, chair, principal investigator	Group (if more than 1 Group listed in the byline) and/or Subgroup (eg, Steering Committee)
Dong Keon	Yon		MD, FACAAI, FAAAAI	Department of Pediatrics, CHA Bundang Medical Center, CHA University School of Medicine, Seongnam, Korea	Seongnam, Korea		
Osman M	Yusuf		MD	The Allergy & Asthma Institute, Pakistan	Karachi, Pakistan		
Heather J	Zar		MD, PhD	Department of Pediatrics & Child Health, Director MRC Unit on Child & Adolescent Health, Red Cross War Memorial Children's Hospital, University of Cape Town.	Cape Town, South Africa		
