# Supplementary material for: Current and Optimal Practices in Childhood Asthma Monitoring Among Multiple International Stakeholders
Source: JAMA Netw Open. 2023 May 12;6(5):e2313120. doi: 10.1001/jamanetworkopen.2023.13120 (PMC10182430; doi:10.1001/jamanetworkopen.2023.13120)
Supplement: Supplement 3. — Data Sharing Statement [file jamanetwopen-e2313120-s003.pdf]

Data Sharing Statement

Papadopoulos. Current and Optimal Practices in Childhood Asthma Monitoring Among Multiple International Stakeholders. *JAMA Netw Open*. Published May 12, 2023.
doi:10.1001/jamanetworkopen.2023.13120

Data
Data available: Yes
Data types: Data (not involving human participants)
How to access data: Available upon request to nikpap@med.uoa.gr
When available: With publication

Supporting Documents
Document types: Statistical/analytic code
How to access documents: Available upon request to nikpap@med.uoa.gr
When available: With publication

Additional Information
Who can access the data: Researchers with proposed study plan, after approval from the PeARL Steering Committee
Types of analyses: Any purpose
Mechanisms of data availability: After approval
